# An annotated nomenclatural checklist of endemic vascular plants distributed in the Ukrainian Carpathians

**DOI:** 10.3897/BDJ.11.e103921

**Published:** 2023-08-11

**Authors:** Andriy Novikov

**Affiliations:** 1 State Museum of Natural History of the NAS of Ukraine, Lviv, Ukraine State Museum of Natural History of the NAS of Ukraine Lviv Ukraine

**Keywords:** GBIF, IPNI, CoL, taxonomic databases, endemic flora, Ukrainian Carpathians, nomenclature, *Sabulinapauciflora*, *comb. nov*.

## Abstract

**Background:**

The current paper presents a nomenclatural checklist for vascular plants validated being (sub)endemic to and present in the flora of the Ukrainian Carpathians. This checklist is a part of the work targeted on an inventory of endemic plants distributed in the Ukrainian Carpathians. It is mainly based on the analysis of primary sources (i.e. original protologues and monographic works), but also uses the data provided in the recent online taxonomic aggregators, such as the Global Biodiversity Information Facility (GBIF), Catalogue of Life (CoL), Plants of the World Online (POWO), Euro+Med PlantBase, World Flora Online (WFO) and others. Over 7,000 specimens deposited in the leading Ukrainian herbaria were also revised and used as a supporting data source during the work on the checklist.

**New information:**

The checklist provides a revised nomenclature, including corrections on publication dates, rediscovered taxonomic protologues, corrected authorships and revised taxonomic status for (sub)endemic (sub)species of vascular plants occurring in the Ukrainian Carpathians. It contains 1,101 names, from which 78 species and subspecies have been accepted as valid and 1023 species and infraspecific taxa are provided as synonyms. It is completed with critical notes on the nomenclature of problematic taxa and brief annotations regarding their distribution in the Ukrainian Carpathians, indicating the endemicity range and sozological status for all analysed (sub)species.

The current checklist is linked with the GBIF taxonomic backbone, provides notes on detected issues and primarily focuses on its update and correction of the nomenclatural issues and taxonomic inconsistencies, but also aims at discussing issues in other popular taxonomic databases.

*Sabulinapauciflora* is proposed as a new combination to comply with a recent revision of the genus *Sabulina*.

## Introduction

The Ukrainian Carpathians are part of the Eastern Carpathians located on the territory of four western regions (i.e. Lviv, Ivano-Frankivsk, Zakarpattia and Chernivtsi) of Ukraine. These mountains stretch for over 280 km and cover about 24,000 km^2^ (Kondracki 1989, Tasenkevich 2004, Novikov 2021). The floristic diversity of the Ukrainian Carpathians consists of over 2500 species and subspecies of vascular plants. It comprises nearly 50% of the flora of the whole Carpathian Mountains range and almost 39% of the entire flora of Ukraine (Tasenkevich 2003, Chopyk and Fedoronchuk 2015). This is one of the most important centres of floristic diversity in Ukraine, having several confirmed glacial refugia and hosting many rare, relict and endemic plant species (Malynovskiy et al. 2002, Kricsfalusy and Budnikov 2007, Mitka et al. 2014). Amongst them, many species have minimal distribution. Some authors report 125 or even more endemic plant taxa from the region, including stenoendemics and microtaxa (Stojko and Tasenkevich 1993, Tasenkevich 2003, Chorney 2006, Chorney 2011).

During our initial analysis, 70 endemic and subendemic taxa (species and subspecies) of vascular plants were selected as those taxonomically non-ambiguous and confirmed as present in the flora of the Ukrainian Carpathians (Novikoff and Hurdu 2015). Since that, the initial list has been critically revised, based on newly-available published sources and routine elaboration of herbarium material. As a result, nine taxa (i.e. Aconitumfirmumsubsp.fussianum Starmühl., *Leontodonkulczynskii* Popov, *Oxytropiscarpatica* R.Uechtr., *Trisetummacrotrichum* Hack., CarduuskerneriSimk.subsp.kerneri, Dactylorhizamaculata(L.)Soósubsp.schurii (Klinge) Soó, Dianthuscarthusianorumsubsp.tenuifolius (Schur) Hegi, *Euphorbiacarpatica* Woł. and FestucarupicolaHeuffel.subsp.saxatilis (Schur) Rauschert) were excluded from the initial list because their distribution was not limited to the Carpathian region and/or due to problematic taxonomic interpretation and chorology. Some more taxa (e.g. Astragalusaustralissubsp.krajinae (Domin) Domin, CarduuskerneriSimk.subsp.kerneri, *Erysimumwahlenbergii* (Asch. & Engl.) Borbás, Festucapsammophilasubsp.dominii (Krajina) P.Šmarda and Leucanthemopsisalpinasubsp.tatrae (Vierh.) Holub,) were considered as potential candidates for the current list, but excluded from further processing due to their unclear taxonomy and/or chorology. *Violajooi* Janka, which is a south-eastern Carpathian endemic, has also been excluded because it had been erroneously reported for the flora of the Ukrainian Carpathians (Sheliag-Sosonko et al. 1980, Chorney 2011). ScabiosacolumbariaL.subsp.pseudobanatica (Schur) Jáv. & Csapody mentioned in the initial list (Novikoff and Hurdu 2015) was excluded from the current list due to unclear taxonomic status and chorology. Seventeen taxa and their verified synonyms were newly added, so the current list contains 78 accepted species from 51 genera, 29 families, 15 orders and two classes of vascular plants supported with 1023 synonyms (including the orthographical variants).

During the work with herbarium material, the need for a comprehensive taxonomic checklist became apparent because many specimens were deposited under different names and had different taxonomic interpretations depending on the identifier. Unfortunately, available online databases and published sources did not entirely fill the gap in nomenclature and synonymy and sometimes even provide controversial interpretations. To solve this issue, the checklist with complete nomenclatural citations was compiled, based on comprehensive data analysis. I believe that this checklist will be useful for other scientists conducting taxonomic revisions of certain plant groups and it will fulfil the missing data in existing checklists and databases, especially those focused on biodiversity and conservation.

### Dedication

The work is dedicated to the bright memory of my teacher and friend, Prof. Dr. hab. Kazimierz Szczepanek from the Jagiellonian University in Kraków.

## Materials and methods

The accepted taxa’s names are typefaced in bold italicised font. The synonyms are typefaced in italicised font. The identity sign (≡) indicates homotypic synonyms. The equality sign (=) indicates the heterotypic regular synonyms. The N-dash sign (–) indicates the names with the types which do not belong to the present taxon, i.e. partial heterotypic synonyms (synonyms *pro parte*), names in the interpretation of a certain author (synonyms *sensu*) or misapplied names. The names applied in the Ukrainian herbaria are marked by an asterisk. Additional information required to clarify or better understand the provided name is indicated in the square brackets [].

In some cases, the same name has been published twice in the same year by the same author(s), but in different works or it was published first as *nomen nudum* and soon supported with comments and/or protologue. In such cases, extended nomenclature citation is provided with an indication of both such works delimited by the term ‘et’. The term ‘et’ is applied to avoid confusion with an ampersand (&), which delimits different authors of the same taxon. In other words, in the nomenclature citation, ampersand delimits authors, while the term ‘et’ delimits publications. For example, *Chrysanthemumrotundifolium* has been published by two authors, Franz Waldstein and Pál Kitaibel. Therefore its nomenclature citation is ‘*Chrysanthemumrotundifolium* Waldst. & Kit., Descr. Icon. Pl. Rar. Hung. 3: 262, t. 236 (1812)’. The name Thlaspidacicumsubsp.dacicum has been published by János Heuffel twice in the same year, but in two different journals – in the Oesterreichische botanische Zeitschrift and in the Verhandlungen der Kaiserlich-Koniglichen Zoologisch-Botanischen Gesellschaft in Wien. Hence, the providded nomenclature citation is ‘Thlaspidacicumsubsp.dacicum Heuff., Oesterr. Bot. Z. 8: 26 (1858) et Verh. K.K. Zool.-Bot. Ges. Wien 8 (Abh.): 61 (1858)’. The name *Silenetranssilvanica* was first published by Philipp Schur in 1858 and later, in 1860, supported with details in the same journal Oesterreichische botanische Zeitschrift as nomen nudum. Hence, the provided nomenclature citation is ‘*Silenetranssilvanica* Schur, Oesterr. Bot. Z. VIII: 22 (1858) [nom. nudum] et Oesterr. Bot. Z. 10: 181 (1860)’.

Recently, for the flora of Ukraine, the assessments following criteria of the International Union for Conservation of Nature (IUCN 2022) were conducted and published by Onyshchenko et al. (2022); ascertained threat categories are indicated here for all analysed (sub)species. The national threat categories follow the last edition of the Red Book of Ukraine (Didukh 2009).

## Data resources

The present checklist has been compiled in 2017–2022, based on the elaboration of such principal monographic works as Flora of USSR (Komarov 1934), Flora of UkrSSR (Bordzilovskiy 1938, Zerov 1950), Flora of the European Part of USSR (Fedorov 1974, Tzvelev 1989), Flora of the Ukrainian Carpathians (Chopyk and Fedoronchuk 2015), Flora of Poland (Szafer and Raciborski 1919, Szafer 1921, Szafer and Pawłowski 1955, Pawłowski 1963, Pawłowski and Jasiewicz 1971, Jasiewicz 1980, Jasiewicz 1985), Flora of Romania (Săvulescu 1952), Flora of Slovakia (Futák 1966, Michalko 1984, Feráková 1993), Synopses of the flora of the Czech Republic (Opiz 1852, Domin 1935); Flora of Hungary (Jávorka 1924), Flora of Bucovina (Herbich 1859), Conspects of the lora of Galicia (Zawadski 1835, Błocki 1883a, Błocki 1883b, Błocki 1883c, Błocki 1883d, Błocki 1883e, Błocki 1883f, Błocki 1883g, Błocki 1883h, Błocki 1883i, Błocki 1884a, Błocki 1884b, Błocki 1884c, Błocki 1884d, Błocki 1884e, Błocki 1884f, Zapałowicz 1906b), Synopses of the Central European flora (Ascherson and Graebner 1896, Soó 1972) and Conspects of the Flora of Transylvania (Baumgarten 1816b, Fuss 1866, Schur 1866, Simonkai 1886). Such checklists as Czerepanov (1995), Tasenkevich (1998), Mosyakin and Fedoronchuk (2015), Mirek et al. (2020) and other specific publications cited directly in the text were also applied. The Biodiversity Heritage Library (BHL 2022), Biblioteca del Real Jardín Botánico (RJB-CSIC 2023), Repository of the Library and Information Centre of the Hungarian Academy of Sciences (REAL-J 2023), Google Books (Google 2023), Internet Archive (2022), Hungaricana (Országgyűlési Könyvtár 2023), E-Periodica (ETH Library 2023), Biblioteca Digitala BCU Cluj (Lucian Blaga Central University Library 2022), Elektronikus Periodika Archívum (Hungarian Electronic Library 2023), Digitální knihovna AV ČR (Akademie věd České republiky 2023), Wielkopolska Biblioteka Cyfrowa (WBC 2023) and Guide to the plant species descriptions published in seed lists from Botanic Gardens for the period 1800−1900 (Lut and Veldkamp 2023) have been used as a source of old printed materials containing the initial protologues of taxa.

Besides this, such databases as the International Plant Name Index (IPNI 2023), Catalogue of Life (Bánki et al. 2023), Euro+Med PlantBase (Euro+Med 2023), Plants of the World Online (POWO 2023), Worldplants (Hassler 2023), Global Biodiversity Information Facility (GBIF 2023), Wikispecies (Wikimedia 2023), World Flora Online (WFO 2023), Florenliste von Deutschland (Gefäßpflanzen) (Hand et al. 2023) and Global Compositae Database (Compositae Working Group 2023) were also intensively used to construct the initial taxonomic backbone of the present checklist and to verify listed names.

The repositories JACQ (JACQ consortium 2023) and Global Plants (JSTOR 2023) were used to reveal existing type material for all analysed taxa, including those listed as synonyms. The TreatmentBank (Plazi 2023) was used to locate digitised taxonomic treatments for all valid taxa.

Finally, over 7,000 vouchers deposited in the leading Ukrainian herbaria (i.e. KW, KWHA, KWU, CHER, UU, LW, LWS and LWKS – see Thiers (2022) for abbreviations) were processed. Additional information gathered from KRA and KRAM Herbaria and Domin’s Card Index deposited at the Institute of Botany of the SAS in Bratislava was also integrated into the checklist. The dataset of processed specimens has been published in GBIF (Novikov and Sup-Novikova 2022a, Novikov and Sup-Novikova 2022b).

Below is the extended list of the analysed endemic species and subspecies of vascular plants distributed in the Ukrainian Carpathians, with some critical remarks and full nomenclature citations. The brief alphabetic list compiled for quick navigation and routine work with herbarium material is provided in Suppl. material 1. The hierarchically organised checklist with collapsed homotypic synonyms for easier nomenclatural work is provided in Suppl. material 2. The links and accession numbers for DNA sequences for all species revealed using the European Nucleotide Archive (EMBL-EBI 2023) facilities and cross-checked with Barcode of Life Data Systems (Ratnasingham and Hebert 2007, BOLD 2023) are provided in Suppl. material 3. The draft table version of this checklist (with some working Cyrillic annotations) used to create the current paper is deposited and freely available from Zenodo (Novikov 2023).

## Checklists

### Endemic vascular plants distributed in the Ukrainian Carpathians

#### Liliopsida

E557369F-FBE9-52B6-BB1B-76CC173E4046

#### Asparagales

CEA044DB-BD31-5FC5-BE8A-89DECDDCB9CC

#### Asparagaceae

C896D0BF-B227-536C-A0F7-15255EC0752D

#### Scilla kladnii

Schur, Enum. Pl. Transsilv.: 668 (1866)

F3C03105-663D-56E3-AA04-4A0CB731BBFE

https://www.catalogueoflife.org/data/taxon/4VJH7

https://www.gbif.org/species/2767548

urn:lsid:ipni.org:names:540858-1

http://www.worldfloraonline.org/taxon/wfo-0000740952

https://powo.science.kew.org/taxon/540858-1

https://species.wikimedia.org/wiki/Scilla_kladnii

https://europlusmed.org/cdm_dataportal/taxon/43877abc-2ef9-4cbf-afaf-d18e92f3cf63

https://www.worldplants.de/world-plants-complete-list/complete-plant-list/?name=Scilla-bifolia

https://www.biodiversitylibrary.org/page/10544719#page/690/

≡ Scillabifoliavar.kladnii (Schur) Nyman, Consp. Fl. Eur.: 730 (1882); GBIF: https://www.gbif.org/species/2767528; IPNI: https://www.ipni.org/n/77291211-1; WFO: http://www.worldfloraonline.org/taxon/wfo-0000802907; POWO: https://powo.science.kew.org/taxon/77291211-1; BHL: https://www.biodiversitylibrary.org/page/11015874#page/741
= *Scillaalpina* Schur, Verh. Mitth. Siebenbürg. Vereins Naturwiss. Hermannstadt 3: 90 (1852); GBIF: https://www.gbif.org/species/2767537; IPNI: https://www.ipni.org/n/540643-1; WFO: http://www.worldfloraonline.org/taxon/wfo-0000734709; POWO: https://powo.science.kew.org/taxon/540643-1; BHL: https://www.biodiversitylibrary.org/page/11525300#page/961
= Scillabifoliasubsp.alpina (Schur) Nyman, Consp. Fl. Eur.: 730 (1882); GBIF: https://www.gbif.org/species/2767527; IPNI: https://www.ipni.org/n/77258410-1; WFO: http://www.worldfloraonline.org/taxon/wfo-0000772133; POWO: https://powo.science.kew.org/taxon/77258410-1; BHL: https://www.biodiversitylibrary.org/page/11015874#page/741
= Scillabifoliavar.alpina (Schur) C.Zahariadi in Nyár., Fl. Rep. Pop. Rom. 11: 314 (1966)
= *Scillabifolia* β [unranked] *gracillima* Grecescu, Consp. Fl. Rom.: 565 (1898), non alior
= Scillabifoliasubsp.subtriphylla (Schur) Domin, Preslia 13–15: 19 (1936); GBIF: https://www.gbif.org/species/6316610; IPNI: https://www.ipni.org/n/77258137-1; WFO: http://www.worldfloraonline.org/taxon/wfo-0000915343; POWO: https://powo.science.kew.org/taxon/77258137-1
= Scillabifoliavar.subtriphylla (Schur) T.Simon, Ann. Biol. Univ. Debrecen. n.s., 1: 154 (1950); GBIF: https://www.gbif.org/species/5952322; IPNI: https://www.ipni.org/n/77291428-1; WFO: http://www.worldfloraonline.org/taxon/wfo-0000915344; POWO: https://powo.science.kew.org/taxon/77291428-1
= *Scillasubtriphylla* Schur, Enum. Pl. Transsilv.: 668 (1866) *; GBIF: https://www.gbif.org/species/2767536; IPNI: https://www.ipni.org/n/541057-1; WFO: http://www.worldfloraonline.org/taxon/wfo-0000741455; POWO: https://powo.science.kew.org/taxon/541057-1; BHL: https://www.biodiversitylibrary.org/page/10544719#page/690
= *Scillatrifolia* Schur, Enum. Pl. Transsilv.: 668 (1866); GBIF: https://www.gbif.org/species/2767525; IPNI: https://www.ipni.org/n/541069-1; WFO: http://www.worldfloraonline.org/taxon/wfo-0000741409; POWO: https://powo.science.kew.org/taxon/541069-1; BHL: https://www.biodiversitylibrary.org/page/10544719#page/690
– *Scillabifolia* L., Sp. Pl. 1: 309 (1753) [p. p., tantum quod plantas ucrain. carpat.], non alior *; GBIF: https://www.gbif.org/species/2767506; IPNI: https://www.ipni.org/n/540689-1; WFO: http://www.worldfloraonline.org/taxon/wfo-0000741049; POWO https://powo.science.kew.org/taxon/540689-1; BHL: https://www.biodiversitylibrary.org/page/358328#page/321
– Scillabifoliavar.nivalis auct. fl. carpat, non Baker
– Scillabifoliasubsp.nivalis (Boiss.) K.Richt., Pl. Eur., 1: 220 (1890) sensu Fodor [non sensu orig.]
– *Scillapraecox* auct. fl. carpat, non Willd.

##### Conservation status

In Ukraine – LC (Onyshchenko et al. 2022).

##### Distribution

Pancarpathian subendemic.

##### Notes

It was shown that *Scillabifolia* L. is represented in the Ukrainian Carpathians by single subspecies – subsp. subtriphylla (Schur) Domin, which is a synonym to *S.kladnii* (Kricsfalusy and Vajnagi 1994, Kolesnyk 2001, Kolesnyk 2003).

#### Iridaceae

FAEECC43-0D55-512B-9C04-66E998340190

#### Crocus banaticus

J.Gay, Bull. Sci. Nat. Géol. (Bull. Férussac), 25: 220, Nr. 178 (1831), non Heuff.

653BE5A0-BD5A-5F50-9852-975230219D4C

https://www.catalogueoflife.org/data/taxon/ZKSS

https://www.gbif.org/species/2747589

urn:lsid:ipni.org:names:436477-1

http://www.worldfloraonline.org/taxon/wfo-0000788544

https://powo.science.kew.org/taxon/436477-1

https://species.wikimedia.org/wiki/Crocus_banaticus

https://europlusmed.org/cdm_dataportal/taxon/d631ff50-1df9-4cff-8d91-1dacd8c44ed2

https://www.worldplants.de/world-plants-complete-list/complete-plant-list/?name=Crocus-banaticus

https://www.biodiversitylibrary.org/item/25919#page/718

https://hal.jacq.org/HAL0134213

https://plants.jstor.org/stable/10.5555/al.ap.specimen.k000499181

https://plants.jstor.org/stable/10.5555/al.ap.specimen.k000499180

https://plants.jstor.org/stable/10.5555/al.ap.specimen.k000499179

https://plants.jstor.org/stable/10.5555/al.ap.specimen.k000499183

https://plants.jstor.org/stable/10.5555/al.ap.specimen.k000499182

https://plants.jstor.org/stable/10.5555/al.ap.specimen.mo-202890

= *Crocusherbertianus* Körn., Index Seminum (B, Berolinensis) 1854 (App.): 15 (1855); GBIF: https://www.gbif.org/species/2747686; IPNI: https://www.ipni.org/n/436554-1; WFO: http://www.worldfloraonline.org/taxon/wfo-0000788677; POWO: https://powo.science.kew.org/taxon/436554-1
= *Crocusiridiflorus* Heuff. ex Rchb., Ic. Fl. Germ. 9: 10, figs. 802, 803 (1847); GBIF: https://www.gbif.org/species/8465903; GBIF: https://www.gbif.org/species/2747687; IPNI: https://www.ipni.org/n/436571-1; WFO: http://www.worldfloraonline.org/taxon/wfo-0000788697; POWO: https://powo.science.kew.org/taxon/436571-1; BHL: https://www.biodiversitylibrary.org/item/28666#page/20; JSTOR Global Plants: https://plants.jstor.org/stable/10.5555/al.ap.specimen.k000499176; JSTOR Global Plants: https://plants.jstor.org/stable/10.5555/al.ap.specimen.k000499177
= *Crocusnudiflorus* Schult., Oestr. Fl., ed. 2, 1: 101 (1814) [nom. illeg.], non alior; GBIF: https://www.gbif.org/species/7380666; IPNI: https://www.ipni.org/n/436632-1; WFO: http://www.worldfloraonline.org/taxon/wfo-0000788771; POWO: https://powo.science.kew.org/taxon/436632-1
= *Crocusspeciosus* Baumg., Enum. Stirp. Transsilv. 1: 60 (1816), non alior
= *Crocusspeciosus* (Baumg.) Host, Fl. Austr. 1: 43 (1827), non alior [nom. illeg.]
= Crocusspeciosusvar.transsylvanicus Hooker, Curt. Bot. Mag. 67: t. 3861 (1840–1841); GBIF: https://www.gbif.org/species/2747724; IPNI: https://www.ipni.org/n/77271176-1; WFO: http://www.worldfloraonline.org/taxon/wfo-0000803365; POWO: https://powo.science.kew.org/taxon/77271176-1; BHL: https://www.biodiversitylibrary.org/item/14345#page/205
= *Crocirisiridiflora* (Heuff. ex Rchb.) Schur, Verh. Mitth. Siebenbürg. Vereins Naturwiss. Hermannstadt 4: 73 (1853); GBIF: https://www.gbif.org/species/2747450; IPNI: https://www.ipni.org/n/436423-1; WFO: http://www.worldfloraonline.org/taxon/wfo-0000782650; POWO: https://powo.science.kew.org/taxon/436423-1; BHL: https://www.biodiversitylibrary.org/page/11525300#page/959
= *Crocirisspeciosa* (Host) Schur, Verh. Mitth. Siebenbürg. Vereins Naturwiss. Hermannstadt 4: 73 (1853); GBIF: https://www.gbif.org/species/2747453; IPNI: https://www.ipni.org/n/436424-1; WFO: http://www.worldfloraonline.org/taxon/wfo-0000782651; POWO: https://powo.science.kew.org/taxon/436424-1; BHL: https://www.biodiversitylibrary.org/page/11525300#page/959
– *Crocusbyzantinus* Ker Gawl., Bot. Mag. 28: t. 1111 (1808) [p. p.]; GBIF: https://www.gbif.org/species/2747452; GBIF: https://www.gbif.org/species/11347665; IPNI: https://www.ipni.org/n/436488-1; WFO: http://www.worldfloraonline.org/taxon/wfo-0000788579; POWO: https://powo.science.kew.org/taxon/436488-1; BHL: https://www.biodiversitylibrary.org/item/14316#page/152

##### Conservation status

In Ukraine – NT (Onyshchenko et al. 2022).

##### Distribution

SE Carpathian subendemic.

##### Notes

In Ukraine, this species occurs in Transcarpathia only and is listed in the Red Book of Ukraine as vulnerable (Mihály and Komendar 1993, Mygal 2009, MEPNR of Ukraine 2021). In his paper, Hooker (1840), in table 3861, indicated that C.speciosusvar.transsylvanicus is a synonym of *C.speciosus* mentioned by Lindley (1839) in table 40. However, Lindley clearly stated that he considered *C.speciosus* sensu Baumg. (not sensu M.Bieb.) and also mentioned these plants under the name *C.nudiflorus*. Both taxa, *C.speciosus* sensu Baumg. and *C.nudiflorus* are known synonyms of *C . banaticus* . Consequently, C.speciosusvar.transsylvanicus is also considered here as a synonym of *C.banaticus*. Danciu and Golban (2009) on page 131 also mentioned *C.speciosus* Rochel, non alior as a synonym of *C.banaticus.* However, in the original work of Rochel (1828), there is no evidence of its synonymy with *C . banaticus*.

GBIF (https://www.gbif.org/species/2747449, accessed on 05.06.2023), POWO (https://powo.science.kew.org/taxon/436685-1, accessed on 05.06.2023), WFO (https://list.worldfloraonline.org/wfo-0000788843, accessed on 05.06.2023) and Euro+Med (https://europlusmed.org/cdm_dataportal/taxon/719bcaf0-a50e-4a66-86b9-fc4b2a73352f, accessed on 05.06.2023) mistakenly indicate *Crocirisiridiflora* Schur and *C.speciosa* Schur as synonyms for *Crocussalzmannii* J.Gay occurring in Spain, Morocco and Algeria. Both Schur’s species were described from the Carpathian region (Schur 1853) and have no relation to *C.salzmannii*, which is sometimes recognised as a subspecies, C.serotinussubsp.salzmannii (J.Gay) B.Mathew). It was shown that *C.banaticus*, which, for a long time, was considered belonging to the independent subgenus CrocirisSchur, is a morphological variant withinsectionCrocus, but still distinct from *C.salzmannii* (Surányi et al. 2010, Harpke et al. 2013). This mistaken synonymisation has been resolved in the Worldplants database (https://www.worldplants.de/world-plants-complete-list/complete-plant-list?name=Crocus-salzmannii, accessed on 07.06.2023).

#### Orchidaceae

5AA0C612-D9BE-507D-ADFA-69D2A44E8AA8

#### Gymnadenia carpatica

(Zapał.) Teppner et E.Klein, Phyton (Horn) 38 (1): 221 (1998)

82EC76E3-5B88-5281-ACF4-2A8BB1247F5D

https://www.catalogueoflife.org/data/taxon/3HP3F

https://www.gbif.org/species/2840452

urn:lsid:ipni.org:names:1003516-1

http://www.worldfloraonline.org/taxon/wfo-0000976550

https://powo.science.kew.org/taxon/1003516-1

https://species.wikimedia.org/wiki/Gymnadenia_carpatica

https://europlusmed.org/cdm_dataportal/taxon/d0bda98f-b0f8-4192-a6fa-e8ce84a65cf6

https://www.worldplants.de/world-plants-complete-list/complete-plant-list/?name=Nigritella-nigra

≡ *Nigritellacarpatica* (Zapał.) Teppner, E.Klein & Zag., Phyton (Horn) 34: 171 (1994) *; GBIF: https://www.gbif.org/species/5323915; IPNI: https://www.ipni.org/n/981684-1; WFO: http://www.worldfloraonline.org/taxon/wfo-0000976550; POWO: https://powo.science.kew.org/taxon/981684-1
≡ Nigritellaangustifoliavar.carpatica Zapał., Consp. Fl. Gallic. Crit. 1: 215 (1906); GBIF: https://www.gbif.org/species/5323917; IPNI: https://www.ipni.org/n/50989850-1; WFO: http://www.worldfloraonline.org/taxon/wfo-0000251887; POWO: https://powo.science.kew.org/taxon/50989850-1
≡ Nigritellanigrasubsp.carpatica (Zapał.) H.Baumann & R.Lorenz, J. Eur. Orch. 37: 717 (2005); GBIF: https://www.gbif.org/species/8213444; IPNI: https://www.ipni.org/n/77070561-1; WFO: http://www.worldfloraonline.org/taxon/wfo-0000806174; POWO: https://powo.science.kew.org/taxon/77070561-1
≡ Nigritellanigravar.carpatica (Zapał.) Pawł., Bull. Int. Acad. Polon. Sci., Cl. Sci. Math., Sér. B 1, Bot. 1947: 85, 96 (1947); GBIF: https://www.gbif.org/species/5323916; WFO: http://www.worldfloraonline.org/taxon/wfo-0000839712; POWO: https://powo.science.kew.org/taxon/377060-4
≡ Nigritellarubraf.carpatica (Zapał.) Soó, Repert. Sp. Nov. Regni Veg. 24: 33 (1927); GBIF: https://www.gbif.org/species/5950762; POWO: https://powo.science.kew.org/taxon/376651-4
– *Gymnadenianigra* auct. fl. ucrain. carpat., non (L.) Rchb.f.
– *Nigritellanigra* auct. fl. ucrain. carpat., non (L.) Rchb.f. *

##### Conservation status

In Ukraine – EN (Onyshchenko et al. 2022).

##### Distribution

SE Carpathian endemic

##### Notes

The Red Book of Ukraine lists this species as obsolescent (Chorney 2009c, MEPNR of Ukraine 2021).

Nigritellarubraf.carpatica (Zapał.) Soó is a homonym of *Gymnadeniacarpatica.* However, GBIF (https://www.gbif.org/species/2840596, accessed on 05.06.2023), POWO (https://powo.science.kew.org/taxon/636560-1, accessed on 05.06.2023) and WFO (https://list.worldfloraonline.org/wfo-0000976634, accessed on 07.06.2023) mistakenly provide Nigritellarubraf.carpatica amongst synonyms to *G.miniata* (Crantz) Hayek. *Gymnadeniaminiata* (= *Gymnadeniarubra* Wettst.) as a distinct West-South-Central European red-flowered species of vanilla orchid (Baumann and Lorenz 2011) that does not occur in the Carpathians (https://powo.science.kew.org/taxon/636560-1, accessed on 07.07.2023).

#### Poales

805A29BB-76F6-5B7E-A982-2FAFA21316BF

#### Juncaceae

ADA3AD45-FA37-5502-8342-A752DD9392B9

#### Luzula alpinopilosa obscura

S.E.Fröhner, Preslia 40: 426 (1968)

E3838538-5E75-5882-B85E-0C3599F6F920

https://www.catalogueoflife.org/data/taxon/5JFTM

https://www.gbif.org/species/7870156

https://www.gbif.org/species/8067039

http://www.worldfloraonline.org/taxon/wfo-0000777604

https://powo.science.kew.org/taxon/50966612-1

https://europlusmed.org/cdm_dataportal/taxon/0a2c93e4-408c-4767-84b9-214de6f3ce86

https://www.worldplants.de/world-plants-complete-list/complete-plant-list/?name=Luzula-alpinopilosa

≡ *Luzulaobscura* (S.E.Fröhner) Novikov, Byull. Moskovsk. Obshch. Isp. Prir., Otd. Biol. 95(6): 66 (1990); GBIF: https://www.gbif.org/species/4204323; IPNI: https://www.ipni.org/n/962193-1; WFO: http://www.worldfloraonline.org/taxon/wfo-0000778005; POWO: https://powo.science.kew.org/taxon/962193-1
= *Luzulacarpatica* Kitt. ex Kanitz, Linnaea 32: 327 (1863); GBIF: https://www.gbif.org/species/9058223; GBIF: https://www.gbif.org/species/7559197; IPNI: https://www.ipni.org/n/443753-1; WFO: http://www.worldfloraonline.org/taxon/wfo-0000777735; POWO: https://powo.science.kew.org/taxon/77242868-1; BHL: https://www.biodiversitylibrary.org/page/118417#page/330
= Luzulaspadiceavar.carpatica (Kitt. ex Kanitz) Nyman, Consp. Fl. Eur., Suppl. 2: 314 (1890); GBIF: https://www.gbif.org/species/7629382; IPNI: https://www.ipni.org/n/77282694-1; WFO: http://www.worldfloraonline.org/taxon/wfo-0000803768; POWO: https://powo.science.kew.org/taxon/77282694-1
= *Luzulaspadicea* [unranked] *carpatica* (Kitt. ex Kanitz) Asch. & Graebn., Syn. Mitteleur. Fl. 2(2): 513 (1904); GBIF: https://www.gbif.org/species/9651841; BHL: https://www.biodiversitylibrary.org/page/25150622#page/523
= Luzulaspadiceaf.carpatica (Kitt. ex Kanitz) I.Grinț., Fl. Rep. Soc. Rom. 11: 594 (1966); GBIF: https://www.gbif.org/species/7942645; WFO: http://www.worldfloraonline.org/taxon/wfo-0000778119; POWO: https://powo.science.kew.org/taxon/316684-4
= *Juncusspadiceus* [unranked] β *glabratus* Wahlenb., Fl. Carp. Princip.: 102 (1814), non Hoppe nec Host
– *Juncusalpinopilosus* Chaix, Hist. Pl. Dauphiné (Villars) 1: 318 (1786) [p. p., tantum quod plantas carpat.]; GBIF: https://www.gbif.org/species/2700949; IPNI: https://www.ipni.org/n/442659-1; WFO: http://www.worldfloraonline.org/taxon/wfo-0000775785; POWO: https://powo.science.kew.org/taxon/442659-1; BHL: https://www.biodiversitylibrary.org/page/50324360#page/416
– *Juncusspadiceus* All., Fl. Pedem. 2: 216 (1785) [nom. invalid., p. p., tantum quod plantas carpat.], non alior; GBIF: https://www.gbif.org/species/7420868; IPNI: https://www.ipni.org/n/443494-1; WFO: http://www.worldfloraonline.org/taxon/wfo-0000777249; POWO: https://powo.science.kew.org/taxon/443494-1
– *Luzulaalpinopilosa* (Chaix) Breistr., Bull. Soc. Sci. Dauph. 61: 609 (1947) [p. p., tantum quod plantas carpat.] *; GBIF: https://www.gbif.org/species/2700948; IPNI: https://www.ipni.org/n/443701-1; WFO: http://www.worldfloraonline.org/taxon/wfo-0000777600; POWO: https://powo.science.kew.org/taxon/443701-1
– *Luzulaspadicea* (All.) Lam. & DC., Fl. Franc. [de Candolle & Lamarck], ed. 3. 3: 159 (1805) [p. p., tantum quod plantas carpat.] *; GBIF: https://www.gbif.org/species/9449570; IPNI: https://www.ipni.org/n/443937-1; WFO: http://www.worldfloraonline.org/taxon/wfo-0000778114; POWO: https://powo.science.kew.org/taxon/443937-1; BHL: https://www.biodiversitylibrary.org/page/49712877#page/169

##### Conservation status

In Ukraine – LC (Onyshchenko et al. 2022).

##### Distribution

Pancarpathian endemic.

##### Notes

Following POWO (https://powo.science.kew.org/taxon/443701-1, accessed on 05.06.2023), there are three subspecies of L.alpinopilosa(Chaix)Breistr –subsp.alpinopilosa(distributed in Western and Central Europe),subsp.deflexa(Kožuharov) Kirschner (native to South Europe) andsubsp.obscura (the only subspecies occurring in the Carpathians). Chopyk and Fedoronchuk (2015), on page 544, considered *L.spadicea* (All.) DC. a synonym of *L.alpinopilosa subsp. obscura* and *L.alpinopilosa*. Similarly, Mirek et al. (2020), on page 112, considered *L.spadicea* a synonym of *L.alpinopilosa* without clarification of the subspecies. Considering the absence of other subspecies in the range, all plants from the Carpathian Mts. identified as *L.alpinopilosa* and *L.spadicea* should be regarded as belonging to L.alpinopilosasubsp.obscura.

#### Poaceae

72263A8C-7B93-5798-B15D-F80AEB67A46F

#### Alopecurus pratensis laguriformis

(Schur) Tzvelev, Novosti Sist. Vyssh. Rast. 8: 19 (1971)

3037CFF0-D2C8-5276-9537-055116CBC277

https://www.catalogueoflife.org/data/taxon/5FJ2M

https://www.gbif.org/species/5672026

urn:lsid:ipni.org:names:881439-1

http://www.worldfloraonline.org/taxon/wfo-0000845668

https://powo.science.kew.org/taxon/881439-1

https://europlusmed.org/cdm_dataportal/taxon/9c3dfa16-2a37-49e2-b6c0-7e576959f9d3

https://www.worldplants.de/world-plants-complete-list/complete-plant-list/?name=Alopecurus-pratensis

https://w.jacq.org/W0025395

https://w.jacq.org/W19160035870

https://plants.jstor.org/stable/10.5555/al.ap.specimen.k000913477

https://plants.jstor.org/stable/10.5555/al.ap.specimen.k000913476

≡ *Alopecuruslaguriformis* Schur, Verh. Siebenb. Ver. Naturw. 1: (1850) 182 [nom. nudum] et Schur ex Gris., Iter. Hung: 362 (1852) *; GBIF: https://www.gbif.org/species/5672208; IPNI: https://www.ipni.org/n/387117-1; WFO: http://www.worldfloraonline.org/taxon/wfo-0000845668; POWO: https://powo.science.kew.org/taxon/387117-1; BHL: https://www.biodiversitylibrary.org/page/11525300#page/190; JSTOR Global Plants: https://plants.jstor.org/stable/10.5555/al.ap.specimen.w0025395
= *Alopecuruslaguriformis* [unranked] a abbreviatus Schur, Oesterr. Bot. Z. 9: 13 (1859); BHL: https://www.biodiversitylibrary.org/item/36437#page/19
= *Alopecuruslaguriformis* [unranked] b *elongatus* Schur, Oesterr. Bot. Z. 9: 13 (1859) et Enum. Pl. Transsilv.: 727 (1866); GBIF: https://www.gbif.org/species/5943426; IPNI: https://www.ipni.org/n/77263212-1; WFO: http://www.worldfloraonline.org/taxon/wfo-0000845589; POWO: https://powo.science.kew.org/taxon/77263212-1; BHL: https://www.biodiversitylibrary.org/item/36437#page/19
= *Alopecurustranssilvanicus* Schur, Enum. Pl. Transsilv.: 727 (1866); GBIF: https://www.gbif.org/species/5671949; IPNI: https://www.ipni.org/n/387210-1; WFO: http://www.worldfloraonline.org/taxon/wfo-0000845481; POWO: https://powo.science.kew.org/taxon/387210-1; BHL: https://www.biodiversitylibrary.org/page/10544778#page/749
– *Alopecurusbrachystachyus* auct. [e.g., Janka], non M.Bieb.
– *Colobachnegerardi* Schur, Enum. Pl. Transsilv.: 728 (1866), non Link.; BHL: https://www.biodiversitylibrary.org/page/10544778#page/750

##### Conservation status

In Ukraine – DD (Onyshchenko et al. 2022).

##### Distribution

SE Carpathian endemic.

##### Notes

Three (https://powo.science.kew.org/taxon/387176-1, accessed on 05.06.2023) to five (https://europlusmed.org/cdm_dataportal/taxon/1d82b70c-8685-4ea4-a6d2-296864770429, accessed on 05.06.2023) subspecies of A.pratensisL. are recognised viz.subsp.pratensis(cosmopolite),subsp.alpestris(Wahlenb.) Selander (occurs in Northern Eurasia),subsp.laguriformis(occurs in Romania and, probably, in Ukraine),subsp.songaricus(Fisch. & C.A.Mey.) N.V.Vlassova (questionable taxon declared for Asia) andsubsp.pseudonigricans O.Schwarz. (dubious taxon mentioned for Germany and Czech republic). WFO (https://list.worldfloraonline.org/wfo-0000845656, accessed on 05.06.2023) also recognises A.pratensisvar.aquaticus (Dumort.) Mathieu (≡ *A.aquaticus* Dumort.), which is, however, synonymised by the rest of the databases with the widely distributed *A.arundinaceus* Poir. Presence of A.pratensissubsp.laguriformis in the Ukrainian Carpathians is doubtful and requires confirmation (Chopyk and Fedoronchuk 2015).

GBIF (https://www.gbif.org/species/5672406, accessed on 05.06.2023) POWO (https://powo.science.kew.org/taxon/387039-1, accessed on 05.06.2023), WFO (https://list.worldfloraonline.org/wfo-0000845481, accessed on 05.06.2023) and Worldplants (https://www.worldplants.de/world-plants-complete-list/complete-plant-list?name=Alopecurus-brachystachyus, accessed on 07.06.2023), mistakenly indicate A.laguriformisvar.elongatus Schur and *A.transsilvanicus* Schur as synonyms of *A.brachystachyus* M.Bieb. Schur (1866), on page 727, indeed synonymised *A.transsilvanicus* with A.laguriformisvar.elongatus distributed in Făgăraș (Schur 1859) and Rodna Mts. (Schur 1866). Schur (1866), on pages 727 and 728, also pointed out that Neilreich (1861) believed that *A.transsilvanicus*, *A.colobachnoides* Trin. and *A.vlassovii* Trin are synonyms of *A.brachystachyus* distributed in the Carpathians (Făgăraș, Rodna and Kronstadt [= Brașov]). However, Neilreich’s taxonomic interpretation of the mentioned taxa was mistaken because he relied on Janka (1858), who concluded that revised specimens of *A.laguriformis* are identical to the plants of *A.vlassovii* from the Altai and Baikal. In fact, this Janka’s conclusion led to the synonymisation of *A.laguriformis* with *A.vlassovii* and, consequently, with *A.colobachnoides*, which are morphologically different and geographically isolated taxa *Tzvelev (1976).* Such Janka’s misinterpretation of *A.laguriformis* was first outlined by Simonkai (1886). Later, Tzvelev (1971), in his revision of the genus *Alopecurus* distinguished *A.brachystachyus* and *A.laguriformis* and downgraded the last one to the rank of subspecies *A.pratensissubsp.laguriformis* Tzvelev (1974), Tzvelev (1976), Tzvelev (1978) and Tzvelev (1978) also pointed out that A.pratensissubsp.laguriformis is known exclusively from the Carpathians, while *A.brachystachyus* (≡ *A.colobachnoides* and = *A.vlassovii*) occurs out of Europe far to the east, in the Caucasus, Siberia, China and Mongolia.

#### Festuca amethystina orientalis

Krajina, Acta Bot. Bohem. 9: 214 (1930), non alior

7699EBFA-189B-553E-8C93-3B8DC21EACA0

https://www.catalogueoflife.org/data/taxon/5HC6W

https://www.gbif.org/species/5940986

urn:lsid:ipni.org:names:77188270-1

http://www.worldfloraonline.org/taxon/wfo-0000869748

https://powo.science.kew.org/taxon/77188270-1

https://europlusmed.org/cdm_dataportal/taxon/aa72de8f-989c-48f8-b841-662e5460634b

https://www.worldplants.de/world-plants-complete-list/complete-plant-list/?name=Festuca-amethystina

= Festucaamethystinasubsp.inarmata (Schur) Krajina, Veröff. Geobot. Inst. Rübel Zürich 10: 29 (1933); GBIF: https://www.gbif.org/species/7651353; WFO: https://list.worldfloraonline.org/wfo-0001097471
= Festucaamethystinasubsp.amethystinavar.amethystinaf.marmarossica (Zapał.) Beldie, Fl. Rep. Pop. Soc. Rom. 12: 557 (1972)
= Festucaamethystinasubsp.amethystinavar.amethystinaf.pauciflora (A.Nyár. & Nyár.) Beldie, Fl. Rep. Soc. Rom. 12: 557 (1972)
= *Festucaamethystina* [unranked] a marmarossica Zapał., Consp. Fl. Galic. Crit. 1: 65 (1906) [ortho. var.] *
= *Festucaamethystina* [unranked] a marmarossiensis Zapał., Consp. Fl. Galic. Crit. 3: 230 (1911)
= *Festucaamethystina* [unranked] a marmarossiensisf.doamnensis Zapał., Consp. Fl. Galic. Crit. 3: 230 (1911)
= Festucaamethystinaf.pauciflora A.Nyár. & Nyár., Studii Cercet. Biol. Ser. Bot. 16(2): 109 (1964)
= Festucaheterophyllavar.inarmata Schur ex Schur, Enum. Pl. Transsilv.: 792 (1866); BHL: https://www.biodiversitylibrary.org/item/7364#page/812
= Festucaheterophyllavar.setifolia Schur ex Schur, Enum. Pl. Transsilv.: 792 (1866); BHL: https://www.biodiversitylibrary.org/item/7364#page/812
= *Festucainarmata* Schur, Verh. Mitth. Siebenbürg. Vereins Naturwiss. Hermannstadt 10: 177 (1859) *; GBIF: https://www.gbif.org/species/4122548; IPNI: https://www.ipni.org/n/402888-1; WFO: http://www.worldfloraonline.org/taxon/wfo-0000870754; POWO: https://powo.science.kew.org/taxon/402888-1; BHL: https://www.biodiversitylibrary.org/page/11528081#page/421
– *Festucaamethystina* L., Sp. Pl. 1: 74 (1753) [p. p., tantum quod plantas ucrain. carpat.] *; GBIF: https://www.gbif.org/species/4113725; IPNI: https://www.ipni.org/n/402336-1; WFO: http://www.worldfloraonline.org/taxon/wfo-0000869744; POWO: https://powo.science.kew.org/taxon/402336-1; BHL: https://www.biodiversitylibrary.org/page/358093#page/86

##### Conservation status

In Ukraine – LC (Onyshchenko et al. 2022).

##### Distribution

SE Carpathian endemic.

##### Notes

Two (https://powo.science.kew.org/taxon/402336-1, accessed on 05.06.2023) to four (https://europlusmed.org/cdm_dataportal/taxon/4427a965-d232-4346-97dd-5275c9dbdb31, accessed on 05.06.2023) subspecies of FestucaamethystinaL. are recognised –subsp.amethystina(distributed almost in the whole of Europe),subsp.orientalis(=subsp.inarmata, endemic to the south-eastern Carpathians; its presence in the Balkans doubted by Kliment et al. (2016)), subsp. kummeri (Beck) Markgr.-Dann. (sporadically represented in South and Central Europe) and subsp. ritschlii (Hack.) Markgr.-Dann. (occurs in Germany, Czech Republic, Slovakia, Poland and Romania – see Jakubowska-Gabara (1994), Indreica (2007), Kiedrzyński et al. (2015), Łazarski (2016), Rewicz et al. (2018)). Euro+Med, based on personal communication with B. Valdés in 2004, but without any further confirmation, also declares the presence of F.amethystinasubsp.ritschlii (https://europlusmed.org/cdm_dataportal/taxon/d2e8901d-25eb-420d-8c69-3b4b93a622d9, accessed on 05.06.2023), as well as subsp. orientalis (https://europlusmed.org/cdm_dataportal/taxon/aa72de8f-989c-48f8-b841-662e5460634b, accessed on 05.06.2023) in Ukraine. Roleček et al. (2019) also suggest the presence of F.amethystinasubsp.orientalis in the Ukrainian Carpathians instead of *F.amethystinasubsp.A.ethystina.* However, at the moment, there is no evidence confirming the presence of F.amethystinasubsp.ritschlii or F.amethystinasubsp.orientalis in the flora of the Ukrainian Carpathians. Only F.amethystinasubsp.orientalis is mentioned by Chopyk and Fedoronchuk (2015) and other Ukrainian authors (e.g. Bednarska (2007)). On the other hand, Rewicz et al. (2018) recently stressed the applicability of morphological traits for the delimitation of infraspecific taxa within *F.amethystina.* They pointed out the need for a taxonomic revision of this species to clarify its biogeography.

#### Festuca carpatica

F.Dietr., Nachtr. Vollst. Lex. Gärtn. 3: 333 (1817)

B37B4FE8-CCB6-555F-8BA1-A3C7A5BFCD95

https://www.catalogueoflife.org/data/taxon/6HRFT

https://www.gbif.org/species/4126785

urn:lsid:ipni.org:names:402534-1

http://www.worldfloraonline.org/taxon/wfo-0000870071

https://powo.science.kew.org/taxon/402534-1

https://species.wikimedia.org/wiki/Festuca_carpatica

https://europlusmed.org/cdm_dataportal/taxon/a38544a4-ae62-4ae4-8ce0-b9ad24647ffc

https://www.worldplants.de/world-plants-complete-list/complete-plant-list/?name=Festuca-carpatica

≡ *Amphigenescarpathica* (F.Dietr.) Janka, Linnaea 30(5): 619 (1859) & Janka ex Hack., Monogr. Fest. Eur.: 187 (1882); GBIF: https://www.gbif.org/species/7541275; GBIF: https://www.gbif.org/species/5671784; IPNI: https://www.ipni.org/n/387285-1; WFO: http://www.worldfloraonline.org/taxon/wfo-0000870071; POWO: https://powo.science.kew.org/taxon/387285-1; BHL: https://www.biodiversitylibrary.org/page/117103#page/622; BHL: https://www.biodiversitylibrary.org/item/52923#page/205
≡ *Leucopoacarpatica* (F.Dietr.) H.Scholz, Willdenowia 35: 242 (2005); GBIF: https://www.gbif.org/species/4136004; IPNI: https://www.ipni.org/n/77071608-1; WFO: http://www.worldfloraonline.org/taxon/wfo-0000870071; POWO: https://powo.science.kew.org/taxon/77071608-1
= *Amphigenesnutans* (Wahlenb.) Janka, Linnaea 30(5): 619 (1859); GBIF: https://www.gbif.org/species/5671783; IPNI: https://www.ipni.org/n/387286-1; WFO: http://www.worldfloraonline.org/taxon/wfo-0000870071; POWO: https://powo.science.kew.org/taxon/387286-1; BHL: https://www.biodiversitylibrary.org/page/117103#page/622
= Festucacarpaticavar.bucegica (Krajina) Beldie, Fl. şi veg. Bucegi: 330 (1967)
= Festucacarpaticavar.carpaticaf.subflavescens (Zapał.) Beldie, Fl. Rep. Soc. Rom. 12: 484 (1972)
= Festucacarpaticavar.carpaticaf.umbrosa Beldie, Fl. şi veg. Bucegi: 329 (1967)
= Festucacarpaticaf.elatior Krajina, Rozpr. Wydz. Mat.-Przyr. Akad. Umiejetn., Dzial B, Nauki Biol. 9: 219 (1930); GBIF: https://www.gbif.org/species/8335125; POWO: https://powo.science.kew.org/taxon/477638-4
= Festucacarpaticaf.pseudolaxa (Schur) Jáv., Magyar Bot. Lapok 10: 266 (1911); BHL: https://www.biodiversitylibrary.org/item/201840#page/298
= Festucacarpaticaf.subflavescens Zapał., Bull. Int. Acad. Sci. Cracovie, Cl. Sci. Math. 4B: 184. (1904); GBIF: https://www.gbif.org/species/7815522; POWO: https://powo.science.kew.org/taxon/477639-4
= *Festucadimorpha* Janka, Oesterr. Bot. Z. 16: 101 (1866), non Guss.; GBIF: https://www.gbif.org/species/8004377; IPNI: https://www.ipni.org/n/402651-1; WFO: http://www.worldfloraonline.org/taxon/wfo-0000870071; POWO: https://powo.science.kew.org/taxon/402651-1; BHL: https://www.biodiversitylibrary.org/page/28749984#page/109
= *Festucalaxa* Schur, Verh. Mitth. Siebenbürg. Vereins Naturwiss. Hermannstadt 10: 177 (1859), non Host; BHL: https://www.biodiversitylibrary.org/item/42663#page/421
= *Festucanutans* Wahlenb., Fl. Carpat. Princ.: 28 (1814), non Host nec Moench; IPNI: https://www.ipni.org/n/403175-1; GBIF: https://www.gbif.org/species/7994264
= *Festucapseudolaxa* Schur, Oesterr. Bot. Z. 8: 22 (1858); GBIF: https://www.gbif.org/species/4116805; IPNI: https://www.ipni.org/n/403338-1; WFO: http://www.worldfloraonline.org/taxon/wfo-0000871712; POWO: https://powo.science.kew.org/taxon/403338-1; BHL: https://www.biodiversitylibrary.org/page/28724891#page/30
= *Festucapseudonutans* Schur, Enum. Pl. Transsilv.: 796 (1866); GBIF: https://www.gbif.org/species/4116775; IPNI: https://www.ipni.org/n/403341-1; WFO: http://www.worldfloraonline.org/taxon/wfo-0000871740; POWO: https://powo.science.kew.org/taxon/403341-1; BHL: https://www.biodiversitylibrary.org/page/10544847#page/818
= Festucapulchellasubsp.scheuchzeriformisvar.bucegica Krajina, Veröff. Geobot. Inst. ETH Stiftung Rübel Zürich 10: 52 (1933)
= *Festucascheuchzeriformis* Schur, Enum. Pl. Transsilv.: 796 (1866); GBIF: https://www.gbif.org/species/4145715; IPNI: https://www.ipni.org/n/403491-1; WFO: http://www.worldfloraonline.org/taxon/wfo-0000871740; POWO: https://powo.science.kew.org/taxon/403491-1; BHL: https://www.biodiversitylibrary.org/page/10544847#page/818

##### Conservation status

In Ukraine – NT (Onyshchenko et al. 2022).

##### Distribution

Pancarpathian endemic.

##### Notes

GBIF (https://www.gbif.org/species/4116614, accessed on 05.06.2023), POWO (https://powo.science.kew.org/taxon/403363-1, accessed on 05.06.2023), Euro+Med (https://europlusmed.org/cdm_dataportal/taxon/e9bc861f-d097-45a6-bd73-706befdd5fc1, accessed on 05.06.2023) and Worldplants (https://www.worldplants.de/world-plants-complete-list/complete-plant-list?name=Leucopoa-pulchella, accessed on 07.06.2023) mistakenly consider *F.pseudonutans* Schur and *F.scheuchzeriformis* Schur as synonyms of *F.pulchella* Schrad. (≡ *Leucopoapulchella* (Schrad.) H.Scholz & Foggi). Schur (1866), who described *F.scheuchzeriformis* and *F.pseudonutans*, later indicated them to be synonymic with *F.carpatica* and joined under the name *F.scheuchzeriformis.* Schur (1866) noted that this species occurs in the outskirts of Kronstadt (= Brașov). Merging of *F.scheuchzeriformis* with *F.pulchella*, perhaps, resulted from recombinations made by Krajina (1933) on page 51, who downgraded *F.scheuchzeriformis* to the rank of subspecies (i.e. F.pulchellasubsp.scheuchzeriformis(Schur) Krajina) and delimited two varieties within this subspecies –var.bucegicaKrajina (Muntii Bucegi, Transsylvania) andvar.plicata (Huter. in Hackel) Krajina (south Austria and Yura, Switzerland). At the same time, Krajina (1933), on page 52, described the second subspecies within *F.pulchella – F.pulchella* subsp. eu-pulchella Krajina, distributed in Italy, Austria, Slovenia, Croatia and Germany. Hence, following Krajina (1933), only F.pulchellasubsp.scheuchzeriformisvar.bucegica occurs in the Carpathians. Therefore, only this variety corresponds to *F.scheuchzeriformis* in the sense of Schur (1866). Consequently, this variety corresponds to *F.carpatica*, endemic to the Carpathians (Kliment et al. 2016).

#### Festuca porcii

Hack., Bot. Centralbl. 2(8): 407 (1881)

00CBCE86-D379-57B3-9029-3576C055FB87

https://www.catalogueoflife.org/data/taxon/6HSGL

https://www.gbif.org/species/4117145

urn:lsid:ipni.org:names:403318-1

http://www.worldfloraonline.org/taxon/wfo-0000871666

https://powo.science.kew.org/taxon/403318-1

https://species.wikimedia.org/wiki/Festuca_porcii

https://europlusmed.org/cdm_dataportal/taxon/a01bf487-a40c-48e8-aa7b-b8f6eb79215a

https://www.worldplants.de/world-plants-complete-list/complete-plant-list/?name=Festuca-porcii

https://www.biodiversitylibrary.org/page/3095011#page/427

https://kfta.jacq.org/KFTA0002813

https://dr.jacq.org/DR052737

https://w.jacq.org/W19160014841

https://w.jacq.org/W19160014842

https://w.jacq.org/W19160014843

https://je.jacq.org/JE00007301

https://plants.jstor.org/stable/10.5555/al.ap.specimen.w19160014841

https://plants.jstor.org/stable/10.5555/al.ap.specimen.je00007301

https://plants.jstor.org/stable/10.5555/al.ap.specimen.w19160014843

https://plants.jstor.org/stable/10.5555/al.ap.specimen.kfta0002813

https://plants.jstor.org/stable/10.5555/al.ap.specimen.w19160014842

https://plants.jstor.org/stable/10.5555/al.ap.specimen.cas0027412

https://plants.jstor.org/stable/10.5555/al.ap.specimen.mpu027818

= Festucaporciif.hirsuta (A.Nyár.) Beldie, Fl. Rep. Soc. Rom. 12: 524 (1972); GBIF: https://www.gbif.org/species/4117144; IPNI: https://www.ipni.org/n/882242-1; WFO: http://www.worldfloraonline.org/taxon/wfo-0000871667; POWO: https://powo.science.kew.org/taxon/882242-1
= Festucaporciivar.hirsuta A.Nyár., Not. Bot. Cluj 2: 83 (1966); GBIF: https://www.gbif.org/species/6312934; WFO: http://www.worldfloraonline.org/taxon/wfo-0001098406; WFO: http://www.worldfloraonline.org/taxon/wfo-0000871668; POWO: https://powo.science.kew.org/taxon/416327-4
= Festucaporciif.longiaristata Krajina, Veröff. Geobot. Inst. ETH Stiftung Rübel Zürich 10: 32 (1933)
= Festucaporciif.vestita (Hack.) Krajina, Veröff. Geobot. Inst. ETH Stiftung Rübel Zürich 10: 31 (1933)
= Festucaporciivar.vestita Hack. ex Zapał., Consp. Fl. Galic. Crit. 1: 67 (1906)

##### Conservation status

In Ukraine – NT (Onyshchenko et al. 2022).

##### Distribution

SE Carpathian endemic.

##### Notes

A vulnerable species listed by the Red Book of Ukraine (Bednarska and Kagalo 2009, MEPNR of Ukraine 2021).

#### Festuca versicolor versicolor

Tausch, Flora 4(2): 559 (1821) et Tausch ex Kraj., Publ. Fac. Sc. Univ. Charles, Prague 106: 25 (1930), non J.Presl ex Kunth

17963831-C8E4-5FCD-A49A-8648119D74E7

https://www.catalogueoflife.org/data/taxon/5HCKN

https://www.gbif.org/species/6085148

urn:lsid:ipni.org:names:403706-1

urn:lsid:ipni.org:names:403707-1

http://www.worldfloraonline.org/taxon/wfo-0000872522

https://powo.science.kew.org/taxon/77173355-1

https://europlusmed.org/cdm_dataportal/taxon/0b134a8c-3aa4-4e34-bdd2-b82875b30957

https://www.worldplants.de/world-plants-complete-list/complete-plant-list/?name=Festuca-versicolor

https://www.biodiversitylibrary.org/page/42515894#page/414

https://w.jacq.org/W0033070

https://w.jacq.org/W0033071

https://wu.jacq.org/WU0062243

= *Festucaminor* Schur, Enum. Pl. Transsilv.: 795 (1866), non St.-Lag.; GBIF: https://www.gbif.org/species/8351667; IPNI: https://www.ipni.org/n/403091-1; POWO: https://powo.science.kew.org/taxon/403091-1; BHL: https://www.biodiversitylibrary.org/page/10544846#page/817
= Festucavariaf.acuminata Sagorski & Schneider, Fl. Centralkarp. 2: 554 (1891), non (Gaudin) Bolzon
= *Festucavaria* [unranked] *giewontica* Zapał., Consp. Fl. Galic. Crit. 1: 71 (1906)
= *Festucavaria* [unranked] *flavescens* Zapał., Consp. Fl. Galic. Crit. 1: 70 (1906), non Gaudin
= Festucavariasubsp.pumila [unranked] *spiculis flavescentibus* Gaudin ex Hack. in Sagorski & Schneider, Fl. Centralkarp. 2: 554 (1891)
= Festucavariavar.scopariaeformis Kotula, Rozmieszczenie roślin naczyniowych w Tatrach: 456 (1890)
= Festucaversicolorsubsp.eu-versicolorvar.genuinasubvar.rodnensis Krajina, Spisy Přír. Fak. Karlovy Univ. 106: 37 (1930)
= Festucaversicolorsubsp.eu-versicolorvar.genuinasubvar.rodnensisf.minor Krajina, Spisy Přír. Fak. Karlovy Univ. 106: 38 (1930)
= Festucaversicolorsubsp.eu-versicolorvar.genuinasubvar.rodnensisf.typica Krajina, Spisy Přír. Fak. Karlovy Univ. 106: 38 (1930)
= Festucaversicolorsubsp.eu-versicolorvar.genuinasubvar.transsilvanica Krajina, Spisy Přír. Fak. Karlovy Univ. 106: 38 (1930); JSTOR Global Plants: https://plants.jstor.org/stable/10.5555/al.ap.specimen.w19160032081
= Festucaversicolorsubsp.eu-versicolorvar.genuinasubvar.transsilvanica f. *Kotschyi* Krajina, Spisy Přír. Fak. Karlovy Univ. 106: 39 (1930)
= Festucaversicolorsubsp.eu-versicolorvar.genuinasubvar.transsilvanicaf.pallens Krajina, Spisy Přír. Fak. Karlovy Univ. 106: 39 (1930)
= Festucaversicolorsubsp.eu-versicolorvar.genuinasubvar.transsilvanicaf.typica Krajina, Spisy Přír. Fak. Karlovy Univ. 106: 39 (1930)
= Festucaversicolorsubsp.eu-versicolorvar.genuinasubvar.vulgaris Krajina, Spisy Přír. Fak. Karlovy Univ. 106: 31 (1930)
= Festucaversicolorsubsp.eu-versicolorvar.genuinasubvar.vulgarisf.chrysantha Krajina, Spisy Přír. Fak. Karlovy Univ. 106: 32 (1930)
= Festucaversicolorsubsp.eu-versicolorvar.genuinasubvar.vulgarisf.curvala Krajina, Spisy Přír. Fak. Karlovy Univ. 106: 32 (1930)
= Festucaversicolorsubsp.eu-versicolorvar.genuinasubvar.vulgarisf.debilis Krajina, Spisy Přír. Fak. Karlovy Univ. 106: 33 (1930)
= Festucaversicolorsubsp.eu-versicolorvar.genuinasubvar.vulgarisf.giewontica (Zapał.) Krajina, Spisy Přír. Fak. Karlovy Univ. 106: 33 (1930)
= Festucaversicolorsubsp.eu-versicolorvar.genuinasubvar.vulgarisf.glaucophylla Krajina, Spisy Přír. Fak. Karlovy Univ.: 33 (1930)
= Festucaversicolorsubsp.eu-versicolorvar.genuinasubvar.vulgarisf.mutica Krajina, Spisy Přír. Fak. Karlovy Univ. 106: 33 (1930)
= Festucaversicolorsubsp.eu-versicolorvar.genuinasubvar.vulgarisf.robustior Krajina, Spisy Přír. Fak. Karlovy Univ. 106: 32 (1930)
= Festucaversicolorsubsp.eu-versicolorvar.genuinasubvar.vulgarisf.scopariaeformis (Kotula) Krajina, Spisy Přír. Fak. Karlovy Univ. 106: 32 (1930)
= Festucaversicolorsubsp.eu-versicolorvar.genuinasubvar.vulgarisf.typica Krajina, Spisy Přír. Fak. Karlovy Univ. 106: 32 (1930)
= Festucaversicolorsubsp.eu-versicolorvar.genuinasubvar.vulgarisf.zapalowiczii Krajina, Spisy Přír. Fak. Karlovy Univ. 106: 33 (1930); JACQ: https://w.jacq.org/W19160013707; JSTOR Global Plants: https://plants.jstor.org/stable/10.5555/al.ap.specimen.w19160013707
= Festucaversicolorvar.minor (Schur) Krajina, Veröff. Geobot. Inst. ETH Stiftung Rübel Zürich 10: 40 (1933)
= Festucaversicolorsubsp.pseudosulcata Krajina, Spisy Přír. Fak. Karlovy Univ. 106: 43 (1930), non Drobow; JACQ: https://w.jacq.org/W19160038597; JSTOR Global Plants: https://plants.jstor.org/stable/10.5555/al.ap.specimen.w19160038597
= Festucaversicolorvar.versicolorf.chrysantha (Krajina) Beldie, Fl. Rep. Soc. Rom. 12: 491 (1972)
= Festucaversicolorvar.versicolorf.debilis (Krajina) Beldie, Fl. Rep. Soc. Rom. 12: 491 (1972)
– Festucavariaf.pallidula auct., non Hack.

##### Conservation status

In Ukraine – LC (Onyshchenko et al. 2022).

##### Distribution

Pancarpathian subendemic.

##### Notes

Two (https://powo.science.kew.org/taxon/403706-1, accessed on 05.06.2023) to four (https://europlusmed.org/cdm_dataportal/taxon/0c5f7c03-e2bc-42b0-8fd0-7dc8fcc48491, accessed on 05.06.2023) subspecies are recognised within F.versicolorTausch, includingsubsp.versicolor (occurs in the Carparpathians and rarely in the Sudetes – Kliment (1999)), subsp. dominii Krajina (endemic of the Rodna Mts. – Kliment et al. (2016)), subsp. pallidula (Hack.) Markgr.-Dann. (endemic of the Austrian Alps occurring on the rocks and screes up to 1700 m a.s.l. – Šmarda (2008), Essl et al. (2009)) and subsp. brachystachys (Hack.) Markgr.-Dann. (another endemic of the Austrian Alps occurring in the alpine habitats up to 2200 m a.s.l. – Essl et al. (2009)).

Euro+Med (https://europlusmed.org/cdm_dataportal/taxon/0b134a8c-3aa4-4e34-bdd2-b82875b30957, accessed on 05.06.2023) indicates the presence of F.versicolorsubsp.dominii in Ukraine, based on personal communication with B. Valdés in 2003. However, no confirmed evidence of its presence in Ukraine has appeared since then. In the original description of F.versicolorsubsp.dominii, Krajina (1930) mentioned the existence of this subspecies only in Rodna Mts. Šmarda et al. (2007) revised this subspecies as belonging to the non-endemic F.psammophilasubsp.dominii with distribution in Austria, Slovakia, the Czech Republic and Poland. At the same time, Šmarda et al. (2007) considered specimens of F.dominiivar.margittaii Krajina belonging to *F.vaginata* Waldst. & Kit. ex Willd., which is distributed in Austria, Slovakia, Hungary, Romania, Bulgaria and Croatia. Unfortunately, Šmarda et al. (2007) did not revise specimens from Ukraine. In Ukraine, the only discovered specimen of F.dominiivar.margittaii from the Szomotor Village (Hungary) is deposited at the KW Herbarium. Therefore, the presence of F.versicolorsubsp.dominii in Ukraine remains unclear.

#### Koeleria transsilvanica

Schur, Oesterr. Bot. Wochenbl. 7: 313 (1857), non Barth.

D13B0763-9A90-5B88-B91D-364467A9824F

https://www.gbif.org/species/4136486

urn:lsid:ipni.org:names:406727-1

http://www.worldfloraonline.org/taxon/wfo-0000877370

https://powo.science.kew.org/taxon/406727-1

https://europlusmed.org/cdm_dataportal/taxon/5170b18f-e88e-413f-ae0e-8f146fd69611

https://www.worldplants.de/world-plants-complete-list/complete-plant-list/?name=Koeleria-macrantha

https://www.biodiversitylibrary.org/page/29953152#page/314/mode/1up

≡ *Koeleriacristata* [unranked] d) *transsilvanica* (Schur) K.Richt., Pl. Eur. 1: 75 (1890); GBIF: https://www.gbif.org/species/9550970; IPNI: https://www.ipni.org/n/77260252-1; WFO: http://www.worldfloraonline.org/taxon/wfo-1200013144; POWO: https://powo.science.kew.org/taxon/77260252-1; BHL: https://www.biodiversitylibrary.org/page/10641492#page/81
≡ Koeleriacristatasubsp.ciliatavar.transsilvanica (Schur) Asch. & Graebn., Syn. Mitteleur. Fl. 2(1): 358 (1900); GBIF: https://www.gbif.org/species/9528021; IPNI: https://www.ipni.org/n/77280772-1; POWO: https://powo.science.kew.org/taxon/77280772-1; BHL: https://www.biodiversitylibrary.org/page/25261416#page/370
≡ Koeleriagracilissubsp.transsilvanica (Schur) Domin, Monographie d. Gattung Koeleria, Biblioth. Bot. 14(65): 239 (1907) et Flora Romaniae Exsiceatae; GBIF: https://www.gbif.org/species/7496966; IPNI: https://www.ipni.org/n/77260417-1; WFO: http://www.worldfloraonline.org/taxon/wfo-0001234481; POWO: https://powo.science.kew.org/taxon/77260417-1; BHL: https://www.biodiversitylibrary.org/page/47145556#page/257
≡ Koeleriagracilisvar.transsilvanica (Schur) Jáv., Magyar Fl.: 87 (1925)
≡ Koeleriamacranthasubsp.transsilvanica (Schur) Beldie, Fl. Rom. Det.: 342 (1977) [nom. illeg.]
≡ Koeleriamacranthasubsp.transsilvanica (Schur) A.Nyár. (1965) [nom. nudum ?]; CoL: https://www.catalogueoflife.org/data/taxon/5J7J2; GBIF: https://www.gbif.org/species/9358421; IPNI: https://www.ipni.org/n/77190062-1; WFO: http://www.worldfloraonline.org/taxon/wfo-0001428176; POWO: https://powo.science.kew.org/taxon/77190062-1
= Koeleriagracilisvar.rohlenae Domin, Biblioth. Bot. 65: 193 (1907); GBIF: https://www.gbif.org/species/5946130; IPNI: https://www.ipni.org/n/77280623-1; WFO: http://www.worldfloraonline.org/taxon/wfo-0000877079; POWO: https://powo.science.kew.org/taxon/77280623-1; BHL: https://www.biodiversitylibrary.org/page/47145510#page/211
= Koeleriagracilisvar.typica Domin, Biblioth. Bot. 65: 230 (1907); IPNI: https://www.ipni.org/n/290732-2; WFO: http://www.worldfloraonline.org/taxon/wfo-0001397716; BHL: https://www.biodiversitylibrary.org/page/47145510#page/248
= *Koeleriatenuipes* (Schur) Ujhelyi, Ann. Hist.-Nat. Mus. Natl. Hung. 57: 191 (1965); GBIF: https://www.gbif.org/species/4136561; IPNI: https://www.ipni.org/n/406721-1; WFO: http://www.worldfloraonline.org/taxon/wfo-0000877153; POWO: https://powo.science.kew.org/taxon/406721-1
= Koeleriatranssilvanicasubsp.tenuipes (Schur) Soó, Acta Bot. Acad. Sci. Hung. 17(1–2): 122 (1972); GBIF: https://www.gbif.org/species/4136485; IPNI: https://www.ipni.org/n/882519-1; WFO: http://www.worldfloraonline.org/taxon/wfo-0000877371; POWO: https://powo.science.kew.org/taxon/882519-1
= Koeleriatranssilvanicavar.tenuipes (Schur) Domin, Magyar Bot. Lapok 3: 259 (1904); GBIF: https://www.gbif.org/species/6313425; WFO: http://www.worldfloraonline.org/taxon/wfo-0000877372; POWO: https://powo.science.kew.org/taxon/421794-4; BHL: https://www.biodiversitylibrary.org/item/202566#page/281
= Koeleriatranssilvanicavar.tenuipesf.discolor Degen ex Domin, Magyar. Bot. Lap. 3: 259 (1904) et Bibl. Bot. 14: 240 (1907); BHL: https://www.biodiversitylibrary.org/item/202566#page/281; BHL: https://www.biodiversitylibrary.org/item/181274#page/258
= *Koeleriatranssilvanica* [unranked] a *tenuipesalpestris* Schur, Enum. Pl. Transsilv.: 750 (1866); BHL: https://www.biodiversitylibrary.org/item/7364#page/770
= *Koeleriatranssilvanica* [unranked] b *tenuipesalpestris* Schur, Oesterr. Bot. Wochenbl. 7: 313 (1857); BHL: https://www.biodiversitylibrary.org/item/94863#page/314
– *Koeleriacolorata* (Heuff.) Nyár. ex Degen, Gramina Hungarica: nr 368 (1911) [p. p.]; GBIF: https://www.gbif.org/species/8083069; WFO: http://www.worldfloraonline.org/taxon/wfo-0001098441
– Koeleriacristatavar.colorata Heuff., Verh. K.K. Zool.-Bot. Ges. Wien 8: 228 (1858) [p. p.]; GBIF: https://www.gbif.org/species/5946095; IPNI https://www.ipni.org/n/77279977-1; WFO: http://www.worldfloraonline.org/taxon/wfo-0000876905; POWO: https://powo.science.kew.org/taxon/77279977-1; BHL: https://www.biodiversitylibrary.org/item/137035#page/412
– Koeleriacristatavar.glabra Kotschy [ex herb., nom. nudum], non alior.
– *Koeleriacristata* [unranked] *foliis vaginisque glabris* Andrä [ex herb., nom. nudum]
– *Koeleriagracilis* Pers, Syn. Pl. [Persoon] 1: 97 (1805) [p. p., ex herb.], non Guss.; GBIF: https://www.gbif.org/species/2705920; IPNI: https://www.ipni.org/n/406567-1; WFO: http://www.worldfloraonline.org/taxon/wfo-0000877004; POWO: https://powo.science.kew.org/taxon/406567-1; BHL: https://www.biodiversitylibrary.org/page/234882#page/109
– Koeleriagracilisf.colorata (Heuff.) Domin, Biblioth. Bot. 65: 232 (1907) [p. p.]; GBIF: https://www.gbif.org/species/4137722; IPNI: https://www.ipni.org/n/290733-2; WFO: http://www.worldfloraonline.org/taxon/wfo-0000877009; POWO: https://powo.science.kew.org/taxon/290733-2; BHL: https://www.biodiversitylibrary.org/page/47145549#page/250
– Koeleriagracilisvar.colorata (Heuff.) Domin, Magyar Bot. Lapok 3: 268 (1904) [p. p.], non alior; BHL: https://www.biodiversitylibrary.org/item/202566#page/290
– Koeleriamacranthavar.colorata (Heuff.) Ghisa, Fl. Rep. Soc. Rom. 12: 237 (1972) [p. p.]; GBIF: https://www.gbif.org/species/4137206; IPNI: https://www.ipni.org/n/882504-1; WFO: http://www.worldfloraonline.org/taxon/wfo-0000877163; POWO: https://powo.science.kew.org/taxon/882504-1
– *Koeleriasetacea* DC. sensu Nyman, Consp. Fl. Eur.: 816 (1878–1882); BHL: https://www.biodiversitylibrary.org/page/11015136#page/827

##### Conservation status

In Ukraine – DD (Onyshchenko et al. 2022).

##### Distribution

SE Carpathian endemic.

##### Notes

Deyl (1934) and Deyl (1940) indicated this species for Petros Mt. in the Ukrainian part of the Maramures Mts. However, there are neither recent field confirmations (Kricsfalusy and Budnikov 2007, Kobiv et al. 2017) nor herbarium vouchers discovered during my investigations. Therefore, the presence of this species in the flora of the Ukrainian Carpathians is questionable.

GBIF (https://www.gbif.org/species/7262109), POWO (https://powo.science.kew.org/taxon/77188152-1, accessed on 05.06.2023), Euro+Med (https://europlusmed.org/cdm_dataportal/taxon/5170b18f-e88e-413f-ae0e-8f146fd69611, accessed on 05.06.2023) and WFO (https://list.worldfloraonline.org/wfo-0000877153, accessed on 05.06.2023) consider *K.tenuipes* (Schur) Ujhelyi and its homotypic derivates as synonyms for K.macranthasubsp.macrantha. However, Ujhelyi (1965), on pages 191–193, indicated that this species occurs exclusively in Transylvania and South Carpathians (Făgăraș Mts.). Ujhelyi (1965) noted that *K.tenuipes* differs from *K.transsilvanica* by generally larger sizes, larger leaves, larger and loose panicles and larger spikelet parts, but similar by lack of stomata in the coastal zone of the juvenile leaves and ciliate auriculae of the sheaths. Moreover, the mentioned databases confuse the authorship of the epithet ‘*tenuipes’.* Ujhelyi (1965) pointed out that it was Schur who first applied this epithet in 1857, while Domin applied it only in 1903 when he described K.transsilvanicavar.tenuipesf.hirsuta (Ujhelyi re-identified Domin’s plants as *K.eriostachya* Pancic).

#### Poa carpatica carpatica

(V.Jirásek) Bernátová, Májovský, Kliment & Topercer, Biologia (Bratislava), Sect. Bot. 61(4): 389-390 (2006)

3E8F9EAA-6613-5320-A6A8-AC56C0C4B51E

urn:lsid:ipni.org:names:77084794-1

http://www.worldfloraonline.org/taxon/wfo-0000908275

https://powo.science.kew.org/taxon/77084794-1

https://europlusmed.org/cdm_dataportal/taxon/3cdfaf21-83dd-4c76-96bf-a7333f269f7f

https://www.worldplants.de/world-plants-complete-list/complete-plant-list/?name=Leucopoa-carpatica

≡ *Poacarpatica* (V.Jirásek) Chopik, Visokogirna Fl. Ukrains’k. Karpat: 174 (1976); CoL: https://www.catalogueoflife.org/data/taxon/4KLGZ; GBIF: https://www.gbif.org/species/4137163; IPNI: https://www.ipni.org/n/77084794-1; WFO: http://www.worldfloraonline.org/taxon/wfo-0000908275; POWO: https://powo.science.kew.org/taxon/77084794-1; JACQ: https://prc.jacq.org/PRC450819; JACQ: https://prc.jacq.org/PRC450820; JACQ: https://prc.jacq.org/PRC450821; JACQ: https://prc.jacq.org/PRC450822; JACQ: https://prc.jacq.org/PRC450823; JSTOR Global Plants: https://plants.jstor.org/stable/10.5555/al.ap.specimen.prc450823; JSTOR Global Plants: https://plants.jstor.org/stable/10.5555/al.ap.specimen.prc450822; JSTOR Global Plants: https://plants.jstor.org/stable/10.5555/al.ap.specimen.prc450820
≡ Poanemoralissubsp.carpatica V.Jirásek, Veda Prir. 15: 207 (1934) *; GBIF: https://www.gbif.org/species/6313736; IPNI: https://www.ipni.org/n/77084793-1; WFO: http://www.worldfloraonline.org/taxon/wfo-0000908275; POWO: https://powo.science.kew.org/taxon/77084793-1
≡ Poanemoralissubsp.nemoralisvar.carpatica (V.Jirásek) Soó, Acta Bot. Acad. Sci. Hung. 17(1–2): 118 (1972); GBIF: https://www.gbif.org/species/4116723; IPNI: https://www.ipni.org/n/882819-1; WFO: http://www.worldfloraonline.org/taxon/wfo-0000908275; POWO: https://powo.science.kew.org/taxon/882819-1
= Poabalfouriif.carpatica Zapał., Spraw. Komis. Fizjogr. 39: 33 (1906)
= Poanemoralissubsp.carpaticaf.minoriformis V.Jirásek, Veda Prir. 15: 208 (1934); JSTOR Global Plants: https://plants.jstor.org/stable/10.5555/al.ap.specimen.prc450819; JSTOR Global Plants: https://plants.jstor.org/stable/10.5555/al.ap.specimen.prc450821
– *Poabalfourii* auct. fl. ucrain. carpat., non Parn. *
– *Poajanczewskii* auct., non Zapał. [tantum quod plantas ucrain. carpat., alp. et subalp. altitud. solum]
– Poanemoralissubsp.montana auct., non (Gaudin) Chrtek & V.Jirásek
– Poanemoralisvar.montana auct. fl. ucrain. carpat., non Gaudin *

##### Conservation status

In Ukraine – LC (Onyshchenko et al. 2022).

##### Distribution

Pancarpathian endemic.

##### Notes

Two subspecies of *P.carpatica* (V.Jirásek) Chopik are recognised: subsp. carpatica (endemic to the Western and Eastern Carpathians – Bernátová et al. (2006)) and subsp. supramontana Bernátová, Májovský, Kliment & Topercer (narrow endemic to the Veľká Fatra Mts and Krivánska Malá Fatra Mts – Bernátová et al. (2006)).

Chopyk and Fedoronchuk (2015), on page 567, mention *P.janczewskii* Zapał. for rocks and screes in subalpine and alpine belts of the Ukrainian Carpathians, with synonyms *P.balfourii* auct., non Parn. and P.nemoralissubsp.carpatica V.Jirásek. At the same time, POWO (https://powo.science.kew.org/taxon/204398-2, accessed on 05.06.2023), WFO (https://list.worldfloraonline.org/wfo-0000893213, accessed on 05.06.2023) and Worldplants (https://www.worldplants.de/world-plants-complete-list/complete-plant-list?name=Poa-palustris, accessed on 07.06.2023), as well as Tzvelev (1974), Tzvelev (1976) and Tzvelev (1995) suggest that *P.janczewskii* is a synonym not for *P.carpatica*, but for *P.palustris* L. Chopyk and Fedoronchuk (2015), on page 568, also independently recognise *P.palustris*, but indicate that it is widely distributed in the forests (i.e. lower altitudes), flooded meadows and other wet habitats of the Ukrainian Carpathians. Hence, Chopyk and Fedoronchuk (2015) delimit *P.janczewskii* and *P.palustris* by morphology and habitat preferences. In the original protologue of *P.janczewskii*, Zapałowicz (1906a), on pages 34–35, wrote that it occurs in wet places at the beginning of the River Chorniy Cheremosh near Mt. Koman in Chyvchyny Mts. at 1700 m altitude together with P.nemoralisvar.pocutica Zapał. Simultaneously, Zapałowicz (1906a), on page 33, delimited P.balfouriif.carpatica Zapał. from the alpine and subalpine habitats. Hence, Zapałowicz’s original description of *P.janczewskii* is close to *P.palustris*, while his consideration of *P.balfourii* is consistent with *P.carpatica*. Nevertheless, in the Ukrainian Carpathians, many specimens from the alpine and subalpine belts are identified as *P.janczewskii*, suggesting that this species can probably reach much higher altitudes and can be confused with *P.carpatica*. Therefore, I include *P.janczewskii* as a misapplied synonym of P.carpaticasubsp.carpatica.

#### Poa granitica disparillis

(Nyár.) Nyár., Rev. Roumaine Biol., Sér. Bot. 10: 355 (1965)

B700E591-0895-529B-9946-8BB69BA7D759

https://www.catalogueoflife.org/data/taxon/5KJCS

https://www.gbif.org/species/5947645

urn:lsid:ipni.org:names:77189564-1

http://www.worldfloraonline.org/taxon/wfo-0001250652

https://powo.science.kew.org/taxon/77189564-1

https://europlusmed.org/cdm_dataportal/taxon/4479d756-9abe-4d15-a1a4-5d97bffc921e

https://www.worldplants.de/world-plants-complete-list/complete-plant-list/?name=Poa-granitica

≡ Poacenisiasubsp.graniticavar.disparillis (Nyár.) Nyár. & Borza, Consp. Fl. Roman. 1: 16 (1947); WFO: http://www.worldfloraonline.org/taxon/wfo-0001284854
≡ Poagraniticavar.disparillis Nyár., Veröff. Geobot. Inst. Rübel Zürich 10: 173 (1933); WFO: http://www.worldfloraonline.org/taxon/wfo-0001250690
= *Poabreazensis* Nyár., Veröff. Geobot. Inst. Rübel Zürich 10: 173 (1933); GBIF: https://www.gbif.org/species/4136051; IPNI: https://www.ipni.org/n/416647-1; WFO: http://www.worldfloraonline.org/taxon/wfo-0000891305; POWO: https://powo.science.kew.org/taxon/416647-1
= *Poacenisia* [unranked] b *pietrosuana* Zapał., Consp. Fl. Gallic. Crit. 6: 227 (1911) *; GBIF: https://www.gbif.org/species/5947644; IPNI: https://www.ipni.org/n/77288143-1; WFO: http://www.worldfloraonline.org/taxon/wfo-0000891923; POWO: https://powo.science.kew.org/taxon/77288143-1
= *Poadeylii* Chrtek & V.Jirásek, Feddes Repert. Spec. Nov. Regni Veg. 69: 177 (1964) *; GBIF: https://www.gbif.org/species/4124344; IPNI: https://www.ipni.org/n/416895-1; WFO: http://www.worldfloraonline.org/taxon/wfo-0000892132; POWO: https://powo.science.kew.org/taxon/416895-1
= Poadeyliivar.deyliif.breazensis (Nyár.) Ghișa & Beldie, Fl. Rep. Soc. Rom. 12: 399 (1972); GBIF: https://www.gbif.org/species/4124343; IPNI: https://www.ipni.org/n/882798-1; WFO: http://www.worldfloraonline.org/taxon/wfo-0000892133; POWO: https://powo.science.kew.org/taxon/882798-1
= Poadeyliivar.deyliif.pietrosuana (Zapał.) Ghișa & Beldie, Fl. Rep. Soc. Rom. 12: 399 (1972); GBIF: https://www.gbif.org/species/4139450; IPNI: https://www.ipni.org/n/882799-1; WFO: http://www.worldfloraonline.org/taxon/wfo-0000892134; POWO: https://powo.science.kew.org/taxon/882799-1
= Poadeyliivar.deyliif.subgranitica (Nyár.) Ghișa & Beldie, Fl. Rep. Soc. Rom. 12: 399 (1972); GBIF: https://www.gbif.org/species/4139468; IPNI: https://www.ipni.org/n/882801-1; WFO: http://www.worldfloraonline.org/taxon/wfo-0000892135; POWO: https://powo.science.kew.org/taxon/882801-1
= Poadeyliisubsp.retezatensis (A.Nyár.) Chrtek, Oesterr. Bot. Z., 115 (4–5): 424 (1968); GBIF: https://www.gbif.org/species/7826350
= Poadeyliivar.retezatensis (A.Nyár.) Ghișa & Beldie, Fl. Rep. Soc. Rom. 12: 399 (1972); GBIF: https://www.gbif.org/species/4139461; IPNI: https://www.ipni.org/n/882800-1; WFO: http://www.worldfloraonline.org/taxon/wfo-0000892136; POWO: https://powo.science.kew.org/taxon/882800-1
= Poagraniticavar.disparillisf.pietrosuana (Zapał.) Nyár., Veröff. Geobot. Inst. Rübel Zürich 10: 173 (1933)
= Poagraniticasubsp.disparillisvar.subgranitica Nyár., Rev. Roumaine Biol., Sér. Bot. 10: 355 (1965); GBIF: https://www.gbif.org/species/5947643; IPNI: https://www.ipni.org/n/77288847-1; WFO: http://www.worldfloraonline.org/taxon/wfo-0000892475; POWO: https://powo.science.kew.org/taxon/77288847-1
= Poagraniticasubsp.retezatensis A.Nyár., Rev. Roumaine Biol., Sér. Bot. 10: 356 (1965); GBIF: https://www.gbif.org/species/5947642; IPNI: https://www.ipni.org/n/77260385-1; WFO: http://www.worldfloraonline.org/taxon/wfo-0000892474; POWO: https://powo.science.kew.org/taxon/77260385-1
= Poagraniticasubsp.subcarpatica (V.Jirásek) Fodor, Flora Zakarpattia: 182 (1974)
= Poagraniticavar.subcarpatica V.Jirásek, Vest. Král. Ceské Spol. Náuk 1935: 11 (1936)
= Poagraniticavar.typica Nyár., Veröff. Geobot. Inst. Rübel Zürich 10: 171–172 (1933)
= Poagraniticavar.typicaf.deminuta Nyár., Veröff. Geobot. Inst. Rübel Zürich 10: 172 (1933)
– *Poacenisia* All., Auct. Fl. Pedem.: 40 (1789) [p. p., tantum quod plantas ucrain. carpat.]; GBIF: https://www.gbif.org/species/4137228; IPNI: https://www.ipni.org/n/30482705-2; WFO: http://www.worldfloraonline.org/taxon/wfo-0000891913; POWO: https://powo.science.kew.org/taxon/30482705-2; BHL: https://www.biodiversitylibrary.org/page/52551645#page/52
– *Poagranitica* Braun-Blanq., Arch. Bot., Caen, Bull. 3: 46 (1929) [p. p., tantum quod plantas ucrain. carpat.] *; GBIF: https://www.gbif.org/species/4121637; IPNI: https://www.ipni.org/n/417145-1; WFO: http://www.worldfloraonline.org/taxon/wfo-0000892473; POWO: https://powo.science.kew.org/taxon/417145-1
– Poagraniticasubsp.granitica Braun-Blanq., Arch. Bot., Caen, Bull. 3: 46 (1929) sensu Tasenkevich [non sensu orig, ex herb. LWS] *

##### Conservation status

In Ukraine – LC (Onyshchenko et al. 2022). Global – DD (Bilz 2011b).

##### Distribution

SE Carpathian endemic.

##### Notes

Two (https://powo.science.kew.org/taxon/417145-1, accessed on 05.06.2023) to three (https://europlusmed.org/cdm_dataportal/taxon/b36321f1-3c80-4639-aa91-71b74301d317, accessed on 05.06.2023) subspecies are recognised within P.graniticaBraun-Blanq. vizsubsp.granitica(distributed in the Polish and Slovakian Carpathians),subsp.disparillis (= *P.deylii* Chrtek & V.Jirásek; distributed in the Polish, Ukrainian and Romanian Carpathians) and subsp. retezatensis Nyár. (distributed exclusively in the Romanian Carpathians and is sometimes considered a synonym of P.graniticasubsp.disparillis).

POWO (https://powo.science.kew.org/taxon/417145-1, accessed on 05.06.2023) indicates the presence of both subspecies (i.e. subsp. granitica and subsp. disparillis) in the Ukrainian Carpathians. These two subspecies seem to be phylogenetically close, but considered to be geographically isolated (Chrtek and Jirásek 1964, Filipaş et al. 2009, Băcilă et al. 2010). However, there are no confirmed occurrences of P.graniticasubsp.granitica from the Ukrainian Carpathians yet. Only a few specimens in LWS were identified by Tasenkevich as *P.granitica* subsp. granitica (without further published mentions), but she also indicated on the specimens' labels that P.graniticasubsp.granitica is a synonym of *P.deylii*.

#### Poa pannonica scabra

(Asch.) Soó, Acta Bot. Acad. Sci. Hung. 5: 483 (1959)

35AF9FF6-717D-5295-A75D-E9A0A3CD185D

https://www.catalogueoflife.org/data/taxon/7KNSV

https://www.gbif.org/species/4932360

urn:lsid:ipni.org:names:77188273-1

http://www.worldfloraonline.org/taxon/wfo-0000893233

https://powo.science.kew.org/taxon/77188273-1

https://europlusmed.org/cdm_dataportal/taxon/c6d5f874-9541-44c9-8061-0101aaae6d93

https://www.worldplants.de/world-plants-complete-list/complete-plant-list/?name=Poa-pannonica

≡ Poapratensisvar.scabra (Asch.) Asch. & Graebn., Syn. Mitteleur. Fl. 2(1): 414 (1900); GBIF: https://www.gbif.org/species/5947834; IPNI: https://www.ipni.org/n/77288290-1; WFO: http://www.worldfloraonline.org/taxon/wfo-0000893466; POWO: https://powo.science.kew.org/taxon/77288290-1; BHL: https://www.biodiversitylibrary.org/page/25261472#page/426
≡ *Poascabra* Asch., Verh. K.K. Zool.-Bot. Ges. Wien 17: 568 (1867), non Ehrh.; GBIF: https://www.gbif.org/species/7933600; BHL: https://www.biodiversitylibrary.org/item/86029#page/772
≡ *Poascabra* Kit. ex Steud., Nomencl. Bot. [Steudel], ed. 2. 2: 362 et Linnaea, 32: 311 (1863) [nom. nudum], non Ehrh. *; GBIF: https://www.gbif.org/species/8481689; GBIF: https://www.gbif.org/species/8162992; IPNI: https://www.ipni.org/n/417977-1; WFO: http://www.worldfloraonline.org/taxon/wfo-0000893691; WFO: http://www.worldfloraonline.org/taxon/wfo-0001250823; POWO: https://powo.science.kew.org/taxon/417977-1; BHL: https://www.biodiversitylibrary.org/page/392077#page/1218
≡ Poasterilissubsp.eu-sterilisvar.scabra (Asch.) Asch. & Graebn., Syn. Mitteleur. Fl. 2(1): 414 (1900), non alior; WFO: http://www.worldfloraonline.org/taxon/wfo-0001250670
= *Poaperscabra* Holub, Folia Geobot. Phytotax. 18(2): 204 (1983); GBIF: https://www.gbif.org/species/4114872; IPNI: https://www.ipni.org/n/907580-1; WFO: http://www.worldfloraonline.org/taxon/wfo-0000893298; POWO: https://powo.science.kew.org/taxon/907580-1
= *Poasterilis* Kerner, Oesterr. Bot. Z. 14: 85 (1864), non M.Bieb.; BHL: https://www.biodiversitylibrary.org/item/91290#page/92

##### Conservation status

In Ukraine – NE (Onyshchenko et al. 2022).

##### Distribution

Pancarpathian subendemic.

##### Notes

Following POWO (https://powo.science.kew.org/taxon/417674-1, accessed on 05.06.2023), there are two subspecies of P.pannonicaA.Kern. –subsp.pannonica(distributed in Serbia, Moldova, Hungary, Romania, Slovakia and, probably, Ukraine) andsubsp.scabra (distributed in Slovakia, Hungary, Romania and, presumably, Ukraine). Both subspecies are mentioned for Ukraine in most online databases. However, reports of P.pannonicasubsp.pannonica are, instead, related to *P.podolica* (Asch. & Graebn.) Błocki ex Zapał., the taxonomic status of which is unclear since it is considered a synonym of P.versicolorBess.subsp.versicolor by Tzvelev (1976) on page 472 and Ghişa and Beldie (1972) on page 407. Regarding P.pannonicasubsp.scabra, there is no recent evidence of its presence in the flora of the Ukrainian Carpathians (Chopyk and Fedoronchuk 2015, Kliment et al. 2016).

In online databases, the authorship of the epithet *scabra* is cited as Ascherson & Graebner (e.g. P.pannonicasubsp.scabra (Asch. & Graebn.) Soó). This is incorrect because it was Ascherson (1867) who, on page 568, validly published the name *P.scabra* and, only later, it reappeared in the synopsis of Ascherson and Graebner (1896). Moreover, IPNI (https://www.ipni.org/n/77288290-1, accessed on 05.06.2023) gives an incorrect nomenclature citation P.pratensisvar.scabra Asch. & Graebn., Syn. Mitteleur. Fl. 2(1): 414 (1900) that should be avoided. Ascherson and Graebner (1896) did not apply such a combination; instead, they used P.sterilissubsp.eu-sterilisvar.scabra.

#### Poa rehmannii

(Asch. et Graebn.) Woł., Fl. Polon. Exs., 10–11: Nr 1020 (1904)

4A24C5EE-2CB2-5280-9E07-A05526A56706

https://www.gbif.org/species/8323860

urn:lsid:ipni.org:names:417893-1

http://www.worldfloraonline.org/taxon/wfo-0000893588

https://powo.science.kew.org/taxon/417893-1

https://europlusmed.org/cdm_dataportal/taxon/d953d4b2-c291-43ab-a284-e929b36ba37f

https://www.worldplants.de/world-plants-complete-list/complete-plant-list/?name=Poa-rehmannii

≡ *Poacaesia* [unranked] d) *rehmannii* K.Richt., Pl. Eur. 1: 83 (1890); BHL: https://www.biodiversitylibrary.org/item/40428#page/89
≡ Poanemoralissubsp.rehmannii Asch. & Graebn., Syn. Mitteleur. Fl. 2(1): 412 (1900); GBIF: https://www.gbif.org/species/5947595; IPNI: https://www.ipni.org/n/77260518-1; WFO: https://list.worldfloraonline.org/wfo-0000893079; POWO: https://powo.science.kew.org/taxon/77260518-1; BHL: https://www.biodiversitylibrary.org/page/25261470#page/424
≡ *Poarehmannii* (Asch. & Graebn.) K.Richt., Pl. Eur. 1: 83 (1889) [nom. nudum]; CoL: https://www.catalogueoflife.org/data/taxon/4KMN8; GBIF: https://www.gbif.org/species/4112622; IPNI: https://www.ipni.org/n/417893-1; WFO: http://www.worldfloraonline.org/taxon/wfo-0000893588; POWO: https://powo.science.kew.org/taxon/417893-1; BHL: https://www.biodiversitylibrary.org/item/40428#page/89
= *Poaanceps* Rehmann, Spraw. Komis. Fizjogr. 7: 5 (1873), non G.Forst.; GBIF: https://www.gbif.org/species/7789150; IPNI: https://www.ipni.org/n/416483-1; WFO: http://www.worldfloraonline.org/taxon/wfo-0000891485; POWO: https://powo.science.kew.org/taxon/416483-1
– *Poarehmannii* Asch. & Gürke sensu Woł. [nom. confus., ex herb. LWS]

##### Conservation status

In Ukraine – VU (Onyshchenko et al. 2022).

##### Distribution

SE Carpathian endemic.

##### Notes

A rare species with only a few known occurrences in the Ukrainian Carpathians (Chorney et al. 2009, MEPNR of Ukraine 2021).

POWO (https://powo.science.kew.org/taxon/urn:lsid:ipni.org:names:417893-1, accessed on 05.06.2023), CoL (https://www.catalogueoflife.org/data/taxon/4KMN8, accessed on 05.06.2023) and GBIF (https://www.gbif.org/species/4112622, accessed on 05.06.2023) incorrectly provide the nomenclature citation *P.rehmannii* (Asch. & Graebn.) K.Richt., Pl. Europ. 1: 83 (1889). Richter (1889) did not apply such a name to the rank of species. Instead of this, he delimited unranked taxon (= variety) within *P.caesia* Sm. Therefore, the correct citation should be *P.caesia* [unranked] d) *rehmanii* K.Richt., Pl. Eur. 1: 83 (1890).

#### Sesleria bielzii

Schur, Verh. Mitth. Siebenbürg. Vereins Naturwiss. Hermannstadt 1: 109 (1850) et Verh. Mitth. Siebenbürg. Vereins Naturwiss. Hermannstadt 4: 84 (1853)

192C3A4C-8EF9-5087-967B-504A32C32BE8

https://www.catalogueoflife.org/data/taxon/4WZRV

https://www.gbif.org/species/4119647

urn:lsid:ipni.org:names:421321-1

http://www.worldfloraonline.org/taxon/wfo-0000898769

https://powo.science.kew.org/taxon/421321-1

https://europlusmed.org/cdm_dataportal/taxon/e8373e56-70c0-40aa-8e9d-6e546996ae0b

https://www.worldplants.de/world-plants-complete-list/complete-plant-list/?name=Sesleria-caerulans

https://www.biodiversitylibrary.org/page/11525300#page/119

https://www.biodiversitylibrary.org/page/11525300#page/970

≡ Sesleriacoerulanssubsp.bielzii (Schur) Gergely & Beldie, Fl. Rep. Soc. Rom. 12: 223 (1972) *; GBIF: https://www.gbif.org/species/4119544; IPNI: https://www.ipni.org/n/883025-1; WFO: http://www.worldfloraonline.org/taxon/wfo-0000898813; POWO: https://powo.science.kew.org/taxon/883025-1
≡ *Sesleriarigida* [unranked] β *bielzii* (Schur) Heuff., Enum. Pl. Banat.: 227 (1858); BHL: https://www.biodiversitylibrary.org/item/40084#page/195
= *Sesleriacaerulea* Janka, Linnaea 30: 615 (1859), non Ard.
= *Sesleriacapitata* (Schur) Schur, Enum. Pl. Transsilv.: 743 (1866); GBIF: https://www.gbif.org/species/4119575; IPNI: https://www.ipni.org/n/421326-1; WFO: http://www.worldfloraonline.org/taxon/wfo-0000898807; POWO: https://powo.science.kew.org/taxon/421326-1; BHL: https://www.biodiversitylibrary.org/page/10544794#page/765
= Sesleriacoerulansvar.borsae Deyl, Opera Bot. Čechina 3: 139 (1946); GBIF: https://www.gbif.org/species/5948416; IPNI: https://www.ipni.org/n/77291267-1; WFO: http://www.worldfloraonline.org/taxon/wfo-0000898814; POWO: https://powo.science.kew.org/taxon/77291267-1
= Sesleriacoerulansf.pseudorigida (Schur) Beldie, Bul. Şt. Acad. R.P.R. 2(5): 248 (1950)
= *Sesleriahaynaldiana* [unanked] g *pseudorigida* Schur, Verh. K.K. Zool.-Bot. Ges. Wien 6: 209 (1856); GBIF: https://www.gbif.org/species/5948414; IPNI: https://www.ipni.org/n/77291730-1; WFO: http://www.worldfloraonline.org/taxon/wfo-0000898840; POWO: https://powo.science.kew.org/taxon/77291730-1; BHL: https://www.biodiversitylibrary.org/openurlmultiple.aspx?id=p16414644|p42929929|p11712977
= *Sesleriapseudorigida* Schur, Enum. Pl. Transsilv.: 745 (1866); GBIF: https://www.gbif.org/species/4118994; IPNI: https://www.ipni.org/n/421386-1; WFO: http://www.worldfloraonline.org/taxon/wfo-0000898890; POWO: https://powo.science.kew.org/taxon/421386-1; BHL: https://www.biodiversitylibrary.org/page/10544796#page/767
= *Sesleriarigida* Griseb., Arch. Naturgesch. (Berlin) 18(1): 361 (1852), non Heuff. ex Rchb.; BHL: https://www.biodiversitylibrary.org/item/31352#page/369
= *Sesleriarigida* [unranked] a capitata Schur, Verh. K.K. Zool.-Bot. Ges. Wien 6: 201 (1856); BHL: https://www.biodiversitylibrary.org/item/137255#page/359
= *Sesleriarigida* [unranked] b *ovoidea* Schur, Verh. K.K. Zool.-Bot. Ges. Wien 6: 201 (1856); BHL: https://www.biodiversitylibrary.org/item/137255#page/359
– *Sesleriacaerulea* Scap. sensu Rehman [nom. confus. ex herb. LWS] *
– *Sesleriacoerulans* Friv., Flora 19(2): 438 (1836) [p. p., tantum quod plantas ucrain. carpat.] *; GBIF: https://www.gbif.org/species/4119531; IPNI: https://www.ipni.org/n/421329-1; WFO: http://www.worldfloraonline.org/taxon/wfo-0000898810; POWO: https://powo.science.kew.org/taxon/421329-1; BHL: https://www.biodiversitylibrary.org/page/34154#page/58

##### Conservation status

In Ukraine – LC (Onyshchenko et al. 2022).

##### Distribution

SE Carpathian endemic.

##### Notes

A problematic taxon with unclear chorology and phylogeny. Ambiguous interpretation of *S.bielzii* has been pointed out in the Flora of Romania (Gergely and Beldie 1972), where two subspecies of S.coerulansFriv. are represented –subsp.coerulansandsubsp.bielzii (Schur) Gergely & Beldie. For both subspecies, *S.bielzii* has been indicated as a synonym with the only difference being in its consideration by Shur – *S.bielzii* Schur, Verh. Mitth. Siebenbürg. Vereins Naturwiss. Hermannstadt 1: 109 (1850) has been indicated as a synonym for S.coerulanssubsp.bielzii, while *S.bielzii* Schur, Enum. Pl. Transsilv.: 743 (1866) has been indicated as a synonym for *S.coerulanssubsp.coerulans* Later, Chorney (2011) noted that *S.bielzii*, being, in fact, a Carpatho-Balcanic species, is erroneously considered endemic and referenced to Deyl (1980). Tzvelev (1976) and Chopyk and Fedoronchuk (2015) also considered *S.bielzii* a synonym of the non-endemic *S.coerulans*. On the other hand, Comănescu and Štefănuţ (2010) showed that these two species have similar distribution ranges; nevertheless, they treated these two species independently. Similarly, Lazarević et al. (2015), Kuzmanović et al. (2015) and Kuzmanović et al. (2017) conducted phylogenetic studies with *S.bielzii* considered an independent species within *the Coerulans* group.

#### Sesleria heufleriana heufleriana

Schur, Verh. Mitth. Siebenbürg. Vereins Naturwiss. Hermannstadt 4: 84 (1853) et Verh. Zool.-Bot. Ges. Wien 6: 203 (1856)

F9BF150E-561B-5A74-B2C9-B511914BDE5C

https://www.catalogueoflife.org/data/taxon/5L62R

https://www.gbif.org/species/9445568

urn:lsid:ipni.org:names:421353-1

urn:lsid:ipni.org:names:421352-1

http://www.worldfloraonline.org/taxon/wfo-0001428678

https://powo.science.kew.org/taxon/421353-1

https://species.wikimedia.org/wiki/Sesleria_heufleriana

https://europlusmed.org/cdm_dataportal/taxon/33685685-5a35-47ea-851e-4c6506dce56f

https://www.worldplants.de/world-plants-complete-list/complete-plant-list/?name=Sesleria-heufleriana

https://www.biodiversitylibrary.org/page/16414642#page/367

https://www.biodiversitylibrary.org/page/11525300#page/970

https://w.jacq.org/W0030257

https://w.jacq.org/W0030258

https://w.jacq.org/W19120010887

https://w.jacq.org/W19490014837

https://w.jacq.org/W18970006748

https://plants.jstor.org/stable/10.5555/al.ap.specimen.w0030257

https://plants.jstor.org/stable/10.5555/al.ap.specimen.ny01842943

https://plants.jstor.org/stable/10.5555/al.ap.specimen.w19490014837

https://plants.jstor.org/stable/10.5555/al.ap.specimen.w0030258

https://plants.jstor.org/stable/10.5555/al.ap.specimen.b%2010%200367408

≡ *Sesleriaheufleriana* Schur, Verh. Mitth. Siebenbürg. Vereins Naturwiss. Hermannstadt 4: 84 (1853) et Verh. Zool.-Bot. Ges. Wien 6: 203 (1856); GBIF: https://www.gbif.org/species/4119290; IPNI http://ipni.org/urn:lsid:ipni.org:names:421353-1; IPNI: http://ipni.org/urn:lsid:ipni.org:names:421352-1; WFO: http://www.worldfloraonline.org/taxon/wfo-0000898845; POWO: https://powo.science.kew.org/taxon/421353-1; BHL: https://www.biodiversitylibrary.org/page/16414642#page/367; BHL: https://www.biodiversitylibrary.org/page/11525300#page/970
≡ *Sesleriaheufleriana* Schur ex Błocki, Oesterr. Bot. Z. 39: 155 (1889) [nom. inval.] *; IPNI: https://www.ipni.org/n/421354-1; BHL: https://www.biodiversitylibrary.org/openurlmultiple.aspx?id=p28750462|p8721928
= *Sesleriacaerulea* [unranked] a interrupta Schur, Enum. Pl. Transsilv.: 743 (1866); GBIF: https://www.gbif.org/species/5948424; IPNI: https://www.ipni.org/n/77291477-1; WFO: http://www.worldfloraonline.org/taxon/wfo-0000898788; POWO: https://powo.science.kew.org/taxon/77291477-1; BHL: https://www.biodiversitylibrary.org/page/10544794#page/765
= *Sesleriacaerulea* [unranked] b *prorepens* Schur, Enum. Pl. Transsilv.: 743 (1866); GBIF: https://www.gbif.org/species/5948423; IPNI: https://www.ipni.org/n/77291959-1; WFO: http://www.worldfloraonline.org/taxon/wfo-0000898790; POWO: https://powo.science.kew.org/taxon/77291959-1; BHL: https://www.biodiversitylibrary.org/page/10544794#page/765
= *Sesleriacaerulea* [unranked] c *praelonga* Schur, Enum. Pl. Transsilv.: 743 (1866); BHL: https://powo.science.kew.org/taxon/77291959-1
= Sesleriacaeruleavar.transilvanica (Schur) Jáv., Magyar Fl. 1: 84 (1924)
= *Sesleriaheufleriana* [unranked] b *digitata* Schur, Verh. Zool.-Bot. Ges. Wien 6: 204 (1856) et Enum. Pl. Transsilv.: 744 (1866); BHL: https://www.biodiversitylibrary.org/item/137255#page/361; BHL: https://www.biodiversitylibrary.org/item/7364#page/764
= *Sesleriaheufleriana* [unranked] c *elongata* Schur, Verh. Zool. -Bot. Ges. Wien 6: 204 (1856), non Host; BHL: https://www.biodiversitylibrary.org/item/137255#page/361
= Sesleriaheuflerianavar.insignis Schur, Verh. Mitth. Siebenbürg. Vereins Naturwiss. Hermannstadt 4: 84 (1853); BHL: https://www.biodiversitylibrary.org/item/42660#page/970
= Sesleriaheuflerianaf.interrupta (Schur) Soó, Acta Bot. Acad. Sci. Hung. 17(1–2): 119 (1972); GBIF: https://www.gbif.org/species/6312632; IPNI: https://www.ipni.org/n/883029-1; WFO: http://www.worldfloraonline.org/taxon/wfo-0000898842; POWO: https://powo.science.kew.org/taxon/883029-1
= *Sesleriaheufleriana* [unranked] a polydactyla Schur, Verh. Zool.-Bot. Ges. Wien 6: 204 (1856); BHL: https://www.biodiversitylibrary.org/item/137255#page/361
= *Sesleriaheufleriana* [unranked] a praelonga Schur, Enum. Pl. Transsilv.: 744 (1866); GBIF: https://www.gbif.org/species/5948422; IPNI: https://www.ipni.org/n/77291902-1; WFO: http://www.worldfloraonline.org/taxon/wfo-0000898848; POWO: https://powo.science.kew.org/taxon/77291902-1; BHL: https://www.biodiversitylibrary.org/page/10544795#page/766
= Sesleriaheuflerianaf.praelonga (Schur) Gergely & Beldie, Fl. Rep. Soc. Rom. 12: 224 (1972); GBIF: https://www.gbif.org/species/6312633; GBIF: https://www.gbif.org/species/4119322; IPNI: https://www.ipni.org/n/883030-1; WFO: http://www.worldfloraonline.org/taxon/wfo-0000898843; POWO: https://powo.science.kew.org/taxon/883030-1
= Sesleriaheuflerianaf.prorepens (Schur) Soó, Acta Bot. Acad. Sci. Hung. 17(1–2): 119 (1972); GBIF: https://www.gbif.org/species/6312631; IPNI: https://www.ipni.org/n/883031-1; WFO: http://www.worldfloraonline.org/taxon/wfo-0000898844; POWO: https://powo.science.kew.org/taxon/883031-1
= *Seslerianitida* Heldr. ex Nyman, Consp. Fl. Eur. 4: 796 (1882) [nom. illeg.], non Ten.; GBIF: https://www.gbif.org/species/8701233; GBIF: https://www.gbif.org/species/7576396; IPNI: https://www.ipni.org/n/421373-1; WFO: http://www.worldfloraonline.org/taxon/wfo-0000898876; POWO: https://powo.science.kew.org/taxon/421373-1; BHL: https://www.biodiversitylibrary.org/page/11015940#page/807
= *Sesleriaprorepens* Schur ex Schur, Enum. Pl. Transsilv.: 743 (1866); BHL: https://www.biodiversitylibrary.org/page/10544795#page/765
= *Sesleriarobusta* Pávai, Oesterr. Bot. Z. 12: 214 (1862) [nom. nudum], non Schott et al.; GBIF: https://www.gbif.org/species/7612267; IPNI: https://www.ipni.org/n/421392-1; WFO: http://www.worldfloraonline.org/taxon/wfo-0000898904; POWO: https://powo.science.kew.org/taxon/421392-1; BHL: https://www.biodiversitylibrary.org/page/28679508#page/222
= *Sesleriatransilvanica* Schur, Verh. Zool.-Bot. Vereins Wien 6: 205 (1856) et Enum. Pl. Transsilv.: 745 (1866); GBIF: https://www.gbif.org/species/4118752; IPNI: https://www.ipni.org/n/421414-1; WFO: http://www.worldfloraonline.org/taxon/wfo-0000898933; POWO: https://powo.science.kew.org/taxon/421414-1; BHL: https://www.biodiversitylibrary.org/openurlmultiple.aspx?id=p16414640|p42929925|p11712973; BHL: https://www.biodiversitylibrary.org/item/7364#page/767
– *Sesleriacaerulea* Baumg., Enum. Stirp. Transsilv. 3: 228, Nr 2013 (1816) [p. p.], non (L.) Ard.

##### Conservation status

In Ukraine – LC (Onyshchenko et al. 2022).

##### Distribution

Pancarpathian subendemic.

##### Notes

Following POWO (https://powo.science.kew.org/taxon/421353-1, accessed on 05.06.2023), two subspecies of S.heuflerianaSchur are delimited –subsp.heufleriana(occurs in Slovakia, Hungary, Romania and Ukraine) andsubsp.hungarica (Ujhelyi) Deyl (occurs in Hungary and Slovakia). In the Ukrainian Carpathians, only S.heuflerianasubsp.heufleriana is present. Therefore, all reports of *S.heufleriana* from the Ukrainian Carpathians should be considered to belong to this subspecies.

In the Worldplants database (https://www.worldplants.de/world-plants-complete-list/complete-plant-list?name=Sesleria-sadleriana, accessed on 07.06.2023), *S.transilvanica* Schur is erroneously indicated amongst synonyms of *S.sadleriana* Janka. Janka (1882), on pages 309–310 and Janka (1884), on pages 28–29, pointed out that plants described as *S.sadleriana* differ from those occurring in Transylvania. Ascherson and Graebner (1898), on page 320, outlined peculiar Janka’s treatment of *S.heufleriana* and, at the same time, synonymised *S.sadleriana* Janka and *S.heufleriana* Janka non Schur under the name *S.budensis* (Borbás) Asch. & Graebn. Hence, *S.sadleriana* Janka and *S.heufleriana* Janka, non Schur are not synonyms of *S.transilvanica*.

#### Trisetum fuscum

(Kit. ex Schult.) Schult. in Roem. et Schult., Syst. Veg. 2: 664 (1817)

57BED123-3E9E-5DB4-A854-6962933FC4EA

https://www.catalogueoflife.org/data/taxon/592G5

https://www.gbif.org/species/4112815

urn:lsid:ipni.org:names:425205-1

http://www.worldfloraonline.org/taxon/wfo-0000905167

https://powo.science.kew.org/taxon/425205-1

https://species.wikimedia.org/wiki/Trisetum_fuscum

https://europlusmed.org/cdm_dataportal/taxon/2c4b09cd-03d0-47a5-8d13-9874fe36f8c2

https://www.worldplants.de/world-plants-complete-list/complete-plant-list/?name=Trisetum-fuscum

https://www.biodiversitylibrary.org/page/720353#page/672

≡ *Avenafusca* Kit. ex Schult., Oesterr. Fl. ed. 2, 1: 268 (1814), non Ard.; GBIF: https://www.gbif.org/species/7744598; GBIF: https://www.gbif.org/species/9231558; IPNI: https://www.ipni.org/n/391464-1; IPNI: https://www.ipni.org/n/77236898-1; WFO: http://www.worldfloraonline.org/taxon/wfo-0000851919; POWO: https://powo.science.kew.org/taxon/77236898-1; JSTOR Global Plants: https://plants.jstor.org/stable/10.5555/al.ap.specimen.m0210842
≡ Trisetumflavescenssubsp.fuscum (Kit. ex Schult.) Hack., Magyar Bot. Lap. 2: 111 (1903); GBIF: https://www.gbif.org/species/12096667; IPNI: https://www.ipni.org/n/77260262-1; WFO: http://www.worldfloraonline.org/taxon/wfo-1200013147; POWO: https://powo.science.kew.org/taxon/77260262-1; BHL: https://www.biodiversitylibrary.org/page/50241353#page/141
≡ *Trisetariafusca* (Kit. ex Schult.) Banfi & Soldano, Atti Soc. Ital. Sci. Nat. Mus. Civico Storia Nat. Milano 135(2): 383 (1996); GBIF: https://www.gbif.org/species/4114671; IPNI: https://www.ipni.org/n/77065416-1; WFO: http://www.worldfloraonline.org/taxon/wfo-0000904909; POWO: https://powo.science.kew.org/taxon/77065416-1
= *Avenaciliaris* Kit. ex Schult., Oesterr. Fl. ed. 2, 1: 268 (1814); GBIF: https://www.gbif.org/species/4154500; GBIF: https://www.gbif.org/species/7470202; IPNI: https://www.ipni.org/n/391393-1; WFO: http://www.worldfloraonline.org/taxon/wfo-0000851780; POWO: https://powo.science.kew.org/taxon/391393-1
= *Trisetumciliare* (Kit. ex Schult.) Domin, Preslia 13-15: 41 (1935) *; GBIF: https://www.gbif.org/species/4113396; IPNI: https://www.ipni.org/n/425167-1; WFO: http://www.worldfloraonline.org/taxon/wfo-0000905078; POWO: https://powo.science.kew.org/taxon/425167-1
= *Trisetumflavescens* [unranked] c *carpaticumf.majus* Zapał., Rozpr. Wydz. Mat.-Przyr. Akad. Umiejetn., Dzial B, Nauki Biol. 4: 108 (1904) et Consp. Fl. Galic. Crit. 1: 35 (1906), non Asch. & Graebn.; GBIF: https://www.gbif.org/species/12027006; POWO: https://powo.science.kew.org/taxon/528276-4
= *Trisetumtransylvanicum* Steud., Syn. Pl. Glumac. 1(3): 226 (1855), non Schur; GBIF: https://www.gbif.org/species/4109945; IPNI: https://www.ipni.org/n/425412-1; WFO: http://www.worldfloraonline.org/taxon/wfo-0000905488; POWO: https://powo.science.kew.org/taxon/425412-1; BHL: https://www.biodiversitylibrary.org/page/44974147#page/240
= Trisetumvariumvar.violaceum Schur, Oesterr. Bot. Z. 10: 75 (1860); GBIF: https://www.gbif.org/species/7770879; IPNI: https://www.ipni.org/n/77294779-1; WFO: http://www.worldfloraonline.org/taxon/wfo-1200021275; POWO: https://powo.science.kew.org/taxon/77294779-1; BHL: https://www.biodiversitylibrary.org/page/28597118#page/83
– *Trisetumtenue* Baumg. ex Steud., Syn. Pl. Glumac. 1(3): 226 (1854) [nom. illeg., pro syn. *T.transylvanicum* Steud.], non Leers; GBIF: https://www.gbif.org/species/8461326; GBIF: https://www.gbif.org/species/8061303; IPNI: https://www.ipni.org/n/425400-1; WFO: http://www.worldfloraonline.org/taxon/wfo-0000905475; POWO: https://powo.science.kew.org/taxon/425400-1; BHL: https://www.biodiversitylibrary.org/page/44974147#page/240
– *Avenacarpatica* auct. [e.g., Błocki ex herb.], non Host
– *Trisetariacarpatica* auct. fl. carpat., non (Host) Baumg
– *Trisetumcarpathicum* auct., non (Host) Roem. & Schult. *

##### Conservation status

In Ukraine – LC (Onyshchenko et al. 2022).

##### Distribution

Pancarpathian endemic.

##### Notes

Steudel (1855), on page 226, mentioned the name *T.tenue* twice, which may confuse. First, he mentioned it as an independent species in the sense of Roemer & Schultes. *Trisetumtenue* Roem. & Schult. (= *Avenatenuis* Moench) is currently recognised as a synonym for *Ventenatadubia* (Leers) Coss. & Durieu, which, following POWO (https://powo.science.kew.org/taxon/426312-1, accessed on 13.06.2023), has pan-European distribution, reaches the north of Africa and is widely introduced in North America. The second time, Steudel (1855) mentioned *T.tenue* in the sense of Baumgarten (concerning herbarium material) as a synonym for a newly-described species *T.transylvanicum* Steud. Barberá et al. (2018) conducted the revision of Trisetumsect.Trisetum and synonymised *T.transylvanicum* with *T.fuscum*. Therefore, *T.tenue* Baumg. ex Steud. should be considered a synonym for *T.fuscum*.

#### Magnoliopsida

46FE14B8-5B0D-521E-BBD1-FBADF9DA04DA

#### Apiales

B5A3EBC1-83E1-57EE-8758-342C1C068AE7

#### Apiaceae

F6F2BA45-8084-5BA2-B5C2-E477D48D6068

#### Heracleum carpaticum

Porcius, Magyar Növénytani Lapok 2: 25 (1878) et Fl. Naséud.: 144 (1881)

CE69450F-F757-50CA-8805-58FA8AD9D7D6

https://www.catalogueoflife.org/data/taxon/3KXBD

https://www.gbif.org/species/7358227

urn:lsid:ipni.org:names:77221836-1

http://www.worldfloraonline.org/taxon/wfo-0000745368

https://powo.science.kew.org/taxon/77221836-1

https://species.wikimedia.org/wiki/Heracleum_carpaticum

https://europlusmed.org/cdm_dataportal/taxon/616ef7e8-6c89-4d43-8f5b-b52b0bb164b0

https://www.worldplants.de/world-plants-complete-list/complete-plant-list/?name=Heracleum-carpaticum

≡ Heracleumsphondyliumsubsp.carpaticum (Porcius) Soó, Acta Bot. Acad. Sci. Hung. 23(3–4): 380 (1978); GBIF: https://www.gbif.org/species/3642778; IPNI: https://www.ipni.org/n/892101-1; WFO: http://www.worldfloraonline.org/taxon/wfo-0001365022; POWO: https://powo.science.kew.org/taxon/892101-1
= *Heracleumalpinum* Baumg., Enum. Stirp. Transsilv. 1: 215 (1816), non alior
= Heracleumcarpaticumf.alpinum (Baumg.) Borza, Consp. Fl. Rom. 2: 204 (1949)
= Heracleumcarpaticumf.palmatifidum Jáv., Magyar Bot. Lapok 9: 162 (1910); BHL: https://www.biodiversitylibrary.org/item/201903#page/582
= Heracleumcarpaticumf.porcii Pax, Grundz. Pfl. Karp. 2: 70 (1908)
= Heracleumcarpaticumf.typicum Nyár & Todor, Fl. Rep. Pop. Roman. 6: 625, 660 (1958)
– Heracleumcarpaticumvar.aconitifolium M.Pop. & Chrshan. [ex herb., nom. inval.], non Woronow
– *Heracleumpollinianum* Nyman, Consp. Fl. Eur. 2: 289 (1879) [p. p., tantum quod plantas ucrain. carpat.], non Bertol.; BHL: https://www.biodiversitylibrary.org/item/41446#page/300
– *Heracleumsimplicifolium* Herb. ex Nyman sensu Borza
– *Heracleumsimplicifolium* Herb., Fl. Bucov.: 302 (1859) et Herb. ex Nyman, Consp. Fl. Eur. 2: 289 (1879) [p. p., tantum quod plantas ucrain. carpat.]; GBIF: https://www.gbif.org/species/8444536; GBIF: https://www.gbif.org/species/7724113; IPNI: https://www.ipni.org/n/843167-1; WFO: http://www.worldfloraonline.org/taxon/wfo-0000720209; POWO: https://powo.science.kew.org/taxon/843167-1; BHL: https://www.biodiversitylibrary.org/item/41446#page/300

##### Conservation status

In Ukraine – NT (Onyshchenko et al. 2022).

##### Distribution

SE Carpathian endemic.

#### Heracleum sphondylium transsilvanicum

(Schur) Brummitt, Feddes Repert. 79: 65 (1968)

30BD9716-F16D-5131-A679-64005D915D7F

https://www.catalogueoflife.org/data/taxon/5HNL9

https://www.gbif.org/species/4928236

urn:lsid:ipni.org:names:77251611-1

http://www.worldfloraonline.org/taxon/wfo-0000745371

https://powo.science.kew.org/taxon/77251611-1

https://species.wikimedia.org/wiki/Heracleum_sphondylium_subsp._transsilvanicum

https://europlusmed.org/cdm_dataportal/taxon/e3a9655b-ed59-4a19-ab5f-0e8f6165eeb0

https://www.worldplants.de/world-plants-complete-list/complete-plant-list/?name=Heracleum-sphondylium

≡ Heracleumpalmatumsubsp.transsilvanicum (Schur) Nyman, Consp. Fl. Eur. 2: 289 (1879); GBIF: https://www.gbif.org/species/7497351; IPNI: https://www.ipni.org/n/77314722-1; WFO: http://www.worldfloraonline.org/taxon/wfo-0000745373; POWO: https://powo.science.kew.org/taxon/2898300-4; BHL: https://www.biodiversitylibrary.org/page/11015433#page/300
≡ Heracleumsphondyliumsubsp.transsilvanicum (Schur) Thellung, Oesterr. Bot. Z. 73: 211 (1924) [nom. invalid.]
≡ *Heracleumtranssilvanicum* Schur, Enum. Pl. Transsilv.: 267 (1866); GBIF: https://www.gbif.org/species/3642249; IPNI: https://www.ipni.org/n/843216-1; WFO: http://www.worldfloraonline.org/taxon/wfo-0000720253; POWO: https://powo.science.kew.org/taxon/843216-1; BHL: https://www.biodiversitylibrary.org/page/10544318#page/289
= Heracleumalpinumsubsp.palmatum (Baumg.) Briquet, Candollea 2: 16 (1924), non Crantz nec Rchb.; GBIF: https://www.gbif.org/species/8099962; IPNI: https://www.ipni.org/n/77314723-1; WFO: http://www.worldfloraonline.org/taxon/wfo-0000745374; POWO: https://powo.science.kew.org/taxon/2898301-4
= *Heracleumpalmatum* Baumg., Enum. Stirp. Transsilv. 1: 215 (1816), non Crantz nec Rchb. *; GBIF: https://www.gbif.org/species/3628887; IPNI: https://www.ipni.org/n/843117-1; WFO: http://www.worldfloraonline.org/taxon/wfo-0000720157; POWO: https://powo.science.kew.org/taxon/843117-1
= *Pastinacapalmata* (Baumg.) Calest., Webbia 1: 245 (1905); GBIF: https://www.gbif.org/species/5538686; IPNI: https://www.ipni.org/n/845748-1; WFO: http://www.worldfloraonline.org/taxon/wfo-0000391732; POWO: https://powo.science.kew.org/taxon/845748-1; BHL: https://www.biodiversitylibrary.org/page/51613911#page/265
– *Heracleumsimplicifolium* Herb., Fl. Bucov.: 302 (1859) et Herb. ex Nyman, Consp. Fl. Eur. 2: 289 (1879) [p. p., tantum quod plantas ucrain. carpat.]; GBIF: https://www.gbif.org/species/8444536; GBIF: https://www.gbif.org/species/7724113; IPNI: https://www.ipni.org/n/843167-1; WFO: http://www.worldfloraonline.org/taxon/wfo-0000720209; POWO: https://powo.science.kew.org/taxon/843167-1; BHL: https://www.biodiversitylibrary.org/item/41446#page/300

##### Conservation status

In Ukraine – NT (Onyshchenko et al. 2022).

##### Distribution

SE Carpathian endemic.

##### Notes

Following POWO (https://powo.science.kew.org/taxon/843179-1, accessed on 05.06.2023), *Heracleumsphondylium* L. comprises 15 subspecies in the World's flora. However, only three subspecies (i.e. *H.sphondylium* subsp. *sphondylium, H.sphondyliumsubsp.sibiricum* (L.) Simonk. and H.sphondyliumsubsp.transsilvanicum) occurs in Ukraine and, in particular, are present in the flora of the Ukrainian Carpathians. From these three subspecies, only H.sphondyliumsubsp.transsilvanicum is endemic and the other two subspecies have narrow distribution ranges.

#### Asterales

384C0FEF-F0B1-5110-99CA-025D1502529B

#### Asteraceae

6C89E956-B4E8-5780-A25C-A98925C22BCE

#### Achillea oxyloba schurii

(Sch.Bip.) Heimerl, Denkschr. Kaiserl. Akad. Wiss., Wien. Math.-Naturwiss. Kl. 48: 137 (1884)

7078D3F7-DEB3-5607-A849-6EC4C9C9E9CD

https://www.catalogueoflife.org/data/taxon/5FD98

https://www.gbif.org/species/4215221

urn:lsid:ipni.org:names:60439416-2

http://www.worldfloraonline.org/taxon/wfo-0000112464

https://powo.science.kew.org/taxon/60439416-2

https://species.wikimedia.org/wiki/Achillea_oxyloba_subsp._schurii

https://europlusmed.org/cdm_dataportal/taxon/c3b2ad2f-00dd-4d16-8000-e8509b369fe3

https://www.worldplants.de/world-plants-complete-list/complete-plant-list/?name=Achillea-oxyloba

https://www.biodiversitylibrary.org/page/7107159#page/225

https://je.jacq.org/JE00010937

https://je.jacq.org/JE00010938

https://je.jacq.org/JE00010939

≡ *Achilleaschurii* Sch.Bip., Oesterr. Bot. Wochenbl. 6: 300 (1856) *; GBIF: https://www.gbif.org/species/4214718; IPNI: https://www.ipni.org/n/174249-1; WFO: http://www.worldfloraonline.org/taxon/wfo-0000103154; POWO: https://powo.science.kew.org/taxon/174249-1; BHL: https://www.biodiversitylibrary.org/page/9838510#page/320
≡ *Anthemisschurii* Sch.Bip., Oesterr. Bot. Wochenbl. 6: 300 (1856) et Sch.Bip. ex Heimerl, Denkschr. Acad. Wien 48: 137 (1884) [nom. nudum]; GBIF: https://www.gbif.org/species/3120193; IPNI: https://www.ipni.org/n/177603-1; WFO: http://www.worldfloraonline.org/taxon/wfo-0000000716; POWO: https://powo.science.kew.org/taxon/177603-1; BHL: https://www.biodiversitylibrary.org/item/37783#page/320; BHL: https://www.biodiversitylibrary.org/item/31429#page/225
≡ *Ptarmicaschurii* Sch.Bip., Oesterr. Bot. Wochenbl. 6: 300 (1856); BHL: https://www.biodiversitylibrary.org/item/37783#page/320
≡ *Anthemistenuifolia* (Schur) Schur., Verh. Siebenb. Ver. Naturw. 2: 171 (1851) [nom. inval.], non *Achilleatenuifolia* Lam.; GBIF: https://www.gbif.org/species/4240009; IPNI: https://www.ipni.org/n/177647-1; WFO: http://www.worldfloraonline.org/taxon/wfo-0000091513; POWO: https://powo.science.kew.org/taxon/177647-1; BHL: https://www.biodiversitylibrary.org/item/42660#page/382
≡ *Ptarmicatenuifolia* (Schur) Schur, Enum. Pl. Transsilv.: 327 (1866), non *Achilleatenuifolia* Lam. *; GBIF: https://www.gbif.org/species/4215545; IPNI: https://www.ipni.org/n/240127-1; WFO: http://www.worldfloraonline.org/taxon/wfo-0000072630; POWO: https://powo.science.kew.org/taxon/240127-1; BHL: https://www.biodiversitylibrary.org/page/10544378#page/349
= *Achilleaatrata* Baumg., Enum. Stirp. Transsilv. 3: 141 (1816), non L.
= *Achilleadacica* Simonk., Termesz. Füzet. 10: 181 (1886) et Enum. Fl. Transsilv.: 317 (1886); GBIF: https://www.gbif.org/species/3120195; IPNI: https://www.ipni.org/n/173928-1; WFO: http://www.worldfloraonline.org/taxon/wfo-0000016766; POWO: https://powo.science.kew.org/taxon/173928-1; BHL: https://www.biodiversitylibrary.org/item/97426#page/203
= Achilleaschuriivar.dacica (Simonk.) Prodan & Nyár., Fl. Rep. Pop. Rom. 9: 369 (1964)
= Achilleaschuriif.pleiocephala Bommüller, Mitt. Thüringischen Bot. Vereins 30: 56 (1913); BHL: https://www.biodiversitylibrary.org/page/14324023#page/422
= Achilleaschuriivar.polycephala (Schur) Prodan & Nyár., Fl. Rep. Pop. Rom. 9: 369 (1964)
= *Anthemisalpina* Baumg., Enum. Stirp. Transsilv. 3: 145 (1816), non alior
= *Anthemiscaespitosa* Herbich, Flora 40: 509 (1857); GBIF: https://www.gbif.org/species/4242092; IPNI: https://www.ipni.org/n/177199-1; WFO: http://www.worldfloraonline.org/taxon/wfo-0000029120; POWO: https://powo.science.kew.org/taxon/177199-1; BHL: https://www.biodiversitylibrary.org/page/50749#page/510; JSTOR Global Plants: https://plants.jstor.org/stable/10.5555/al.ap.specimen.je00010937
= *Anthemisoxyloba* Schur, Enum. Pl. Transsilv.: 884 (1866), non *Achilleaoxyloba* (DC.) Sch.Bip.; GBIF: https://www.gbif.org/species/3120196; IPNI: https://www.ipni.org/n/177490-1; WFO: http://www.worldfloraonline.org/taxon/wfo-0000079117; POWO: https://powo.science.kew.org/taxon/177490-1; BHL: https://www.biodiversitylibrary.org/page/10544378#page/906
= *Anthemispseudo-atrata* Schur ex Schur, Enum. Pl. Transsilv.: 327 (1866); GBIF: https://www.gbif.org/species/4240513; IPNI: https://www.ipni.org/n/177530-1; WFO: http://www.worldfloraonline.org/taxon/wfo-0000124115; POWO: https://powo.science.kew.org/taxon/177530-1; BHL: https://www.biodiversitylibrary.org/page/10544378#page/349
= *Anthemistenuifolia* [unranked] a *simplex monocephala* Schur., Verh. Siebenb. Ver. Naturw. 4: 40 (1851); BHL: https://www.biodiversitylibrary.org/item/42660#page/926
= *Anthemistenuifolia* [unranked] b *ramosapolycephala* Schur., Verh. Siebenb. Ver. Naturw. 4: 40 (1851); BHL: https://www.biodiversitylibrary.org/item/42660#page/926
= *Anthemistenuifolia* [unranked] c *pilosa minima polaris* Schur., Verh. Siebenb. Ver. Naturw. 4: 40 (1851); BHL: https://www.biodiversitylibrary.org/item/42660#page/926
= *Ptarmicaoxyloba* Schur, Enum. Pl. Transsilv.: 326 (1866), non DC., non *Achilleaoxyloba* (DC.) Sch.Bip.; BHL: https://www.biodiversitylibrary.org/page/10544378#page/348/
= *Ptarmicapseudo-atrata* Schur ex Schur, Enum. Pl. Transsilv.: 327 (1866); GBIF: https://www.gbif.org/species/4215659; IPNI: https://www.ipni.org/n/240111-1; WFO: http://www.worldfloraonline.org/taxon/wfo-0000130464; POWO: https://powo.science.kew.org/taxon/240111-1; BHL: https://www.biodiversitylibrary.org/page/10544378#page/349
= *Ptarmicatenuifolia* [unranked] a macrocephala Schur, Enum. Pl. Transsilv.: 327 (1866), non alior; BHL: https://www.biodiversitylibrary.org/item/7364#page/347; JSTOR Global Plants: https://plants.jstor.org/stable/10.5555/al.ap.specimen.je00010939; JSTOR Global Plants: https://plants.jstor.org/stable/10.5555/al.ap.specimen.je00010938
= *Ptarmicatenuifolia* [unranked] b *polycephala* (Schur) Schur, Enum. Pl. Transsilv.: 327 (1866); BHL: https://www.biodiversitylibrary.org/item/7364#page/347

##### Conservation status

In Ukraine – LC (Onyshchenko et al. 2022).

##### Distribution

SE Carpathian endemic.

##### Notes

Sometimes A.oxylobasubsp.schurii is confused with *A.tenuifolia* Schur, which is a separate species. Despite the LC status given to it by Onyshchenko et al. (2022), *Ptarmicatenuifolia* (= A.oxylobasubsp.schurii) is considered a rare species by Zyman and Chorney (2009) and was recently approved for inclusion in the new edition of the Red Book of Ukraine (MEPNR of Ukraine 2021).

CoL (https://www.catalogueoflife.org/data/taxon/5FD98, accessed on 08.06.2023), GBIF (https://www.gbif.org/species/3120193, accessed on 08.06.2023), IPNI (https://www.ipni.org/n/177603-1, accessed on 08.06.2023) and other databases designate the authorship of *Anthemisschurii* to Anton Heimerl and indicate the place of publication – Denkschr. Acad. Wien 48: 137 (1884). However, Heimerl is not the author of this name. In his paper, Heimerl (1884), on page 137, indicates that the correct author of this name is Carl Schultz Bipontius and references Oesterr. Bot. Wochenbl. 6: 300 (1856). Schultz Bipontius (1856) applied even three names on the same page as an alternative – *Achilleaschurii, Anthemisschurii* and *Ptarmicaschurii*.

#### Antennaria carpatica carpatica

(Wahlenb.) Hook. in Bluff et Fingerh., Comp. Fl. German. 2: 348 (1825)

443A2A5B-FDB6-569A-BD7C-17E3B86485F8

https://www.catalogueoflife.org/data/taxon/5FN57

https://www.gbif.org/species/7222270

urn:lsid:ipni.org:names:77091531-1

http://www.worldfloraonline.org/taxon/wfo-0000005645

https://powo.science.kew.org/taxon/77091531-1

https://species.wikimedia.org/wiki/Antennaria_carpatica_subsp._carpatica

https://europlusmed.org/cdm_dataportal/taxon/d2ef5c0a-f220-4ebf-9676-b6d11e7feb9a

https://www.worldplants.de/world-plants-complete-list/complete-plant-list/?name=Antennaria-carpatica

https://www.biodiversitylibrary.org/page/6111900#page/370

https://plants.jstor.org/stable/10.5555/al.ap.specimen.goet001007

https://treatment.plazi.org/id/12F27F67-D6B0-A929-B3DC-162A02D4248C

Antennaria carpatica (Wahlenb.) Hook., Fl. Bor.-Amer. 1(suppl.): 329 (1834); GBIF: https://www.gbif.org/species/8243354; IPNI: https://www.ipni.org/n/1086760-2; WFO: http://www.worldfloraonline.org/taxon/wfo-0000003726; BHL: https://www.biodiversitylibrary.org/page/413406#page/340
≡ *Antennariacarpatica* (Wahlenb.) Hook. in Bluff & Fingerh., Comp. Fl. German. 2: 348 (1825); GBIF: https://www.gbif.org/species/7847414; IPNI: https://www.ipni.org/n/77091531-1; WFO: http://www.worldfloraonline.org/taxon/wfo-0000005645; POWO: https://powo.science.kew.org/taxon/77091531-1; BHL: https://www.biodiversitylibrary.org/page/6111900#page/370
≡ *Antennariacarpatica* (Wahlenb.) R.Br., Trans. Linn. Soc. London 12: 123 (1818) [nom. inval.] *; GBIF: https://www.gbif.org/species/8252547; IPNI: https://www.ipni.org/n/176874-1; WFO: http://www.worldfloraonline.org/taxon/wfo-0000084027; BHL: https://www.biodiversitylibrary.org/openurlmultiple.aspx?id=p758681|p12904620
≡ *Antennariacarpatica* (Wahlenb.) Trautv., Acta Horti Petropolitani 6(1): 24 (1879); BHL: https://www.biodiversitylibrary.org/item/53600#page/26
≡ *Chamaezelumcarpaticum* (Wahlenb.) Link, Handbuch Erkennung nutz. häufigsten vorkomm. Gewachse 1: 719 (1829); GBIF: https://www.gbif.org/species/3088158; IPNI: https://www.ipni.org/n/192559-1; WFO: http://www.worldfloraonline.org/taxon/wfo-0000034028; POWO: https://powo.science.kew.org/taxon/192559-1; BHL: https://www.biodiversitylibrary.org/page/53335348#page/729
≡ *Gnaphaliumcarpathicum* Wahlenb., Fl. Carpat. Princ.: 258, tab. 3, 260 (1814) et Fl. Suec., ed. 2, 2: 515 (1833); GBIF: https://www.gbif.org/species/5697227; IPNI: https://www.ipni.org/n/209230-1; IPNI: https://www.ipni.org/n/326707-2; IPNI: https://www.ipni.org/n/60445618-2; WFO: http://www.worldfloraonline.org/taxon/wfo-0000037729; POWO: https://powo.science.kew.org/taxon/60445618-2; POWO: https://powo.science.kew.org/taxon/326707-2; BHL: https://www.biodiversitylibrary.org/item/97776#page/93
= *Gnaphaliumwahlenbergii* Sieber ex Steud., Nomencl. Bot., ed. 2. 1: 696 (1841); GBIF: https://www.gbif.org/species/5385621; IPNI: https://www.ipni.org/n/210219-1; WFO: http://www.worldfloraonline.org/taxon/wfo-0000042044; POWO: https://powo.science.kew.org/taxon/210219-1; BHL: https://www.biodiversitylibrary.org/page/391920#page/699
– *Antennariaalpina* Ledeb., Fl. Ross. 2(2): 612 (1845–1846) [p. p., tantum quod plantas ucrain. carpat.], non (L.) Gaertn.; BHL: https://www.biodiversitylibrary.org/page/6104581#page/642
– *Antennariaalpina* auct fl. carpat. [e.g., Baumg.; Schur], non (L.) Gaertn.
– *Gnaphaliumalpinum* Willd., Sp. Pl., ed. 4 3(3): 1883 (1803), non L. [p.p., tantum quod plantas ucrain. carpat.]; GBIF: https://www.gbif.org/species/7859021; IPNI: https://www.ipni.org/n/209074-1; WFO: http://www.worldfloraonline.org/taxon/wfo-0000118765; POWO: https://powo.science.kew.org/taxon/209074-1; BHL: https://www.biodiversitylibrary.org/page/667272#page/409

##### Conservation status

In Ukraine – EN (Onyshchenko et al. 2022).

##### Distribution

Pancarpathian endemic.

##### Notes

Antennariacarpaticasubsp.carpatica is listed in the Red Book of Ukraine as a rare taxon (Zyman and Bulakh 2009). However, its threat status has recently been increased to an ‘obsolescent’ level (MEPNR of Ukraine 2021).

There are two commonly recognised subspecies of A.carpatica(Wahlenb.)Bluff & Fingerh. –subsp.carpaticaandsubsp.helvetica (Chrtek & Pouzar) Chrtek & Pouzar. Only A.carpaticasubsp.carpatica occurs in Carpathians, while A.carpaticasubsp.helvetica is present in the Alps. Chrtek and Pouzar (1985) also described A.carpaticasubsp.amphilanata Chrtek & Pouzar occurring in the Alps and Pyrenees, but it was later synonymised with A.carpaticasubsp.helvetica (Greuter 2006). It is worth noting that many collectors and authors out of Carpathians, under the name *A.carpatica*, considered it exactly as A.carpaticasubsp.helvetica.

Moreover, there is a close species, *A.lanata* Greene (= A.carpaticavar.lanata Hook., = A.carpaticavar.laestadiana Trautv., = *A.villifera* Boris.) that occurs not only in Eurasia, but also in North America (https://powo.science.kew.org/taxon/14721-2, accessed on 07.07.2023; Greene (1898)). Another close species, *A.lanatula* Chrtek & Pouzar, occurs exclusively in the south-west of North America (Chrtek and Pouzar 1985).

#### Centaurea maramarosiensis

(Jáv.) Czerep., Bot. Mater. Gerb. Bot. Inst. Komarova Akad. Nauk SSSR. 20: 395 (1960)

476AA9FC-529A-5B06-9610-A425A6DFD150

https://www.catalogueoflife.org/data/taxon/S6XN

https://www.gbif.org/species/4251406

https://www.gbif.org/species/3089524

urn:lsid:ipni.org:names:190962-1

http://www.worldfloraonline.org/taxon/wfo-0000136824

https://powo.science.kew.org/taxon/190962-1

https://species.wikimedia.org/wiki/Centaurea_maramarosiensis

https://europlusmed.org/cdm_dataportal/taxon/212de873-0e21-48a1-9fc3-2c6cc9c17f2f

https://www.worldplants.de/world-plants-complete-list/complete-plant-list/?name=Centaurea-maramarosiensis

≡ Centaureamollissubsp.marmarosiensis (Jáv.) Soó, Acta Bot. Acad. Sci. Hung. 13: 309 (1967); GBIF: https://www.gbif.org/species/6450873; GBIF: https://www.gbif.org/species/10972838; GBIF: https://www.gbif.org/species/6293888; WFO: http://www.worldfloraonline.org/taxon/wfo-0000102373
≡ Centaureamollisf.maramarosiensis Jáv., Magyar Fl. 3: 1170 (1925); GBIF: https://www.gbif.org/species/6076144; WFO: http://www.worldfloraonline.org/taxon/wfo-0001403923; POWO: https://powo.science.kew.org/taxon/50909566-1
≡ Centaureamontanasubsp.maramarosiensis (Jáv.) Soják, Čas. Nár. Mus., Odd. Přír. 140(3–4): 131 (1972); GBIF: https://www.gbif.org/species/9594652
≡ Centaureamontanasubsp.mollisvar.typicaf.maramarosiensis (Jáv.) Dostál, Acta Bot. Bohem. 10: 69 (1931)
≡ *Cyanusmaramarosiensis* (Jáv.) Dostál, Folia Mus. Rerum Nat. Bohemiae Occid., Bot. 21: 14 (1984); GBIF: https://www.gbif.org/species/9145130
≡ Cyanusmontanussubsp.maramarosiensis (Jáv.) Soják, Čas. Nár. Mus., Odd. Přír. 140 (3–4): 131 (1972); GBIF: https://www.gbif.org/species/5701665; IPNI: https://www.ipni.org/n/877128-1; WFO: http://www.worldfloraonline.org/taxon/wfo-0000059604; POWO: https://powo.science.kew.org/taxon/877128-1
≡ Cyanusmollissubsp.marmarosiensis (Jáv.) Soó [nom. et. des. invalid]; GBIF: https://www.gbif.org/species/9700012
= Centaureamollisf.ramosa Czakó in Jáv., Magyar Fl. 3: 1170 (1925), non *Centaurearamosa* (Gugler) Hayek
= Centaureamontanasubsp.mollisvar.ramosa (Czakó) Dostál, Acta Bot. Bohem. 10: 69 (1931), non *Centaurearamosa* (Gugler) Hayek

##### Conservation status

In Ukraine – LC (Onyshchenko et al. 2022).

##### Distribution

SE Carpathian endemic.

#### Centaurea phrygia carpatica

(Porcius) Dostál, Bot. J. Linn. Soc. 71(3): 207 (1976)

5C24FE14-D288-571D-BE6F-3CEEB92BF579

https://www.catalogueoflife.org/data/taxon/7JJJ4

https://www.gbif.org/species/4249913

urn:lsid:ipni.org:names:876940-1

http://www.worldfloraonline.org/taxon/wfo-0000109781

https://powo.science.kew.org/taxon/876940-1

https://europlusmed.org/cdm_dataportal/taxon/ac14c1f6-ac44-401e-b7ce-4517235d6760

https://www.worldplants.de/world-plants-complete-list/complete-plant-list/?name=Centaurea-phrygia

≡ *Centaureacarpatica* (Porcius) Porcius, Magyar Növényt. Lapok 9: 128 (1885) *; GBIF: https://www.gbif.org/species/8237713; IPNI: https://www.ipni.org/n/190150-1; WFO: http://www.worldfloraonline.org/taxon/wfo-0000007134; POWO: https://powo.science.kew.org/taxon/190150-1
≡ *Centaureacarpatica* (Porcius) Wagner, Cent. Hung.: 157 (1910)
≡ *Centaureacarpatica* (Porcius) Formánek, Oesterr. Bot. Z. 37: 153 (1887); GBIF: https://www.gbif.org/species/3097081; IPNI: https://www.ipni.org/n/190151-1; WFO: http://www.worldfloraonline.org/taxon/wfo-0000001725; BHL: https://www.biodiversitylibrary.org/page/28752056#page/159
≡ Centaureaplumosavar.carpatica Porcius, Enum. Pl. Phanerogam. Distr. Quondam Naszódiensis: 34 (1878) [nom. inval.]; GBIF: https://www.gbif.org/species/6076567; WFO: http://www.worldfloraonline.org/taxon/wfo-0000107278; POWO: https://powo.science.kew.org/taxon/50921977-1
≡ Centaureapseudophrygiaf.intercedenssubf.carpatica (Porcius) Gugler, Ann. Hist.-Nat. Musei Nat. Hungarici 6: 92 (1908); BHL: https://www.biodiversitylibrary.org/item/256063#page/104
≡ *Jaceacarpatica* (Porcius) Soják, Čas. Nár. Mus., Odd. Přír. 140(3–4): 132 (1972); GBIF: https://www.gbif.org/species/5695987; IPNI: https://www.ipni.org/n/226482-1; WFO: http://www.worldfloraonline.org/taxon/wfo-0000092877; POWO: https://powo.science.kew.org/taxon/226482-1
≡ Jaceaphrygiasubsp.carpatica (Porcius) Dostál, Folia Mus. Rer. Nat. Bohem. Occid., Bot. 21: 14 (1984); GBIF: https://www.gbif.org/species/5695845; IPNI: https://www.ipni.org/n/923452-1; WFO: http://www.worldfloraonline.org/taxon/wfo-0000108795; POWO: https://powo.science.kew.org/taxon/923452-1
= *Centaureaplumosa* β [unranked] *polycephala* Porcius, Enum. Pl. Phanerogam. Distr. Quondam Naszódiensis: 34 (1878)
= *Centaurearodnensis* Simonk., Enum. Fl. Transsilv.: 620 (1886) *; GBIF: https://www.gbif.org/species/7223238; WFO: http://www.worldfloraonline.org/taxon/wfo-0000117428; BHL: https://www.biodiversitylibrary.org/page/10524121#page/684
– Centaureamontanasubsp.mollis (Waldst. & Kit.) Gugler, Ann. Hist.-Nat. Mus. Natl. Hung. 6: 104 (1907) sensu Katina

##### Conservation status

In Ukraine – LC (Onyshchenko et al. 2022).

##### Distribution

SE Carpathian endemic.

##### Notes

Following POWO (https://powo.science.kew.org/taxon/191259-1, accessed on 05.06.2023), *Centaureaphrygia* L. includes 14 subspecies. From this number, only three subspecies (i.e. subsp. phrygia, subsp. carpatica and subsp. melanocalathia (Borbás ex Czakó) Dostál) occur in the Ukrainian Carpathians (Chopyk and Fedoronchuk 2015). Even though the last subspecies has a limited distribution and is considered as a Pancarpathian endemic (Tasenkevich 2003), Carpathian subendemic (Kricsfalusy and Budnikov 2002) or Carpatho-Balcanic taxon (Dostál 1989, Malynovskiy et al. 2002), it has a hybridogenous origin (Koutecký et al. 2012, Kliment et al. 2016) and, therefore, is not considered here. Hence, the only endemic representative in the Ukrainian Carpathians from the *C.phrygia* complex is C.phrygiasubsp.carpatica.

The Euro+Med PlantBase (Greuter 2006), amongst the homotypic synonyms of C.phrygiasubsp.carpatica, provides C.plumosavar.carpatica Porcius that is supposed to be published on p. 34 of “Enumeratio plantarum phanerogamicarum districtus quondam naszódiensis” (Porcius 1878). Similarly, this combination is also mentioned by Prodan and Nyárády (1964) on page 890 and Czerepanov (1994) on page 276. However, in this publication, there is no such combination published. Instead, Porcius (1878) on page 34, published a new combination *C.plumosa* β *polycephala* Porcius and only indicated *C.carpatica* as its synonym. I was also unable to detect where C.plumosavar.carpatica had been published by Porcius. Most probably, the combination C.plumosavar.carpatica Porcius arose mistakenly due to misinterpretation of this name by other authors and has never been published by Porcius.

#### Doronicum carpaticum

(Griseb. et Schenk) Nyman, Syll. Fl. Eur. suppl.: 1 (1865)

A6F9503D-B682-5873-861D-EDD1B69C04A1

https://www.catalogueoflife.org/data/taxon/37DTP

https://www.gbif.org/species/3142985

https://www.ipni.org/n/1016070-2

http://www.worldfloraonline.org/taxon/wfo-0000094993

https://powo.science.kew.org/taxon/1016070-2

https://species.wikimedia.org/wiki/Doronicum_carpaticum

https://europlusmed.org/cdm_dataportal/taxon/087b010f-cb20-410d-bab5-5a1211f34564

https://www.worldplants.de/world-plants-complete-list/complete-plant-list/?name=Doronicum-carpaticum

≡ *Aronicumcarpaticum* (Griseb. & Schenk) Schur, Bot. Rundr.: 71 (1853) et Verh. Siebenb. Ver. Naturw. 10: 137 (1859) *; GBIF: https://www.gbif.org/species/3142986; IPNI: https://www.ipni.org/n/1017626-2; POWO: https://powo.science.kew.org/taxon/1017626-2; BHL: https://www.biodiversitylibrary.org/item/42663#page/381
≡ *Aronicumcarpathicum* (Griseb. & Schenk) Schur, Bot. Rundr.: 71 (1853) et Verh. Siebenb. Ver. Naturw. 10: 137 (1859) [ortho. var.] *; GBIF: https://www.gbif.org/species/3142986; IPNI: https://www.ipni.org/n/1017626-2; POWO: https://powo.science.kew.org/taxon/1017626-2; BHL: https://www.biodiversitylibrary.org/item/42663#page/381
≡ *Aronicumcarpathicum* (Griseb. & Schenk) Fuss, Progr. Gymn. Hermannstadt: 12 (1854)
≡ Aronicumscorpioidesvar.carpaticum Griseb. & Schenk in Wiegm., Arch. Naturgesch. 18(1): 342 (1852); GBIF: https://www.gbif.org/species/4232202; IPNI: https://www.ipni.org/n/329148-2; WFO: http://www.worldfloraonline.org/taxon/wfo-0000027914; POWO: https://powo.science.kew.org/taxon/329148-2; BHL: https://www.biodiversitylibrary.org/page/13707751#page/350; JSTOR Global Plants: https://plants.jstor.org/stable/10.5555/al.ap.specimen.goet011169
≡ Doronicumcolumnaesubsp.carpaticum (Griseb. & Schenk) Sóo, Scripta Bot. Mus. Transsilv. 3(3–5): 10 (1944); GBIF: https://www.gbif.org/species/10954590; IPNI: https://www.ipni.org/n/60459784-2; WFO: http://www.worldfloraonline.org/taxon/wfo-0001360332; POWO: https://powo.science.kew.org/taxon/60459784-2
≡ Doronicumgrandiflorumsubsp.carpaticum (Griseb. & A. Schenk) Rouy, Rev. Bot. Syst. Geogr. Bot. 1: 53 (1903); GBIF: https://www.gbif.org/species/4231483; IPNI: https://www.ipni.org/n/1162227-2; WFO: http://www.worldfloraonline.org/taxon/wfo-4000012446; POWO: https://powo.science.kew.org/taxon/1162227-2; BHL: https://www.biodiversitylibrary.org/page/14597664#page/81
= *Aronicumbarcense* Simonk., Enum. Fl. Transsilv.: 322 (1886); GBIF: https://www.gbif.org/species/3142997; IPNI: https://www.ipni.org/n/179115-1; WFO: http://www.worldfloraonline.org/taxon/wfo-0000106373; POWO: https://powo.science.kew.org/taxon/179115-1; BHL: https://www.biodiversitylibrary.org/page/10524457#page/386
= *Aronicumcarpaticum* [unranked] a polyphyllum Schur, Enum. Pl. Transsilv.: 341 (1866); BHL: https://www.biodiversitylibrary.org/item/7364#page/361
= *Aronicumlatifolium* Schur, Verh. Mitth. Siebenbürg. Vereins Naturwiss. Hermannstadt 2: 171 (1851) [nom. nudum], non Rchb.; BHL: https://www.biodiversitylibrary.org/item/42660#page/382
= Doronicumcarpaticumvar.barcense (Simonk.) Borbás, Termr. Füz. 19: 219 (1896); BHL: https://www.biodiversitylibrary.org/item/96856#page/589
= Doronicumcordatumvar.asperum Borbás, Oesterr. Bot. Z. 28: 311 (1878); BHL: https://www.biodiversitylibrary.org/item/91412#page/327
= *Doronicumpardalianches* Heuff., [Enum. Pl. Banat. Temes.] Verh. K.K. Zool.-Bot. Ges. Wien 8: 137 (1858), non alior; BHL: https://www.biodiversitylibrary.org/item/137035#page/321
= *Doronicumorientale* Kotschy, Verh. Zool.-Bot. Vereins Wien 3: 140 (1853) [nom. nudum], non alior; BHL: https://www.biodiversitylibrary.org/item/86007#page/394
– *Arnicascorpioides* Baumg., Enum. Stirp. Transsilv. 3: 135 (1816), non alior

##### Conservation status

In Ukraine – LC (Onyshchenko et al. 2022).

##### Distribution

SE Carpathian endemic.

#### Leucanthemum rotundifolium

(Waldst. et Kit. in Willd.) DC., Prodr. 6: 46 (1838), non Opiz

59096653-FEF4-5E1D-9BEF-718B4952EFF8

https://www.catalogueoflife.org/data/taxon/6PRWY

https://www.gbif.org/species/5400956

urn:lsid:ipni.org:names:230081-1

http://www.worldfloraonline.org/taxon/wfo-0000137878

https://powo.science.kew.org/taxon/230081-1

https://species.wikimedia.org/wiki/Leucanthemum_rotundifolium

https://europlusmed.org/cdm_dataportal/taxon/f0573a53-5b02-442d-a43e-e60605a4ff11

https://www.worldplants.de/world-plants-complete-list/complete-plant-list/?name=Leucanthemum-rotundifolium

≡ *Leucanthemumrotundifolium* (Waldst. & Kit. in Willd.) Baumg., Enum. Stirp. Transsilv. 3: 107 (1817)
≡ *Leucanthemumrotundifolium* (Waldst. & Kit. in Willd.) Schur, Enum. Pl. Transsilv.: 339 (1866); GBIF: https://www.gbif.org/species/8333000; IPNI: https://www.ipni.org/n/230080-1; WFO: http://www.worldfloraonline.org/taxon/wfo-0000048186; POWO: https://powo.science.kew.org/taxon/230080-1; BHL: www.biodiversitylibrary.org/page/10544390#page/361
≡ *Chrysanthemumrotundifolium* Waldst. & Kit. in Willd., Sp. Pl. 3(3): 2144 (1803) *; BHL: https://www.biodiversitylibrary.org/item/14564#page/670
≡ *Chrysanthemumrotundifolium* Waldst. & Kit., Descr. Icon. Pl. Rar. Hung. 3: 262, t. 236 (1812); GBIF: https://www.gbif.org/species/3134081; IPNI: https://www.ipni.org/n/193725-1; WFO: http://www.worldfloraonline.org/taxon/wfo-0000096787; POWO: https://powo.science.kew.org/taxon/193725-1
≡ *Matricariarotundifolia* (Waldst. & Kit. in Willd.) Poir., Encycl. [J. Lamarck et al.] Suppl. 3.: 608 (1814); GBIF: https://www.gbif.org/species/4232595; IPNI: https://www.ipni.org/n/231991-1; WFO: http://www.worldfloraonline.org/taxon/wfo-0000035953; POWO: https://powo.science.kew.org/taxon/231991-1; BHL: https://www.biodiversitylibrary.org/page/738767#page/611
≡ *Tanacetumrotundifolium* (Waldst. & Kit. in Willd.) Simonk., Enum. Fl. Transsilv.: 313 (1886), non DC. *; GBIF: https://www.gbif.org/species/8145981; IPNI: https://www.ipni.org/n/252496-1; POWO: https://powo.science.kew.org/taxon/252496-1; BHL: https://www.biodiversitylibrary.org/page/10524448#page/377
= *Leucanthemumwaldsteinii* (Sch.Bip.) Pouzar, Preslia 47: 158 (1975); GBIF: https://www.gbif.org/species/5400957; IPNI: https://www.ipni.org/n/230104-1; WFO: http://www.worldfloraonline.org/taxon/wfo-0000061727; POWO: https://powo.science.kew.org/taxon/230104-1
= *Pyrethrumwaldsteinii* (Sch.Bip.) Janka, Bot. Jahresber. (Just) 4: 1062 (1878); GBIF: https://www.gbif.org/species/5692501; IPNI: https://www.ipni.org/n/241003-1; WFO: http://www.worldfloraonline.org/taxon/wfo-0000073828; POWO: https://powo.science.kew.org/taxon/241003-1; BHL: https://www.biodiversitylibrary.org/page/2232018#page/366
= *Tanacetumwaldsteinii* Sch.Bip., Tanaceteen: 35 (1844); GBIF: https://www.gbif.org/species/3134080; IPNI: https://www.ipni.org/n/252569-1; WFO: http://www.worldfloraonline.org/taxon/wfo-0000076302; POWO: https://powo.science.kew.org/taxon/252569-1
= Tanacetumwaldsteiniivar.ramosum Ilse &. Fritze, Verh. K.K. Zool.-Bot. Ges. Wien 20: 488 (1870); BHL: https://www.biodiversitylibrary.org/item/137000#page/626
– *Chrysanthemummontanum* Csató, Erd. Muz. [Az Erdélyi Múzeum-Egylet Évkönyveiben] 4: 82 (1868), non alior

##### Conservation status

In Ukraine – LC (Onyshchenko et al. 2022).

##### Distribution

Pancarpathian subendemic.

#### Saussurea porcii

Degen, Magyar Bot. Lapok 3: 311 (1904)

39D0BA90-B8EA-544F-84A1-4835FAAB0DAA

https://www.catalogueoflife.org/data/taxon/6Y3D4

https://www.gbif.org/species/5404478

urn:lsid:ipni.org:names:242550-1

http://www.worldfloraonline.org/taxon/wfo-0000009499

https://powo.science.kew.org/taxon/242550-1

https://europlusmed.org/cdm_dataportal/taxon/4f83275a-2213-4d2a-8b89-f016fc1c308d

https://www.worldplants.de/world-plants-complete-list/complete-plant-list/?name=Saussurea-porcii

https://www.biodiversitylibrary.org/page/50401658#page/333

http://herbarium.bgbm.org/object/B101113971

= *Saussureaalata* Porcius & Czetz, Transilvania 15–16: 118 (1881), non DC.
= *Saussureaserrata* Janka, Oesterr. Bot. Z. 8: 200 (1858), non DC.; BHL: https://www.biodiversitylibrary.org/item/91263#page/208
– *Saussureaparviflora* auct., non (Poir.) DC.
– *Saussureaserrata* auct. Transsilv., non DC.

##### Conservation status

In Ukraine – NT (Onyshchenko et al. 2022).

##### Distribution

SE Carpathian endemic.

##### Notes

This species is listed as rare in the last edition and has been recently approved for the new edition of the Red Book of Ukraine (Chorney and Danylyk 2009, MEPNR of Ukraine 2021). IPNI, POWO,and WFO (see links above; accessed on 05.06.2023) incorrectly indicate the page of the protologue – it should be 311 instead of 811.

#### Scorzoneroides pseudotaraxaci

(Schur) Holub, Folia Geobot. Phytotax. 12: 307 (1977)

2D14CB53-1C3F-5232-B1D2-ECD34376CA91

https://www.catalogueoflife.org/data/taxon/4VXKC

https://www.gbif.org/species/3133494

urn:lsid:ipni.org:names:243487-1

http://www.worldfloraonline.org/taxon/wfo-0000001257

https://powo.science.kew.org/taxon/243487-1

https://species.wikimedia.org/wiki/Scorzoneroides_pseudotaraxaci

https://europlusmed.org/cdm_dataportal/taxon/1a39d5e8-1034-4c66-aa01-25c0381eae52

https://www.worldplants.de/world-plants-complete-list/complete-plant-list/?name=Scorzoneroides-pseudotaraxaci

https://je.jacq.org/JE00017817

https://je.jacq.org/JE00017818

https://je.jacq.org/JE00017819

≡ *Leontodonpseudotaraxaci* Schur, Enum. Pl. Transsilv.: 357 (1866) *; GBIF: https://www.gbif.org/species/3133496; IPNI: https://www.ipni.org/n/229460-1; WFO: http://www.worldfloraonline.org/taxon/wfo-0000061097; POWO: https://powo.science.kew.org/taxon/229460-1; BHL: https://www.biodiversitylibrary.org/page/10544408#page/379
≡ Leontodonmontanussubsp.pseudotaraxaci (Schur) Finch & P.D.Sell, Bot. J. Linn. Soc. 71: 242 (1976); GBIF: https://www.gbif.org/species/4253414; IPNI: https://www.ipni.org/n/877573-1; WFO: http://www.worldfloraonline.org/taxon/wfo-0000110905; POWO: https://powo.science.kew.org/taxon/877573-1
≡ Scorzoneroidesmontana(Lam.)J.Holubsubsp.pseudotaraxaci [des. et nom. inval.]; GBIF: https://www.gbif.org/species/6082260
= *Leontodonclavatus* Sagorski & Schneider, Fl. Centralkarpath. 2: 254 (1890–1891) *; GBIF: https://www.gbif.org/species/3133495; IPNI: https://www.ipni.org/n/229281-1; WFO: http://www.worldfloraonline.org/taxon/wfo-0000123339; POWO: https://powo.science.kew.org/taxon/229281-1; JSTOR Global Plants: https://plants.jstor.org/stable/10.5555/al.ap.specimen.je00017818; JSTOR Global Plants: https://plants.jstor.org/stable/10.5555/al.ap.specimen.je00017819; JSTOR Global Plants: https://plants.jstor.org/stable/10.5555/al.ap.specimen.je00017817
= *Leontodonmedius* Simonk., Enum. Fl. Transsilv.: 352 (1886) et Bot. Centralbl. 49: 268 (1892), non *Apargiamedia* Host; GBIF: https://www.gbif.org/species/4253470; IPNI: https://www.ipni.org/n/229408-1; WFO: http://www.worldfloraonline.org/taxon/wfo-0000095956; POWO: https://powo.science.kew.org/taxon/229408-1; BHL: https://www.biodiversitylibrary.org/page/2119961#page/278; BHL: https://www.biodiversitylibrary.org/item/40105#page/416
= Leontodontaraxacivar.tatricus Kotula, Distr. Pl. Vasc. Mont. Tatr.: 356 (1890)
= *Leontodontatricis* (Kotula) Woł., Fl. Pol. Exs.: 545 (1897) [ortho. var.]
= *Leontodontatricus* (Kotula) Woł., Fl. Pol. Exs.: 545 (1897)
– *Apargiaaurea* Baumg., Enum. Stirp. Transsilv. 3: 16 (1816), non (L.) F.W.Schmidt, non *Leontodonaureum* L., nec *Ceraciumaureum* Schur.
– *Apargiataraxaci* Wahlenb., Fl. Carpat. Princ.: 235 (1814), non Willd.
– *Leontodontaraxaci* auct., non (L.) Loisel.
– *Leontodontaraxaci* R.Uechtr., Oesterr. Bot. Z. 14: 386 (1864) [nom. illeg] , non Loisel.; BHL: https://www.biodiversitylibrary.org/item/36417#page/378
– *Leontodonpyrenaeus* R.Uechtr., Oesterr. Bot. Wochenbl. 7: 370 (1857) [nom. illeg], non Gouan; BHL: https://www.biodiversitylibrary.org/item/94863#page/371
– *Leontodonpyrenaicus* Hoborski, Oesterr. Bot. Wochenbl. 3: 19 (1853) [nom. illeg], non Gouan; BHL: https://www.biodiversitylibrary.org/item/91226#page/29

##### Conservation status

In Ukraine – DD (Onyshchenko et al. 2022).

##### Distribution

Pancarpathian endemic.

##### Notes

All databases, except Euro+Med (https://europlusmed.org/cdm_dataportal/taxon/1a39d5e8-1034-4c66-aa01-25c0381eae52, accessed on 15.06.2023), indicate *Leontodonclavatus* Sagorsky & Schneider. as a synonym for *Scorzoneroideshispidula* (Delile) Greuter & Talavera. This confusion, perhaps, resulted from the fact that Sagorski and Schneider (1891), on page 254, supported the newly-described species with several controversial synonyms, i.e. *Apargiataraxaci*, *Leontodontaraxaci* and *Leontodonpyrenaicus*, which led to the further nomenclatural collapse of these names. Following POWO (https://powo.science.kew.org/taxon/77075521-1, accessed on 15.06.2023) and other databases, *Apargiataraxaci* Willd. and *Leontodontaraxaci* Loisel. are both synonyms for *Scorzoneroideshispidula*. In addition, following POWO (https://powo.science.kew.org/taxon/77180809-1, accessed on 15.06.2023), *Leontodonpyrenaicus* Gouan is a synonym for Scorzoneroidespyrenaica(Gouan)Holubsubsp.pyrenaica. Neither *Scorzoneroideshispidula* nor *S.pyrenaica* occurs in the Carpathians. At the same time, Sagorski and Schneider (1891) clearly indicated that their species is described from the Carpathians and even included specimens from the Ukrainian part of the Carpathians (i.e. Volovets town). Sagorski and Schneider (1891), later in the text, indicated differences between newly-described *L.clavatus* from *Apargiataraxaci* Willd., *Leontodontaraxaci* Loisel. and *Leontodonpyrenaicus* Gouan that occurs out of the Carpathians. They also pointed out that they consider *Apargiataraxaci*, *Leontodontaraxaci* and *Leontodonpyrenaicus* as synonyms for *L.clavatus* exclusively in the meaning of authors from the Carpathian region. In particular, they mentioned *Apargiataraxaci* Wahlenb., Fl. Carpat. Princ.: 235 (1814), non Willd.; *Leontodontaraxaci* R.Uechtr., Oesterr. Bot. Z. 14: 386 (1864), non Loisel.; *Leontodonpyrenaeus* R.Uechtr., Oesterr. Bot. Wochenbl. 7: 370 (1857); and *Leontodonpyrenaicus* Hoborski, Oesterr. Bot. Wochenbl. 3: 19 (1853), non Gouan. Therefore, *L.clavatus* is not a synonym, neither for *Scorzoneroideshispidula* nor for *S.pyrenaica*; it is a synonym for *S.pseudotaraxaci* as indicated by Euro+Med.

#### Senecio hercynicus ucranicus

(Hodálová) Greuter, Willdenowia 33: 247 (2003)

8AAC2E71-A8E4-55A2-880E-6221F407EA8A

https://www.catalogueoflife.org/data/taxon/5L5FB

https://www.gbif.org/species/4232604

urn:lsid:ipni.org:names:50426498-2

http://www.worldfloraonline.org/taxon/wfo-0000112942

https://powo.science.kew.org/taxon/50426498-2

https://europlusmed.org/cdm_dataportal/taxon/facab36a-04e8-4d3b-bfcc-65805e2cf833

https://www.worldplants.de/world-plants-complete-list/complete-plant-list/?name=Senecio-hercynicus

≡ *Senecioucranicus* Hodálová, Folia Geobot. 34 (3): 334 (1999), non Besser. *; GBIF: https://www.gbif.org/species/4215062; IPNI: https://www.ipni.org/n/1011450-1; WFO: http://www.worldfloraonline.org/taxon/wfo-0000135296; POWO: https://powo.science.kew.org/taxon/1011450-1

##### Conservation status

In Ukraine – DD (Onyshchenko et al. 2022).

##### Distribution

SE Carpathian endemic.

##### Notes

*Senecioucranicus* Hodálová has been described from the montane and subalpine belts. In the Ukrainian Carpathians, it is mentioned for Chyvchyny and Chornohora Mts. (Hodálová 1999). Unfortunately, I found no specimen of *S.ucranicus* (≡ S.hercynicussubsp.ucranicus) in the Ukrainian herbaria.

#### Campanulaceae

A17E83CD-E8FA-589A-8629-5114CE8553E5

#### Campanula carpatica

Jacq., Hort. Bot. Vindob. 1: 22, tab. 57 (1770), non C. carpatha Halácsy

ABD46F72-E0E0-53E4-ADA7-DFDF3FFA25BE

https://www.catalogueoflife.org/data/taxon/5X8SY

https://www.gbif.org/species/5410826

urn:lsid:ipni.org:names:140068-1

http://www.worldfloraonline.org/taxon/wfo-0000826758

https://powo.science.kew.org/taxon/140068-1

https://species.wikimedia.org/wiki/Campanula_carpatica

https://europlusmed.org/cdm_dataportal/taxon/6a581420-8d46-4fb2-986f-4bb346a00d02

https://www.worldplants.de/world-plants-complete-list/complete-plant-list/?name=Campanula-carpatica

https://www.biodiversitylibrary.org/page/307434#page/32

≡ *Campanulacordifolia* Vuk., Linnaea 26(3): 328 (1854), non K.Koch; GBIF: https://www.gbif.org/species/7407199; IPNI: https://www.ipni.org/n/140151-1; WFO: http://www.worldfloraonline.org/taxon/wfo-0000826972; POWO: https://powo.science.kew.org/taxon/140151-1; BHL: https://www.biodiversitylibrary.org/page/113484#page/330
≡ *Neocodoncarpaticus* (Jacq.) Kolak. & Serdyuk, Zametki Sist. Geogr. Rast. 40: 28 (1984); GBIF: https://www.gbif.org/species/3163033; IPNI: https://www.ipni.org/n/914284-1; WFO: http://www.worldfloraonline.org/taxon/wfo-0000816913; POWO: https://powo.science.kew.org/taxon/914284-1
= *Campanulacarpatica* Baumg. ex Schur, Enum. Pl. Transsilv.: 440 (1866) [nom. inval.]; BHL: https://www.biodiversitylibrary.org/item/7364#page/460
= *Campanulacarpatica* L. ex Schur, Enum. Pl. Transsilv.: 440 (1866) [nom. inval.]; BHL: https://www.biodiversitylibrary.org/item/7364#page/460
= *Campanulacarpatica* [unranked] *alba* (Voss) J.R.Duncan & V.C.Davies, Nursery Cat. (Duncan & Davies) 1925: 17 (1925)
= Campanulacarpaticavar.brachyphylla Morariu, Fl. Rep. Pop. Rom. 9: 76, 960 (1964)
= Campanulacarpaticavar.dasycarpa Schur, Enum. Pl. Transsilv.: 440 (1866); GBIF: https://www.gbif.org/species/5410828; WFO: http://www.worldfloraonline.org/taxon/wfo-0000826759; POWO: https://powo.science.kew.org/taxon/364196-4; BHL: https://www.biodiversitylibrary.org/item/7364#page/460
= Campanulacarpaticaf.dasycarpa (Schur) Tacik, Fl. Polska 12: 84 (1971); GBIF: https://www.gbif.org/species/7665609; IPNI: https://www.ipni.org/n/875483-1; WFO: http://www.worldfloraonline.org/taxon/wfo-0000826760; POWO: https://powo.science.kew.org/taxon/875483-1
= Campanulacarpaticavar.grandiflora Schur, Enum. Pl. Transsilv.: 440 (1866); GBIF: https://www.gbif.org/species/5410836; IPNI: https://www.ipni.org/n/77267465-1; WFO: http://www.worldfloraonline.org/taxon/wfo-0000826761; POWO: https://powo.science.kew.org/taxon/77267465-1; BHL: https://www.biodiversitylibrary.org/page/10544491#page/462
= Campanulacarpaticavar.hemisphaerica Schur, Enum. Pl. Transsilv.: 440 (1866); GBIF: https://www.gbif.org/species/5410841; IPNI: https://www.ipni.org/n/77267638-1; WFO: http://www.worldfloraonline.org/taxon/wfo-0000826762; POWO: https://powo.science.kew.org/taxon/77267638-1; BHL: https://www.biodiversitylibrary.org/page/10544491#page/462
= Campanulacarpaticavar.longifolia Morariu, Fl. Rep. Pop. Rom. 9: 79, 960 (1964)
= Campanulacarpaticavar.longifoliaf.parviflora Săvul. ex Morariu & Nyár., Fl. Rep. Pop. Rom. 9: 79, 960 (1964)
= Campanulacarpaticavar.oreophila Schur, Enum. Pl. Transsilv.: 440 (1866); GBIF: https://www.gbif.org/species/5410842; IPNI: https://www.ipni.org/n/77266926-1; WFO: http://www.worldfloraonline.org/taxon/wfo-0000826766; POWO: https://powo.science.kew.org/taxon/77266926-1; BHL: https://www.biodiversitylibrary.org/page/10544491#page/462
= *Campanulacarpatica* [unranked] *pelviformis* Froebel ex André, Rev. Hort. (Paris) 54: 509 (1882); GBIF: https://www.gbif.org/species/5410840; IPNI: https://www.ipni.org/n/77267279-1; POWO: https://powo.science.kew.org/taxon/77267279-1; BHL: https://www.biodiversitylibrary.org/page/49658684#page/559
= *Campanulacarpatica* [unranked] *riverslea* J.R.Duncan & V.C.Davies, Nursery Cat. (Duncan & Davies) 1925: 17 (1925)
= Campanulacarpaticavar.porrecta Morariu, Fl. Rep. Pop. Rom. 9: 76, 959 (1964)
= Campanulacarpaticavar.porrectaf.minor Morariu, Fl. Rep. Pop. Rom. 9: 76, 959 (1964)
= Campanulacarpaticavar.schuriana Săvul. ex Morariu & Nyár., Fl. Rep. Pop. Rom. 9: 76, 960 (1964)
= Campanulacarpaticavar.subdasycarpa Morariu & Nyár., Fl. Rep. Pop. Rom. 9: 79, 960 (1964)
= *Campanulacarpatica* [unranked] b *subpilosa* Schur, Enum. Pl. Transsilv.: 440 (1866); GBIF: https://www.gbif.org/species/5410838; WFO: https://list.worldfloraonline.org/wfo-0000826768; POWO: https://powo.science.kew.org/taxon/364203-4; BHL: https://www.biodiversitylibrary.org/item/7364#page/460
= Campanulacarpaticaf.subpilosa (Schur) Tacik, Fl. Polska 12: 84 (1971); GBIF: https://www.gbif.org/species/8353661; IPNI: https://www.ipni.org/n/875484-1; WFO: http://www.worldfloraonline.org/taxon/wfo-0000826769; POWO: https://powo.science.kew.org/taxon/875484-1
= Campanulacarpaticavar.tomentosa Kotschy, Verh. Zool.-Bot. Ges. Wien 3: 140 (1853); BHL: https://www.biodiversitylibrary.org/item/86007#page/394
= Campanulacarpaticavar.transsilvanica Schur, Bot. Rundreise [unpublished work]: 108 (1853) [nom. illeg.] et Verh. Mitth. Siebenbürg. Vereins Naturwiss. Hermannstadt 10: 174 (1859), non *Campanulatranssilvanica* Schur ex Andrae; BHL: https://www.biodiversitylibrary.org/item/42663
= Campanulacarpaticasubsp.turbinata (Schott, Nyman & Kotschy) Nyman, Consp. Fl. Eur. 482 (1879); GBIF: https://www.gbif.org/species/5410830; IPNI: https://www.ipni.org/n/77258441-1; WFO: http://www.worldfloraonline.org/taxon/wfo-0000826770; POWO: https://powo.science.kew.org/taxon/77258441-1; BHL: https://www.biodiversitylibrary.org/page/11015626#page/493
= Campanulacarpaticavar.turbinata (Schott, Nyman & Kotschy) Fuss, Fl. Transsilv. Exc.: 420 (1866)
= Campanulacarpaticavar.turbinata (Schott, Nyman & Kotschy) Nichols, Garden (London, 1871–1927) 45: 171 (1893); GBIF: https://www.gbif.org/species/11989529; GBIF: https://www.gbif.org/species/168085137; IPNI: https://www.ipni.org/n/77267254-1; WFO: http://www.worldfloraonline.org/taxon/wfo-0000826771; POWO: https://powo.science.kew.org/taxon/77267254-1; BHL: https://www.biodiversitylibrary.org/page/25626743#page/207
= Campanulacarpaticavar.turbinataf.rotundata Morariu, Fl. Rep. Pop. Rom. 9: 79, 960 (1964)
= *Campanuladasycarpa* Schur ex Schur, Enum. Pl. Transsilv.: 440 (1866) [nom. illeg.], non Kit. ex Schult.; GBIF: https://www.gbif.org/species/7907817; IPNI: https://www.ipni.org/n/140187-1; WFO: http://www.worldfloraonline.org/taxon/wfo-0000827018; POWO: https://powo.science.kew.org/taxon/140187-1; BHL: https://www.biodiversitylibrary.org/page/10544491#page/462
= *Campanuladasycarpa* Fuss. ex Schur, Enum. Pl. Transsilv.: 440 (1866), non Kit. ex Schult.; BHL: https://www.biodiversitylibrary.org/item/7364#page/460
= *Campanulaoreophila* Schur ex Schur, Enum. Pl. Transsilv.: 441 (1866); GBIF: https://www.gbif.org/species/5410835; IPNI: https://www.ipni.org/n/140769-1; WFO: http://www.worldfloraonline.org/taxon/wfo-0000828027; POWO: https://powo.science.kew.org/taxon/140769-1; BHL: https://www.biodiversitylibrary.org/page/10544492#page/463
= *Campanulapseudocarpatica* Schur, Enum. Pl. Transsilv.: 441 (1866); GBIF: https://www.gbif.org/species/5604956; IPNI: https://www.ipni.org/n/140887-1; WFO: http://www.worldfloraonline.org/taxon/wfo-0000828286; POWO: https://powo.science.kew.org/taxon/140887-1; BHL: https://www.biodiversitylibrary.org/page/10544492#page/463
= *Campanulareniformis* Schur, Enum. Pl. Transsilv.: 440 (1866); GBIF: https://www.gbif.org/species/5604927; IPNI: https://www.ipni.org/n/140968-1; WFO: http://www.worldfloraonline.org/taxon/wfo-0000828484; POWO: https://powo.science.kew.org/taxon/140968-1; BHL: https://www.biodiversitylibrary.org/page/10544492#page/463
= *Campanulaturbinata* Schott, Nyman & Kotschy, Analect. Bot.: 14 (1854); GBIF: https://www.gbif.org/species/5410839; IPNI: https://www.ipni.org/n/141258-1; WFO: http://www.worldfloraonline.org/taxon/wfo-0000829252; POWO: https://powo.science.kew.org/taxon/141258-1; BHL: https://www.biodiversitylibrary.org/page/10488333#page/26
= Campanulaturbinataf.alba Voss, Vilm. Blumengärtn. ed. 3, 1: 570 (1894); GBIF: https://www.gbif.org/species/5410827; WFO: http://www.worldfloraonline.org/taxon/wfo-0000829253; POWO: https://powo.science.kew.org/taxon/366618-4; BHL: https://www.biodiversitylibrary.org/item/134968#page/662
= Campanulaturbinataf.lilacina Voss, Vilm. Blumengärtn. ed. 3, 1: 570 (1894); GBIF: https://www.gbif.org/species/5410832; WFO: http://www.worldfloraonline.org/taxon/wfo-0000829254; POWO: https://powo.science.kew.org/taxon/366619-4; BHL: https://www.biodiversitylibrary.org/item/134968#page/662
= Campanulaturbinataf.pelviformis (Froebel ex André) Voss, Vilm. Blumengärtn. ed. 3, 1: 570 (1894); GBIF: https://www.gbif.org/species/5410833; GBIF: https://www.gbif.org/species/7407199; WFO: http://www.worldfloraonline.org/taxon/wfo-0000829256; POWO: https://powo.science.kew.org/taxon/366620-4; BHL: https://www.biodiversitylibrary.org/item/134968#page/662
– Campanulacarpaticavar.hendersonii (C.Wolley Dod) W.T.Mill., Cycl. Amer. Hort. 231 (1900) [hort.]; GBIF: https://www.gbif.org/species/5410837; IPNI: https://www.ipni.org/n/77267624-1; POWO: https://powo.science.kew.org/taxon/77267624-1
– *Campanulafergusonii* A.M.Ferguson, Rev. Hort. 76: 557 (1904) [pro hybr., hort]; GBIF: https://www.gbif.org/species/11265590; IPNI: https://www.ipni.org/n/140301-1; WFO: http://www.worldfloraonline.org/taxon/wfo-0000827170; POWO: https://powo.science.kew.org/taxon/140301-1; BHL: https://www.biodiversitylibrary.org/item/197157#page/607
– *Campanulahendersonii* C.Wolley Dod, Gard. Chron. n.s., 18: 502 (1882) [hort.]; GBIF: https://www.gbif.org/species/12053643; IPNI: https://www.ipni.org/n/140413-1; WFO: http://www.worldfloraonline.org/taxon/wfo-0000827394; POWO: https://powo.science.kew.org/taxon/140413-1; BHL: https://www.biodiversitylibrary.org/page/32986798#page/517
– *Campanula trans[s]ilvanica* Schur, Verh. Mitth. Siebenbürg. Vereins Naturwiss. Hermannstadt 10: 174 (1859), non *C.transsilvanica* Schur ex Andrae (1855) nec Schur (1866); BHL: https://www.biodiversitylibrary.org/item/42663#page/418

##### Conservation status

In Ukraine – NT (Onyshchenko et al. 2022).

##### Distribution

Pancarpathian endemic.

##### Notes

This species is listed in the last edition (Kagalo and Sytschak 2009a) and has been approved for the new edition (MEPNR of Ukraine 2021) of the Red Book of Ukraine with a status ‘rare’.

Schur (1859b) described *C.carpathica* var. *trans[s]ilvanica* and indicated among its synonyms *C. trans[s]ilvanica* Schur. He also noted "affinis valde *C.dasycarpae* Kit." [very similar to *C.dasycarpae* Kit.]. However, later Schur (1866) delimited *C.transilvanica* (p. 436) as independent species from *C.carpathica* [unranked] c *dasycarpa* (p. 440). Amongst the synonyms of *C.transilvanica*, Schur (1866) indicated *C.transsilvanica* Schur ex Andrae. Amongst the synonyms of *C.carpathica* [unranked] c *dasycarpa*, Schur (1866) provided *C.dasycarpae* Schur and *C.dasycarpa* Fuss and pointed out that he considers this species not in the sense of Pál Kitaibel. Besides this, amongst the synonyms of *C.carpathica* [unranked] c *dasycarpa*, Schur (1866) also provided C.carpathicavar.transsilvanica, but did not mention *C.transsilvanica* Schur in a rank of species anymore, as he did in 1859. Therefore, *C.transsilvanica* in sense of Schur (1859b) is not the same as *C.transsilvanica* in the sense of Schur ex Andrae (1855) and Schur (1866) and requires extra attention to avoid confusion.

#### Campanula kladniana

(Schur) Witasek, Abh. Zool.-Bot. Ges. Wien 1: 39 (1902)

0A530332-0496-5A59-9537-368FDA3A5A30

https://www.catalogueoflife.org/data/taxon/5X8SK

https://www.gbif.org/species/5411315

urn:lsid:ipni.org:names:140522-1

http://www.worldfloraonline.org/taxon/wfo-0000827551

https://powo.science.kew.org/taxon/140522-1

https://species.wikimedia.org/wiki/Campanula_kladniana

https://europlusmed.org/cdm_dataportal/taxon/2d7ebdb9-7c6e-4197-848c-924879558528

https://www.worldplants.de/world-plants-complete-list/complete-plant-list/?name=Campanula-carnica

≡ Campanulascheuchzerivar.kladniana Schur, Enum. Pl. Transsilv.: 443 (1866); GBIF: https://www.gbif.org/species/5411313; IPNI: https://www.ipni.org/n/77268441-1; WFO: http://www.worldfloraonline.org/taxon/wfo-0000828844; POWO: https://powo.science.kew.org/taxon/77268441-1; BHL: https://www.biodiversitylibrary.org/page/10544494#page/465; Plazi: https://treatment.plazi.org/id/DFCA53C1-A804-B864-9D6D-9BA3B82B505B
≡ Campanularotundifoliasubsp.kladniana (Schur) Tacik in Pawł. & Jasiewicz, Fl. Polska 12: 76 (1971); GBIF: https://www.gbif.org/species/5411317; IPNI: https://www.ipni.org/n/875512-1; WFO: http://www.worldfloraonline.org/taxon/wfo-0000828636; POWO: https://powo.science.kew.org/taxon/875512-1

##### Conservation status

In Ukraine – NT (Onyshchenko et al. 2022).

##### Distribution

SE Carpathian endemic.

##### Notes

*Campanulakladniana* is a rare alpine species listed by the Red Book of Ukraine (Zyman et al. 2009, MEPNR of Ukraine 2021).

#### Campanula serrata

(Kit. ex Schult.) Hendrych, Taxon 11: 123 (1962)

F3A73951-BB3B-5049-BEB6-0F222163250B

https://www.catalogueoflife.org/data/taxon/QBXQ

https://www.gbif.org/species/5411208

urn:lsid:ipni.org:names:141081-1

http://www.worldfloraonline.org/taxon/wfo-0000828909

https://powo.science.kew.org/taxon/141081-1

https://species.wikimedia.org/wiki/Campanula_serrata

https://europlusmed.org/cdm_dataportal/taxon/e349678d-60b7-4d07-a437-99a950e91334

https://www.worldplants.de/world-plants-complete-list/complete-plant-list/?name=Campanula-serrata

https://je.jacq.org/JE00007049

≡ *Thesiumserratum* Kit. ex Schult., Oesterr. Fl. ed. 2, 1: 437 (1814); GBIF: https://www.gbif.org/species/7614045; GBIF: https://www.gbif.org/species/7390879; IPNI: https://www.ipni.org/n/781116-1; POWO: https://powo.science.kew.org/taxon/781116-1
≡ *Thesiumserratum* Kit. ex D.Dietr., Syn. Pl. [D. Dietrich] 1: 878 (1839); GBIF: https://www.gbif.org/species/8101990; IPNI: https://www.ipni.org/n/781115-1; WFO: http://www.worldfloraonline.org/taxon/wfo-0001290288; BHL: https://www.biodiversitylibrary.org/page/678847#page/897
= *Campanulaarcuata* Schur, Verh. Mitth. Siebenbürg. Vereins Naturwiss. Hermannstadt 10: 138 (1859); GBIF: https://www.gbif.org/species/5411245; IPNI: https://www.ipni.org/n/139919-1; WFO: http://www.worldfloraonline.org/taxon/wfo-0000826522; POWO: https://powo.science.kew.org/taxon/139919-1; BHL: https://www.biodiversitylibrary.org/page/11528081#page/382
= *Campanulahornungiana* Schur, Enum. Pl. Transsilv.: 442 (1866); GBIF: https://www.gbif.org/species/5411233; IPNI: https://www.ipni.org/n/140450-1; WFO: http://www.worldfloraonline.org/taxon/wfo-0000827447; POWO: https://powo.science.kew.org/taxon/140450-1; BHL: https://www.biodiversitylibrary.org/page/10544493#page/464
= *Campanulakitaibeliana* Roem. & Schult., Syst. Veg., ed. 15 bis 5: 90 (1819); GBIF: https://www.gbif.org/species/5411255; IPNI: https://www.ipni.org/n/140521-1; WFO: http://www.worldfloraonline.org/taxon/wfo-0000827550; POWO: https://powo.science.kew.org/taxon/140521-1; BHL: https://www.biodiversitylibrary.org/page/714802#page/149
= *Campanulalanceolata* Neilr., Aufzählung Ungarn Slavonien Gefässpflanzen: 145 (1866), non alior; BHL: https://www.biodiversitylibrary.org/item/40087#page/293
= Campanulalanceolatasubsp.arcuata (Schur) Simonk., Enum. Fl. Transsilv.: 385 (1887); GBIF: https://www.gbif.org/species/5411228; IPNI: https://www.ipni.org/n/77258608-1; WFO: http://www.worldfloraonline.org/taxon/wfo-0000827597; POWO: https://powo.science.kew.org/taxon/77258608-1; BHL: https://www.biodiversitylibrary.org/page/10524520#page/449
= Campanulalanceolatavar.hornungiana (Schur) Simonk., Enum. Fl. Transsilv.: 385 (1887); GBIF: https://www.gbif.org/species/5411242; IPNI: https://www.ipni.org/n/77267534-1; WFO: http://www.worldfloraonline.org/taxon/wfo-0000827599; POWO: https://powo.science.kew.org/taxon/77267534-1; BHL: https://www.biodiversitylibrary.org/page/10524520#page/449
= *Campanulamicrophylla* Kit. ex Schult., Oesterr. Fl. ed. 2: 400 (1814), non Cav. [nom. illeg.]; GBIF: https://www.gbif.org/species/5411213; GBIF: https://www.gbif.org/species/7654241; IPNI: https://www.ipni.org/n/140679-1; WFO: http://www.worldfloraonline.org/taxon/wfo-0000827871; POWO: https://powo.science.kew.org/taxon/140679-1
= *Campanulanapuligera* Schur, Enum. Pl. Transsilv.: 444 (1866) *; GBIF: https://www.gbif.org/species/5411231; IPNI: https://www.ipni.org/n/140729-1; WFO: http://www.worldfloraonline.org/taxon/wfo-0000827943; POWO: https://powo.science.kew.org/taxon/140729-1; BHL: https://www.biodiversitylibrary.org/page/10544495#page/466
= Campanulanapuligeraf.albiflora Raclaru, Analele Univ. Bucureşti, Biol. Veg. 22: 125 (1973) [nom. nudum]; GBIF: https://www.gbif.org/species/5604644; IPNI: https://www.ipni.org/n/875497-1; WFO: http://www.worldfloraonline.org/taxon/wfo-0001325946
= Campanulanapuligeravar.alpiniformis Nyár. ex Morariu, Fl. Rep. Pop. Rom. 9: 96 (1964); GBIF: https://www.gbif.org/species/8554545; GBIF: https://www.gbif.org/species/5411220; IPNI: https://www.ipni.org/n/77267043-1; WFO: http://www.worldfloraonline.org/taxon/wfo-0000827944; POWO: https://powo.science.kew.org/taxon/77267043-1
= Campanulanapuligeraf.angustifrons Hruby, Magyar Bot. Lapok 29: 217 (1930); GBIF: https://www.gbif.org/species/5411211; WFO: http://www.worldfloraonline.org/taxon/wfo-0000827945; POWO: https://powo.science.kew.org/taxon/365333-4
= Campanulanapuligerasubf.angustifrons Hruby, Magyar Bot. Lapok 29: 217 (1930); POWO: https://powo.science.kew.org/taxon/365332-4
= Campanulanapuligeraf.arcuata (Schur) Hruby, Magyar Bot. Lapok 29: 217 (1930); GBIF: https://www.gbif.org/species/7645517; WFO: https://list.worldfloraonline.org/wfo-0000827946; POWO: https://powo.science.kew.org/taxon/365334-4
= *Campanulanapuligera*. var. arcuata (Schur) Morariu, Fl. Rep. Pop. Rom. 9: 93 (1964); GBIF: https://www.gbif.org/species/5411239; IPNI: https://www.ipni.org/n/77267658-1; WFO: http://www.worldfloraonline.org/taxon/wfo-0000827947; POWO: https://powo.science.kew.org/taxon/77267658-1
= Campanulanapuligerasubf.brachyantha Hruby, Magyar Bot. Lapok 29: 220 (1930); POWO: https://powo.science.kew.org/taxon/365336-4
= Campanulanapuligeravar.elatior (Săvul.) Morariu, Fl. Rep. Pop. Rom. 9: 94 (1964); GBIF: https://www.gbif.org/species/5411225; WFO: https://list.worldfloraonline.org/wfo-0000827948; POWO: https://powo.science.kew.org/taxon/365337-4
= Campanulanapuligeraf.genuina Hruby, Magyar Bot. Lapok 29: 220 (1930); GBIF: https://www.gbif.org/species/5411250; GBIF: https://www.gbif.org/species/12125287; POWO: https://powo.science.kew.org/taxon/365338-4; POWO: https://powo.science.kew.org/taxon/365339-4
= Campanulanapuligeraf.glabrescens Hruby, Magyar Bot. Lapok 29: 218 (1930); GBIF: https://www.gbif.org/species/5411229; GBIF: https://www.gbif.org/species/12159969; WFO: https://list.worldfloraonline.org/wfo-0000827950; POWO: https://powo.science.kew.org/taxon/365340-4; POWO: https://powo.science.kew.org/taxon/365341-4
= Campanulanapuligeravar.hirsuta Hruby, Magyar Bot. Lapok 29: 220 (1930); GBIF: https://www.gbif.org/species/5411217; IPNI: https://www.ipni.org/n/77267234-1; WFO: http://www.worldfloraonline.org/taxon/wfo-0000827951; POWO: https://powo.science.kew.org/taxon/77267234-1
= Campanulanapuligeravar.hornungiana (Schur) Morariu, Fl. Rep. Pop. Rom. 9: 97 (1964); GBIF: https://www.gbif.org/species/5411209; IPNI: https://www.ipni.org/n/77266939-1; WFO: http://www.worldfloraonline.org/taxon/wfo-0000827952; POWO: https://powo.science.kew.org/taxon/77266939-1
= Campanulanapuligeraf.humilis Hruby, Magyar Bot. Lapok 29: 218 (1930); GBIF: https://www.gbif.org/species/5411252; WFO: http://www.worldfloraonline.org/taxon/wfo-0000827953; POWO: https://powo.science.kew.org/taxon/365344-4
= Campanulanapuligeraf.intermedia Hruby, Magyar Bot. Lapok 29: 218 (1930); GBIF: https://www.gbif.org/species/5411243; WFO: http://www.worldfloraonline.org/taxon/wfo-0000827954; POWO: https://powo.science.kew.org/taxon/365345-4
= Campanulanapuligeraf.latifrons Hruby, Magyar Bot. Lapok, 29: 216 (1930); GBIF: https://www.gbif.org/species/5411221; WFO: http://www.worldfloraonline.org/taxon/wfo-0000827955; POWO: https://powo.science.kew.org/taxon/365347-4
= Campanulanapuligerasubf.latifrons Hruby, Magyar Bot. Lapok 29: 218 (1930); POWO: https://powo.science.kew.org/taxon/365346-4
= Campanulanapuligeraf.longisepala (Nyár.) Morariu, Fl. Rep. Pop. Rom. 9: 101 (1964); GBIF: https://www.gbif.org/species/8397712; WFO: http://www.worldfloraonline.org/taxon/wfo-0000827957; POWO: https://powo.science.kew.org/taxon/365349-4
= Campanulanapuligeravar.longisepala Nyár., Bul. Grad. Bot. Univ. Cluj 14: 95 (1934); GBIF: https://www.gbif.org/species/5411212; IPNI: https://www.ipni.org/n/77267556-1; WFO: http://www.worldfloraonline.org/taxon/wfo-0000827956; POWO: https://powo.science.kew.org/taxon/77267556-1
= Campanulanapuligeraf.minima (Săvul.) Morariu, Fl. Rep. Pop. Rom. 9: 97 (1964); GBIF: https://www.gbif.org/species/5411247; WFO: http://www.worldfloraonline.org/taxon/wfo-0000827959; POWO: https://powo.science.kew.org/taxon/365350-4
= Campanulanapuligeraf.parvula Morariu, Fl. Rep. Pop. Rom. 9: 96 (1964); GBIF: https://www.gbif.org/species/5411240; WFO: http://www.worldfloraonline.org/taxon/wfo-0000827960; POWO: https://powo.science.kew.org/taxon/365351-4
= Campanulanapuligeravar.redux (Schott, Nyman & Kotschy) Hruby, Magyar Bot. Lapok 29: 219 (1930) [nom. inval.]; GBIF: https://www.gbif.org/species/7690740; IPNI: https://www.ipni.org/n/77267368-1; WFO: http://www.worldfloraonline.org/taxon/wfo-0000827962; POWO: https://powo.science.kew.org/taxon/77267368-1
= Campanulanapuligeravar.redux (Schott, Nyman & Kotschy) Nyman, Consp. Fl. Eur.: 479 (1879); GBIF: https://www.gbif.org/species/5411226; IPNI: https://www.ipni.org/n/77267203-1; WFO: http://www.worldfloraonline.org/taxon/wfo-0000827961; POWO: https://powo.science.kew.org/taxon/77267203-1; BHL: https://www.biodiversitylibrary.org/page/11015623#page/490
= Campanulanapuligeraf.robusta Hruby, Magyar Bot. Lapok 29: 220 (1930); GBIF: https://www.gbif.org/species/5411216; WFO: http://www.worldfloraonline.org/taxon/wfo-0000827963; POWO: https://powo.science.kew.org/taxon/365354-4
= Campanulanapuligeraf.savulescui Morariu, Fl. Rep. Pop. Rom. 9: 97 (1964); GBIF: https://www.gbif.org/species/5411251; WFO: http://www.worldfloraonline.org/taxon/wfo-0000827964; POWO: https://powo.science.kew.org/taxon/365355-4
= Campanulanapuligeravar.savulescui Morariu, Fl. Rep. Pop. Rom. 9: 96 (1964); GBIF: https://www.gbif.org/species/7891049; IPNI: https://www.ipni.org/n/77267146-1; WFO: http://www.worldfloraonline.org/taxon/wfo-0000827965; POWO: https://powo.science.kew.org/taxon/77267146-1
= Campanulanapuligeraf.scheuzeriformis (Nyár.) Morariu, Fl. Rep. Pop. Rom. 9: 98. (1964); GBIF: https://www.gbif.org/species/5411230; WFO: http://www.worldfloraonline.org/taxon/wfo-0000827967; POWO: https://powo.science.kew.org/taxon/365358-4
= Campanulanapuligeravar.scheuzeriformis Nyár., Bul. Grad. Bot. Univ. Cluj 14: 95 (1934); GBIF: https://www.gbif.org/species/7733859; IPNI: https://www.ipni.org/n/77267493-1; WFO: http://www.worldfloraonline.org/taxon/wfo-0000827966; POWO: https://powo.science.kew.org/taxon/77267493-1
= Campanulanapuligeraf.semiamplexicaulis (Vladescu & Săvul.) Morariu, Fl. Rep. Pop. Rom. 9: 98 (1964); GBIF: https://www.gbif.org/species/5411218; WFO: http://www.worldfloraonline.org/taxon/wfo-0000827968; POWO: https://powo.science.kew.org/taxon/365359-4
= Campanulanapuligeraf.setulosa Morariu, Fl. Rep. Pop. Rom. 9: 97 (1964); GBIF: https://www.gbif.org/species/5411254; WFO: http://www.worldfloraonline.org/taxon/wfo-0000827970; POWO: https://powo.science.kew.org/taxon/365360-4
= Campanulanapuligeraf.simplex Hruby, Magyar Bot. Lapok 29: 216 (1930); GBIF: https://www.gbif.org/species/5411244; WFO: http://www.worldfloraonline.org/taxon/wfo-0000827971; POWO: https://powo.science.kew.org/taxon/365361-4
= Campanulanapuligeraf.stenophylloides Nyár., Bul. Grad. Bot. Univ. Cluj 14: 96 (1934); GBIF: https://www.gbif.org/species/5411232; WFO: http://www.worldfloraonline.org/taxon/wfo-0000827972; POWO: https://powo.science.kew.org/taxon/365362-4
= Campanulanapuligeravar.stenophylloides (Nyár.) Morariu, Fl. Rep. Pop. Rom. 9: 98 (1964); GBIF: https://www.gbif.org/species/7996662; WFO: http://www.worldfloraonline.org/taxon/wfo-0000827973; POWO: https://powo.science.kew.org/taxon/365363-4
= Campanulanapuligeravar.stricta Hruby, Magyar Bot. Lapok 29: 216 (1930); GBIF: https://www.gbif.org/species/5411222; IPNI: https://www.ipni.org/n/77267597-1; WFO: http://www.worldfloraonline.org/taxon/wfo-0000827974; POWO: https://powo.science.kew.org/taxon/77267597-1
= Campanulanapuligerasubf.tenella Hruby, Magyar Bot. Lapok 29: 220 (1930); POWO: https://powo.science.kew.org/taxon/365365-4
= Campanulanapuligeravar.transsilvanica (Săvul.) Morariu, Fl. Rep. Pop. Rom. 9: 93 (1964); GBIF: https://www.gbif.org/species/5411241; WFO: http://www.worldfloraonline.org/taxon/wfo-0000827975; POWO: https://powo.science.kew.org/taxon/365366-4
= Campanulanapuligeravar.umbrosa Hruby, Magyar Bot. Lapok 29: 218 (1930); GBIF: https://www.gbif.org/species/5411236; IPNI: https://www.ipni.org/n/77267720-1; WFO: http://www.worldfloraonline.org/taxon/wfo-0000827976; POWO: https://powo.science.kew.org/taxon/77267720-1
= Campanulapolymorphaf.pseudolanceolata (Pant.) Hruby, Magyar Bot. Lapok 29: 203 (1930); GBIF: https://www.gbif.org/species/5411248; WFO: http://www.worldfloraonline.org/taxon/wfo-0000828232; POWO: https://powo.science.kew.org/taxon/365612-4
= *Campanulapseudolanceolata* Pant., Magyar Növényt. Lapok 6: 162 (1882) et Pant. ex A.Kern., Sched. Fl. Exs. Austro-Hung. [Kerner] 9: 37 (1902) *; GBIF: https://www.gbif.org/species/5411219; IPNI https://www.ipni.org/n/140889-1; WFO: http://www.worldfloraonline.org/taxon/wfo-0000828274; POWO: https://powo.science.kew.org/taxon/140889-1; JSTOR Global Plants: https://plants.jstor.org/stable/10.5555/al.ap.specimen.kfta0001704; JSTOR Global Plants: https://plants.jstor.org/stable/10.5555/al.ap.specimen.je00007049; JSTOR Global Plants: https://plants.jstor.org/stable/10.5555/al.ap.specimen.kfta0001705
= Campanulapseudolanceolataf.albiflora Săvul., Stud. Sp. Campanula: 81 (1916); GBIF: https://www.gbif.org/species/5411210; WFO: http://www.worldfloraonline.org/taxon/wfo-0000828275; POWO: https://powo.science.kew.org/taxon/365654-4
= Campanulapseudolanceolatavar.arcuata (Schur) Porcius, Analele Acad. Romane ser. 2, 14: 196 (1893); GBIF: https://www.gbif.org/species/5411256; IPNI: https://www.ipni.org/n/77268680-1; WFO: http://www.worldfloraonline.org/taxon/wfo-0000828276; POWO: https://powo.science.kew.org/taxon/77268680-1
= Campanulapseudolanceolataf.elatior Săvul., Stud. Sp. Campanula: 78 (1916); GBIF: https://www.gbif.org/species/5411246; WFO: http://www.worldfloraonline.org/taxon/wfo-0000828277; POWO: https://powo.science.kew.org/taxon/365656-4
= Campanulapseudolanceolatavar.hornungiana (Schur) Porcius, Anal. Acad. Rom., Ser. 2 14: 202 (1893); GBIF: https://www.gbif.org/species/5411238; IPNI: https://www.ipni.org/n/77268124-1; WFO: http://www.worldfloraonline.org/taxon/wfo-0000828278; POWO: https://powo.science.kew.org/taxon/77268124-1
= Campanulapseudolanceolataf.minima Săvul., Stud. Sp. Campanula: 81 (1916); GBIF: https://www.gbif.org/species/5411234; WFO: http://www.worldfloraonline.org/taxon/wfo-0000828279; POWO: http://www.worldfloraonline.org/taxon/wfo-0000828279
= Campanulapseudolanceolatavar.porcii Săvul., Stud. Sp. Campanula: 84 (1916); GBIF: https://www.gbif.org/species/5411224; IPNI: https://www.ipni.org/n/77268764-1; WFO: http://www.worldfloraonline.org/taxon/wfo-0000828280; POWO: https://powo.science.kew.org/taxon/77268764-1
= Campanulapseudolanceolatasubsp.semiamplexicaulis Vladescu & Săvul., Stud. Sp. Campanula: 86 (1916); GBIF: https://www.gbif.org/species/5411257; IPNI: https://www.ipni.org/n/77258837-1; WFO: http://www.worldfloraonline.org/taxon/wfo-0000828282; POWO: https://powo.science.kew.org/taxon/77258837-1
= Campanulapseudolanceolataf.transsilvanica Săvul., Stud. Sp. Campanula: 78 (1916); GBIF: https://www.gbif.org/species/5411249; WFO: http://www.worldfloraonline.org/taxon/wfo-0000828283; POWO: https://powo.science.kew.org/taxon/365661-4
= Campanulapseudolanceolataf.umbraticola Săvul., Stud. Sp. Campanula: 78 (1916); GBIF: https://www.gbif.org/species/5411237; WFO: http://www.worldfloraonline.org/taxon/wfo-0000828284; POWO: https://powo.science.kew.org/taxon/365662-4
= *Campanularedux* Schott, Nyman & Kotschy, Analect. Bot.: 9 (1854); GBIF: https://www.gbif.org/species/5411223; IPNI: https://www.ipni.org/n/140961-1; WFO: http://www.worldfloraonline.org/taxon/wfo-0000828474; POWO: https://powo.science.kew.org/taxon/140961-1; BHL: https://www.biodiversitylibrary.org/page/10488366#page/21
= Campanularhomboidalisvar.angustifolia Neilr., Aufz. Ungarn Slavon. Gefässpfl.: 145 (1866); GBIF: https://www.gbif.org/species/5411227; IPNI: https://www.ipni.org/n/77268806-1; WFO: http://www.worldfloraonline.org/taxon/wfo-0000828500; POWO: https://powo.science.kew.org/taxon/77268806-1; BHL: https://www.biodiversitylibrary.org/item/40087#page/293
= Campanularhomboidalissubsp.pseudolanceolata (Pant.) Nyman, Consp. Fl. Eur. Suppl. 2: 208 (1889); GBIF: https://www.gbif.org/species/5411215; IPNI: https://www.ipni.org/n/77258595-1; WFO: http://www.worldfloraonline.org/taxon/wfo-0000828514; POWO: https://powo.science.kew.org/taxon/77258595-1; BHL: https://www.biodiversitylibrary.org/page/11015350#page/217
= *Campanularhomboidea* [unranked] β *foliis ovato-oblongis* Wahlenberg, Fl. Carpat.: 60 (1814), non L.
= Campanularotundifoliavar.alpina Schur, Enum. Pl. Transsilv.: 444 (1866) [nom. illeg.], non Tuck.; GBIF: https://www.gbif.org/species/8234052; POWO: https://powo.science.kew.org/taxon/365931-4; BHL: https://www.biodiversitylibrary.org/item/7364#page/464
= Campanularotundifoliavar.arcuata (Schur) Nyman, Consp. Fl. Eur.: 479 (1879); GBIF: https://www.gbif.org/species/5411253; IPNI: https://www.ipni.org/n/77267922-1; WFO: http://www.worldfloraonline.org/taxon/wfo-0000828565; POWO: https://powo.science.kew.org/taxon/77267922-1; BHL: https://www.biodiversitylibrary.org/page/11015623#page/490
= Campanularotundifoliavar.dentata Schur, Enum. Pl. Transsilv.: 444 (1866), non N.Coleman; GBIF: https://www.gbif.org/species/5410676; POWO: https://powo.science.kew.org/taxon/365967-4; BHL: https://www.biodiversitylibrary.org/item/7364#page/464
= Campanularotundifoliavar.grandiflora J.A.Knapp, Pfl. Galiz.: 173 (1872) [nom. illeg.], non alior; GBIF: https://www.gbif.org/species/7764236; IPNI: https://www.ipni.org/n/77268584-1; POWO: https://powo.science.kew.org/taxon/77268584-1
– Campanulaserratavar.elatior (Săvul.) Tasenkevych [nom. provis. et inval., ex herb. LWS]
– Campanulaserratavar.elatiorf.latifrons Hruby [comb. inval. ex herb. CHER] *
– Campanulaserratavar.hornungiana (Schur.) Tasenkevych [nom. provis. et inval., ex herb. LWS]
– *Campanulalancifolia* Schur, Enum. Pl. Transsilv.: 445 (1866) [nom. illeg.] sensu Błocki, non Witasek

##### Conservation status

In Ukraine – LC (Onyshchenko et al. 2022). GLobal – LC (Bilz 2011a).

##### Distribution

Pancarpathian endemic.

##### Notes

This morphologically variable complex basically includes three hardly distinguishable species – *C.serrata*, *C.napuligera* and *C.pseudolanceolata*. CoL (https://www.catalogueoflife.org/data/taxon/QBXQ, accessed on 07.06.2023), GBIF (https://www.gbif.org/species/5411208, accessed on 07.06.2023), POWO (https://powo.science.kew.org/taxon/141081-1, accessed on 05.06.2023), WFO (https://list.worldfloraonline.org/wfo-0000828909, accessed on 05.06.2023) and Worldplants (https://www.worldplants.de/world-plants-complete-list/complete-plant-list?name=Campanula-serrata, accessed on 07.06.2023) also include *C.arcuata, C.hornungiana, C.kitaibeliana, C.microphylla, C.redux* and some infraspecific taxa from *C.rotundifolia* to this complex, making it one of the most saturated by synonyms. POWO (https://powo.science.kew.org/taxon/140550-1, accessed on 05.06.2023), WFO (https://list.worldfloraonline.org/wfo-0000828550, accessed on 05.06.2023) and Worldplants (https://www.worldplants.de/world-plants-complete-list/complete-plant-list?name=Campanula-rotundifolia, accessed on 07.06.2023) consider *C.lancifolia* Schur, non Witasek as a synonym of *C.rotundifolia* L. subsp. rotundifolia. However, Błocki in the original label on the specimen LWS 92206, probably mistakenly, indicated *C.lancifolia* Schur as a synonym of *C.pseudolanceolata* Pant.

Campanulaserratasubsp.recta (Dulac) Podlech (≡ *C.recta* Dulac) is a confusing name applied for Pyreneinian plants that do not belong to *C.serrata* s.str., but rather to C.scheuchzerisubsp.lanceolata (Lapeyr.) J.-M.Tison.

#### Campanula tatrae tatrae

Borbás, Magyar Bot. Lapok 1: 319 (1902)

C42DC870-8DFC-5C55-B9DE-B85FD329FB85

https://www.catalogueoflife.org/data/taxon/7JFNM

https://www.gbif.org/species/7222073

urn:lsid:ipni.org:names:141196-1

http://www.worldfloraonline.org/taxon/wfo-0000829119

https://powo.science.kew.org/taxon/77168970-1

https://species.wikimedia.org/wiki/Campanula_tatrae

https://europlusmed.org/cdm_dataportal/taxon/c454d5af-c66c-4d0a-b009-a0e8754e9c9c

https://www.worldplants.de/world-plants-complete-list/complete-plant-list/?name=Campanula-tatrae

https://www.biodiversitylibrary.org/page/50326303#page/345

= Campanulakladnianasubsp.polymorpha Witasek, Magyar Bot. Lapok 5: 239 (1906); GBIF: https://www.gbif.org/species/5410038; IPNI: https://www.ipni.org/n/77255487-1; WFO: http://www.worldfloraonline.org/taxon/wfo-0000827554; POWO: https://powo.science.kew.org/taxon/77255487-1; BHL: https://www.biodiversitylibrary.org/page/50344447#page/645; JACQ: https://wu.jacq.org/WU0071526
= Campanulakladnianavar.polymorpha (Witasek) Pawł., Acta Soc. Bot. Pol. 1: 5 (1923); GBIF: https://www.gbif.org/species/8038018; WFO: http://www.worldfloraonline.org/taxon/wfo-0000827555; POWO: https://powo.science.kew.org/taxon/364966-4
= Campanulakladnianasubsp.stenophylla (Schur) Witasek, Magyar Bot. Lapok 5: 238 (1906); GBIF: https://www.gbif.org/species/5410040; IPNI: https://www.ipni.org/n/77256020-1; WFO: http://www.worldfloraonline.org/taxon/wfo-0000827556; POWO: https://powo.science.kew.org/taxon/77256020-1; BHL: https://www.biodiversitylibrary.org/page/50344446#page/644
= *Campanulapolymorpha* (Witasek) Prain, Index Kew. Suppl. 4: 35 (1913), non Banks & Sol. ex A.DC. *; GBIF: https://www.gbif.org/species/5410057; IPNI: https://www.ipni.org/n/77237988-1; WFO: http://www.worldfloraonline.org/taxon/wfo-0000828219; POWO: https://powo.science.kew.org/taxon/77237988-1; BHL: https://www.biodiversitylibrary.org/item/133932#page/47
= Campanulapolymorphavar.intercedens Hruby, Magyar Bot. Lapok 29: 199 (1930), non C.witasekianavar.intercedens Hruby; GBIF: https://www.gbif.org/species/5410061; IPNI: https://www.ipni.org/n/77266950-1; WFO: http://www.worldfloraonline.org/taxon/wfo-0000828224; POWO: https://powo.science.kew.org/taxon/77266950-1
= Campanulapolymorphavar.intercedensf.angustifolia Hruby, Magyar Bot. Lapok 29: 200 (1930); GBIF: https://www.gbif.org/species/5410043; WFO: http://www.worldfloraonline.org/taxon/wfo-0000828220; POWO: https://powo.science.kew.org/taxon/365601-4
= Campanulapolymorphavar.intercedensf.exigua Hruby, Magyar Bot. Lapok 29: 200 (1930); GBIF: https://www.gbif.org/species/5410058; WFO: http://www.worldfloraonline.org/taxon/wfo-0000828222; POWO: https://powo.science.kew.org/taxon/365603-4
= Campanulapolymorphavar.intercedensf.latifolia Hruby, Magyar Bot. Lapok 29: 199 (1930); GBIF: https://www.gbif.org/species/5410046; WFO: http://www.worldfloraonline.org/taxon/wfo-0000828226; POWO: https://powo.science.kew.org/taxon/365607-4
= Campanulapolymorphavar.intercedensf.reflectans Hruby, Magyar Bot. Lapok 29: 200 (1930); GBIF: https://www.gbif.org/species/5410039; WFO: http://www.worldfloraonline.org/taxon/wfo-0000828233; POWO: https://powo.science.kew.org/taxon/365614-4
= Campanulapolymorphavar.intercedensf.umbrosa Hruby, Magyar Bot. Lapok 29: 200 (1930); GBIF: https://www.gbif.org/species/5410036; WFO: http://www.worldfloraonline.org/taxon/wfo-0000828236; POWO: https://powo.science.kew.org/taxon/365619-4
= Campanulapolymorphavar.lepida Nyár. ex Hruby, Magyar Bot. Lapok 29: 201 (1930)
= Campanulapolymorphavar.pluriflora Nyár. ex Hruby, Magyar Bot. Lapok 29: 198 (1930)
= Campanulapolymorphavar.praticola Hruby, Magyar Bot. Lapok 29: 198 (1930), non C.witasekianavar.praticola Hruby; GBIF: https://www.gbif.org/species/5410053; IPNI: https://www.ipni.org/n/77268347-1; WFO: http://www.worldfloraonline.org/taxon/wfo-0000828231; POWO: https://powo.science.kew.org/taxon/77268347-1
= Campanulapolymorphavar.praticolaf.hirta (Nyár.) Hruby, Magyar Bot. Lapok 29: 199 (1930)
= Campanulapolymorphavar.praticolaf.pluriflora (Nyár.) Hruby, Magyar Bot. Lapok 29: 199 (1930)
= Campanulapolymorphavar.stenophylla (Schur) Hruby, Magyar Bot. Lapok 29: 205 (1930); GBIF: https://www.gbif.org/species/5410054; WFO: http://www.worldfloraonline.org/taxon/wfo-0000828235; POWO: https://powo.science.kew.org/taxon/365616-4
= Campanulapolymorphavar.stenophyllaf.brachyphylla Hruby, Magyar Bot. Lapok 29: 206 (1930); GBIF: https://www.gbif.org/species/5410056; WFO: http://www.worldfloraonline.org/taxon/wfo-0000828221; POWO: https://powo.science.kew.org/taxon/365602-4
= Campanulapolymorphavar.stenophyllaf.genuina Hruby, Magyar Bot. Lapok 29: 206 (1930)
= Campanulapolymorphavar.stenophyllaf.gracilis Hruby, Magyar Bot. Lapok 29: 206 (1930); GBIF: https://www.gbif.org/species/5410050; WFO: http://www.worldfloraonline.org/taxon/wfo-0000828223; POWO: https://powo.science.kew.org/taxon/365604-4
= Campanulapolymorphavar.typica Hruby, Magyar Bot. Lapok 29: 198, 201 (1930); GBIF: https://www.gbif.org/species/5410048; POWO: https://powo.science.kew.org/taxon/365617-4
= Campanulapolymorphavar.typicaf.fasciculata Nyár. ex Hruby, Magyar Bot. Lapok 29: 202 (1930)
= Campanulapolymorphavar.typicaf.fasciculatasubf.deltoidea Hruby, Magyar Bot. Lapok 29: 203 (1930)
= Campanulapolymorphavar.typicaf.kladnianioides Nyárady ex Hruby, Magyar Bot. Lapok 29: 201 (1930); GBIF: https://www.gbif.org/species/8517923; GBIF: https://www.gbif.org/species/5410051; WFO: http://www.worldfloraonline.org/taxon/wfo-0000828225; POWO: https://powo.science.kew.org/taxon/365606-4
= Campanulapolymorphavar.typicaf.latifolia Hruby, Magyar Bot. Lapok 29: 201 (1930); GBIF: https://www.gbif.org/species/5410046; WFO: http://www.worldfloraonline.org/taxon/wfo-0000828226; POWO: https://powo.science.kew.org/taxon/365607-4
= Campanulapolymorphavar.typicaf.latifoliasubf.umbrosa Hruby, Magyar Bot. Lapok 29: 202 (1930); GBIF: https://www.gbif.org/species/5410036; POWO: https://powo.science.kew.org/taxon/365618-4
= Campanulapolymorphavar.typicaf.lepida (Nyár.) Hruby, Magyar Bot. Lapok 29: 202 (1930); GBIF: https://www.gbif.org/species/5410037; WFO: http://www.worldfloraonline.org/taxon/wfo-0000828227; POWO: https://powo.science.kew.org/taxon/365608-4
= Campanulapolymorphavar.typicaf.lepidasubf.reflectans Hruby, Magyar Bot. Lapok 29: 202 (1930); GBIF: https://www.gbif.org/species/5410039; POWO: https://powo.science.kew.org/taxon/365613-4
= Campanulapolymorphavar.typicaf.saxiphila Hruby, Magyar Bot. Lapok 29: 204 (1930)
= Campanulapolymorphavar.typicaf.saxiphilasubf.reflectans Hruby, Magyar Bot. Lapok 29: 205 (1930)
= Campanularotundifoliasubsp.polymorpha (Witasek) Tacik in Jasiewicz, Monogr. Bot. 20: 254 (1965); GBIF: https://www.gbif.org/species/7685150; IPNI: https://www.ipni.org/n/77255879-1; WFO: http://www.worldfloraonline.org/taxon/wfo-0000828685; POWO: https://powo.science.kew.org/taxon/77255879-1
= *Campanulascheuchzeri* [unranked] β *dacica* Porcius, Enum. Pl. Phan. Naszód.: 37 (1878)
= Campanulascheuchzerivar.dacica Porcius, Fl. Naseud.: 98 (1885); GBIF: https://www.gbif.org/species/5410055; IPNI: https://www.ipni.org/n/77268262-1; WFO: http://www.worldfloraonline.org/taxon/wfo-0000828824; POWO: https://powo.science.kew.org/taxon/77268262-1
= Campanulascheuchzerivar.stenophylla Schur, Enum. Pl. Transsilv.: 443 (1866); GBIF: https://www.gbif.org/species/5410059; IPNI: https://www.ipni.org/n/77268259-1; WFO: http://www.worldfloraonline.org/taxon/wfo-0000828864; POWO: https://powo.science.kew.org/taxon/77268259-1; BHL: https://www.biodiversitylibrary.org/page/10544494#page/465
= *Campanulastenophylla* (Schur) Prain, Index Kew. Suppl. 4: 35 (1913) [nom. inval.]; GBIF: https://www.gbif.org/species/5410042; IPNI: https://www.ipni.org/n/77238117-1; WFO: http://www.worldfloraonline.org/taxon/wfo-0000829052; POWO: https://powo.science.kew.org/taxon/77238117-1; BHL: https://www.biodiversitylibrary.org/item/133932#page/47
= *Campanulastenophylla* (Schur) Witasek, Magyar Bot. Lapok 5: 238 (1906), non Boiss. & Heldr.; GBIF: https://www.gbif.org/species/7633288; IPNI: https://www.ipni.org/n/141149-1; BHL: https://www.biodiversitylibrary.org/item/202161#page/644
– *Campanulacarnica* auct. fl. transsilv., non Schiede
– *Campanulaconsanguinea* Simonk., Enum. Fl. Transsilv.: 385 (1886) [p. p.], non Schott; BHL: https://www.biodiversitylibrary.org/item/40105#page/449
– *Campanulakladniana* (Schur) Witasek, Abh. Zool.-Bot. Ges. Wien 1: 39 (1902) [p. p. min., non sensu Schur orig.]; GBIF: https://www.gbif.org/species/5411315; IPNI: https://www.ipni.org/n/140522-1; WFO: http://www.worldfloraonline.org/taxon/wfo-0000827551; POWO: https://powo.science.kew.org/taxon/140522-1
– *Campanulalinifolia* auct. [e.g., Wahlenb.], non Jacq.
– Campanulapolymorphaf.sciaphila Hruby, Magyar Bot. Lapok 29: 204 (1930) [nom. et des. inval.]; GBIF: https://www.gbif.org/species/5410041; WFO: http://www.worldfloraonline.org/taxon/wfo-0000828234; POWO: https://powo.science.kew.org/taxon/365615-4
– *Campanularotundifolia* L., Sp. Pl. 1: 163 (1753) [p. p. minor, tantum quod plantas ucrain. carpat.], non alior *; GBIF: https://www.gbif.org/species/5410907; IPNI: https://www.ipni.org/n/30036649-2; WFO: http://www.worldfloraonline.org/taxon/wfo-0000828550; POWO: https://powo.science.kew.org/taxon/30036649-2; BHL: https://www.biodiversitylibrary.org/page/358182#page/175
– *Campanulapusilla* auct. fl. ucrain. carpat., non Haenke
– *Campanulascheuchzeri* auct. [e.g., Reuss, Května Slov.: 278 (1853); Sagorski & Schneider, Fl. Centralkarp.: 369 (1891)], non Vill. *

##### Conservation status

In Ukraine – LC (Onyshchenko et al. 2022).

##### Distribution

Pancarpathian endemic.

##### Notes

In general, there are three subspecies of *C.tatrae* (https://powo.science.kew.org/taxon/141196-1, accessed on 05.06.2023) – subsp. tatrae, subsp. mentiens (Witasek) Kovanda and subsp. sudetica (Hruby) Kovanda. The last two subspecies occur exclusively in the Western Carpathians; therefore, only C.tatraesubsp.tatrae is represented in the flora of the Ukrainian Carpathians.

However, C.tatraesubsp.tatrae has a long and complex naming history. In the Ukrainian Carpathians, it combines two main taxa that are often distinguished as independent species (i.e. *C.polymorpha* (Witasek) Prain and *C.rotundifolia* L., p.p.) by certain authors (e.g. Dremliuga and Zyman (2012), Chopyk and Fedoronchuk (2015)). However, Kovanda (1975) conducted nomenclatural explorations and stressed the taxonomic concept that Witaseks applied to Schur's *C.kladniana* in her early publication (Witasek 1902). To avoid misinterpretation of plants from the Tatrae Mts. that were often incorrectly identified as *C.kladniana* (Schur) Witasek and synonymised with *C.polymorha*, Kovanda (1975) proposed using the name *C.tatrae* that has been published earlier by Borbás (1902) and had an unambiguous sense. Many infraspecific taxa from *Campanulascheuchzeri* auct., non Vill. seem to be a part of C.tatraesubsp.tatrae too (Geslot 1971). On the other hand, in the recent checklists (e.g. POWO, WFO and Euro+Med – https://powo.science.kew.org/taxon/77168970-1, https://list.worldfloraonline.org/wfo-0000838737, https://europlusmed.org/cdm_dataportal/taxon/c454d5af-c66c-4d0a-b009-a0e8754e9c9c, all databases being accessed on 05.06.2023), *C.scheuchzeriformis* Hayek and derived C.balcanicavar.scheuchzeriformis (Hayek) Hruby are wrongly listed amongst synonyms of *C.tatrae* subsp. *tatrae. Campanula scheuchzeriformis* occurs in North Albania (Hayek 1921, Hruby 1930). Such synonymisation probably results from confusion because Hayek (1921), in the protologue of *C.scheuchzeriformis*, mentioned that it is similar to *C.scheuchzeri.* Another taxon, C.rotundifoliavar.alpicola Hayek (≡ C.rotundifoliaf.alpicola (Hayek) Hruby), is also mistakenly indicated as a synonym of C.tatraesubsp.tatrae in the Worldplants checklist (https://www.worldplants.de/world-plants-complete-list/complete-plant-list?name=Campanula-tatrae, accessed on 07.06.2023) and GBIF (https://www.gbif.org/species/7222073, accessed on 05.06.2023). Finally, Worldplants wrongly indicate the presence of C.carnicasubsp.carnica Mert. & W.D.J.Koch for Ukraine. This species and subspecies do not occur in Ukraine. Such confusion of C.rotundifoliaf.alpicola probably results from the mistaken synonymisation of *C.kladniana* with C.carnicasubsp.carnica due to ambiguous interpretation of the taxonomic limits of *C.kladniana* (Podlech 1965, Kovanda 1975).

#### Phyteuma tetramerium

Schur, Verh. Mitth. Siebenbürg. Vereins Naturwiss. Hermannstadt 4: 47 (1853)

5C4A1E06-2289-5100-800E-B6F60350600D

https://www.catalogueoflife.org/data/taxon/4HK3D

https://www.gbif.org/species/3166600

urn:lsid:ipni.org:names:144500-1

http://www.worldfloraonline.org/taxon/wfo-0000816731

https://powo.science.kew.org/taxon/144500-1

https://species.wikimedia.org/wiki/Phyteuma_tetramerum

https://europlusmed.org/cdm_dataportal/taxon/a0297a55-f260-4c09-b97f-adcf68c1f1ee

https://www.worldplants.de/world-plants-complete-list/complete-plant-list/?name=Phyteuma-tetramerum

https://www.biodiversitylibrary.org/page/11525300#page/933

≡ *Phyteumatetramerum* Schur, Verh. Mitth. Siebenbürg. Vereins Naturwiss. Hermannstadt 4: 47 (1853) [ortho. var.]; IPNI: https://ipni.org/n/144500-1; WFO: http://www.worldfloraonline.org/taxon/wfo-0000816731; POWO: https://powo.science.kew.org/taxon/144500-1; BHL: https://www.biodiversitylibrary.org/page/11525300#page/933
= *Phyteumaspicatum* Baumg., Enum. Stirp. Transsilv. 1: 158 (1816), non L. nec Lapeyr.
= *Phyteumaspicatum* Nyman, Consp. Fl. Eur.: 484 (1879) [nom. illeg.], non L. nec Lapeyr.; GBIF: https://www.gbif.org/species/3166601; BHL: https://www.biodiversitylibrary.org/item/41446#page/495
– Phyteumaspicatumvar.tetramerum (Schur) Nyman sensu auct. multipl. [nom. nudum]; IPNI: https://www.ipni.org/n/77287349-1; WFO: http://www.worldfloraonline.org/taxon/wfo-0000821849; POWO: https://powo.science.kew.org/taxon/77287349-1; BHL: https://www.biodiversitylibrary.org/page/11015628#page/495

##### Conservation status

In Ukraine – LC (Onyshchenko et al. 2022).

##### Distribution

SE Carpathian endemic.

##### Notes

This species has several nomenclatural issues. All databases (accessed on 05.06.2023), except for IPNI, as well as most of the publications (e.g. Tzvelev (1995), Tasenkevich (1998), Chorney (2011), Chopyk and Fedoronchuk (2015), Mosyakin and Fedoronchuk (2015), Kliment et al. (2016) and Kliment et al. (2016)) indicate the name *P.tetramerum* as introduced by Schur (1853). This orthographical variant is the most abundant and commonly applied by botanists. However, in the original protologue and further (Schur 1866), Schur applied a slightly different name – *P.tetramerium*. It is unclear why and when the specific epithet lost the letter 'i'. Another issue with this species is that GBIF (https://www.gbif.org/species/3166601, accessed on 05.06.2023), WFO (http://www.worldfloraonline.org/taxon/wfo-0000821849, accessed on 05.06.2023), POWO (https://powo.science.kew.org/taxon/77287349-1, accessed on 05.06.2023) and Worldplants (https://www.worldplants.de/world-plants-complete-list/complete-plant-list?name=Phyteuma-tetramerum, accessed on 07.06.2023) provide the synonym P.spicatumvar.tetramerum (Schur) Nyman. IPNI (https://www.ipni.org/n/77287349-1, accessed on 05.06.2023) even has a link to the 'protologue' of P.spicatumvar.tetramerum on BHL (https://www.biodiversitylibrary.org/page/11015628#page/495, accessed on 05.06.2023). However, Nyman never made such a combination and, in the original publication, Nyman (1878) only indicated that *P.tetramerium* is a synonym for *P.spicatum*.

#### Phyteuma vagneri

A.Kern in Vágner, Máram. Növ.: 192 (1875) et A.Kern., Sched. Fl. Exs. Austro-Hung. [Kerner] 3: 107 (1884)

8B1A282B-CBCC-5053-8A5F-8136211B1DA2

https://www.catalogueoflife.org/data/taxon/4HK3J

https://www.gbif.org/species/3166602

urn:lsid:ipni.org:names:144507-1

http://www.worldfloraonline.org/taxon/wfo-0000816776

https://powo.science.kew.org/taxon/144507-1

https://europlusmed.org/cdm_dataportal/taxon/9b4fe0e6-8050-4e83-9efa-3e4a9024bd53

https://www.worldplants.de/world-plants-complete-list/complete-plant-list/?name=Phyteuma-vagneri

https://je.jacq.org/JE00020985

https://wu.jacq.org/WU0066442

https://wu.jacq.org/WU0066443

https://wu.jacq.org/WU0066444

https://wu.jacq.org/WU0066445

https://wu.jacq.org/WU0066446

https://w.jacq.org/W18870004173

https://w.jacq.org/W19260020726

https://plants.jstor.org/stable/10.5555/al.ap.specimen.je00020985

https://plants.jstor.org/stable/10.5555/al.ap.specimen.dao000457292

https://plants.jstor.org/stable/10.5555/al.ap.specimen.kw000115548

≡ *Phyteumaatropurpureum* Schur, Verh. Mitth. Siebenbürg. Vereins Naturwiss. Hermannstadt 3: 88 (1852) [nom. nudum], non Hoppe; GBIF: https://www.gbif.org/species/8164990; IPNI: https://ipni.org/n/77245178-1; WFO: http://www.worldfloraonline.org/taxon/wfo-0000821850; POWO: https://powo.science.kew.org/taxon/77245178-1; BHL: https://www.biodiversitylibrary.org/page/11526224#page/538
≡ Phyteumanigrumvar.atropurpureum Schur, Enum. Pl. Transsilv.: 430 (1866); GBIF: https://www.gbif.org/species/3166605; IPNI: https://ipni.org/n/77287825-1; WFO: http://www.worldfloraonline.org/taxon/wfo-0000821851; POWO: https://powo.science.kew.org/taxon/77287825-1; BHL: https://www.biodiversitylibrary.org/page/10544481#page/452
≡ *Phyteumaspiciforme* Rochel, Bot. Reise Banat: 69 (1838) [nom. nudum] et Rochel ex Domin & Podp., Klic Ulpne Kvet. Rep. Ceskoslov.: 542 (1928); GBIF: https://www.gbif.org/species/7536094; IPNI: https://ipni.org/n/144491-1; IPNI: https://ipni.org/n/77244339-1; WFO: http://www.worldfloraonline.org/taxon/wfo-0001351008; POWO: https://powo.science.kew.org/taxon/77244339-1
= *Phyteumamichelii* Sternh., Fl. Sieb.: 20 (1846), non alior
= Phyteumavagnerif.alpinum Rich. Schulz, Monogr. Phyteuma: 79 (1904); GBIF: https://www.gbif.org/species/3166606; WFO: https://list.worldfloraonline.org/wfo-0000821855; POWO: https://powo.science.kew.org/taxon/359508-4
= Phyteumavagnerif.brevibracteatum Rich. Schulz, Monogr. Phyteuma: 78 (1904); GBIF: https://www.gbif.org/species/3166603; WFO: https://list.worldfloraonline.org/wfo-0000821852; POWO: https://powo.science.kew.org/taxon/359505-4
= Phyteumavagnerif.grossidentatum Rich. Schulz, Monogr. Phyteuma: 78 (1904); GBIF: https://www.gbif.org/species/3166604; WFO: https://list.worldfloraonline.org/wfo-0000821853; POWO: https://powo.science.kew.org/taxon/359506-4
= Phyteumavagnerif.latibracteatum Rich. Schulz, Monogr. Phyteuma: 78 (1904); GBIF: https://www.gbif.org/species/3166608; WFO: https://list.worldfloraonline.org/wfo-0000821854; POWO: https://powo.science.kew.org/taxon/359507-4
= Phyteumavagnerivar.pallida Porcius, Enum. Pl. Phanerogam. Distr. Quondam Naszódiensis: 37 (1878)
– *Phyteumabetonicaefolium* Baumg., Mant.: 16 (1846) et auct. transsilv., non Vill. [nom. nudum ?]
– *Phyteumahalleri* auct. transsilv., non All.
– *Phyteumanigrum* auct. [e.g., Baumg.], non Schmalh.
– *Phyteumaovatum* auct. [e.g., Baumg.], non Schmalh.

##### Conservation status

In Ukraine – LC (Onyshchenko et al. 2022).

##### Distribution

SE Carpathian endemic.

#### Boraginales

51FCEB09-4F58-51D2-9670-D5097F0C25C9

#### Boraginaceae

70ACAB6F-FF03-5BCA-BCFB-BE4B63B4A7C6

#### Pulmonaria filarszkyana

Jáv., Bot. Közlem. 15: 52 (1916)

67A8D1AE-DFF5-5881-8223-B4B49F6E7EC9

https://www.catalogueoflife.org/data/taxon/4QGPK

https://www.gbif.org/species/9193276

https://www.gbif.org/species/7506258

urn:lsid:ipni.org:names:120366-1

http://www.worldfloraonline.org/taxon/wfo-0001215442

https://powo.science.kew.org/taxon/120366-1

https://species.wikimedia.org/wiki/Pulmonaria_filarszkyana

https://europlusmed.org/cdm_dataportal/taxon/f2a7dc7e-0988-4764-a575-9d361ab223ed

https://www.worldplants.de/world-plants-complete-list/complete-plant-list/?name=Pulmonaria-filarszkyana

https://plants.jstor.org/stable/10.5555/al.ap.specimen.s12-12742

≡ Pulmonariarubrasubsp.filarszkyana (Jáv.) Domin, Preslia 13-15: 175, in adnot. (1935); GBIF: https://www.gbif.org/species/7763716; IPNI: https://ipni.org/n/77252395-1; WFO: https://list.worldfloraonline.org/wfo-0001215862; POWO: https://powo.science.kew.org/taxon/77252395-1
≡ Pulmonariarubravar.filarszkyana (Jáv.) Guşul., Bul. Fac. St. Cern. 3: 330 (1929) *
– *Pulmonariaangustifolia* Kern., Monogr. Pulm.: 9 (1878) [p. p., quoad plantas marmaros. et rodn.], non L.
– *Pulmonariadacica* (Simonk.) Simonk., Enum. Fl. Transsilv.: 406 (1886) [p. p.] *; GBIF: https://www.gbif.org/species/8393929; IPNI: https://ipni.org/n/120357-1; POWO: https://powo.science.kew.org/taxon/120357-1; BHL: https://www.biodiversitylibrary.org/page/10524541#page/470
– *Pulmonariadacica* (Simonk.) Porcius [p. p., nom et des. invalid]; GBIF: https://www.gbif.org/species/5660545; IPNI: https://ipni.org/n/120358-1; WFO: http://www.worldfloraonline.org/taxon/wfo-0000397437
– Pulmonariarubravar.dacica Simonk., Math. Termeszettud. Közlem. 15: 583 (1878) [p. p.]; GBIF: https://www.gbif.org/species/7372640; POWO: https://powo.science.kew.org/taxon/3234704-4

##### Conservation status

In Ukraine – LC (Onyshchenko et al. 2022).

##### Distribution

SE Carpathian endemic.

#### Symphytum cordatum

Waldst. et Kit. ex Willd., Neue Schriften Ges. Naturf. Freunde Berlin 2: 121 (1799), non M.Bieb.

D3C00C7D-D7B0-51B0-8715-75F2C4BAFB99

https://www.catalogueoflife.org/data/taxon/53QF8

https://www.gbif.org/species/7377391

urn:lsid:ipni.org:names:120768-1

http://www.worldfloraonline.org/taxon/wfo-0000432288

https://powo.science.kew.org/taxon/120768-1

https://species.wikimedia.org/wiki/Symphytum_cordatum

https://europlusmed.org/cdm_dataportal/taxon/cfeda693-ff16-478b-85e7-6ce6913583b1

https://www.worldplants.de/world-plants-complete-list/complete-plant-list/?name=Symphytum-cordatum

https://plants.jstor.org/stable/10.5555/al.ap.specimen.m0188157

≡ *Symphytumcordatum* Waldst. & Kit., Descr. Icon. Pl. Hung. 1: 6, t. 7 (1799–1802), non M.Bieb.; GBIF: https://www.gbif.org/species/4067440; IPNI: https://www.ipni.org/n/120770-1; POWO: https://powo.science.kew.org/taxon/120768-1
= *Symphytumcordifolium* Baumg., Enum. Stirp. Transsilv. 1: 126 (1816) *; GBIF: https://www.gbif.org/species/4067436; IPNI: https://ipni.org/n/120771-1; WFO: http://www.worldfloraonline.org/taxon/wfo-0000432289; POWO: https://powo.science.kew.org/taxon/120771-1
= *Symphytumpannonicum* Pers., Syn. Pl. [Persoon] 1: 161 (1805) *; GBIF: https://www.gbif.org/species/4067084; IPNI: https://ipni.org/n/120821-1; WFO: http://www.worldfloraonline.org/taxon/wfo-0000432232; POWO: https://powo.science.kew.org/taxon/120821-1; BHL: https://www.biodiversitylibrary.org/page/234946#page/173
– *Symphytumcordatum* M.Bieb., Fl. Taur.-Caucas. 1: 130 (1808) [p. p., nom. inval.]; GBIF: https://www.gbif.org/species/7937522; IPNI: https://ipni.org/n/120769-1; WFO: http://www.worldfloraonline.org/taxon/wfo-0001350379; POWO: https://powo.science.kew.org/taxon/120769-1; BHL: https://www.biodiversitylibrary.org/page/11268210#page/138

##### Conservation status

In Ukraine – LC (Onyshchenko et al. 2022).

##### Distribution

Pancarpathian subendemic.

##### Notes

This species is closely and primarily associated with the Carpathian Mts. (Novikoff and Hurdu 2015); however, it also rarely occurs in the Ukrainian lowland areas in Volhyn (Chopyk 1976) and Podillia (Kobiv 2007, Chorney 2011) going far from the mountain range. As a result, Malynovskiy et al. (2002) considered it Carpathian-Volhynia-Podolian species and Chorney (2011) treated it as a non-endemic taxon with a Central European distribution range. Nevertheless, numerous authors (e.g. Pawłowski (1961), Tasenkevich (2003), Kobiv (2007), Piękoś-Mirkowa and Mirek (2011), Hurdu et al. (2012), Negrean et al. (2015), Novikoff and Hurdu (2015), Mráz et al. (2016) and Kliment et al. (2016)) treated *S.cordatum* as a Carpathian endemic or subendemic species. Considering the extension of *S.cordatum*'s distribution range to the lowlands, it cannot be considered endemic and, therefore, it is listed here as a subendemic. However, further phylogeographical studies of this species are of great interest and would clarify its disjunctive distribution pattern.

#### Brassicales

69D64D8E-BD04-5D59-8BD3-CFBB79AFD576

#### Brassicaceae

4A4F2FA9-333E-59F1-9E1F-2144E06B900F

#### Arabidopsis neglecta

(Schult.) O'Kane et Al-Shehbaz, Novon 7(3): 326 (1997)

3107228D-D579-5445-982F-74D1AFEEB420

https://www.catalogueoflife.org/data/taxon/G266

https://www.gbif.org/species/3052529

urn:lsid:ipni.org:names:997561-1

http://www.worldfloraonline.org/taxon/wfo-0000541803

https://powo.science.kew.org/taxon/997561-1

https://species.wikimedia.org/wiki/Arabidopsis_neglecta

https://europlusmed.org/cdm_dataportal/taxon/9d2eec80-d9a2-459d-a2fc-ec92315eca7d

https://www.worldplants.de/world-plants-complete-list/complete-plant-list/?name=Arabidopsis-neglecta

https://www.biodiversitylibrary.org/page/640143#page/326

≡ *Arabisneglecta* Schult., Oesterr. Fl., ed. 2: 248 (1814) *; GBIF: https://www.gbif.org/species/5377122; IPNI: https://ipni.org/n/278429-1; WFO: http://www.worldfloraonline.org/taxon/wfo-0000542551; POWO: https://powo.science.kew.org/taxon/278429-1
≡ *Cardaminopsisneglecta* (Schult.) Hayek, Fl. Steiermark 1: 480 (1908) *; GBIF: https://www.gbif.org/species/3052532; IPNI: https://ipni.org/n/280774-1; WFO: http://www.worldfloraonline.org/taxon/wfo-0000587713; POWO: https://powo.science.kew.org/taxon/280774-1; BHL: https://www.biodiversitylibrary.org/page/10560532#page/516
≡ *Erysimumneglectum* (Schult.) Kuntze, Revis. Gen. Pl. 2: 933 (1891); GBIF: https://www.gbif.org/species/3692864; IPNI: https://ipni.org/n/284014-1; WFO: http://www.worldfloraonline.org/taxon/wfo-0000678999; POWO: https://powo.science.kew.org/taxon/284014-1; BHL: https://www.biodiversitylibrary.org/page/4355#page/559
= *Arabistranssilvanica* Schur, Enum. Pl. Transsilv.: 43 (1866); GBIF: https://www.gbif.org/species/5377124; IPNI: https://ipni.org/n/278696-1; WFO: http://www.worldfloraonline.org/taxon/wfo-0000542956; POWO: https://powo.science.kew.org/taxon/278696-1; BHL: https://www.biodiversitylibrary.org/page/10544094#page/65
= *Arabisfloribunda* Schur, Enum. Pl. Transsilv.: 44 (1866); GBIF: https://www.gbif.org/species/5377121; IPNI: https://ipni.org/n/278219-1; WFO: http://www.worldfloraonline.org/taxon/wfo-0000542213; POWO: https://powo.science.kew.org/taxon/278219-1; BHL: https://www.biodiversitylibrary.org/page/10544095#page/66
= *Arabisglareosa* Schur, Verh. Mitth. Siebenbürg. Vereins Naturwiss. Hermannstadt 1: 106 (1850) et Verh. Mitth. Siebenbürg. Vereins Naturwiss. Hermannstadt 4: 59 (1853); GBIF: https://www.gbif.org/species/5377123; IPNI: https://ipni.org/n/278235-1; WFO: http://www.worldfloraonline.org/taxon/wfo-0000542238; POWO: https://powo.science.kew.org/taxon/278235-1; BHL: https://www.biodiversitylibrary.org/page/11525300#page/116; BHL: https://www.biodiversitylibrary.org/page/11525300#page/711; JACQ: https://gzu.jacq.org/GZU000054862; JACQ: https://gzu.jacq.org/GZU000276989; JSTOR Global Plants: https://plants.jstor.org/stable/10.5555/al.ap.specimen.gzu000054862; JSTOR Global Plants: https://plants.jstor.org/stable/10.5555/al.ap.specimen.gzu000276989
= Cardaminopsisneglectasubsp.glareosa (Schur) Soó, Acta Bot. Acad. Sci. Hung. 16 (3–4): 371 (1971); GBIF: https://www.gbif.org/species/3689071; IPNI: https://ipni.org/n/879116-1; WFO: http://www.worldfloraonline.org/taxon/wfo-0000587714; POWO: https://powo.science.kew.org/taxon/879116-1
– *Cardamineenneaphyllos* Turcz. [nom. inval., ex herb. KW], non (L.) Crantz ex Crantz
– *Dentariaenneaphyllos* auct. flora ucrain. carpat., non L. [ex herb. LWS] *

##### Conservation status

In Ukraine – NT (Onyshchenko et al. 2022).

##### Distribution

Pancarpathian endemic.

##### Notes

The distribution of *A.neglecta* in the Ukrainian Carpathians remains unclear due to frequent confusion with *A.arenosa* (L.) Lawalrée, which is widely spread both in mountains and lowlands (Pachschwöll and Pachschwöll 2019). Schmickl et al. (2012) recognised two cytotypes of A.neglecta–subsp.neglecta(diploid from the alpine habitats) andsubsp.robusta Schmickl et al. [nom. illeg.] (tetraploid occurring in different vegetation belts, including montane and submontane). Contrary to Schmickl et al. (2012), Knotek et al. (2020) reported that tetraploids are more successful in colonising alpine habitats, but, in general, both cytotypes can be present at different elevations. For Ukraine, Kolář et al. (2016) used only three diploid samples of *A.arenosa subsp. arenosa* sampled in lowermost altitudes near road and railway banks in the Lviv region (Ciscarpathia and Beskyds). Similarly, Knotek et al. (2020) used only two tetraploid alpine samples from the only mesoregion of the Ukrainian Carpathians, Svydovets Mts. Therefore, the ploidy level and diversity of *Arenosa* group in the Ukrainian Carpathians remain unclear, but it seems that *A.neglecta* is associated here with subalpine and alpine habitats only (Chopyk and Fedoronchuk 2015).

#### Cardamine glanduligera

O.Schwarz, Repert. Spec. Nov. Regni Veg. 46: 188 (1939)

4F33EACB-0CF3-5C17-9C2D-1E9B4880B021

https://www.catalogueoflife.org/data/taxon/5WZN4

https://www.gbif.org/species/3045875

urn:lsid:ipni.org:names:280334-1

http://www.worldfloraonline.org/taxon/wfo-0000586858

https://powo.science.kew.org/taxon/280334-1

https://species.wikimedia.org/wiki/Cardamine_glanduligera

https://europlusmed.org/cdm_dataportal/taxon/66346298-7ce1-4e25-b8f9-5488fd71f965

https://www.worldplants.de/world-plants-complete-list/complete-plant-list/?name=Cardamine-glanduligera

≡ *Dentariaglandulosa* Waldst. & Kit., Descr. Icon. Pl. Hung. 3: 302, t. 272 (1801); GBIF: https://www.gbif.org/species/5374792; IPNI: https://ipni.org/n/281965-1; WFO: http://www.worldfloraonline.org/taxon/wfo-0000641631; POWO: https://powo.science.kew.org/taxon/281965-1; JACQ: https://www.jacq.org/detail.php?ID=557296; JSTOR Global Plants: https://plants.jstor.org/stable/10.5555/al.ap.specimen.bm000750037; JSTOR Global Plants: https://plants.jstor.org/stable/10.5555/al.ap.specimen.lecb0001853
≡ *Cardamineglandulosa* (Waldst. & Kit.) Schmalh., Fl. Sredn. Yuzhn. Rossii 1: 50 (1895) [nom. illeg.], non Blanco; GBIF: https://www.gbif.org/species/3045876; IPNI: https://ipni.org/n/280336-1; WFO: http://www.worldfloraonline.org/taxon/wfo-0000586860; POWO: https://powo.science.kew.org/taxon/280336-1
≡ *Cruciferanovemfolia* E.H.L.Krause, Deutschl. Fl. (Sturm), ed. 2 6: 118 (1902); GBIF: https://www.gbif.org/species/5551108; IPNI: https://ipni.org/n/281820-1; WFO: http://www.worldfloraonline.org/taxon/wfo-0000626932; POWO: https://powo.science.kew.org/taxon/281820-1; BHL: https://www.biodiversitylibrary.org/page/55369098#page/120

##### Conservation status

In Ukraine – LC (Onyshchenko et al. 2022).

##### Distribution

Pancarpathian subendemic.

#### Erysimum witmanni transsilvanicum

(Schur) P.W.Ball, Feddes Repert. 69: 151 (1964)

8923633E-FBD0-511C-9619-9DC3AD16AB25

https://www.gbif.org/species/8043371

http://www.worldfloraonline.org/taxon/wfo-0001217687

https://powo.science.kew.org/taxon/3007567-4

https://europlusmed.org/cdm_dataportal/taxon/24dbb669-b2b9-4190-af4b-74f414833444

https://www.worldplants.de/world-plants-complete-list/complete-plant-list/?name=Erysimum-witmannii

≡ *Erysimumtranssilvanicum* Schur, Enum. Pl. Transsilv.: 57 (1866) *; CoL: https://www.catalogueoflife.org/data/taxon/3BBTL; GBIF: https://www.gbif.org/species/3048278; IPNI: https://ipni.org/n/284236-1; WFO: http://www.worldfloraonline.org/taxon/wfo-0000679281; POWO: https://powo.science.kew.org/taxon/284236-1; BHL: https://www.biodiversitylibrary.org/page/10544108#page/79; JACQ: https://www.jacq.org/detail.php?ID=365438; JACQ: https://www.jacq.org/detail.php?ID=365584; JACQ: https://www.jacq.org/detail.php?ID=365589; JSTOR Global Plants: https://plants.jstor.org/stable/10.5555/al.ap.specimen.lw00206088; JSTOR Global Plants: https://plants.jstor.org/stable/10.5555/al.ap.specimen.lw00206058; JSTOR Global Plants: https://plants.jstor.org/stable/10.5555/al.ap.specimen.lw00206087
= *Erysimumbaumgartenianum* Jáv., Magyar Bot. Lapok 11: 30 (1912), non Schur
= *Erysimumczetzianum* Schur, Enum. Pl. Transsilv.: 57 (1866); GBIF: https://www.gbif.org/species/3048279; IPNI: https://ipni.org/n/283752-1; WFO: http://www.worldfloraonline.org/taxon/wfo-0000678651; POWO: https://powo.science.kew.org/taxon/283752-1; BHL: https://www.biodiversitylibrary.org/page/10544108#page/79
= *Erysimumczetzianum* Schur ex Jáv., Magyar Bot. Lapok 11: 29 (1912); BHL: https://www.biodiversitylibrary.org/item/201840#page/553
= Erysimumpannonicumf.viridis Simonk., Termesz. Fuzet. 5: 55 (1881); BHL: https://www.biodiversitylibrary.org/item/96887#page/63
= Erysimumpumilumvar.transilvanica Schur, Verh. Mitth. Siebenburg. Vereins Naturwiss. Hermannstadt 10: 143 (1859); BHL: https://www.biodiversitylibrary.org/item/42663#page/387
= Erysimumtranssilvanicumf.czetzianum (Schur) Nyár., Fl. Rep. Pop. Roman. 3: 177 (1955)
= Erysimumtranssilvanicumf.luxurians Nyár., Fl. Rep. Pop. Roman. 3: 177, 640 (1955)
= Erysimumtranssilvanicumf.rarifolium Nyár., Fl. Rep. Pop. Roman. 3: 177, 640 (1955)
= Erysimumwitmanniisubsp.czetzianum (Schur) Zapał. [ex herb. Mądalski, nom. inval. ?]
= Erysimumwitmanniivar.czetzianum (Schur) Borza, Bul. Grăd. Bot. Cluj 26: (1946)
= Erysimumwitmanniivar.czetziano Nyár., Fl. Rep. Pop. Roman. 3: 174 (1955)
– *Erysimumcheiranthus* Herbich, Fl. Bucovina: 354 (1859), non alior
– *Erysimumodoratum* Baumg., Enum. Stirp. Transsilv. 2: 262 (1816) [p. p.], non Ehr.; GBIF: https://www.gbif.org/species/7699439; IPNI: https://ipni.org/n/284036-1; WFO: http://www.worldfloraonline.org/taxon/wfo-0000679040; POWO: https://powo.science.kew.org/taxon/284036-1
– *Erysimumpannonicum* auct. carpat., non Crantz
– *Erysimumwahlenbergii* Simonk., Enum. Fl. Transsilv.: 85 (1886), non Asch. & Engl.; GBIF: https://www.gbif.org/species/7583006; IPNI: https://ipni.org/n/284262-1; WFO: http://www.worldfloraonline.org/taxon/wfo-0001034410; BHL: https://www.biodiversitylibrary.org/page/10524220#page/149
– *Erysimumwitmannii* auct. flora ucrain. carpat. et Grec., Consp. Fl. Rom.: 61 (1891) [p. p.], non Zaw. *

##### Conservation status

In Ukraine – DD (Onyshchenko et al. 2022).

##### Distribution

SE Carpathian endemic.

##### Notes

Kliment et al. (2016) recognise three subspecies within E.witmanni–subsp.witmanni(Pancarpathian endemic),subsp.pallidiflorum(Szepligeti ex Jav.) Soó (Western Carpathian endemic) andsubsp.transsilvanicum (south-eastern Carpathian endemic). In the Ukrainian Carpathians, only E.witmannisubsp.transsilvanicum is very narrowly present in the Chorniy Dil mountain range (Chopyk and Fedoronchuk 2015). During my explorations, I have found in KW herbarium a few specimens collected by Ilyinska from lowlands (Brody and Zolochiv Districts of Lviv region and Tlumach District of Ivano-Frankivsk region) that were mistakenly identified as *E.witmanni*.

#### Noccaea dacica dacica

(Heuff.) F.K.Mey, Feddes Repert. 84(5-6): 464 (1973)

60B3F160-A787-5CAE-9501-8EFDCE991D15

https://www.gbif.org/species/7225610

urn:lsid:ipni.org:names:287746-1

http://www.worldfloraonline.org/taxon/wfo-0001217939

https://powo.science.kew.org/taxon/77224759-1

https://europlusmed.org/cdm_dataportal/taxon/279426f5-029b-419c-9000-f56c8dad0bff

https://www.worldplants.de/world-plants-complete-list/complete-plant-list/?name=Noccaea-dacica

≡ *Noccaeadacica* (Heuff.) F.K.Mey., Feddes Repert. 84(5-6): 464 (1973); CoL: https://www.catalogueoflife.org/data/taxon/47KQH; GBIF: https://www.gbif.org/species/8622945; IPNI: https://www.ipni.org/n/287746-1; WFO: http://www.worldfloraonline.org/taxon/wfo-0001217939; POWO: https://powo.science.kew.org/taxon/287746-1
≡ *Thlaspidacicum* Heuff., Oesterr. Bot. Z. 8: 26 (1858) et Verh. K.K. Zool.-Bot. Ges. Wien 8 (Abh.): 61 (1858) *; GBIF: https://www.gbif.org/species/3045405; IPNI: https://www.ipni.org/n/290543-1; WFO: http://www.worldfloraonline.org/taxon/wfo-0000407340; POWO: https://powo.science.kew.org/taxon/290543-1; BHL: https://www.biodiversitylibrary.org/page/12053912#page/257
= *Thlaspidacicum* [unranked] β *rodnense* Porcius, Enum. Pl. Phanerogam. Distr. Quondam Naszódiensis: 7 (1878)
= *Thlaspidacicum* [unranked] β *transsilvanicum* Porcius, Fl. Năsăud: 169 (1881)
= *Thlaspicommutatum* Rochel, Bot. Reise Banat: 83 (1838), non Reiche; GBIF: https://www.gbif.org/species/3045398; IPNI: https://www.ipni.org/n/290525-1; WFO: http://www.worldfloraonline.org/taxon/wfo-0001037431; POWO: https://powo.science.kew.org/taxon/290525-1
= *Thlaspicorongianum* Czetz ex Nyman, Consp. Fl. Eur. 1: 63 (1878) [ortho. var.]; GBIF: https://www.gbif.org/species/8492602; GBIF: https://www.gbif.org/species/3692457; IPNI: https://www.ipni.org/n/290531-1; WFO: http://www.worldfloraonline.org/taxon/wfo-0000407352; POWO: https://powo.science.kew.org/taxon/290531-1; BHL: https://www.biodiversitylibrary.org/page/11015205#page/72
= *Thlaspikorongianum* Czetz ex Nyman, Syll. Suppl.: 37 (1865); GBIF: https://www.gbif.org/species/8601316; GBIF: https://www.gbif.org/species/3045399; IPNI: https://www.ipni.org/n/290605-1; WFO: http://www.worldfloraonline.org/taxon/wfo-0000407324; POWO: https://powo.science.kew.org/taxon/290605-1; JACQ: https://wu.jacq.org/WU0034620
= *Thlaspitrojagense* Zapał., Rozprawy Wydziału Mat.-Przyrod. Akad. Um., Dział B. Nauki Biol. 13: 316, 317 (1913); GBIF: https://www.gbif.org/species/3045402; IPNI: https://www.ipni.org/n/290749-1; WFO: http://www.worldfloraonline.org/taxon/wfo-0001037439; POWO: https://powo.science.kew.org/taxon/290749-1
= Thlaspitrojagensef.abbreviatum Zapał., Rozprawy Wydziału Mat.-Przyrod. Akad. Um., Dział B. Nauki Biol. 13: 317 (1913)
– *Thlaspialpestre* auct. [e.g., Schur, Fuss., Baumg.], non L.
– *Thlaspirotundifolium* auct. fl. transsilv., non Gaud

##### Conservation status

In Ukraine – DD (Onyshchenko et al. 2022).

##### Distribution

SE Carpathian endemic.

##### Notes

Following POWO (https://powo.science.kew.org/taxon/287746-1, accessed on 05.06.2023), there are two subspecies of N.dacica–subsp.dacica(occurs in Romania and Ukraine) andsubsp.montenegrina F.K.Mey. (the distribution is limited to Montenegro – Meyer (1973)). The close species, *N.banatica* (R.Uechtr.) F.K.Mey. (≡ Thlaspidacicumsubsp.banaticum (R.Uechtr.) Nyár.), is sometimes included in N.dacicasubsp.dacica (e.g. in the Worldplants database – https://www.worldplants.de/world-plants-complete-list/complete-plant-list?name=Noccaea-dacica, accessed on 07.06.2023). However, Al-Shehbaz (2014) distinguished *N.banatica* as an independent species, while Kliment et al. (2016) outlined it as a South Carpathian endemic taxon. Therefore, only N.dacicasubsp.dacica occurs in the Ukrainian Carpathians.

#### Caryophyllales

866BEF3A-6780-5819-80E1-54467CB782D5

#### Caryophyllaceae

92AC6E36-C9BB-5271-94F7-DF249E7153A9

#### Dianthus spiculifolius

Schur, Enum. Pl. Transsilv.: 98 (1866), non Borbás

61E85F2F-8106-53E0-BB25-19161DC58620

https://www.catalogueoflife.org/data/taxon/35B4J

https://www.gbif.org/species/3808113

urn:lsid:ipni.org:names:153899-1

http://www.worldfloraonline.org/taxon/wfo-0000644280

https://powo.science.kew.org/taxon/153899-1

https://species.wikimedia.org/wiki/Dianthus_spiculifolius

https://europlusmed.org/cdm_dataportal/taxon/1b8d3f50-2765-46fd-a107-6f489b247cad

https://www.worldplants.de/world-plants-complete-list/complete-plant-list/?name=Dianthus-spiculifolius

https://www.biodiversitylibrary.org/page/10544149#page/120

≡ Dianthuskitaibeliisubsp.spiculifolius (Schur) Novák, Sborník Klubu Přírodověd. v Praze. Sv. 4. 1914–1920, Č. 4: 23(1) (1922) et Věst. Král. české spol. nauk. Tř. mat.-přírod. 1923(11): 30 (1924)
≡ Dianthuspetraeussubsp.spiculifolius (Schur) Ciocârlan, Illustr. Fl. Rom.: 217-223 (2000); GBIF: https://www.gbif.org/species/7886591
≡ Dianthusplumariussubsp.spiculifolius (Schur) Baksay, Symposia Biol. Hung. 12: 153 (1972)
= *Dianthusacicularis* Schur, Enum. Pl. Transsilv.: 98 (1866), non Fisch. ex Ledeb.; GBIF: https://www.gbif.org/species/3815472; IPNI: https://www.ipni.org/n/153009-1; BHL: https://www.biodiversitylibrary.org/page/10544149#page/120
= *Dianthusbrachyanthus* Schur, Enum. Pl. Transsilv.: 96 (1866), non Boiss.; GBIF: https://www.gbif.org/species/7371609; IPNI: https://www.ipni.org/n/153147-1; WFO: http://www.worldfloraonline.org/taxon/wfo-0000643176; POWO: https://powo.science.kew.org/taxon/153147-1; BHL: https://www.biodiversitylibrary.org/page/10544147#page/118; JACQ: https://www.jacq.org/detail.php?ID=366863; JSTOR Global Plants: https://plants.jstor.org/stable/10.5555/al.ap.specimen.lw00203891
= *Dianthuscarpathicus* Borbás, Termesz. Fuzet. 12: 44 (1889), non Woł. [nom. inval.]; GBIF: https://www.gbif.org/species/3813900; IPNI: https://www.ipni.org/n/153199-1; BHL: https://www.biodiversitylibrary.org/page/31106623#page/620
= *Dianthushungaricus* (Andrae) Simonk., Enum. Fl. Transsilv.: 121 (1886), non alior; BHL: https://www.biodiversitylibrary.org/item/40105#page/185
= *Dianthus microche[i]lus* B.S.Williams, Pinks Centr. Eur.: 37 (1890) et B.S.Williams ex Wettst., Oesterr. Bot. Z. 41: 176 (1891); GBIF: https://www.gbif.org/species/3810687; IPNI: https://www.ipni.org/n/153597-1; WFO: https://list.worldfloraonline.org/wfo-0000643859; POWO: https://powo.science.kew.org/taxon/153597-1; BHL: https://www.biodiversitylibrary.org/page/28710681#page/182
= *Dianthuspetraeus* Janka, Bot. Közlem. 12: 187 (1913), non Waldst. & Kit. nec M.Bieb.
= *Dianthuspetraeus* Kerner, Oesterr. Bot. Z. 18: 18, 126 (1868) [nom. nudum], non Waldst. & Kit. nec M.Bieb.; BHL: https://www.biodiversitylibrary.org/item/91216#page/26
= *Dianthusplumarius* Baumg., Enum. Stirp. Transsilv. 1: 390 (1816) et auct. transsilv., non L. nec Gunnerus
= Dianthusplumariusvar.erythrocalyx Schott ex Simonk., Enum. Fl. Transsilv.: 121 (1886); BHL: https://www.biodiversitylibrary.org/item/40105#page/185
= Dianthusplumariusvar.hungaricus Andrae, Bot. Zeitung 11: 436 (1853), non alior; BHL: https://www.biodiversitylibrary.org/item/105829#page/230
= *Dianthusserotinus* Barth, Verh. Mitth. Siebenbürg. Vereins Naturwiss. Hermannstadt 19: 144 (1868) [nom. nudum], non Waldst. & Kit.; BHL: https://www.biodiversitylibrary.org/item/110590#page/442
= *Dianthusserotinus* Salzer, Verh. Mitth. Siebenbürg. Vereins Naturwiss. Hermannstadt 15: 50 (1864) [nom. nudum], non Waldst. & Kit.; BHL: https://www.biodiversitylibrary.org/item/105000#page/546
= Dianthusspiculifoliusf.petraeiformis Novák, Sborník Klubu Přírodověd. v Praze. Sv. 4. 1914–1920, Č. 4: 25(3) (1922)

##### Conservation status

In Ukraine – DD (Onyshchenko et al. 2022).

##### Distribution

SE Carpathian endemic.

##### Notes

This species is probably absent in the recent flora of the Ukrainian Carpathians since no new finding confirms its presence. It was mentioned for Chyvchyny Mts. (south Bukovina), based on old herbarium specimens by Fedoronchuk and Chorney (2005). However, it is not listed for the flora of the Ukrainian Carpathians by Chopyk and Fedoronchuk (2015). While working in the Ukrainian herbaria, I could not locate any specimen of *D.spiculifolius* Probably such specimens could be refined in the Schur’s collection hosted at LW, but, unfortunately, this collection has remained permanently unavailable for the last few years.

Borbás (1889), on page 44, indicated that *D.carpathicus* Borbás (*D.callizonus × D.tenuifolius*) is a synonym of *D.brachyanthus* Schur, non Boiss. and indicated it in the protologue “in rupibus calcareis alp. Kyrálikő (i.e. Piatra Craiului Mts. in Hungarian), circa 2000 mt. s.m. Aug. 1858 (Schur !)”. Schur (1866), on page 96, as the protologue to *D.brachyanthus* wrote “Auf Kalkfelsen des Königstein (i.e. Piatra Craiului Mts. in German) bei Kronstadt (i.e. Brașov). 6000–7000'. Aug.” Williams (1890), on page 37 and Williams (1893), on page 415, synonymised *D.brachyanthus* with *D.microchelus* Williams, and also cited it for Kronstadt in Transylvania.

It is unclear why CoL (https://www.catalogueoflife.org/data/taxon/6CQ3R, accessed on 07.06.2023), GBIF (https://www.gbif.org/species/7267406, accessed on 07.06.2023), WFO (http://www.worldfloraonline.org/taxon/wfo-0000643860, accessed on 05.06.2023), POWO (https://powo.science.kew.org/taxon/urn:lsid:ipni.org:names:153598-1, accessed on 05.06.2023) and Worldplants (https://www.worldplants.de/world-plants-complete-list/complete-plant-list?name=Dianthus-microlepis, accessed on 05.06.2023), as well as *Fassou et al. (2022)*, provide *D.brachyanthus* Schur amongst synonyms for *D.microlepis* Boiss., because Boissier (1843), on page 22, described *D.microlepis* out of the Carpathian region, i.e. from Rumelia, Balkans. The current known distribution of *D.microlepis* is limited to Bulgaria, Greece, Montenegro, Bosnia and Herzegovina, North Macedonia, Serbia, Slovenia, Croatia and Kosovo (Marhold 2022). Perhaps, the synonymisation of the Carpathian *D.brachyanthus* Schur with Balcanian *D.microlepis* is a result of a confusion of *D.brachyanthus* in the sense of Schur (1866) [= *D.microchelus* Williams] with *D.brachyanthus* in the sense of earlier Boissier's publication (Boissier 1837: p. 85). Therefore, *D.brachyanthus* Schur, non Boiss. should be considered a synonym of *D.microchelus* Williams and *D.carpathicus* Borbás, non Woł. and, consequently, a synonym of *D.spiculifolius* Schur.

Interestingly, in all mentioned databases, including IPNI (https://www.ipni.org/n/153597-1, accessed on 05.06.2023), *D.microchelus* is misspelled as *D. microche[i]lus* and the incorrect place of publication of this name is indicated. It is wrongly stated that Williams published this species in 1891 in Oesterreichische Botanische Zeitschrift. Instead, Williams published it a year before, in 1890, in his monography “The pinks of Central Europe” (Williams 1890: p. 37), while later, in Oesterreichische Botanische Zeitschrift, the editor of this journal, Richard R. von Wettstein, published a brief review on Williams' book with the indication of newly-proposed taxa (Wettstein 1891: p. 176). Thus, Williams never personally published this name in Oesterreichische Botanische Zeitschrift.

#### Sabulina oxypetala

(Woł.) Mosyakin & Fedor., Phytotaxa 231(1): 96 (2015)

714DC2FA-1306-5E6A-8164-9C775EC996DE

https://www.catalogueoflife.org/data/taxon/78YD9

https://www.gbif.org/species/8565536

urn:lsid:ipni.org:names:77150589-1

https://powo.science.kew.org/taxon/77150589-1

https://www.worldplants.de/world-plants-complete-list/complete-plant-list/?name=Sabulina-oxypetala

≡ *Alsineoxypetala* Woł., Spraw. Komis. Fizjograf. 22(2): 214 (1888); GBIF: https://www.gbif.org/species/7758064; IPNI: https://www.ipni.org/n/150666-1; WFO: http://www.worldfloraonline.org/taxon/wfo-0001291954; POWO: https://powo.science.kew.org/taxon/2630520-4; JACQ: https://www.jacq.org/detail.php?ID=392011; JSTOR Global Plants: https://plants.jstor.org/stable/10.5555/al.ap.specimen.lw00059883
≡ *Alsineverna* [unranked] *oxypetala* Zapał. [nom. inval. ?]
≡ Alsinezarencznyivar.oxypetala Woł., Sprawozd. Kom. Fizjograf. 21(2): 111–139 (1887) [?]
≡ *Minuartiaoxypetala* (Woł.) Kulczyński, Fl. Polska 2: 231 (1921) *; GBIF: https://www.gbif.org/species/7504529; WFO: http://www.worldfloraonline.org/taxon/wfo-0001292114; POWO: https://powo.science.kew.org/taxon/3007630-4
≡ Minuartiavernasubsp.oxypetala (Woł.) G.Halliday, Feddes Repert. 69: 13 (1964); GBIF: https://www.gbif.org/species/8333640; WFO: http://www.worldfloraonline.org/taxon/wfo-0001292121; WFO: http://www.worldfloraonline.org/taxon/wfo-0000734418; POWO: https://powo.science.kew.org/taxon/2869879-4
≡ Minuartiavernavar.oxypetala (Woł.) Prodan, Fl. Rep. Pop. Rom. 2: 86 (1953)
≡ *Minuartiaverna* [unranked] B *attica* [unranked] *oxypetala* Graebn. in Asch. & Graebn., Syn. Mitteleur. Fl. 5(1): 745 (1918)
≡ Sabulinavernasubsp.oxypetala (Woł.) Dillenb. & Kadereit, Taxon 63(1): 88 (2014); GBIF: https://www.gbif.org/species/7684653; IPNI: https://www.ipni.org/n/77137604-1; POWO: https://powo.science.kew.org/taxon/77137604-1
= Alsinezarencznyivar.neglecta Zapał., Consp. Fl. Galic. Crit. 3: 26 (1911)
= Alsinezarencznyivar.neglectaf.ramificans Zapał., Consp. Fl. Galic. Crit. 3: 27 (1911)
= Alsinezarencznyivar.neglectaf.subcaespitosa Zapał., Consp. Fl. Galic. Crit. 3: 27 (1911)
= Alsinezarencznyivar.neglectaf.subcolorata Zapał., Consp. Fl. Galic. Crit. 3: 27 (1911)
= *Alsinezarencznyi* [unranked] c *oxypetalaf.acutissima* Zapał., Consp. Fl. Galic. Crit. 3: 27 (1911)
= *Alsinezarencznyi* [unranked] c *oxypetalaf.micropetala* Zapał., Consp. Fl. Galic. Crit. 3: 28 (1911)
= Minuartiavernavar.oxypetalaf.micropetala (Zapał.) Prodan, Fl. Rep. Pop. Rom. 2: 86 (1953)
– *Minuartiazarencznii* auct. [i.e., Chopyk 1976], non (Zapał.) Klokov

##### Conservation status

In Ukraine – EN (Onyshchenko et al. 2022).

##### Distribution

SE Carpathian endemic.

##### Notes

A rare stenoendemic, which occurs in the Ukrainian Carpathians only in the Chyvchyny Mts. (Fedoronchuk and Chorney 2009, Chorney 2011).

#### Sabulina pauciflora

(Kit.) A.Novikov, comb. nov.

C6CBA696-EFEF-5840-8AF5-387DB1CB906F

https://www.worldplants.de/world-plants-complete-list/complete-plant-list/?name=Minuartia-pauciflora

≡ *Alsinepauciflora* Kit. ex Nyman, Consp. Fl. Eur. 1: 119 (1878); GBIF: https://www.gbif.org/species/8455786; GBIF: https://www.gbif.org/species/3807842; IPNI: https://www.ipni.org/n/150674-1; WFO: http://www.worldfloraonline.org/taxon/wfo-0000527884; POWO: https://powo.science.kew.org/taxon/150674-1; BHL: https://www.biodiversitylibrary.org/page/11015261#page/128
≡ *Arenariapauciflora* Kit., Linnaea 32(4–5): 510 (1864), non Prodan; GBIF: https://www.gbif.org/species/7644104; IPNI: https://www.ipni.org/n/151539-1; WFO: http://www.worldfloraonline.org/taxon/wfo-0000546400; POWO: https://powo.science.kew.org/taxon/151539-1; BHL: https://www.biodiversitylibrary.org/page/118601#page/513
≡ *Minuartiapauciflora* (Kit.) Dvořaková, Preslia 75(4): 350 (2003) *; CoL: https://www.catalogueoflife.org/data/taxon/43KDB; GBIF: https://www.gbif.org/species/3811893; IPNI: https://www.ipni.org/n/60435605-2; WFO: http://www.worldfloraonline.org/taxon/wfo-0000378331; POWO: https://powo.science.kew.org/taxon/60435605-2
= *Alsineverna* [unranked] δ *carpatica* Porcius, Enum. Pl. Phanerogam. Distr. Quondam Naszódiensis: 11 (1878) et Anal. Acad. Rom.: 54 (1893)
= *Alsineverna* [unranked] a. zarencnyi (Zapał.) Hermann, Fl. Deutschl. Fennoskand.: 185 (1912)
= *Alsinezarencznyi* Zapał., Bull. Int. Acad. Sci. Cracovie, Cl. Sci. Math. 1910(3B): 168 (1910) et Consp. Fl. Galic. Crit. 3: 25 (1911) [excl. var. c]; GBIF: https://www.gbif.org/species/3817114; IPNI: https://www.ipni.org/n/150829-1; WFO: http://www.worldfloraonline.org/taxon/wfo-0001291956; POWO: https://powo.science.kew.org/taxon/150828-1
= Alsinezarencznyivar.divestita Zapał., Consp. Fl. Galic. Crit. 3: 27 (1911); GBIF: https://www.gbif.org/species/8108754; GBIF: https://www.gbif.org/species/11110050; POWO: https://powo.science.kew.org/taxon/3298158-4
= Alsinezarencznyivar.pseudogerardiana Zapał., Consp. Fl. Galic. Crit. 3: 28 (1911)
= Alsinezarencznyivar.zarencznyif.bryophila Zapał., Consp. Fl. Galic. Crit. 3: 26 (1911)
= Alsinezarencznyivar.zarencznyif.minima Zapał., Consp. Fl. Galic. Crit. 3: 26 (1911)
= Alsinezarencznyivar.zarencznyif.paucicaulis Zapał., Consp. Fl. Galic. Crit. 3: 26 (1911)
= Alsinezarencznyivar.zarencznyif.subpurpuea Zapał., Consp. Fl. Galic. Crit. 3: 26 (1911)
= Alsinezarencznyivar.zarencznyif.supraglandulosa Zapał., Consp. Fl. Galic. Crit. 3: 26 (1911)
= Minuartiavernasubsp.gerardii [unranked] b. *carpatica* (Porcius) Graebn. in Asch. & Graebn., Syn. Mitteleur. Fl. 5(1): 749 (1918) *
= *Minuartiazarecznyi* (Zapał.) Klokov, Fl. UkrSSR 4: 480 (1952) *; GBIF: https://www.gbif.org/species/7267413; IPNI: https://www.ipni.org/n/155697-1; WFO: http://www.worldfloraonline.org/taxon/wfo-0001292126; POWO: https://powo.science.kew.org/taxon/155697-1
= *Minuartiazarencznii* (Zapał.) Klokov, Fl. UkrSSR 4: 480 (1952) [ortho. var.]; GBIF: https://www.gbif.org/species/7267413; IPNI: https://www.ipni.org/n/155697-1; WFO: http://www.worldfloraonline.org/taxon/wfo-0001292126; POWO: https://powo.science.kew.org/taxon/155697-1
= Minuartiazarecznyivar.divestita (Zapał.) Tzvelev, Bot. Zhurn. 87(3): 125 (2002); GBIF: https://www.gbif.org/species/3810712; IPNI: https://www.ipni.org/n/20006371-1; POWO: https://powo.science.kew.org/taxon/20006371-1
– *Alsinegerardii* auct. flora carpat., non Willd.
– *Arenariagerardii* auct. fl. carpat., non Willd.
– *Alsineverna* auct. fl. carpat., non (L.) Wahlenb. nec Bartl
– *Alsineverna* Knapp, Pfl. Galic. u. Bukov.: 331 (1872) [p. p.], non Wahlenb. s. str. *
– *Minuartiagerardii* auct. fl. carpat., non (Willd.) Hayek *
– *Minuartiaverna* auct. flora carpat., non (L.) Hiern *
– *Minuartiaverna* Kulczyński, Fl. Polska 2: 230 (1921), non (L.) Hiern.
– *Minuartiaverna* [unranked] α *caespitosa* (Ehrn.) Graebn. in Asch. & Graebn., Syn. Mitteleur. Fl. 5(1): 742 (1918) sensu Tovt [ex herb. UU] *
– Minuartiavernasubsp.gerardii (Willd.) Graebn. in Asch. & Graebn., Syn. Mitteleur. Fl. 5(1): 747 (1918) [p. p., tantum quod plantas carpat.], non Sabulinavernasubsp.gerardii (Willd.) Dillenb. s. str. *
– Minuartiavernavar.gerardi Kulczyński, Fl. Polska 2: 230 (1921), non Schinz. & Keller
– *Sabulinagerardii* auct. fl. carpat., non (Willd.) Rchb.
– Sabulinavernasubsp.gerardii auct. fl. carpat., non (Willd.) Dillenb.
– *Tryphanegerardi* auct. fl. carpat., non (Willd.) Rchb.

##### Conservation status

In Ukraine – VU (Onyshchenko et al. 2022).

##### Distribution

Pancarpathian endemic.

##### Notes

A rare species mentioned (as *M.pauciflora*) only for three regions of the Ukrainian Carpathians – Chornohora, Maramures and Svydovets (Chorney and Fedoronchuk 2009, MEPNR of Ukraine 2021).

In general, *Sabulinaverna* (L.) Rchb. (= *Minuartiaverna* (L.) Hiern) has a wide distribution and branched infraspecific subdivision with variable acceptance by different authors. In particular, *M.verna* auct. fl. carpat., together with *M.zarecznyi* (Zapał.) Klokov, is considered a synonym of *M.pauciflora* (Mirek et al. 2020). Moreover, *M.pauciflora* is often wrongly reported from the Carpathians as *M.gerardii* (Willd.) Hayek, which is a species from the Alps (Chorney 2011, Kliment et al. 2016, Nunvářová Kabátová et al. 2019). Inconsistency in the taxonomic interpretation and unclear chorology of *M.gerardii* led to its consideration, including Carpathian plants, as a synonym of *M.verna* s. str. (i.e. M.vernasubsp.verna or Sabulinavernasubsp.verna) (Fedoronchuk and Mosyakin 2016). However, recent investigations showed that *M.pauciflora* is clearly distinguished from *M.verna* s. str. *Nunvářová Kabátová et al. (2019)* also confirmed the belonging of Carpathian plants identified as *M.gerardii* to *M.pauciflora*.

CoL (https://www.catalogueoflife.org/data/taxon/43KDB, accessed on 07.06.2023), GBIF (https://www.gbif.org/species/3811893, accessed on 05.06.2023), POWO (https://powo.science.kew.org/taxon/60435605-2, accessed on 07.06.2023) and Worldplants (https://www.worldplants.de/world-plants-complete-list/complete-plant-list?name=Minuartia-pauciflora, accessed on 07.06.2023) still provide *M.pauciflora* as an independent species from the genus *Minuartia*, while *M.verna* was reconsidered as belonging to the genus *Sabulina* Rchb. as *S.verna* (Fedoronchuk and Mosyakin 2016). To keep the nomenclatural consistency within *S.verna* group, the new combination *Sabulinapauciflora* (Kit.) A. Novikov, *comb. nov.* is proposed here.

Only two taxa from the *Sabulinaverna* group are present in the Ukrainian Carpathians (Chopyk and Fedoronchuk 2015) viz. *S.pauciflora, comb. nov.* (≡ *M.pauciflora*) and *S.oxypetala* (Woł.) Mosyakin & Fedor. (≡ *M.oxypetala* (Woł.) Kulczyński). Both species were previously interpreted as belonging to *Alsine* L. and were recently reconsidered within the genus *Sabulina* Rchb. (Mosyakin and Fedoronchuk 2015, Fedoronchuk and Mosyakin 2016, Nunvářová Kabátová et al. 2019).

#### Silene nutans dubia

(Herbich) Zapał., Bull. Int. Acad. Sci. Cracovie, Cl. Sci. Math., Sér. B, Sci. Nat. 11: 151 (1911)

AA696DDD-2803-52C0-88F7-6708FCAF7061

https://www.catalogueoflife.org/data/taxon/7J3SQ

https://www.gbif.org/species/7719003

urn:lsid:ipni.org:names:77250338-1

https://list.worldfloraonline.org/wfo-0000734610

https://powo.science.kew.org/taxon/77250338-1

https://species.wikimedia.org/wiki/Silene_dubia

https://europlusmed.org/cdm_dataportal/taxon/0d4533cf-8151-49a6-9c83-2a8a5b067164

https://www.worldplants.de/world-plants-complete-list/complete-plant-list/?name=Silene-nutans

≡ *Silenedubia* Herbich, Fl. Bucovina: 388 (1859), non alior; GBIF: https://www.gbif.org/species/7592009; POWO: https://powo.science.kew.org/taxon/2488403-4; JACQ: https://www.jacq.org/detail.php?ID=442588; JACQ: https://www.jacq.org/detail.php?ID=442591; JACQ: https://www.jacq.org/detail.php?ID=442592; JACQ: https://www.jacq.org/detail.php?ID=442595; JACQ: https://www.jacq.org/detail.php?ID=442597; JSTOR Global Plants: https://plants.jstor.org/stable/10.5555/al.ap.specimen.cher0200022; JSTOR Global Plants: https://plants.jstor.org/stable/10.5555/al.ap.specimen.cher0200023; JSTOR Global Plants: https://plants.jstor.org/stable/10.5555/al.ap.specimen.cher0200019; JSTOR Global Plants: https://plants.jstor.org/stable/10.5555/al.ap.specimen.cher0200020; JSTOR Global Plants: https://plants.jstor.org/stable/10.5555/al.ap.specimen.cher0200021
≡ *Silenedubia* Herbich ex Rohrb., Monogr. Silene: 217 (1869), non alior; GBIF: https://www.gbif.org/species/8297181; IPNI: https://www.ipni.org/n/157265-1; WFO: http://www.worldfloraonline.org/taxon/wfo-0000440631; BHL: https://www.biodiversitylibrary.org/page/15321329#page/229
≡ Silenenutansvar.dubia (Herbich) Zapał., Sprawozd. Kom. Fizjograf. 24: 111 (1889)
= Silenedubiavar.glabriuscula (Zapał.) Guşul., Fl. Rep. Pop. Rom. 2: 179 (1953)
= Silenedubiavar.hormuzakii Guşul., Fl. Rep. Pop. Rom. 2: 179 (1953)
= Silenedubiavar.hormuzakiif.acaulis Guşul., Fl. Rep. Pop. Rom. 2: 179, 665 (1953) [monster forma]
= Silenedubiavar.hormuzakiif.apricorum (Zapał.) Graebn. in Asch. & Graebn. emend. Guşul., Fl. Rep. Pop. Rom. 2: 179 (1953)
= Silenedubiavar.hormuzakiif.herbichii (Zapał.) Graebn. in Asch. & Graebn. emend. Guşul., Fl. Rep. Pop. Rom. 2: 179 (1953)
= Silenedubiavar.hormuzakiif.kelemenensis (Zapał.) Graebn. in Asch. & Graebn. emend. Guşul., Fl. Rep. Pop. Rom. 2: 179 (1953)
= Silenedubiavar.hormuzakiif.lilacina (Zapał.) Guşul., Fl. Rep. Pop. Rom. 2: 179 (1953)
= Silenedubiavar.hormuzakiif.robustior (Schur) Graebn. in Asch. & Graebn. emend. Guşul., Fl. Rep. Pop. Rom. 2: 179 (1953)
= Silenenutanssubsp.dubiavar.dubiaf.apricorum Zapał., Consp. Fl. Galic. Crit. 3: 195 (1911)
= Silenenutanssubsp.dubia [unranked] b *herbichii* Zapał., Consp. Fl. Galic. Crit. 3: 195 (1911)
= Silenenutanssubsp.dubia[unranked]akelemenensis Zapał., Consp. Fl. Galic. Crit. 3: 195 (1911)
= Silenenutanssubsp.dubia[unranked]akelemenensisf.lilacina Zapał., Consp. Fl. Galic. Crit. 3: 195 (1911)
= Silenenutanssubsp.dubiavar.dubiaf.luxuriosa Zapał., Consp. Fl. Galic. Crit. 3: 195 (1911)
= Silenenutanssubsp.dubiavar.dubiaf.tenuis Zapał., Consp. Fl. Galic. Crit. 3: 195 (1911)
= *Silenenutans* [unranked] c. *glabriuscula* Zapał., Consp. Fl. Galic. Crit. 3: 192 (1911); GBIF: https://www.gbif.org/species/11954760
= *Silenenutans* [unanked] β *transsilvanica* Grec., Consp. Fl. Rom.: 109 (1898)
= *Silenetranssilvanica* Schur, Oesterr. Bot. Z. 8: 22 (1858) [nom. nudum] et Oesterr. Bot. Z. 10: 181 (1860); GBIF: https://www.gbif.org/species/5587908; IPNI: https://www.ipni.org/n/158548-1; WFO: http://www.worldfloraonline.org/taxon/wfo-0000440073; POWO: https://powo.science.kew.org/taxon/158548-1; BHL: https://www.biodiversitylibrary.org/page/28724891#page/30; JACQ: https://www.jacq.org/detail.php?ID=369397; JACQ: https://www.jacq.org/detail.php?ID=369398; JACQ: https://www.jacq.org/detail.php?ID=369401; JACQ: https://www.jacq.org/detail.php?ID=369407; JACQ: https://www.jacq.org/detail.php?ID=369408; JACQ: https://www.jacq.org/detail.php?ID=369417; JSTOR Global Plants: https://plants.jstor.org/stable/10.5555/al.ap.specimen.lw00204020; JSTOR Global Plants: https://plants.jstor.org/stable/10.5555/al.ap.specimen.lw00204018a; JSTOR Global Plants: https://plants.jstor.org/stable/10.5555/al.ap.specimen.lw00204022; JSTOR Global Plants: https://plants.jstor.org/stable/10.5555/al.ap.specimen.lw00204019; JSTOR Global Plants: https://plants.jstor.org/stable/10.5555/al.ap.specimen.lw00204018b; JSTOR Global Plants: https://plants.jstor.org/stable/10.5555/al.ap.specimen.lw00204017
= Silenetranssilvanicavar.angustifolia Hormuz., Oesterr. Bot. Z. 61: 147 (1911); BHL: https://www.biodiversitylibrary.org/item/37201#page/159
= Silenesaxatilis [unranked] a racemosa Schur, Enum. Pl. Transsilv.: 101 (1866); BHL: https://www.biodiversitylibrary.org/item/7364#page/121
= Silenesaxatilis [unranked] a robustior Schur, Enum. Pl. Transsilv.: 101 (1866); BHL: https://www.biodiversitylibrary.org/item/7364#page/121; JSTOR Global Plants: https://plants.jstor.org/stable/10.5555/al.ap.specimen.lw00203992
– *Silenesaxatilis* Schur, Enum. Pl. Transsilv.: 101 (1866), non Sims nec M.Bieb.; BHL: https://www.biodiversitylibrary.org/item/7364#page/121

##### Conservation status

In Ukraine – LC (Onyshchenko et al. 2022).

##### Distribution

SE Carpathian endemic.

##### Notes

Following Euro+Med (https://europlusmed.org/cdm_dataportal/taxon/d201548b-883c-4f46-8151-aed4fac379ee, accessed on 05.06.2023) and Worldplants (https://www.worldplants.de/world-plants-complete-list/complete-plant-list?name=Silene-nutans, accessed on 07.06.2023), S.nutansL. includes three subspecies –subsp.nutans(most widely distributed, with Eurasian range),subsp.insubrica (Gaudin) Soldano (distributed mainly in the Alps, but also occurs in Greece, Hungary, Romania and some other countries) and subsp. dubia (occurs exclusively in the south-eastern Carpathians). Additionally, S.nutanssubsp.smithiana (Moss) Jeanm. & Bocquet distributed in France, Belgium, Great Britain and the Netherlands was described by Jeanmonod and Bocquet (1983). Silenenutanssubsp.smithiana in mentioned databases is synonymised with *S.nutans* susbp. *nutans*. However, recent studies showed that this subspecies is a separate taxon represented by two haplotypes (Martin et al. 2016). Besides this, POWO (https://powo.science.kew.org/taxon/157927-1, accessed on 05.06.2023) and WFO (https://list.worldfloraonline.org/wfo-0000440799, accessed on 05.06.2023) recognise S.nutanssubsp.livida (Willd.) Gremli. However, S.nutanssubsp.livida is a direct synonym of S.nutanssubsp.insubrica (Jeanmonod and Bocquet 1983, Soldano 1991, Soldano 2001).

Two of the four mentioned subspecies occur in the Ukrainian Carpathians (Chopyk and Fedoronchuk 2015) – *S.nutans* susbp. *nutans* (occupies light forested and adjacent habitats in the montane belt) and S.nutanssubsp.dubia (occupies rocky and stony slopes in the montane and subalpine belts).

#### Silene zawadzkii

Herbich, Enum. Pl. Galic. Bucow.: 191 (1835)

D376ECC9-F895-5B4C-BF1B-C6ABAD101D5D

https://www.catalogueoflife.org/data/taxon/9YK58

https://www.gbif.org/species/5587094

urn:lsid:ipni.org:names:158695-1

http://www.worldfloraonline.org/taxon/wfo-0000439914

https://powo.science.kew.org/taxon/158695-1

https://species.wikimedia.org/wiki/Silene_zawadzkii

https://europlusmed.org/cdm_dataportal/taxon/807e3ed7-4af8-4f62-ae46-a23e4f845c28

https://www.worldplants.de/world-plants-complete-list/complete-plant-list/?name=Silene-zawadzkii

≡ *Melandriumzawadskii* (Herbich) A.Braun, Flora 26: 387 (1843) [nom. nudum]; GBIF: https://www.gbif.org/species/3814125; WFO: http://www.worldfloraonline.org/taxon/wfo-0001292100; BHL: https://www.biodiversitylibrary.org/item/940#page/388
≡ *Elisanthezawadzkii* (Herbich) Fuss, Fl. Transsilv.: 106 (1866); GBIF: https://www.gbif.org/species/10950560; IPNI: https://www.ipni.org/n/154224-1; WFO: http://www.worldfloraonline.org/taxon/wfo-0000666384; POWO: https://powo.science.kew.org/taxon/154224-1
≡ *Elisanthezawadskii* (Herbich) Klokov, Fl. UkrSSR 4: 574 (1952) [nom. illeg.]; GBIF: https://www.gbif.org/species/3815102; IPNI: https://www.ipni.org/n/154225-1
≡ *Silenanthezawadzkii* (Herbich) Griseb. & Schenk, Arch. Naturgesch. (Berlin) 18(1): 300 (1852); GBIF: https://www.gbif.org/species/3811613; IPNI: https://www.ipni.org/n/156730-1; WFO: http://www.worldfloraonline.org/taxon/wfo-0000438300; POWO: https://powo.science.kew.org/taxon/156730-1; BHL: https://www.biodiversitylibrary.org/page/13707709#page/308

##### Conservation status

In Ukraine – VU (Onyshchenko et al. 2022).

##### Distribution

SE Carpathian endemic.

##### Notes

A rare species that occurs in the Ukrainian Carpathians only in the Chyvchyny Mts. (Chorney 2009a, Chopyk and Fedoronchuk 2015, MEPNR of Ukraine 2021). This species was considered to belong to the genera *Melandrium* Röhl., *Elisanthe* Rchb. or *Silenanthe* Griseb. & Schenk. It was also considered a synonym of Silenevulgaris(Moench)Garckesubsp.vulgaris, but recent molecular studies showed that this narrow endemic is an independent and well-separated species nested within Silenesect.Physolichnis s.l. (Martyniuk et al. 2018, Petri and Oxelman 2019, Jafari et al. 2020).

GBIF (https://www.gbif.org/species/3814125, accessed on 18.06.2023), WFO (http://www.worldfloraonline.org/taxon/wfo-0001292100, accessed on 18.06.2023) and Euro+Med (https://europlusmed.org/cdm_dataportal/taxon/807e3ed7-4af8-4f62-ae46-a23e4f845c28, accessed on 18.06.2023) provide the name *Melandriumzawadskii* (Herbich) A.Braun with reference to Braun (1843). This name is quite popular and often used in the Ukrainian herbaria and regional floras. However, in the original publication, Braun (1843) did not apply such a combination. Instead, he used the name *Silenezawadskii* and only indicated that this species belongs to the group Melandrien (Elisanthen). Therefore, it is not clear who was the actual author of the combination *M.zawadskii* and whether it was validly published at all.

#### Plumbaginaceae

0E05B27A-9CD1-5EDF-9F87-C17887C8B042

#### Armeria pocutica

Pawł., Fragm. Florist. Geobot. 8: 399 (1962)

20966748-D0D6-5CD7-A532-DB205E948AA2

https://www.catalogueoflife.org/data/taxon/5W4RS

https://www.gbif.org/species/5668250

urn:lsid:ipni.org:names:686381-1

http://www.worldfloraonline.org/taxon/wfo-0000549162

https://powo.science.kew.org/taxon/686381-1

https://europlusmed.org/cdm_dataportal/taxon/fd28cfaf-32e3-44bc-85d6-fa1e03aabe18

https://www.worldplants.de/world-plants-complete-list/complete-plant-list/?name=Armeria-pocutica

– *Armeriaelongata* auct., non (Hoffm.) Koch
– Armeriamaritimasubsp.elongata auct., non (Hoffm.) Bonnier
– *Armeriavulgaris* auct., non Willd.

##### Conservation status

In Ukraine – RE (Onyshchenko et al. 2022).

##### Distribution

SE Carpathian endemic.

##### Notes

This species is extinct in the Ukrainian Carpathians (Kagalo and Sytschak 2009b, MEPNR of Ukraine 2021).

#### Dipsacales

6D6E676A-A96F-5652-B84A-4F3758CADFAB

#### Caprifoliaceae

9C1D315E-7E57-5F41-93E4-6BDEFA803CEA

#### Scabiosa lucida barbata

Nyár., Enum. Pl. Vasc. Cheia Turzii: 280 (1939)

EA6C5BDD-7392-5A47-8AD4-3B080F676963

https://www.catalogueoflife.org/data/taxon/B89FK

urn:lsid:ipni.org:names:77252742-1

https://powo.science.kew.org/taxon/77252742-1

https://europlusmed.org/cdm_dataportal/taxon/8580928c-4139-4518-914f-d08b075d3436

https://www.worldplants.de/world-plants-complete-list/complete-plant-list/?name=Scabiosa-lucida

≡ *Scabiosabarbata* Nyár. ex Chopyk & Fedoronchuk, Fl. Ukr. Carpath.: 436 (2015) [des. et nom. invalid.] *
≡ Scabiosapseudobanaticasubsp.barbata (Nyár.) Chrtek, Preslia 57: 201 (1985); POWO: https://powo.science.kew.org/taxon/3007691-4
= *Asterocephaluslucidus* [unranked] a.alpicolus Schur, Enum. Pl. Transssilv.: 300 (1866); BHL: https://www.biodiversitylibrary.org/item/7364#page/320
= *Asterocephaluslucidus* [unranked] b. *subalpinus* Schur, Enum. Pl. Transssilv.: 300 (1866); BHL: https://www.biodiversitylibrary.org/item/7364#page/320
= Scabiosacolumbariasubsp.subalpina Brügger, Flora Curiensis. Naturgeschichtliche Beiträge zur Kenntniss der Umgebungen von Chur: 65 (1874) [nom. nudum]
= Scabiosacolumbariasubsp.subalpina (Brügger) Killias, Jahresber. Naturf. Ges. Graubündens 31: 82 (1887–1888); BHL: https://www.biodiversitylibrary.org/item/43011#page/278
= Scabiosacolumbariasubsp.lucidavar.subalpina (Brügger) Braun-Blanq., Jahresber. Naturf. Ges. Graubünd. 58: 94 (1918); GBIF: https://www.gbif.org/species/12141628
= Scabiosalucidasubsp.barbataf.alpicola (Schur) Prodan, Fl. Rep. Pop. Rom. 8: 685 (1961); POWO: https://powo.science.kew.org/taxon/3007684-4
= Scabiosalucidasubsp.barbataf.hirticaulis (Nyár.) Prodan, Fl. Rep. Pop. Rom. 8: 685 (1961)
= Scabiosalucidasubsp.barbataf.perramosa (Nyár.) Prodan, Fl. Rep. Pop. Rom. 8: 685 (1961)
= Scabiosalucidasubsp.barbataf.subalpina (Schur) Prodan, Fl. Rep. Pop. Rom. 8: 685 (1961); POWO: https://powo.science.kew.org/taxon/3007687-4
= Scabiosalucidaf.elata Nyár., Enum. Pl. Vasc. Cheia Turzii: 280 (1939); POWO: https://powo.science.kew.org/taxon/3007682-4
= Scabiosalucidaf.hirticaulis Nyár., Enum. Pl. Vasc. Cheia Turzii: 280 (1939); POWO: https://powo.science.kew.org/taxon/3007681-4
= Scabiosalucidaf.perramosa Nyár., Enum. Pl. Vasc. Cheia Turzii: 280 (1939); POWO: https://powo.science.kew.org/taxon/3007683-4
= Scabiosalucidaf.scaposa Nyár., Enum. Pl. Vasc. Cheia Turzii: 280 (1939); POWO: https://powo.science.kew.org/taxon/3007680-4
= Scabiosalucidavar.subalpina (Brügger) Hayek & Hegi, Ill. Fl. Mitt.-Eur. 6(1): 308 (1908)
= *Scabiosaopaca* Klokov, Novosti Sist. Vyssh. Nizsh. Rast.: 112 (1974) *; CoL: https://www.catalogueoflife.org/data/taxon/6Y55F; GBIF: https://www.gbif.org/species/4103554; IPNI: https://www.ipni.org/n/1004941-1; WFO: http://www.worldfloraonline.org/taxon/wfo-0001046436; POWO: https://powo.science.kew.org/taxon/1004941-1
= *Scabiosasubalpina* Brügger, Jahresber. Nat. Gesell. Graubünd. 29: 137 (1886); GBIF: https://www.gbif.org/species/4104181; IPNI: https://www.ipni.org/n/320160-1; WFO: http://www.worldfloraonline.org/taxon/wfo-0000500648; POWO: https://powo.science.kew.org/taxon/320160-1; BHL: https://www.biodiversitylibrary.org/page/11661731#page/167
– *Scabiosalucida* Vill., Prosp. Hist. Pl. Dauphiné: 18 (1779) [p. p., tantum quod plantas ucrain. carpat.], non W.T.Aiton *; GBIF: https://www.gbif.org/species/7475424; IPNI: https://www.ipni.org/n/319978-1; WFO: http://www.worldfloraonline.org/taxon/wfo-0000500124; POWO: https://powo.science.kew.org/taxon/319978-1
– Scabiosalucidasubsp.lucida Vill., Prosp. Hist. Pl. Dauphiné: 18 (1779) sensu Tasenkevych [non sensu orig.]

##### Conservation status

In Ukraine – LC (Onyshchenko et al. 2022).

##### Distribution

SE Carpathian endemic.

##### Notes

There are four (https://powo.science.kew.org/taxon/319978-1, accessed on 05.06.2023) to five (https://europlusmed.org/cdm_dataportal/taxon/e6eec090-a11f-40b2-a8d1-a9f47863d58f, accessed on 05.06.2023) accepted subspecies of S.lucidaVill. –subsp.lucida (non-endemic taxon distributed in European mountains, starting from ca. 1000 m elevation – Štěpánek and Holub (1997)), subsp. calcicola Błoński (endemic of W Carpathians – Štěpánek and Holub (1997), Danihelka et al. (2012)), subsp. pseudobanatica (Schur) Holub (problematic taxon with Carpathian-Pannonian distribution range that prefers lower elevations and occurs in Ukraine, Slovakia and Romania, but often overlooked or confused – Chrtek (1985), Chrtek and Goliašová (1985), Tasenkevich (2006)), subsp. stricta (Waldst. & Kit.) Jasiewicz (distributed in southern Europe) and subsp. barbata Nyár. (south-eastern Carpathian endemic – Kliment et al. (2016)).

In the Flora of UkrSSR (Kotov 1961: p. 378), S.lucidais indicated as havingvar.subalpina (Brügger) Hegi with *S.subalpina* Brügger, Zur. Fl. Tirol (1860) amongst its synonyms. However, POWO (https://powo.science.kew.org/taxon/77227194-1, accessed on 05.06.2023), WFO (https://list.worldfloraonline.org/wfo-0000500277, accessed on 05.06.2023) and Worldplants (https://www.worldplants.de/world-plants-complete-list/complete-plant-list?name=Scabiosa-columbaria, accessed on 07.06.2023) indicate *S.subalpina* to be a synonym for *S.columbaria* L., a quite distinct species. After checking, it was found that Brügger did not describe *S.subalpina* in his mentioned work “Zur Flora Tirols” (Brügger 1860). IPNI (https://www.ipni.org/n/320160-1, accessed on 05.06.2023) says that Brügger described this species much later, in 1887–1888 (i.e. *S.subalpina* Brügger, Jahresber. Naturf. Ges. Graubündens 31, Beil. 82 (1887-88). However, this is also wrong because IPNI references the work of Killias (1887), who only repeated an epithet *subalpina* for S.columbariasubsp.subalpina (the rank of subspecies is indicated by the author in the taxon’s description) following Brügger (1874), who, in turn, published this subspecies without the protologue. Considering the mistake in IPNI, it is worth noting that complete and correct nomenclatural citations for this subspecies should be S.columbariasubsp.subalpina Brügger, Fl. Cur.: 65 (1874) [nom. nudum] and S.columbariasubsp.subalpina (Brügger) Killias, Jahresber. Naturf. Ges. Graubündens 31: 82 (1887–1888). Nevertheless, this does not answer the question about where the initial Brügger’s species name has been applied. In fact, Brügger first applied the epithet *subalpina* in 1874 (without any protologue provided) and later, in 1886, with a subsequent self-reprint of the last paper in the same 1886 that, however, has changed pagination (Brügger 1874, Brügger 1886a, Brügger 1886b). Therefore the correct nomenclatural citation for this species should be *S.subalpina* Brügger, Fl. Cur.: 65 (1874) [nom. nudum] et Jahresber. Nat. Gesell. Graubünd.: 137 (1886). Brügger (1886a) and Brügger (1886b) described *S.subalpina* as an intermediate species between *S.columbaria* and *S.lucida.* It was Hayek and Hegi (1908), who, on page 308, determined *S.subalpina* belonging to *S.lucida* and applied the combination S.lucidavar.subalpina (Brügger) Hayek & Hegi, Ill. Fl. Mitt.-Eur. 6(1): 308 (1908). Schur (1866) also believed that *subalpina* plants belong to the *lucida* group and applied the new name *Asterocephaluslucidus* [unranked] b. *subalpinus* Schur, Enum. Pl. Transssilv.: 300 (1866). Later, Pawłowski (Planta Poloniae Exsiccata deposited at KW) synonymised S.lucidavar.subalpina with *S.lucidavar.lucida* Considering this, I believe that the mentioned databases mistakenly synonymise *S.subalpina* (and derivatives) with *S.columbaria*. Instead, *S.subalpina* should be considered as a synonym of *S.lucida.* Moreover, after herbarium inspection, I found that at least part of the specimens identified as S.lucidavar.subalpina belong to S.lucidasubsp.barbata.

It is important to note that Brügger mentioned many taxa (e.g. *Hepatica rhaetica, Malus hortensis, Batrachium micranthum, Nasturtium montanum* etc.) for the first in Flora Сuriensis (Brügger 1874); however, this book is entirely ignored by IPNI and, consequently, many other databases, for some reason.

#### Ericales

B98F9275-FB93-5317-99C1-288FB048AE81

#### Ericaceae

84FB99E5-96FE-5622-BA99-7D9FD22E22B4

#### Pyrola carpatica

Holub et Křísa, Folia Geobot. Phytotax. 6(1): 82 (1971)

9871E597-4ACC-5FBE-BB22-0607419A75C8

https://www.catalogueoflife.org/data/taxon/6WQBQ

https://www.gbif.org/species/4171403

urn:lsid:ipni.org:names:706939-1

http://www.worldfloraonline.org/taxon/wfo-0000396313

https://powo.science.kew.org/taxon/706939-1

https://europlusmed.org/cdm_dataportal/taxon/ab1d2989-c95f-4369-9f82-7c9ceb6aca26

https://www.worldplants.de/world-plants-complete-list/complete-plant-list/?name=Pyrola-carpatica

https://prc.jacq.org/PRC454358

https://prc.jacq.org/PRC454359

https://plants.jstor.org/stable/10.5555/al.ap.specimen.prc454359

https://plants.jstor.org/stable/10.5555/al.ap.specimen.prc454358

≡ Pyrolarotundifoliasubsp.carpatica (Holub & Křísa) Váczy & Beldie, Fl. Rep. Soc. Rom. 13: 46 (1976)
– *Pyrolaintermedia* auct., non Schleich. ex Arcang.
– *Pyrolaintermedia* Schleich. sensu Szafer in Kulczyński & Pawłowski, Rośliny Polskie: 459 (1924) [nom. illeg.], non Schleich. ex Arcang., Comp. Fl. Ital.: 460 (1882)
– *Pyrolarotundifolia* [unranked] *arenaria* Scheele sensu Jáv., Magyar Fl.: 797 (1924)
– Pyrolarotundifoliasubsp.intermedia (Alef.) Wohlfahrt in W.D.J.Koch, Syn. Deut. Schweiz. Fl., Bd. 2: 1946 (1902) [p. p., tantum quod plantas carpat.]; GBIF: https://www.gbif.org/species/12116320
– Pyrolarotundifoliasubsp.intermedia(Schleich.) Dostál, Květena ČSR: 1115 (1949) [p. p., tantum quod plantas carpat., excl.var.arenaria Koch; nom. illeg.]; GBIF: https://www.gbif.org/species/11041003

##### Conservation status

In Ukraine – CR (Onyshchenko et al. 2022).

##### Distribution

Pancarpathian endemic.

##### Notes

High-mountain species occurring in the Ukrainian Carpathians only in a few subalpine and alpine habitats on Chornohora and Svydovets (Holub and Křísa 1971, Parnikoza and Gilchuk 2002, Chopyk and Fedoronchuk 2015).

#### Primulaceae

6E98A68B-73F4-575C-8168-E9EF4CC5681C

#### Soldanella hungarica

Simonk., Enum Fl. Transsilv.: 461 (1886) et Oesterr. Bot. Z. 39: 219 (1889)

ACCF6078-CBB3-576B-A17F-C208E6D5BFB0

https://www.catalogueoflife.org/data/taxon/4Y57T

https://www.gbif.org/species/4005274

urn:lsid:ipni.org:names:702873-1

http://www.worldfloraonline.org/taxon/wfo-0000504539

https://powo.science.kew.org/taxon/702873-1

https://europlusmed.org/cdm_dataportal/taxon/2946e82e-d01f-4883-aba9-46020cc08b2e

https://www.worldplants.de/world-plants-complete-list/complete-plant-list/?name=Soldanella-hungarica

https://www.biodiversitylibrary.org/page/10524596#page/525

**A. *Soldanellahungarica* Simonk.**, Enum Fl. Transsilv.: 461 (1886) et Oester. Bot. Z. 39: 219 (1889) [s. str.] *
≡ Soldanellahungaricasubsp.hungarica Simonk., Enum Fl. Transsilv.: 461 (1886) et Oesterr. Bot. Z. 39: 219 (1889) [s. str.]; CoL: https://www.catalogueoflife.org/data/taxon/5L7SN; GBIF: https://www.gbif.org/species/7683860; IPNI: https://ipni.org/n/702873-1; WFO: https://list.worldfloraonline.org/wfo-0000504539; POWO: https://powo.science.kew.org/taxon/702873-1; Euro+Med: https://europlusmed.org/cdm_dataportal/taxon/08c3b726-14a8-483b-8f42-ee2b144702fb; BHL: https://www.biodiversitylibrary.org/page/10524596#page/525
≡ Soldanellamontanasubsp.hungarica (Simonk.) Lüdi in Hegi, Illustr. Fl. Mittel-Europa 5(3): 1827 (1927); GBIF: https://www.gbif.org/species/7912534; WFO: http://www.worldfloraonline.org/taxon/wfo-0001105527
≡ Soldanellamontanavar.hungarica (Simonk.) Grinţ., Gen. Soldan. Haretii: 10 (1908)
≡ Soldanellaalpinavar.hungarica (Simonk.) Stojanoff & Stefanoff, Ann. Arch. Min. Agri. Dom. Roy. Bulg. 5: 865 (1925) [p. p.]
= Soldanellamajorf.parviflora Morariu in Morariu, Nyár. & Guşul., Fl. Rep. Pop. Rom. 7: 642 (1960) [nom. inval.]
= Soldanellamajorf.purpureifolia R.Rös., Comun. Bot. 7: 58 (1963) [nom. inval.]
= *Soldanellapseudomontana* F.K.Meyer, Haussknechtia 2: 15 (1985); GBIF: https://www.gbif.org/species/4005090; IPNI: https://ipni.org/n/929081-1; WFO: http://www.worldfloraonline.org/taxon/wfo-0000504554; POWO: https://powo.science.kew.org/taxon/929081-1
– *Soldanellaalpina* [unranked] *minor* Clus., Rar. Plant. Hist.: 309 (1601) [p.p.]
– *Soldanellaalpina* [unranked] β *minor* (Clus.) Neilr. [p.p.], Nachtraege Fl. Wien: 219 (1851) et Fl. Wien, Bd. 2: 219 (1868), non Seringe *; GBIF: https://www.gbif.org/species/9326958; POWO: https://powo.science.kew.org/taxon/3221241-4
– *Soldanellaalpina* [unranked] a minor Schur [p.p., nom. illeg.], Enum. Pl. Transsilv.: 556 (1866), non Seringe; POWO: https://powo.science.kew.org/taxon/3221253-4; BHL: https://www.biodiversitylibrary.org/item/7364#page/576
– Soldanellahungaricavar.minor (Schur) Vierh. in Hannig & Winkler, Pflanzenareale 1(1): Karte 7–8 (1926) [p. p.]; POWO: https://powo.science.kew.org/taxon/3221255-4
– Soldanellamajorf.hungarica (Simonk.) Jáv., Fl. Hung.: 811 (1925) [p. p.]
– Soldanellamajorf.macrocarpa Morariu in Morariu, Nyár. & Guşul., Fl. Rep. Pop. Rom. 7: 642 (1960) [p. p., nom. inval.]
– Soldanellamontanasubsp.hungaricavar.minor (Schur) Vierch. [nom. inval., ex herb. LWS] *
– Soldanellamontanavar.hungaricaf.minor (Schur) G.Kozij [nom. inval., ex herb. LWS] *
– Soldanellamontanavar.minor (Schur) Borbás, Beih. Bot. Centralb. 10: 282 (1901) [p. p.]; POWO: https://powo.science.kew.org/taxon/3221254-4
**B. *Soldanellamajor* (Neilr.) Vierh. in Urban & Graebn.**, Festschr. Asch.: 502 (1904), **emend. Zhang & Kadereit**, Nordic J. Bot. 22(2): 153 (2002) *; GBIF: https://www.gbif.org/species/8202303; IPNI: https://www.ipni.org/n/77205570-1; WFO: http://www.worldfloraonline.org/taxon/wfo-0000747205; POWO: https://powo.science.kew.org/taxon/77205570-1; Euro+Med: https://europlusmed.org/cdm_dataportal/taxon/f3a6a754-dd75-45ff-9787-5944c19710b0; Worldplants: https://www.worldplants.de/world-plants-complete-list/complete-plant-list/?name=Soldanella-major; BHL: https://www.biodiversitylibrary.org/item/42427#page/574; JACQ: https://je.jacq.org/JE00006613
≡ *Soldanellaalpina* [unranked] α *major* Neilr., Nachtraege Fl. Wien: 219 (1851) et Fl. Wien, Bd. 2: 219 (1868) *; GBIF: https://www.gbif.org/species/8334456; WFO: http://www.worldfloraonline.org/taxon/wfo-0000747204; POWO: https://powo.science.kew.org/taxon/2900745-4
≡ Soldanellamontanasubsp.hungaricavar.major (Neilr.) Lüdi in Hegi, Illustr. Fl. Mittel-Europa 5(3): 1827 (1930)
= *Soldanellastiriaca* F.K.Meyer, Haussknechtia 2: 20 (1985) [nom. inval., superfl.]; GBIF: https://www.gbif.org/species/4005020; POWO: https://powo.science.kew.org/taxon/3221343-4; JACQ: https://gzu.jacq.org/GZU000274107; JACQ: https://wu.jacq.org/WU0025155; JSTOR Global Plants: https://plants.jstor.org/stable/10.5555/al.ap.specimen.m0002803; JSTOR Global Plants: https://plants.jstor.org/stable/10.5555/al.ap.specimen.g00440606; JSTOR Global Plants: https://plants.jstor.org/stable/10.5555/al.ap.specimen.g00440605; JSTOR Global Plants: https://plants.jstor.org/stable/10.5555/al.ap.specimen.je00006613; JSTOR Global Plants: https://plants.jstor.org/stable/10.5555/al.ap.specimen.g00440607; JSTOR Global Plants: https://plants.jstor.org/stable/10.5555/al.ap.specimen.c10017495
– Soldanellahungaricasubsp.major (Neilr.) Pawłowska, Fragm. Florist. Geobot. 9: 11 (1963) [p. p.] *; CoL: https://www.catalogueoflife.org/data/taxon/5L7SP; GBIF: https://www.gbif.org/species/7505299; WFO: http://www.worldfloraonline.org/taxon/wfo-0000747203; POWO: https://powo.science.kew.org/taxon/2900744-4
– Soldanellaalpinavar.vulgaris Seringe, Mus. Helv. Hist. Nat., Ser. Bot. 1: 83 (1823) [p. p., nom. inval.]; GBIF: https://www.gbif.org/species/9560877; POWO: https://powo.science.kew.org/taxon/3255240-4
– Soldanellamajorsubsp.margittaniana Fodor [nom. nudum, ex herb. UU] *
**C. *Soldanellamarmarossiensis* Klášt.**, Preslia 9: 19 (1930), **emend. Zhang & Kadereit**, Nordic J. Bot. 22(2): 148 (2002) *; GBIF: https://www.gbif.org/species/10930409; IPNI: https://www.ipni.org/n/702877-1; WFO: http://www.worldfloraonline.org/taxon/wfo-0001105526; POWO: https://powo.science.kew.org/taxon/702877-1; Worldplants: https://www.worldplants.de/world-plants-complete-list/complete-plant-list/?name=Soldanella-haretii; JACQ: https://je.jacq.org/JE00024665; JACQ: https://je.jacq.org/JE00024666
≡ Soldanellarichterisubsp.marmarossiensis (Klášt.) Niederle, Skalnickáruv rok 75: 27 (2017); GBIF: https://www.gbif.org/species/11089257; GBIF: https://www.gbif.org/species/9291156; IPNI: https://ipni.org/n/60473714-2; WFO: http://www.worldfloraonline.org/taxon/wfo-0001368119; POWO: https://powo.science.kew.org/taxon/60473714-2
= *Soldanellaharetii* Grinţ., Gen. Soldan. Soldan. Haretii: 7 (1908); GBIF: https://www.gbif.org/species/10744534; IPNI: https://ipni.org/n/77205518-1; POWO: https://powo.science.kew.org/taxon/77205518-1
= Soldanellamajorf.haretii (Grinţ.) Guşul. in Morariu, Nyár. & Guşul., Fl. Rep. Pop. Rom. 7: 67 (1960); POWO: https://powo.science.kew.org/taxon/3221191-4
= Soldanellamontanavar.repanda Grinţ., Gen. Soldan. Soldan. Haretii: 12 (1908)
– Soldanellamontanasubsp.faceta A.Kress, Primulaceen-Studien 11: 22 (1993) [p. p.]; GBIF: https://www.gbif.org/species/4005175; POWO: https://powo.science.kew.org/taxon/3221190-4
– Soldanellamontanasubsp.hungaricavar.marmarossiensis (Klášt.) Fodor, Fl. Zakarpattia: 57 (1974) [p. p.]

##### Conservation status

In Ukraine – LC (Onyshchenko et al. 2022).

##### Distribution

Pancarpathian endemic.

##### Notes

*Soldanellahungarica* is a critical taxon considered here in a broad sense due to its unclear chorology and taxonomy in the Ukrainian Carpathians, with provisional aggregation of *S.hungarica* Simonk. [s. str.], *S.major* (Neilr.) Vierh. in Urban & Graebn. and *S.marmarossiensis* Klášt. that are recognised separately by Zhang and Kadereit (2004).

#### Fabales

CA89DA9D-29A4-50C2-AB1D-0F9D4185919E

#### Fabaceae

199A91D3-D790-5FB4-88ED-CCCD73378EEB

#### Genista tinctoria oligosperma

(Andrae) Soó, Feddes Repert. 83(3): 169 (1972)

0B9F29EE-4AC0-5B7B-B43A-65D936A5CE3C

https://www.catalogueoflife.org/data/taxon/9DRQQ

https://www.gbif.org/species/8231729

https://www.gbif.org/species/7840990

urn:lsid:ipni.org:names:885285-1

http://www.worldfloraonline.org/taxon/wfo-0000208982

https://powo.science.kew.org/taxon/885285-1

https://europlusmed.org/cdm_dataportal/taxon/120f2415-eff0-4cbd-bc7c-04d4e826ac24

https://www.worldplants.de/world-plants-complete-list/complete-plant-list/?name=Genista-tinctoria

≡ *Genistaoligosperma* (Andrae) Simonk., Enum. Fl. Transsilv.: 169 (1886) *; GBIF: https://www.gbif.org/species/5347633; WFO: http://www.worldfloraonline.org/taxon/wfo-0000214674; POWO: https://powo.science.kew.org/taxon/2819438-4; BHL: https://www.biodiversitylibrary.org/item/40105#page/233
≡ Genistatinctoriasubsp.oligosperma (Andrae) Malinovsky, Ukr. Bot. J. 19(3): 75 (1962) [comb. invalid.]
≡ Genistatinctoriavar.oligosperma Andrae, Bot. Zeitung 11: 440 (1853); GBIF: https://www.gbif.org/species/5347624; WFO: http://www.worldfloraonline.org/taxon/wfo-0000208981; POWO: https://powo.science.kew.org/taxon/2819649-4; BHL: https://www.biodiversitylibrary.org/item/105829#page/232
= *Genistaalpicola* Schur, Enum. Pl. Transsilv.: 145 (1866); GBIF: https://www.gbif.org/species/5627746; IPNI: https://ipni.org/n/495869-1; WFO: http://www.worldfloraonline.org/taxon/wfo-0000696334; POWO: https://powo.science.kew.org/taxon/495869-1; BHL: https://www.biodiversitylibrary.org/page/10544196#page/167; JACQ: https://www.jacq.org/detail.php?ID=373262; JSTOR Global Plants: https://plants.jstor.org/stable/10.5555/al.ap.specimen.lw00205812
= Genistaoligospermaf.alpicola (Schur) Morariu, Fl. Rep. Pop. Rom. 5: 62 (1957)
= Genistaoligospermaf.ghisae Pawł., Bul. Grăd. Bot. Cluj 19: 6 (1939)
= *Genistarupestris* Schur, Enum. Pl. Transsilv.: 145 (1866) *; GBIF: https://www.gbif.org/species/5347637; IPNI: https://ipni.org/n/496316-1; WFO: http://www.worldfloraonline.org/taxon/wfo-0000208980; POWO: https://powo.science.kew.org/taxon/496316-1; BHL: https://www.biodiversitylibrary.org/page/10544196#page/167; JACQ: https://www.jacq.org/detail.php?ID=446081; JACQ: https://www.jacq.org/detail.php?ID=446082; JSTOR Global Plants: https://plants.jstor.org/stable/10.5555/al.ap.specimen.lw00205824; JSTOR Global Plants: https://plants.jstor.org/stable/10.5555/al.ap.specimen.lw00205825; JSTOR Global Plants: https://plants.jstor.org/stable/10.5555/al.ap.specimen.m0219545
= *Genistasigeriana* Fuss, Fl. Transsilv.: 149 (1866); GBIF: https://www.gbif.org/species/5626815; IPNI: https://ipni.org/n/496357-1; WFO: http://www.worldfloraonline.org/taxon/wfo-0000696633; POWO: https://powo.science.kew.org/taxon/496357-1
= Genistatinctoriavar.prostrata auct., non Bab.
– *Genistaprocumbens* Baumg. ex Fuss, Fl. Transsilv.: 150 (1866) [nom. inval.], non alior

##### Conservation status

In Ukraine – EN (Onyshchenko et al. 2022).

##### Distribution

SE Carpathian endemic.

##### Notes

There are two (https://www.worldplants.de/world-plants-complete-list/complete-plant-list?name=Genista-tinctoria, accessed on 07.06.2023) to five (https://powo.science.kew.org/taxon/496408-1, accessed on 05.06.2023) subspecies within G.tinctoria L. – subsp. tinctoria (has a wide Eurasian distribution with a secondary presence on other continents), subsp. ovata (Waldst. & Kit.) Arcang. (has a western–south-eastern European distribution), subsp. insubrica (Brügger) Pignatti (endemic to Italy, sometimes, for example, by Worldplants, considered as a synonym of subsp. tinctoria), subsp. littoralis (Corb.) Rothm. (occurs in France and Italy, sometimes considered a synonym of subsp. tinctoria) and subsp. oligosperma (occurs in Ukrainian and Romanian Carpathians).

Genistatinctoriasubsp.oligosperma has been considered extinct for the Ukrainian Carpathians and previously has been reported only from the Maramures Mts. (Kagalo 2009). However, Kobiv et al. (2017) recently rediscovered a small population on Mt. Berlebashka (Latundur) in the Maramures Mts. Chopyk and Fedoronchuk (2015) also mentioned this subspecies for Pip Ivan Chornohirskiy Mt. (Chornohora Mts.). However, the presence of G.tinctoriasubsp.oligosperma in Chornohora Mts. requires validation because I found no respective herbarium material, except a doubtful (hard to identify unambiguously) specimen of Fodor collected from ‘Polonyna Kvasy’ in 1966 (unnumbered specimen deposited at UU).

GBIF (https://www.gbif.org/species/5347632, https://www.gbif.org/species/11406566, accessed on 05.06.2023), POWO (https://powo.science.kew.org/taxon/885285-1, accessed on 05.06.2023) and WFO (https://list.worldfloraonline.org/wfo-0000208982, accessed on 05.06.2023) mistakenly indicate that *G.tenuifolia* Loisel. and *G.tinctoria subsp. tenuifolia* (Loisel.) Pignatti are synonyms of *G.tinctoria* subsp. *oligosperma. Genistatenuifolia* has been described from Cavaglià in Piedmont, Italy (Loiseleur-Deslongchamps 1810) and has nothing in common with G.tinctoriasubsp.oligosperma.

#### Lathyrus transsilvanicus

(Spreng.) Rchb.f., Icon. Fl. Germ. Helv. 22: t. 220, fig. 4, nr. 8-12 (1886)

08129659-B3F7-5AFD-986B-3236A4BDED60

https://www.catalogueoflife.org/data/taxon/9D78Y

https://www.gbif.org/species/5356553

urn:lsid:ipni.org:names:502057-1

http://www.worldfloraonline.org/taxon/wfo-0000209548

https://powo.science.kew.org/taxon/502057-1

https://species.wikimedia.org/wiki/Lathyrus_transsylvanicus

https://europlusmed.org/cdm_dataportal/taxon/399ea63e-d18b-499b-9eed-0b615c34b13c

https://www.worldplants.de/world-plants-complete-list/complete-plant-list/?name=Lathyrus-transsylvanicus

≡ *Lathyrustranssilvanicus* (Spreng.) R.M.Fritsch, Sitzungsber. Kaiserl. Akad. Wiss., Math.-Naturwiss. Cl., Abt. 1, 104: 517 (1895) [nom. inval.]; GBIF: https://www.gbif.org/species/8329194; IPNI: https://ipni.org/n/502057-1; BHL: https://www.biodiversitylibrary.org/item/120550#page/599
≡ Lathyruslaevigatussubsp.transsylvanicus (Spreng.) Breistr., Bull. Soc. Bot. France 87: 53 (1940); GBIF: https://www.gbif.org/species/5356554; WFO: http://www.worldfloraonline.org/taxon/wfo-0000209549; POWO: https://powo.science.kew.org/taxon/3013336-4
≡ Lathyruslinnaeif.transsilvanicus (Spreng.) Rouy in Rouy & Foucad, Fl. France 5: 269 (1899)
≡ Lathyrusluteussubsp.transsylvanicus (Spreng.) Dostal, Květena ČSR: 821 (1949); GBIF: https://www.gbif.org/species/5356555; WFO: http://www.worldfloraonline.org/taxon/wfo-0000209552; POWO: https://powo.science.kew.org/taxon/3013337-4
≡ *Lathyrusluteus* [unranked] a transsilvanicus (Spreng.) Ascherson & Graebn., Syn. Mitteleur. Fl. 6(2): 1044 (1906–1910)
≡ *Lathyrusluteus* [unranked] c *transsylvanicus* (Spreng.) Beck in Rchb., Icon. Fl. Germ. Helv. 22: 155 (1903); GBIF: https://www.gbif.org/species/11385726; WFO: http://www.worldfloraonline.org/taxon/wfo-0001446301; POWO: https://powo.science.kew.org/taxon/3013335-4; BHL: https://www.biodiversitylibrary.org/item/29430#page/165
≡ Orobusluteussubsp.transsylvanicus (Spreng.) Nyman, Consp. Fl. Eur. 1: 204 (1878) [nom. et des. inval.]; GBIF: https://www.gbif.org/species/11408855; WFO: http://www.worldfloraonline.org/taxon/wfo-0001448072; POWO: https://powo.science.kew.org/taxon/3013334-4; BHL: https://www.biodiversitylibrary.org/page/11015817#page/213
≡ *Orobustranssylvanicus* Spreng., Syst. Veg., ed. 16, 3: 260 (1826); GBIF: https://www.gbif.org/species/2962476; IPNI: https://www.ipni.org/n/urn:lsid:ipni.org:names:511062-1; WFO: http://www.worldfloraonline.org/taxon/wfo-0000209547; POWO: https://powo.science.kew.org/taxon/511062-1; BHL: https://www.biodiversitylibrary.org/page/792612#page/261
= Lathyrustranssilvanicusf.trichocarpus Borbás in Nyár., Herb. Kv. fl.: 335 (1941-1944)
– *Orobuslaevigatus* Baumg., Enum. Stirp. Transsilv. 2: 329 (1816), non Waldst. & Kit.

##### Conservation status

In Ukraine – EN (Onyshchenko et al. 2022).

##### Distribution

Pancarpathian subendemic.

##### Notes

Obsolescent species, which is narrowly distributed only in the volcanic part of the Ukrainian Carpathians. It is listed by the Red Book of Ukraine (Prots and Kish 2009, MEPNR of Ukraine 2021).

GBIF (https://www.gbif.org/species/8318457) provides the name L.laevigatussubsp.transsylvanicus (Spreng.) Soó without indicating the publication details, which seems to be some technical mistake since I could not locate a publication where Soó applied such a name.

#### Trifolium sarosiense

Hazsl., Éjsz. Magyarh. Vir.: 76 (1864) et Hazsl. ex Neilr., Diagn. Gefaesspfl.: 35 (1864)

BA0F3996-5935-5045-9EA1-85CB1E21FE42

https://www.catalogueoflife.org/data/taxon/58Q5X

https://www.gbif.org/species/5358815

https://www.gbif.org/species/8013811

urn:lsid:ipni.org:names:523672-1

http://www.worldfloraonline.org/taxon/wfo-0000163734

https://powo.science.kew.org/taxon/523672-1

https://species.wikimedia.org/wiki/Trifolium_medium_var._sarosiense

https://europlusmed.org/cdm_dataportal/taxon/61648c41-a0e4-4b5d-adb9-cda45bced2c5

https://www.worldplants.de/world-plants-complete-list/complete-plant-list/?name=Trifolium-medium

≡ Trifoliumflexuosumsubsp.sarosiense (Hazsl.) Gibelli & Belli, Mem. Reale Accad. Sci. Torino ser. 2, 39 (1): 333 (1889); GBIF: https://www.gbif.org/species/11394034; WFO: http://www.worldfloraonline.org/taxon/wfo-0001449053; POWO: https://powo.science.kew.org/taxon/3223699-4; BHL: https://www.biodiversitylibrary.org/item/138459#page/381
≡ Trifoliummediumsubsp.sarosiense (Hazsl.) Simonk., Enum. Fl. Transsilv.: 180 (1887) *; GBIF: https://www.gbif.org/species/5358814; WFO: http://www.worldfloraonline.org/taxon/wfo-0000214196; POWO: https://powo.science.kew.org/taxon/2873267-4; BHL: https://www.biodiversitylibrary.org/item/40105#page/244
≡ Trifoliummediumvar.sarosiense (Hazsl.) A.Nyár. in Săvul., Fl. Rep. Pop. Rom. 5: 208 (1952); GBIF: https://www.gbif.org/species/7456349; WFO: http://www.worldfloraonline.org/taxon/wfo-0001449124; POWO: https://powo.science.kew.org/taxon/3223843-4
= *Trifoliumbanaticum* (Heuff.) Májovský, Acta Fac. Rerum Nat. Univ. Comen., Bot. 35: 6 (1988); GBIF: https://www.gbif.org/species/5633914; IPNI: https://ipni.org/n/946118-1; WFO: http://www.worldfloraonline.org/taxon/wfo-0000412696; POWO: https://powo.science.kew.org/taxon/946118-1
= Trifoliummediumsubsp.banaticum (Heuff.) Hendrych, Preslia 28: 405 (1956); GBIF: https://www.gbif.org/species/5358817; WFO: http://www.worldfloraonline.org/taxon/wfo-0000214194; POWO: https://powo.science.kew.org/taxon/2873266-4
= Trifoliummediumvar.banaticum Heuff., Verh. K.K. Zool.-Bot. Ges. Wien 8(Abh.): 89 (1858); GBIF: https://www.gbif.org/species/8309775; WFO: http://www.worldfloraonline.org/taxon/wfo-0000185251; POWO: https://powo.science.kew.org/taxon/2873265-4
= Trifoliummediumvar.sarosiensef.bracteolatum A.Nyár. in Săvul., Fl. Rep. Pop. Rom. 5: 208, 540 (1952)
= Trifoliummediumvar.sarosiensef.eciliatum A.Nyár. in Săvul., Fl. Rep. Pop. Rom. 5: 208, 540 (1952)
= Trifoliumsarosiensesubsp.banaticum (Heuff.) Holub, Folia Geobot. Phytotax. 18(2): 205 (1983); GBIF: https://www.gbif.org/species/5632674; IPNI: https://ipni.org/n/922821-1; WFO: http://www.worldfloraonline.org/taxon/wfo-0001365469; POWO: https://powo.science.kew.org/taxon/922821-1
= *Trifoliummedium* [unranked] e *humile* Schur, Enum. Pl. Transsilv.: 155 (1866); BHL: https://www.biodiversitylibrary.org/item/7364#page/175; JACQ: https://www.jacq.org/detail.php?ID=374793; JSTOR Global Plants: https://plants.jstor.org/stable/10.5555/al.ap.specimen.lw00205914

##### Conservation status

In Ukraine – NE (Onyshchenko et al. 2022).

##### Distribution

Pancarpathian subendemic.

##### Notes

For T.mediumvar.sarosiense, GBIF (https://www.gbif.org/species/7456349, accessed on 05.06.2023) mistakenly indicated the authorship (Hazsl.) Săvul. & Rayss. Instead of this, it should be (Hazsl.) A.Nyár. in Săvul. (Săvulescu 1952).

#### Gentianales

1C1FBF4C-CC60-5040-8690-A156B20F3416

#### Gentianaceae

5232C2F9-487D-5992-83D0-95FD01640B6E

#### Gentiana laciniata

Kit. ex Kanitz, Verh. Zool.-Bot. Ges. Wien 12: 572 (1862)

791836EE-AA8D-569E-9F09-B1E53D6271B4

https://www.catalogueoflife.org/data/taxon/3FMBY

https://www.gbif.org/species/7483254

https://www.gbif.org/species/7654718

https://www.gbif.org/species/9234218

urn:lsid:ipni.org:names:77205254-1

http://www.worldfloraonline.org/taxon/wfo-0000698412

https://powo.science.kew.org/taxon/77205254-1

https://species.wikimedia.org/wiki/Gentiana_pyrenaica

https://europlusmed.org/cdm_dataportal/taxon/eea7efa0-65c0-4870-963d-199a771d42d8

https://www.worldplants.de/world-plants-complete-list/complete-plant-list/?name=Gentiana-pyrenaica

https://www.biodiversitylibrary.org/item/95692#page/112

≡ Ciminalisdshimilensissubsp.laciniata (Kit. ex Kanitz) Zuev, Turczaninowia 22(3): 147 (2019); GBIF: https://www.gbif.org/species/11090477; IPNI: https://ipni.org/n/77205255-1; WFO: http://www.worldfloraonline.org/taxon/wfo-1200008725; POWO: https://powo.science.kew.org/taxon/77205255-1
≡ Gentianapyrenaicavar.laciniata (Kit. ex Kanitz) Jáv., Shed. Fl. Hung. Exs. 8: Nr 786 (1927) *; POWO: https://powo.science.kew.org/taxon/2933142-4
= *Gentianavagneriana* Janka, Oesterr. Bot. Z. 35: 109 (1885); POWO: https://powo.science.kew.org/taxon/2933141-4; BHL: https://www.biodiversitylibrary.org/item/91439#page/117
= *Gentianawagneri* Janka, Oesterr. Bot. Z. 35: 109 (1885) [nom. inval.; ortho. var.]; GBIF: https://www.gbif.org/species/7631972; WFO: http://www.worldfloraonline.org/taxon/wfo-0000698952; BHL: https://www.biodiversitylibrary.org/item/91439#page/117
– *Gentianapyrenaica* auct. fl. ucrain. carpat., non L. *

##### Conservation status

In Ukraine – LC (Onyshchenko et al. 2022).

##### Distribution

SE Carpathian endemic.

##### Notes

The Red Book of Ukraine lists this species as rare (Zyman and Shiyan 2009, MEPNR of Ukraine 2021).

This species is of unclear taxonomy and chorology. In Ukraine, it is often equated with *G.pyrenaica* L. (Mosyakin and Fedoronchuk 2015). Tutin (1972) also considered *G.laciniata* a synonym of *G.pyrenaica*. In GBIF (https://www.gbif.org/species/7270399, accessed on 05.06.2023), WFO (https://list.worldfloraonline.org/wfo-0000698412, accessed on 05.06.2023) and Euro+Med (https://europlusmed.org/cdm_dataportal/taxon/eea7efa0-65c0-4870-963d-199a771d42d8, accessed on 05.06.2023), *G.laciniata* is also regarded as a synonym of *G.pyrenaica.* However, Tzvelev (1978) argued its independent position due to differences in calyx morphology (3–5 mm long laciniate vs. 2–3 mm long ovate-laciniate segments) and distribution (Carpathian vs. Pyrenean). Kliment et al. (2016), based on the studies of Rybczyński et al. (2014), who showed its isolated position from *G.pyrenaica*, also concluded that *G.laciniata* is an independent species, an eastern Carpathian endemic narrowly distributed in the Ukrainian Carpathians. Nevertheless, Zuev (2019) later reconsidered it as a subspecies within the genus *Ciminalis*, i.e. C.dshimilensissubsp.laciniata (Kit. ex Kanitz) Zuev and indicated a much wider, Caucasian-Balkanian-Carpathian, distribution. Zuev (2019) also proposed a new combination for *G.pyrenaica* – *Ciminalispyrenaica* (L.) Zuev and placed it together with C.dshimilensissubsp.laciniata in the same section Pyrenaicae (Grossh.) Zuev. However, he did not indicate either distribution or morphological differences between *C.pyrenaica* and *C.dshimilensissubsp.L.ciniata.* Later, Favre et al. (2020) made several taxonomic recombinations, based on molecular data. In particular, Favre et al. (2020) placed *G.pyrenaica* into the section Chondrophyllae Bunge (≡ Ciminalissect.Chondrophyllae (Bunge) Zuev), but the position and identity of *G.laciniata* remained unclear. Hence, due to controversial opinions, the taxonomic status and chorology of *G.laciniata* require further discussion.

#### Swertia punctata

Baumg., Enum. Stirp. Transsilv. 1: 190 (1816)

B9DF5C86-F0FC-5BA0-AF47-70490EBD9C4F

https://www.gbif.org/species/5595494

urn:lsid:ipni.org:names:371052-1

http://www.worldfloraonline.org/taxon/wfo-0000498017

https://powo.science.kew.org/taxon/371052-1

https://species.wikimedia.org/wiki/Swertia_perennis_subsp._perennis

https://europlusmed.org/cdm_dataportal/taxon/8849d45a-513e-4769-be0e-172d4a9dc41f

https://www.worldplants.de/world-plants-complete-list/complete-plant-list/?name=Swertia-perennis

≡ Swertiaperennissubsp.punctata (Baumg.) Ciocârlan, Fl. Ilustr. Rom. Vol. 2: 104 (1990), non S.dichotomavar.punctata T.N.Ho & J.X.Yang; POWO: https://powo.science.kew.org/taxon/3007716-4
= *Swertiaperennis* M.Bieb. ex Boiss., Fl. Orient. [Boissier] 4(1): 78 (1879), non L.; GBIF: https://www.gbif.org/species/8516331; GBIF: https://www.gbif.org/species/7945478; IPNI: https://ipni.org/n/371025-1; BHL: https://www.biodiversitylibrary.org/page/18114750#page/86
= *Swertiastigmantha* K.Koch, Linnaea 23: 586 (1850); GBIF: https://www.gbif.org/species/5595375; IPNI: https://ipni.org/n/371096-1; WFO: http://www.worldfloraonline.org/taxon/wfo-0000497939; POWO: https://powo.science.kew.org/taxon/371096-1; BHL: https://www.biodiversitylibrary.org/item/109824#page/594
– *Swertiaperennis* L., Sp. Pl. 1: 226 (1753) [p. p. minor]; CoL: https://www.catalogueoflife.org/data/taxon/9KSFD; GBIF: https://www.gbif.org/species/7270211; IPNI: https://ipni.org/n/371026-1; WFO: http://www.worldfloraonline.org/taxon/wfo-0000498075; POWO: https://powo.science.kew.org/taxon/371026-1; BHL: https://www.biodiversitylibrary.org/page/358245#page/238

##### Conservation status

In Ukraine – EN (Onyshchenko et al. 2022).

##### Distribution

SE Carpathian endemic.

##### Notes

GBIF (https://www.gbif.org/species/5595494, accessed on 06.06.2023), POWO (https://powo.science.kew.org/taxon/371096-1, accessed on 06.06.2023) and WFO (https://list.worldfloraonline.org/wfo-0000498017, accessed on 06.06.2023) provide *S.stigmantha* K.Koch amongst the synonyms of *S.punctata*. However, *S.stigmantha* has been described from Kazbek Mt. in the Caucasus and in its protologue, it was indicated that this species is just similar to *S.perennis* L. and *S.punctata* (Koch 1850: pp. 586–587). Perhaps, due to the occasional synonymisation of *S.stigmantha* and *S.punctata* within the framework of *S.perennis*, these two species were considered as direct synonyms. However, they are not – *S.punctata* occurs in the Carpathians and has only a few confirmed localities outside these mountains – in Bulgaria and Kosovo (Tan and Vladimirov 2001, Anchev et al. 2009, Kliment et al. 2016).

Boissier (1879), on page 78, mentioned *S.punctata* for Hungarian Mts. and Transylvania and provided *S . perennis* in the sense of M.Bieb., non L. amongst its synonyms. However, for some reason, he also included the Caucasian plants *S.iberica* Fisch. ex C.A.Mey. and S.obtusavar.albiflora Ledeb. in *S.punctata*.

#### Rubiaceae

C91A0FD6-89BA-5F3C-A487-F01EBCF45643

#### Galium album suberectum

(Klokov) Michálk., Karpatskaja Fl.: 78 (1988) et Biología, Bot. (Czechoslovakia) 48(1): 48 (1993)

35E34EB3-E8C5-5264-BCBA-4044C28FF329

https://www.catalogueoflife.org/data/taxon/7JSZR

https://www.gbif.org/species/2915138

urn:lsid:ipni.org:names:975731-1

http://www.worldfloraonline.org/taxon/wfo-0000968241

http://www.worldfloraonline.org/taxon/wfo-0001257913

https://powo.science.kew.org/taxon/975731-1

https://europlusmed.org/cdm_dataportal/taxon/e9e55f52-c0a6-4ff1-bfc2-850fc687fe7c

https://www.worldplants.de/world-plants-complete-list/complete-plant-list/?name=Galium-album

≡ Galiumerectumsubsp.suberectum (Klokov) Kobiv et al., Visnyk Lviv Univ., Ser. Biol. 49: 68 (2009) [nom. illeg.]
≡ *Galiumsuberectum* Klokov, Fl. UkrSSR 10: 463 (1961) *; GBIF: https://www.gbif.org/species/2915139; IPNI: https://ipni.org/n/750685-1; WFO: http://www.worldfloraonline.org/taxon/wfo-0000970278; POWO: https://powo.science.kew.org/taxon/750685-1
= Galiummollugosubsp.erectumf.longifolium Kucowa in Pawł., Fl. Polska 11: 311, 324 (1967)
– *Galiumerectum* auct. fl. ucrain. carpat., non Huds.
– Galiummollugosubsp.erectum (Huds.) Syme sensu Kucowa in Pawł., Fl. Polska 11: 311, 324 (1967) [p. p.]

##### Conservation status

In Ukraine – LC (Onyshchenko et al. 2022).

##### Distribution

SE Carpathian endemic.

#### Galium transcarpaticum

Stojko et Tasenk., Ukr. Bot. J. 36(6): 594 (1979)

DB4983DA-7B4B-5650-9020-F2521EE40E36

https://www.catalogueoflife.org/data/taxon/3F6MG

https://www.gbif.org/species/2914196

urn:lsid:ipni.org:names:750754-1

http://www.worldfloraonline.org/taxon/wfo-0000970423

https://powo.science.kew.org/taxon/750754-1

https://europlusmed.org/cdm_dataportal/taxon/d23eb453-2c4e-44b0-88b7-a1017ad5b096

https://www.worldplants.de/world-plants-complete-list/complete-plant-list/?name=Galium-transcarpaticum

https://www.jacq.org/detail.php?ID=406667

https://plants.jstor.org/stable/10.5555/al.ap.specimen.lws0017273

##### Conservation status

In Ukraine – EN (Onyshchenko et al. 2022).

##### Distribution

SE Carpathian endemic.

##### Notes

Controversial species (probably a local morphotype of Galiumalbumsubsp.suberectum) with an unclear systematic position (Stojko and Tasenkevich 1979). It was excluded from the new edition of the Flora of the Ukrainian Carpathians (Chopyk and Fedoronchuk 2015) due to its ambiguous delimiting morphological features and almost total absence of specimens identified as *G.transcarpaticum* by other researchers besides Tasenkevich. However, it was accepted by Mosyakin and Fedoronchuk (1999) and still listed as an endangered species by Onyshchenko et al. (2022) due to the absence of any further investigations on this species. Only for this reason, despite solid doubts, I retained this species as valid in the current list.

#### Lamiales

F5C3D9DD-B001-5C87-B371-8E0E97AAAB1D

#### Lamiaceae

FCD92A74-AC37-5138-BBEB-8D08025109CD

#### Thymus alternans

Klokov, Bot. Mater. Gerb. Bot. Inst. Komarova Akad. Nauk SSSR 16: 293 (1954)

F788E7CD-3A05-5852-A56C-DC23F9218BCE

https://www.catalogueoflife.org/data/taxon/56QNV

https://www.gbif.org/species/5607756

urn:lsid:ipni.org:names:460875-1

http://www.worldfloraonline.org/taxon/wfo-0000323669

https://powo.science.kew.org/taxon/460875-1

https://europlusmed.org/cdm_dataportal/taxon/d4cedbae-5fc0-4f8a-b5cc-deeb11ca2ad4

https://www.worldplants.de/world-plants-complete-list/complete-plant-list/?name=Thymus-alternans

– *Thymusmarschallianus* auct., non Willd. *
– *Thymusglabrescens* auct., non Willd.
– Thymusserpyllumf.margittaianus auct., non Lyka in Jav.
– *Thymusroegneri* K.Koch, Linnaea 21(6): 666 (1849) [p. p., tantum quod plantas ucrain. carpat.] *; GBIF: https://www.gbif.org/species/5605565; IPNI: https://ipni.org/n/461608-1; WFO: http://www.worldfloraonline.org/taxon/wfo-0000324688; POWO: https://powo.science.kew.org/taxon/461608-1; BHL: https://www.biodiversitylibrary.org/page/110603#page/669
– Thymusserpyllumvar.roegneri (K.Koch) Nyman, Consp. Fl. Eur. Suppl. 2: 257 (1890) [p. p., tantum quod plantas ucrain. carpat.]; IPNI: https://ipni.org/n/77293372-1; WFO: http://www.worldfloraonline.org/taxon/wfo-0000803343; POWO: https://powo.science.kew.org/taxon/77293372-1; BHL: https://www.biodiversitylibrary.org/page/11015401#page/268

##### Conservation status

In Ukraine – LC (Onyshchenko et al. 2022).

##### Distribution

Pancarpathian subendemic.

##### Notes

Euro+Med (https://europlusmed.org/cdm_dataportal/taxon/d4cedbae-5fc0-4f8a-b5cc-deeb11ca2ad4, accessed on 06.06.2023) and Worldplants (https://www.worldplants.de/world-plants-complete-list/complete-plant-list?name=Thymus-roegneri, accessed on 06.06.2023) consider *T.alternans* a synonym of *T.roegneri* K.Koch, which is widely distributed. However, Kliment et al. (2016) suggest it to be a valid subendemic species. Mártonfi (1996), Nachychko (2014) and Nachychko and Honcharenko (2017) also support the independence of *T.alternans*. Nevertheless, *T.alternans* plants from the Ukrainian Carpathians were sometimes misidentified as *T.roegneri*.

#### Thymus pulcherrimus pulcherrimus

Schur, Verh. Mitth. Siebenbürg. Vereins Naturwiss. Hermannstadt 10: 140 (1859) et Enum. Pl. Transssilv.: 526 (1866)

5C904E4F-7DA2-578A-80DF-05B2FBD16E8A

https://www.catalogueoflife.org/data/taxon/56RBT

https://www.gbif.org/species/7306766

urn:lsid:ipni.org:names:461561-1

http://www.worldfloraonline.org/taxon/wfo-0000324604

https://powo.science.kew.org/taxon/461561-1

https://species.wikimedia.org/wiki/Thymus_pulcherrimus

https://europlusmed.org/cdm_dataportal/taxon/633a82db-648d-4296-8a92-d38b5b1d68af

https://www.worldplants.de/world-plants-complete-list/complete-plant-list/?name=Thymus-pulcherrimus

https://www.biodiversitylibrary.org/page/11528081#page/384

≡ Thymuschamaedryssubsp.pulcherrimus (Schur) Simonk., Enum. Fl. Transsilv.: 442 (1886); GBIF: https://www.gbif.org/species/9273406; IPNI: https://ipni.org/n/77257228-1; WFO: http://www.worldfloraonline.org/taxon/wfo-1200012678; POWO: https://powo.science.kew.org/taxon/77257228-1; BHL: https://www.biodiversitylibrary.org/page/10524577#page/506
≡ *Thymuspulcherrimus* Schur, Verh. Mitth. Siebenbürg. Vereins Naturwiss. Hermannstadt 10: 140 (1859) [s. str.] *; CoL: https://www.catalogueoflife.org/data/taxon/56RBT; GBIF: https://www.gbif.org/species/7306766; IPNI: https://ipni.org/n/461561-1; WFO: http://www.worldfloraonline.org/taxon/wfo-0000324604; POWO: https://powo.science.kew.org/taxon/461561-1; BHL: https://www.biodiversitylibrary.org/page/11528081#page/384
≡ Thymusserpyllumsubsp.pulcherrimus (Schur) Lyka in Jáv., Magyar Fl.: 902 (1925); GBIF: https://www.gbif.org/species/7921817; IPNI: https://ipni.org/n/77257325-1; WFO: http://www.worldfloraonline.org/taxon/wfo-0000813146; POWO: https://powo.science.kew.org/taxon/77257325-1
≡ Thymusserpyllumvar.pulcherrimus (Schur) Nyman, Consp. Fl. Eur., Suppl. 2: 257 (1890); GBIF: https://www.gbif.org/species/8243035; IPNI: https://ipni.org/n/77293914-1; WFO: http://www.worldfloraonline.org/taxon/wfo-0000803341; POWO: https://powo.science.kew.org/taxon/77293914-1; BHL: https://www.biodiversitylibrary.org/page/11015401#page/268
= *Thymusrotundifolius* Schur, Verh. Mitth. Siebenbürg. Vereins Naturwiss. Hermannstadt 1: 108 (1850), non alior; GBIF: https://www.gbif.org/species/8345586; IPNI: https://ipni.org/n/461614-1; WFO: http://www.worldfloraonline.org/taxon/wfo-0000324696; POWO: https://powo.science.kew.org/taxon/461614-1; BHL: https://www.biodiversitylibrary.org/page/11525300#page/118
= Thymusserpyllumf.oreades Lyka ex Jáv., Magyar Fl.: 902 (1925); GBIF: https://www.gbif.org/species/8043403; WFO: http://www.worldfloraonline.org/taxon/wfo-0000813147; POWO: https://powo.science.kew.org/taxon/351274-4
= Thymuspulcherrimusvar.oreades (Lyka) Borza, Consp. Fl. Rom.: 233 (1947)
= Thymuspulcherrimusf.oreades (Lyka) Guşul. in Săvul., Fl. Rep. Pop. Rom. 8: 330 (1961)
= Thymuspulcherrimusf.beldiei Guşul. in Săvul., Fl. Rep. Pop. Rom. 8: 689 (1961) [nom. invalid.]
= *Thymuscircumcinctus* Klokov, Bot. Mater. Gerb. Bot. Inst. Komarova Akad. Nauk SSSR 16: 294 (1954) *; GBIF: https://www.gbif.org/species/5607459; IPNI: https://ipni.org/n/461039-1; WFO: http://www.worldfloraonline.org/taxon/wfo-0000323880; POWO: https://powo.science.kew.org/taxon/461039-1
– *Thymuscarpathicus* auct fl. ucrain. carpat., non Čelak. *
– *Thymusmontanus* auct., non Waldst. & Kit.
– *Thymussudeticus* Opiz ex Rchb., Fl. Germ. Excurs.: 312 (1830–1832) et Opiz ex Borbás, Math. Term. Közlem. 24(2): 103 (1890) [p. p., tantum quod plantas ucrain. carpat.] *; GBIF: https://www.gbif.org/species/5605220; GBIF: https://www.gbif.org/species/8040934; GBIF: https://www.gbif.org/species/8594373; IPNI: https://ipni.org/n/461700-1; WFO: http://www.worldfloraonline.org/taxon/wfo-0000324862; POWO: https://powo.science.kew.org/taxon/461701-1; BHL: https://www.biodiversitylibrary.org/page/6026374#page/385

##### Conservation status

In Ukraine – LC (Onyshchenko et al. 2022).

##### Distribution

Pancarpathian endemic.

##### Notes

Mártonfi (1997) delimited T.pulcherimussubsp.carpaticus(=subsp.sudeticus (Lyka) P.A.Schmidt) distributed in the western Carpathians and Sudetes from the eastern Carpathian subspecies T.pulcherimussubsp.pulcherimus (Mártonfi and Marhold 1998, Štěpánek and Tomšovic 2000). Amongst synonyms of T.pulcherimussubsp.carpaticus, Mártonfi (1997) surprisingly indicated *T.circumcinctus* Klokov, which has been described from the eastern Carpathians (Klokov 1960: pp. 301–302). However, in the following paper (Mártonfi and Marhold 1998), this confusing synonym and some other synonyms have been excluded.

#### Oleaceae

6C6A7332-368C-56DF-B73A-B5E5A582F0D1

#### Syringa josikaea

J.Jacq. ex Rchb.f., Iconogr. Bot. Pl. Crit. 8: 32 (1830) et J.Jacq., Flora 14(1): 67, 399 (1831)

CCC359D9-B7F5-5927-892E-ED543D718EC9

https://www.catalogueoflife.org/data/taxon/8X5YV

https://www.gbif.org/species/7636833

https://www.gbif.org/species/5549698

urn:lsid:ipni.org:names:60466522-2

urn:lsid:ipni.org:names:611121-1

http://www.worldfloraonline.org/taxon/wfo-0000818449

https://powo.science.kew.org/taxon/60466522-2?_gl=1

https://species.wikimedia.org/wiki/Syringa_josikaea

https://europlusmed.org/cdm_dataportal/taxon/b96456c9-76ec-444e-a8ff-6e36428ecf45

https://www.worldplants.de/world-plants-complete-list/complete-plant-list/?name=Syringa-josikaea

https://www.biodiversitylibrary.org/page/27340#page/72

= Syringahenryivar.eximia Rehder, Mitt. Deutsch. Dendrol. Ges. 24: 227 (1915); GBIF: https://www.gbif.org/species/7903992; WFO: http://www.worldfloraonline.org/taxon/wfo-0000821332; POWO: https://powo.science.kew.org/taxon/359007-4; BHL: https://www.biodiversitylibrary.org/item/49730#page/257
= Syringajosikaeavar.eximia Froebel ex Olbrich, Möller’s Deutsche Gärtn.-Zeitung 16: 561 (1901); GBIF: https://www.gbif.org/species/12061256; IPNI: https://www.ipni.org/n/77293238-1; WFO: http://www.worldfloraonline.org/taxon/wfo-1200020780; POWO: https://powo.science.kew.org/taxon/77293238-1
= *Syringajosikaea* [unranked] *eximia* hort. ex Beissner, Schelle & Zabel, Handb. Laubholzben.: 415 (1903); GBIF: https://www.gbif.org/species/11982022; POWO: https://powo.science.kew.org/taxon/495110-4
= Syringajosikaeaf.monstrosa Jägger ex Morariu, Fl. Rep. Pop. Rom. 8: 513 (1961)
= Syringajosikaeaf.pallida Jägger ex Morariu, Fl. Rep. Pop. Rom. 8: 513 (1961)
= *Syringajosikaea* [unranked] *pallida* hort. ex Beissner, Schelle & Zabel, Handb. Laubholzben.: 415 (1903)
= Syringajosikaeaf.rosea Miemetz ex Morariu, Fl. Rep. Pop. Rom. 8: 513 (1961)
= Syringajosikaeaf.rubra hort. ex Morariu, Fl. Rep. Pop. Rom. 8: 513 (1961)
= *Syringajosikaea* [unranked] *rubra* hort. ex Beissner, Schelle & Zabel, Handb. Laubholzben.: 415 (1903)
= Syringajosikaeaf.simia Froebel ex Morariu, Fl. Rep. Pop. Rom. 8: 513 (1961)
= Syringajosikaeaf.zabelii Froebel ex Morariu, Fl. Rep. Pop. Rom. 8: 513 (1961)
= *Syringajosikaea* [unranked] *zabeli* hort. ex Beissner, Schelle & Zabel, Handb. Laubholzben.: 415 (1903); GBIF: https://www.gbif.org/species/7596635; IPNI: https://www.ipni.org/n/77293646-1; WFO: http://www.worldfloraonline.org/taxon/wfo-0000821333; POWO: https://powo.science.kew.org/taxon/77293646-1
= *Syringaprunifolia* Kit. ex Lingelsh., Pflanzenr. [Engler] 72: 78 (1920); GBIF: https://www.gbif.org/species/5549631; IPNI: https://www.ipni.org/n/611152-1; WFO: http://www.worldfloraonline.org/taxon/wfo-0001354634; BHL: https://www.biodiversitylibrary.org/item/61953#page/84
= *Syringavincetoxifolia* Baumg. ex Steud., Nomencl. Bot., ed. 2 2: 656 (1841); GBIF: https://www.gbif.org/species/8462335; GBIF: https://www.gbif.org/species/5549581; IPNI: https://www.ipni.org/n/611183-1; WFO: http://www.worldfloraonline.org/taxon/wfo-0000818702; POWO: https://powo.science.kew.org/taxon/611183-1; BHL: https://www.biodiversitylibrary.org/page/392733#page/1512

##### Conservation status

In Ukraine – VU (Onyshchenko et al. 2022). Global – EN (Höhn and Lendvay 2018).

##### Distribution

SE Carpathian endemic.

##### Notes

This vulnerable species is listed by the Red Book of Ukraine (Mygal et al. 2009, MEPNR of Ukraine 2021) and by IUCN Red List (Höhn and Lendvay 2018).

Vasiliev (1952) and Macalik et al. (2013) mentioned *S.prunifolia* as a synonym of *S.josikaea*, but provided incorrect taxonomic authorship Kit. in Sched. ex Borbás, while the proper authorship is Kit. ex Lingelsh. (https://www.ipni.org/n/611152-1, accessed on 07.07.2023).

#### Orobanchaceae

7DA4D268-DD08-581C-BB71-1967D24BA9E6

#### Euphrasia tatrae

Wettst., Oesterr. Bot. Z. 44: 248 (1894)

680B0EC1-6BB4-5197-AFCF-9DCC1E655132

https://www.catalogueoflife.org/data/taxon/3CS5Z

https://www.gbif.org/species/3736062

urn:lsid:ipni.org:names:802898-1

http://www.worldfloraonline.org/taxon/wfo-0000682687

https://powo.science.kew.org/taxon/802898-1

https://europlusmed.org/cdm_dataportal/taxon/3f527fb0-810c-433f-a045-dbc651817be3

https://www.worldplants.de/world-plants-complete-list/complete-plant-list/?name=Euphrasia-tatrae

https://www.biodiversitylibrary.org/openurlmultiple.aspx?id=p28757191|p8735912

https://kfta.jacq.org/KFTA0001455

https://wu.jacq.org/WU0037599

https://wu.jacq.org/WU0037600

https://wu.jacq.org/WU0037601

https://wu.jacq.org/WU0037602

https://plants.jstor.org/stable/10.5555/al.ap.specimen.mpu020694

https://plants.jstor.org/stable/10.5555/al.ap.specimen.g00356782

https://plants.jstor.org/stable/10.5555/al.ap.specimen.mpu020693

https://plants.jstor.org/stable/10.5555/al.ap.specimen.kfta0001455

≡ Euphrasiaminimasubsp.tatrae (Wettst.) Hayek in Hegi, Ill. Fl. Mitteleur. 6(1): 91 (1913) *; GBIF: https://www.gbif.org/species/7531784; WFO: http://www.worldfloraonline.org/taxon/wfo-0001138644; POWO: https://powo.science.kew.org/taxon/2900710-4; JACQ: https://je.jacq.org/JE00017586; JACQ: https://je.jacq.org/JE00017587; JACQ: https://je.jacq.org/JE00017588; JACQ: https://je.jacq.org/JE00017589; JACQ: https://je.jacq.org/JE00017590; JACQ: https://je.jacq.org/JE00017591; JACQ: https://je.jacq.org/JE00017592; JSTOR Global Plants: https://plants.jstor.org/stable/10.5555/al.ap.specimen.je00017590; JSTOR Global Plants: https://plants.jstor.org/stable/10.5555/al.ap.specimen.je00017592; JSTOR Global Plants: https://plants.jstor.org/stable/10.5555/al.ap.specimen.je00017589; JSTOR Global Plants: https://plants.jstor.org/stable/10.5555/al.ap.specimen.je00017588; JSTOR Global Plants: https://plants.jstor.org/stable/10.5555/al.ap.specimen.je00017591; JSTOR Global Plants: https://plants.jstor.org/stable/10.5555/al.ap.specimen.je00017586; JSTOR Global Plants: https://plants.jstor.org/stable/10.5555/al.ap.specimen.je00017587
≡ Euphrasiaminimavar.tatrae (Wettst.) Pawł., Fl. Polska 11: 17 (1967)
= Euphrasiaminimavar.carpathica Freyn in Sagorski & Schneider, Fl. Centralkarpat. 2: 421 (1891) non *Euphrasiacarpatica* Zapał.; POWO: https://powo.science.kew.org/taxon/2936289-4; JACQ: https://wu.jacq.org/WU0037573
= Euphrasiaminimavar.tatraef.glandulifera (Wettst.) Răvăruţ, Fl. Rep. Pop. Rom. 7: 586 (1960)
= *Euphrasiaofficinalis* [unranked] δ *alpestris* Freyn, Verh. K.K. Zool.-Bot. Ges. Wien 22: 350 (1872), non Günther, Grab. & Wimm.; BHL: https://www.biodiversitylibrary.org/item/136833#page/494
= Euphrasiatatraesubsp.glandulifera (Wettst.) Staszk., Fragm. Florist. Geobot. 22(2): 292 (2015); GBIF: https://www.gbif.org/species/8923251; IPNI: https://www.ipni.org/n/77154667-1; WFO: http://www.worldfloraonline.org/taxon/wfo-0001368423; POWO: https://powo.science.kew.org/taxon/77154667-1
= Euphrasiatatraef.glandulifera Wettst., Monogr. Gatt. Euphrasia: 165 (1896)

##### Conservation status

In Ukraine – LC (Onyshchenko et al. 2022).

##### Distribution

Pancarpathian subendemic.

##### Notes

Wettstein, who described *E.tatrae* in 1894, later distinguished E.tatraef.glandulifera Wettst. by the presence of glandular trichomes (Wettstein 1894, Wettstein 1896). However, he noted that plants with glandular and eglandular trichomes co-occur and can probably hybridise. Staszkiewicz (2015) raised this form to rank of subspecies and delimited subsp. tatrae and subsp. glandulifera (Wettst.) Staszk. Staszkiewicz (2015) also noted that E.tatraesubsp.glandulifera is a hybrid of *E.rostkoviana* Hayne and *E.nemorosa* (Pers.) Wallr. Euphrasiatatraesubsp.glandulifera is not mentioned for the flora of the Ukrainian Carpathians, but both mentioned parental species occur there and, therefore, the presence of their hybrid is highly possible. It is also worth noting that Mirek et al. (2020) consider *E.tatrae* as a synonym of *E.minima* Jacq. ex DC. At the same time, Tzvelev (1981) and Peregrym (2010) believed that *E.minima* and *E.tatrae* are two different species. He pointed out that *E.minima* occurs in more western areas of Europe and does not occur in the USSR (i.e. in the Ukrainian Carpathians), where it is displaced by *E.tatrae*. Hence, due to the absence of special morphological studies of *E.tatrae* in the Ukrainian Carpathians and its questionable taxonomy, here I am not delimiting the subspecies or forms within this species and consider E.tatraesubsp./f.glandulifera a synonym of *E.tatrae*.

#### Plantaginaceae

C3367436-B3DB-57EE-9926-5F9EEE405886

#### Plantago atrata carpatica

(Pilg.) Soó, Acta Geobot. Hung. 3: 61 (1940)

224B26CD-A3C1-5A54-BDFE-3EAD6F5CA555

https://www.catalogueoflife.org/data/taxon/5KH8H

https://www.gbif.org/species/7624228

urn:lsid:ipni.org:names:77252604-1

http://www.worldfloraonline.org/taxon/wfo-1200011825

https://powo.science.kew.org/taxon/77252604-1

https://europlusmed.org/cdm_dataportal/taxon/8cf71b0a-d70e-47e4-a6fc-328e266ce518

https://www.worldplants.de/world-plants-complete-list/complete-plant-list/?name=Plantago-atrata

≡ Plantagoatratasubsp.atratavar.carpathica (Pilg.) Pilg., Pflanzenr. (Engler) 102: 296 (1937); GBIF: https://www.gbif.org/species/8397828; BHL: https://www.biodiversitylibrary.org/item/71651#page/304
≡ Plantagomontanasubsp.atratavar.carpathica Pilg., Repert. Spec. Nov. Regni Veg. 23: 256 (1926–1927); GBIF: https://www.gbif.org/species/7564352; POWO: https://powo.science.kew.org/taxon/3007639-4
≡ Plantagomontanasubsp.carpatica (Pilg.) Soó ex Balázs, Acta Geobot. Hung. 2: 40 (1938–1939); POWO: https://powo.science.kew.org/taxon/3007638-4
= Plantagoatratasubsp.atratavar.carpathicasubvar.rigidior (Pilg.) Pilg., Pflanzenr. (Engler): 296 (1937); BHL: https://www.biodiversitylibrary.org/item/71651#page/304
= Plantagoatratasubsp.atratavar.carpathicasubvar.vestita (Pilg.) Pilg., Pflanzenr. (Engler): 296 (1937); BHL: https://www.biodiversitylibrary.org/item/71651#page/304
= Plantagoatratavar.carpathicaf.vestita (Pilg.) Borza, Consp. Fl. Rom.: 255 (1949)
= Plantagoatratasubsp.carpathicaf.vestita (Pilg.) Soó, Acta Geobot. Hung. 3: 61 (1940)
= *Plantagolanceolata* [unranked] β *alpestris* Wahlenb., Fl. Carpat. Princip.: 44 (1814)
= *Plantagomontana* [unranked] *alpestre* Wahlenb., Fl. Carpat. Princip.: 44 (1814)
= Plantagomontanasubsp.atratavar.carpathicasubvar.rigidior Pilg., Repert. Spec. Nov. Regni Veg. 23: 257 (1926–1927)
= Plantagomontanasubsp.atratavar.carpathicasubvar.vestita Pilg., Repert. Spec. Nov. Regni Veg. 23: 257 (1926–1927)
= Plantagomontanasubsp.carpaticasubvar.rigidior (Pilg.) Balázs, Acta Geobot. Hung. 2: 40 (1938–1939)
= Plantagomontanasubsp.carpaticasubvar.vestita (Pilg.) Balázs, Acta Geobot. Hung. 2: 40 (1938–1939)
– *Plantagoalpina* Vill. sensu Rochel, Pl. Banat. Rar.: 32; Nr. 4, Tab. 1, fig. 4 (1828)
– *Plantagoalpina* Vill. sensu Schur, Enum. Pl. Transsilv.: 564 (1866), non alior; BHL: https://www.biodiversitylibrary.org/item/7364#page/584
– *Plantagoatrata* Hoppe, Bot. Taschenb. 1799: 85 (1799) [p. p., tantum quod plantas ucrain. carpat.] *; GBIF: https://www.gbif.org/species/8083357; IPNI: https://www.ipni.org/n/684903-1; WFO: http://www.worldfloraonline.org/taxon/wfo-0000487646; POWO: powo.science.kew.org/taxon/684903-1; BHL: https://www.biodiversitylibrary.org/item/22931#page/95
– *Plantagomontana* Lam. sensu Schur, Enum. Pl. Transsilv.: 564 (1866), non alior *; BHL: https://www.biodiversitylibrary.org/item/7364#page/584
– *Plantagosaxatilis* M.Bieb., Fl. Taur.-Caucasus 1: 109 (1808) [p. p.]; GBIF: https://www.gbif.org/species/4156187; IPNI: https://www.ipni.org/n/685623-1; WFO: http://www.worldfloraonline.org/taxon/wfo-0000487134; POWO: https://powo.science.kew.org/taxon/685623-1; BHL: https://www.biodiversitylibrary.org/page/11268189#page/117

##### Conservation status

In Ukraine – LC (Onyshchenko et al. 2022).

##### Distribution

Pancarpathian endemic.

##### Notes

There are nine subspecies of *P.atrata* Hoppe, from which only P.atratasubsp.carpatica is usually reported for the Ukrainian Carpathians. However, Chrtek (2000) also delimited P.atratasubsp.ucrainica Chrtek that, as he indicated, mainly occurs in the Svydovets Mts. Besides this, he mentioned the presence of this subspecies in Romania (Slănic Moldova and Retezat). Plantagoatratasubsp.ucrainica differs by erect ascending (vs. decumbent to prostrate in P.atratasubsp.carpatica), longer (up to 17 cm long vs. 14 cm in P.atratasubsp.carpatica) and more narrow (up to 8 mm wide vs. 16 mm P.atratasubsp.carpatica) leaves. Distribution and phylogenetic position of P.atratasubsp.ucrainica still requires clarifications since, after Chrtek (2000), there were no further corresponding investigations on this subspecies.

The combination P.montanasubsp.carpatica Soó, Acta Geobot. Hung. 2: 40 (1938–1939) and consequent recombination P.atratasubsp.carpatica (Soó) Soó, Acta Geobot. Hung. 3: 61 (1940), commonly circulated in the checklists, seem to be incorrect because it was Pilger who first applied the epithet *carpathica* in the name P.montanasubsp.atratavar.carpathica Pilg. in 1926 (Pilger 1926). Later, in 1937, Pilger introduced a new combination P.atratasubsp.atratavar.carpathica (Pilg.) Pilg. (Pilger 1937). It looks like Soó made further taxonomic recombinations, based on these two Pilger’s names, but, unfortunately, I could not locate the original works of Soó regarding *P.atrata* to check.

GBIF (https://www.gbif.org/species/11030083, accessed on 06.06.2023) incorrectly provides the name P.atratasubsp.carpathica (Pilg.) Pilg. It should be either P.atratavar.carpathica (Pilg.) Pilg. (incorrect taxonomic rank is indicated) or P.atratasubsp.carpatica (Pilg.) Soó (the incorrect authorship is provided). In both cases, the entry is duplicating other existing records.

#### Scrophulariaceae

F9E9C8FB-262A-5E76-9B75-BA4FBD915E07

#### Melampyrum saxosum

Baumg., Enum. Stirp. Transsilv. 2: 199 (1816)

C2760214-A3FC-512F-B90F-1C3480BE8A48

https://www.catalogueoflife.org/data/taxon/6R9T5

https://www.gbif.org/species/3725032

urn:lsid:ipni.org:names:805724-1

http://www.worldfloraonline.org/taxon/wfo-0001138715

https://powo.science.kew.org/taxon/805724-1

https://europlusmed.org/cdm_dataportal/taxon/6a35a594-7f27-40ef-8379-37fa68e734fe

https://www.worldplants.de/world-plants-complete-list/complete-plant-list/?name=Melampyrum-saxosum

≡ *Melampyrumsylvaticum* [unranked] *M.saxosum* (Baumg.) Nyman, Consp. Fl. Eur.: 556 (1881); BHL: https://www.biodiversitylibrary.org/item/41446#page/567
≡ Melampyrumsylvaticumsubsp.saxosum (Baumg.) G.Beauvis., Bull. Soc. Bot. Geneve 4: 418 (1912) et Mem. Soc. Phys. Hist. Nat. Geneve 38(6): 581 (1916) *; BHL: https://www.biodiversitylibrary.org/item/27563#page/810; BHL: https://www.biodiversitylibrary.org/item/50058#page/665
= *Melampyrumherbichii* Woł., Spraw Kom. Fizyi. Krajow. 21: 133 (1888) *; CoL: https://www.catalogueoflife.org/data/taxon/3Z5BY; GBIF: https://www.gbif.org/species/7331709; IPNI: https://www.ipni.org/n/805671-1; WFO: http://www.worldfloraonline.org/taxon/wfo-0001138706; POWO: https://powo.science.kew.org/2507126-4
= Melampyrumherbichiisubsp.csatoi (Soó) Soó, Feddes Repert. 83(3): 181 (1972); GBIF: https://www.gbif.org/species/3725616; IPNI: https://www.ipni.org/n/891377-1; WFO: http://www.worldfloraonline.org/taxon/wfo-0001364943; POWO: https://powo.science.kew.org/taxon/891377-1
= Melampyrumherbichiisubsp.woloszczakii Jasiewicz, Fragm. Florist. Geobot. 4: 112 (1958); GBIF: https://www.gbif.org/species/7867330
= Melampyrumsaxosumsubsp.baumgartenii (Soó) Soó, Feddes Repert. 24: 176 (1927); GBIF: https://www.gbif.org/species/8046716; WFO: http://www.worldfloraonline.org/taxon/wfo-0000747163; POWO: https://powo.science.kew.org/taxon/2900697-4
= *Melampyrumsaxosum* [unranked] *baumgartenii* Soó ex Jáv., Magyar Fl.: 1011 (1925); GBIF: https://www.gbif.org/species/8117461; WFO: http://www.worldfloraonline.org/taxon/wfo-0000747164; POWO: https://powo.science.kew.org/taxon/2900698-4
= Melampyrumsaxosumvar.baumgartenii (Soó) Nyár., Flora Rep. Pop. Rom. 7: 637, 646 (1960)
= Melampyrumsaxosumsubsp.javorkae (Soó) Soó, Feddes Repert. 24: 176 (1927); GBIF: https://www.gbif.org/species/7459548; WFO: http://www.worldfloraonline.org/taxon/wfo-0000747162; POWO: https://powo.science.kew.org/taxon/2900696-4
= *Melampyrumsaxosum* [unranked] *javorkae* Soó ex Jáv., Magyar Fl.: 1011 (1925); GBIF: https://www.gbif.org/species/7653737; WFO: http://www.worldfloraonline.org/taxon/wfo-0000747165; POWO: https://powo.science.kew.org/taxon/2900699-4
= Melampyrumsaxosumvar.javorkae (Soó) Nyár., Flora Rep. Pop. Rom. 7: 637, 646 (1960)
= Melampyrumsaxosumvar.typicum Nyár., Flora Rep. Pop. Rom. 7: 637, 646 (1960)
= Melampyrumsylvaticumf.csatoi Soó, Feddes Repert. 24: 174 (1927); GBIF: https://www.gbif.org/species/8389454; POWO: https://powo.science.kew.org/taxon/2900694-4
= Melampyrumsylvaticumsubsp.moeszianum Soó, Feddes Repert. 24: 190 (1927); GBIF: https://www.gbif.org/species/7632053; POWO: https://powo.science.kew.org/taxon/2900695-4
= *Melampyrumsylvaticum* [unranked] α *pictum* Herbich, Select. Pl. Rar. Galic. Bucov.: Nr 39 (1836) et Fl. Bucov.: 275 (1859)
= Melampyrumsylvaticumvar. βsaxosum Willkomm, Führer Pfl. Deutsch., Österr. und Schweiz: 535 (1881)
= Melampyrumsylvaticumsubsp.saxosumvar.herbichii (Woł.) G.Beauvis., Mem. Soc. Phys. Hist. Nat. Geneve 38: 582 (1916)
= Melampyrumsylvaticumsubsp.saxosumvar. βpictum (Herbich) G.Beauvis., Mem. Soc. Phys. Hist. Nat. Geneve 38: 581 (1916); BHL: https://www.biodiversitylibrary.org/item/50058#page/665
= Melampyrumsylvaticumsubsp.saxosumvar.pictumsubvar.eu-pictum G.Beauvis., Mem. Soc. Phys. Hist. Nat. Geneve 38: 582 (1916); BHL: https://www.biodiversitylibrary.org/item/50058#page/666
= Melampyrumsylvaticumsubsp.saxosumvar.pictumsubvar.eu-saxosum G.Beauvis., Mem. Soc. Phys. Hist. Nat. Geneve 38: 582 (1916); BHL: https://www.biodiversitylibrary.org/item/50058#page/666
– *Melampyrumpictum* Herbich [nom inval., ex herb LWS] *
– *Melampyrumsylvaticum* Simonk., Enum. Fl. Transsilv.: 429 (1886) [p. p.], non L.; BHL: https://www.biodiversitylibrary.org/item/40105#page/493

##### Conservation status

In Ukraine – LC (Onyshchenko et al. 2022).

##### Distribution

SE Capathian endemic.

##### Notes

GBIF (https://www.gbif.org/species/7331709, accessed on 06.06.2023), CoL (https://www.catalogueoflife.org/data/taxon/3Z5BY, accessed on 06.06.2023), WFO (https://list.worldfloraonline.org/wfo-0001138706, accessed on 06.06.2023) and Euro+Med (https://europlusmed.org/cdm_dataportal/taxon/dd655aaa-26d0-4250-8474-fdb870a3cea8) consider *M.herbichii* Woł. an independent species. However, Štech and Drábková (2005) and Těšitel and Štech (2007) concluded that *M.herbichii* is morphologically identical to *M.saxosum* and differs only by perianth colouration. Later, Těšitel et al. (2009), based on comprehensive morphological and molecular analyses, confirmed that these two species are to be united.

#### Malpighiales

7FCF33E0-BD65-5478-A5FD-76DD729CC87E

#### Linaceae

6975270F-438C-51D1-9EBC-A13E793194B2

#### Linum extraaxillare

Kit. ex Rochel, Pl. Banat. Rar.: 26 (1828) [nom. nudum] et Kit., Linnaea 32(4-5): 573 (1864)

59225B6E-123D-5C8F-BD49-F7FFFBFAA2E2

https://www.gbif.org/species/4049149

urn:lsid:ipni.org:names:544466-1

http://www.worldfloraonline.org/taxon/wfo-0000363481

https://powo.science.kew.org/taxon/544466-1

https://species.wikimedia.org/wiki/Linum_extraaxillare

https://europlusmed.org/cdm_dataportal/taxon/2e6f70ed-2b39-4165-b65a-3fc35efc0590

https://www.worldplants.de/world-plants-complete-list/complete-plant-list/?name=Linum-perenne

https://www.biodiversitylibrary.org/page/118664#page/576

≡ Linumperennesubsp.extraaxillare (Kit. ex Rochel) Nyman, Consp. Fl. Eur., Suppl. 2: 71 (1889); CoL: https://www.catalogueoflife.org/data/taxon/7K9NB; GBIF: https://www.gbif.org/species/6711238; WFO: https://list.worldfloraonline.org/wfo-0000737557; POWO: https://powo.science.kew.org/taxon/2873434-4
– *Linummontanum* auct. fl. transsilv., non Schleich.
– *Linumalpinum* auct. fl. transsilv., non L.

##### Conservation status

In Ukraine – NT (Onyshchenko et al. 2022).

##### Distribution

Pancarpathian subendemic.

#### Salicaceae

BD0E4931-D0D7-529D-BBAD-AE4FB29A721B

#### Salix kitaibeliana

Willd., Sp. Pl., ed. 4 [Willdenow] 4(2): 683-684 (1806)

1F958268-A9F1-5A69-AA2B-15022B24C335

https://www.gbif.org/species/5583534

urn:lsid:ipni.org:names:777938-1

http://www.worldfloraonline.org/taxon/wfo-0000928719

https://powo.science.kew.org/taxon/777938-1

https://europlusmed.org/cdm_dataportal/taxon/fbc32a8d-dcc0-4db9-8ae3-d3cad32cc004

https://www.worldplants.de/world-plants-complete-list/complete-plant-list/?name=Salix-retusa

https://www.biodiversitylibrary.org/page/566410#page/52

≡ Salixretusasubsp.kitaibeliana (Willd.) Jáv., Magyar Fl.: 235 (1924) *; POWO: https://powo.science.kew.org/taxon/3007645-4
≡ Salixretusaf.kitaibeliana (Willd.) Rouy, Fl. France [Rouy & Foucaud] 12: 219 (1910) *; POWO: https://powo.science.kew.org/taxon/3253081-4
≡ *Salixretusa* [unranked] γ *kitaibeliana* (Willd.) Rchb., Reichenbachianae Fl. German.: 15 (1833) et Icon. Fl. Germ. Helv. 11: 16, fig. 1187 (1849); GBIF: https://www.gbif.org/species/9285964; BHL: https://www.biodiversitylibrary.org/page/5763497#page/22
= *Salixretusa* [unranked] b *serrulata* Roch., Pl. Banat.: 78, tab. 38, fig. 80 (1828)
= Salixretusavar.serrulata Roch. ex Rchb., Fl. Germ. Excurs.: 166 (1831); POWO: https://powo.science.kew.org/taxon/3235618-4; BHL: https://www.biodiversitylibrary.org/item/7359#page/239
= Salixretusavar.major Rchb., Fl. Germ. Excurs.: 166 (1830–1832); POWO: https://powo.science.kew.org/taxon/3235617-4; BHL: https://www.biodiversitylibrary.org/item/7359#page/239
= *Salixretusa* [unranked] β *major* W.D.J. Koch, Syn. Fl. Germ. Helv.: 660 (1837) [nom. superfl.]; GBIF: https://www.gbif.org/species/11966312; BHL: https://www.biodiversitylibrary.org/item/29532#page/724

##### Conservation status

In Ukraine – EN (Onyshchenko et al. 2022).

##### Distribution

Pancarpathian endemic.

##### Notes

A rare species listed by the Red Book of Ukraine (Danylyk 2009, MEPNR of Ukraine 2021) with an unclear taxonomic position.

In all databases that were accessed on 06.06.2023, including CoL (https://www.catalogueoflife.org/data/taxon/6XDTN), GBIF (https://www.gbif.org/species/8119241), POWO (https://powo.science.kew.org/taxon/778676-1), Euro+Med (https://europlusmed.org/cdm_dataportal/taxon/fbc32a8d-dcc0-4db9-8ae3-d3cad32cc004) and Worldplants (https://www.worldplants.de/world-plants-complete-list/complete-plant-list/?name=Salix-retusa), *S.kitaibeliana* is provided as a synonym for *S.retusa* L., a Paneuropean mountainous species. Similarly, it is synonymised with *S.retusa* by many Ukrainian authors (e.g. Mosyakin and Fedoronchuk (1999), Danylyk (2009), Chorney (2011), Ishchuk (2017)). It is also synonymised by Kucowa (1954) and Mirek et al. (2020). However, Kliment et al. (2016), like some other authors (e.g. Piscová et al. (2021)), consider *S.kitaibeliana* as an independent species. Chopyk and Fedoronchuk (2015) noted that these two species are very close, but also still delimited them, based on the differences in the leaf morphology (leaves are up to 2 cm long obovate, with a retuse tip in *S.retusa* and up to 4 cm long, oblong-obovate, with a pointed tip in *S.kitaibeliana*). The same differences in the leaf morphology applied to delimit *S.kitaibeliana* and *S.retusa* in the Flora of Romania (Beldie 1952), where they are, however, provided in the rank of varieties. *Salixretusa* s. str. is considered in the Flora of Romania as S.retusavar.genuina Rchb. and *S.kitaibeliana* – as S.retusavar.kitaibeliana (Willd.) Rchb. Additionally, Beldie (1952) mentioned differences in their habitus (short creeping stems and branches in *S.retusa* and firm and sometimes ascending stems in *S.kitaibeliana*). The difference in the leaf morphology of these two species was statistically confirmed by Kosiński and Adreas Hilpold (2017). However, later phylogenetic studies (Kosiński et al. 2019) regarding ploidy did not allow delimiting *S.kitaibeliana*.

It is worth noting that Pawłowski (1946) also recognised *S.retusa* and *S.kitaibeliana* separately. He pointed out that, despite these two species often co-occurring, *S.retusa* prefers lime substrates while *S.kitaibeliana* mainly grows on granite outcrops and rocks. Myklestad and Birks (1993) partially confirmed such ecological differentiation of these two species in their ecogeographical studies – on the provided graphs, *S.kitaibeliana* is well separated from *S.retusa*.

#### Violaceae

96F5CC7F-03B1-5681-9922-C104F1781B5C

#### Viola declinata

Waldst. et Kit., Descr. Icon. Pl. Rar. Hung. 3: 248 (1807)

1792E949-8F6E-5CE7-A6DB-743B36EC72C6

https://www.catalogueoflife.org/data/taxon/5BGMT

https://www.gbif.org/species/5664655

urn:lsid:ipni.org:names:868009-1

http://www.worldfloraonline.org/taxon/wfo-0000424016

https://powo.science.kew.org/taxon/868009-1

https://species.wikimedia.org/wiki/Viola_declinata

https://europlusmed.org/cdm_dataportal/taxon/02a632cf-6c07-4887-adef-70bd54e7d0ec

https://www.worldplants.de/world-plants-complete-list/complete-plant-list/?name=Viola-declinata

https://w.jacq.org/W0020444

https://plants.jstor.org/stable/10.5555/al.ap.specimen.m0112758

= Violadeclinatavar.knechtelii Grec., Consp. Fl. Rom.: 88 (1898)
= Violadeclinatavar.major (Roch.) Grec., Consp. Fl. Rom.: 88 (1898)
= *Violadeclinata* [unranked] b *montana* Schur, Enum. Pl. Transsilv.: 86 (1866); BHL: https://www.biodiversitylibrary.org/item/7364#page/106
= *Violagracilis* Rchb., Fl. Germ. Excurs. 709 (1832), non alior; GBIF: https://www.gbif.org/species/7951755; IPNI: https://www.ipni.org/n/868244-1; BHL: https://www.biodiversitylibrary.org/page/6164133#page/276
= *Violamutabilis* [unranked] b *intermedia* Roch., Enum. Pl. Banat.: 6 (1828) [nom. nudum]
= *Violamutabilis* [unranked] e *major* Roch., Enum. Pl. Banat.: 6 (1828) [nom. nudum]

##### Conservation status

In Ukraine – LC (Onyshchenko et al. 2022).

##### Distribution

SE Capathian endemic.

##### Notes

*Violadeclinata* is often considered a Carpatho-Balkanic species (Kricsfalusy and Budnikov 2002, Oprea 2005, Ciocârlan 2009). However, Velev and Apostolova (2009) reported that it does not occur in Serbia and Bulgaria, as suggested before. Considering the questionable presence of *V.declinata* in the Balkans (perhaps it is introduced), Chorney (2011) and Kliment et al. (2016) considered it a Carpathian endemic.

POWO (https://powo.science.kew.org/taxon/868009-1, accessed on 06.06.2023) erroneously indicates *V.latisepala* Wettst. amongst synonyms of *V.declinata. Violalatisepala* (= *V.elegantula subsp. latisepala* (Wettst.) W.Becker) is a problematic taxon, which is often considered a synonym for *V.elegantula*, a Balkan endemic (Valentine et al. 1968, Tomović et al. 2016). Currently *V.latisepala* is considered as a synonym for *V.tricolor* L. (Marcussen et al. 2022). Moreover, there are some other species (e.g. *V.aetolica* Boiss. & Heldr. and *V.dacica* Borbás) that are occasionally misidentified as *V.L.tisepala.* (Tomović et al. 2014).

#### Ranunculales

8CA65C83-E9F0-590D-985E-68604F665D89

#### Ranunculaceae

CB351E34-ADA0-5AE1-8703-987D8C919D28

#### Aconitum bucovinense

Zapał., Rozpr. Wydz. Mat.-Przyr. Akad. Umiej., Dział B. Nauki Biol. 48: 8990 (1908)

A247946B-6F5A-52D6-A96D-6CE94417A95E

https://www.catalogueoflife.org/data/taxon/8S2V7

https://www.gbif.org/species/3926782

urn:lsid:ipni.org:names:707221-1

http://www.worldfloraonline.org/taxon/wfo-0000517006

https://powo.science.kew.org/taxon/707221-1

https://europlusmed.org/cdm_dataportal/taxon/3e3c3e87-e094-4e54-ae24-fef1882829ed

https://www.worldplants.de/world-plants-complete-list/complete-plant-list/?name=Aconitum-bucovinense

≡ Aconitumcallibotryonsubsp.bucovinense (Zapał.) Grinţ., Fl. Rep. Pop. Rom. 2: 481 (1953); GBIF: https://www.gbif.org/species/8002043; WFO: https://list.worldfloraonline.org/wfo-0000517021; POWO: https://powo.science.kew.org/taxon/2618479-4
≡ Aconitumfirmumsubsp.bucovinense (Zapał.) Aschers. & Graebn., Syn. Mitteleurop. Fl. 5/2: 781 (1929); GBIF: https://www.gbif.org/species/8007137; WFO: https://list.worldfloraonline.org/wfo-0000517250; POWO: https://powo.science.kew.org/taxon/2618700-4
= Aconitumbucovinensef.orthotricha Gáyer, Magyar Bot. Lap. 8: 168 (1909); GBIF: https://www.gbif.org/species/12044665; POWO: https://powo.science.kew.org/taxon/3284484-4; BHL: https://www.biodiversitylibrary.org/item/201903#page/194
= Aconitumcallibotryonsubsp.bucovinensef.altum Grinţ., Fl. Rep. Pop. Rom. 2: 482, 685 (1953)
= Aconitumcallibotryonsubsp.bucovinensef.densum Grinţ., Fl. Rep. Pop. Rom. 2: 482, 685 (1953)
= Aconitumcallibotryonsubsp.bucovinensef.glaberrimum Grinţ., Fl. Rep. Pop. Rom. 2: 482, 684 (1953)
= Aconitumcallibotryonsubsp.bucovinensef.laxum Grinţ., Fl. Rep. Pop. Rom. 2: 482, 685 (1953)
= Aconitumcallibotryonsubsp.bucovinensef.pilosum Grinţ., Fl. Rep. Pop. Rom. 2: 482, 684 (1953)
= Aconitumcallibotryonsubsp.bucovinensef.pyramidatum Grinţ., Fl. Rep. Pop. Rom. 2: 482, 685 (1953)
= Aconitumcallibotryonsubsp.rigidum (Rchb.) Grinţ., Fl. Rep. Pop. Rom. 2: 482 (1953)
= Aconitumcallibotryonsubsp.rigidumf.glabrum Grinţ., Fl. Rep. Pop. Rom. 2: 482, 683 (1953)
= Aconitumcallibotryonsubsp.rigidumf.pubescens Grinţ., Fl. Rep. Pop. Rom. 2: 482, 684 (1953); JACQ: https://ere.jacq.org/ERE0005605
= *Aconitumcommutatum* Rchb., Uebers. Aconitum: 36 (1819); GBIF: https://www.gbif.org/species/3926625; IPNI: https://www.ipni.org/n/707272-1; WFO: http://www.worldfloraonline.org/taxon/wfo-0000517107; POWO: https://powo.science.kew.org/taxon/707272-1
= Aconitumfirmumf.rigidum (Rchb.) Gáyer, Magyar Bot. Lapok 8: 165 (1909); BHL: https://www.biodiversitylibrary.org/item/201903#page/191
= *Aconitumlaetum* [unranked] β *rigidum* Rchb., Icon. Fl. Germ. Helv. 4: 25, tab. 97, fig. 4708b (1840); BHL: https://www.biodiversitylibrary.org/item/28665#page/127/
= Aconitumnapellusf.commutatum (Rchb.) Gáyer in G. Hegi, Ill. Fl. Mitt.-Eur. 3: 499 (1912); GBIF: https://www.gbif.org/species/12132273; POWO: https://powo.science.kew.org/taxon/2619134-4
– *Aconitumbernhardianum* Rchb., Uebers. Aconitum: 34 (1819) et Illustrat. Spec. Aconitum: tab. 68 (1823–1827), non Wallr.; GBIF: https://www.gbif.org/species/3926951; IPNI: https://www.ipni.org/n/707197-1; WFO: http://www.worldfloraonline.org/taxon/wfo-0000516975; POWO: https://powo.science.kew.org/taxon/707197-1

##### Conservation status

In Ukraine – EN (Onyshchenko et al. 2022).

##### Distribution

SE Carpathian endemic.

##### Notes

The nomenclature and synonymy of the genus *Aconitum* L. follow Mitka (2003), Mitka (2008) and Mitka et al. (2021) with my minor additions and some notes.

#### Aconitum firmum firmum

Rchb., Uebers. Aconitum: 20 (1819)

79940187-0E49-5AAD-871A-61A321108B99

https://www.catalogueoflife.org/data/taxon/5FDHQ

https://www.gbif.org/species/7277350

urn:lsid:ipni.org:names:707355-1

http://www.worldfloraonline.org/taxon/wfo-0000517247

https://powo.science.kew.org/taxon/51035059-1

https://species.wikimedia.org/wiki/Aconitum_firmum

https://europlusmed.org/cdm_dataportal/taxon/7f24502b-f9bb-438a-93e5-be837558865e

https://www.worldplants.de/world-plants-complete-list/complete-plant-list?name=Aconitum-firmum

https://plants.jstor.org/stable/10.5555/al.ap.specimen.bm000613691

https://plants.jstor.org/stable/10.5555/al.ap.specimen.bm000613690

https://plants.jstor.org/stable/10.5555/al.ap.specimen.bm000613679

≡ Aconitumkoelleanumvar.firmum (Rchb.) Rchb., Mon. Aconitum: 85, Tab. 14, fig. 1 (1821); GBIF: https://www.gbif.org/species/12133197; GBIF: https://www.gbif.org/species/8102235; WFO: https://list.worldfloraonline.org/wfo-0000517479; POWO: https://powo.science.kew.org/taxon/2618919-4
≡ Aconitumnapellussubsp.firmum (Rchb.) Gáyer in G.Hegi, Ill. Fl. Mitt.-Eur. 3: 498 (1912); GBIF: https://www.gbif.org/species/8325653; WFO: https://list.worldfloraonline.org/wfo-0000517706; POWO: https://powo.science.kew.org/taxon/2619142-4
≡ Aconitumnapellusvar.firmum (Rchb.) Pawł., FI. Tatr. 1: 274 (1956) *; GBIF: https://www.gbif.org/species/11952496; WFO: https://list.worldfloraonline.org/wfo-0000517707; POWO: https://powo.science.kew.org/taxon/2619143-4
= *Aconitumnapellus* [unranked] e *babiogorense* Zapał., Consp. FI. Gal. Crit. 2: 226 (1908); GBIF: https://www.gbif.org/species/12106242; GBIF: https://www.gbif.org/species/7637895; WFO: https://list.worldfloraonline.org/wfo-0000517696; POWO: https://powo.science.kew.org/taxon/2619122-4
= *Aconitumnapellus* [unranked] e *babiogorensef.babiogorense* Zapał., Consp. FI. Gal. Crit. 2: 226 (1908); GBIF: https://www.gbif.org/species/8383207; WFO: https://list.worldfloraonline.org/wfo-0000517697
= *Aconitumnapellus* [unranked] e *babiogorensef.subfissum* Zapał., Consp. FI. Gal. Crit. 2: 227 (1908); GBIF: https://www.gbif.org/species/8104841; GBIF: https://www.gbif.org/species/12048859; WFO: https://list.worldfloraonline.org/wfo-0000517734; POWO: https://powo.science.kew.org/taxon/2619197-4
= *Aconitumnapellus* [unranked] d *carpaticumf.carpaticum* Zapał., Consp. FI. Gal. Crit. 2: 226 (1908); GBIF: https://www.gbif.org/species/12091834; GBIF: https://www.gbif.org/species/12091834; WFO: https://list.worldfloraonline.org/wfo-0000517699
= *Aconitumnapellus* [unranked] b *subtatrense* Zapał., Consp. FI. Gal. Crit. 2: 225 (1908); GBIF: https://www.gbif.org/species/8026543; GBIF: https://www.gbif.org/species/12162386; WFO: https://list.worldfloraonline.org/wfo-0000517736; POWO: https://powo.science.kew.org/taxon/2619199-4
= *Aconitumnapellus* [unranked] b *subtatrensef.abnorme* Zapał., Consp. FI. Gal. Crit. 2: 225 (1908); GBIF: https://www.gbif.org/species/7907677; WFO: https://list.worldfloraonline.org/wfo-0000517691; POWO: https://powo.science.kew.org/taxon/2619113-4
= *Aconitumnapellus* [unranked] b *subtatrensef.latisectum* Zapał., Consp. FI. Gal. Crit. 2: 225 (1908); GBIF: https://www.gbif.org/species/7593018; GBIF: https://www.gbif.org/species/12063034; WFO: https://list.worldfloraonline.org/wfo-0000517717; POWO: https://powo.science.kew.org/taxon/2619162-4
= *Aconitumnapellus* [unranked] b *subtatrensef.subtatrense* Zapał., Consp. FI. Gal. Crit. 2: 225 (1908); GBIF: https://www.gbif.org/species/7982741; WFO: https://list.worldfloraonline.org/wfo-0000517735; POWO: https://powo.science.kew.org/taxon/2619198-4
= *Aconitumnapellus* [unranked] g *tatrense* Zapał., Consp. FI. Gal. Crit. 2: 227 (1908); GBIF: https://www.gbif.org/species/7478935; GBIF: https://www.gbif.org/species/11994294; WFO: https://list.worldfloraonline.org/wfo-0000517740; POWO: https://powo.science.kew.org/taxon/2619201-4
– *Aconitumpalmatifidum* Rchb., Uebers. Gat. Aconitum: 48 (1819) [p. p.]; GBIF: https://www.gbif.org/species/3922207; IPNI: https://www.ipni.org/n/707672-1; WFO: http://www.worldfloraonline.org/taxon/wfo-0000517830; POWO: https://powo.science.kew.org/taxon/707672-1
– *Aconitumskerisorae* auct [e.g., Seitz, Soó], non Gáyer *
– *Aconitumtatrae* Borbás in Pallas, Nagy Lexikona 15: 15 (1897) [p. p.]; GBIF: https://www.gbif.org/species/7278294; WFO: https://list.worldfloraonline.org/wfo-0000518167; POWO: https://powo.science.kew.org/taxon/2619606-4
– *Aconitumtauricum* auct. fl. carpat., non Wulfen

##### Conservation status

In Ukraine – NT (Onyshchenko et al. 2022).

##### Distribution

Pancarpathian endemic.

##### Notes

GBIF (https://www.gbif.org/species/7569378, accessed on 06.06.2023) incorrectly provides A.callibotryonsubsp.scarisorense Grinţ. as a synonym for A.firmumsubsp.firmum. At the same time, GBIF correctly indicates that A.napellussubsp.scarisorense (Grinţ.) Jalas (https://www.gbif.org/species/3922734, accessed on 06.06.2023), a homotypic synonym of A.callibotryonsubsp.scarisorense, belongs to A.firmumsubsp.skerisorae (Gáyer) Starm. (https://www.gbif.org/species/10985587, accessed on 06.06.2023).

#### Aconitum firmum fissurae

Nyár., Enum. Pl. Cheia Turzii: 132 (1939)

88BB089A-CCB7-500E-B9A6-30D8D03E9A99

https://www.catalogueoflife.org/data/taxon/8S9QC

https://www.gbif.org/species/7501250

urn:lsid:ipni.org:names:77122905-1

http://www.worldfloraonline.org/taxon/wfo-0000517252

https://powo.science.kew.org/taxon/77122905-1

https://europlusmed.org/cdm_dataportal/taxon/e6d4ad09-6515-4fc2-a11c-22936e7e1aa9

https://www.worldplants.de/world-plants-complete-list/complete-plant-list?name=Aconitum-firmum

≡ Aconitumnapellussubsp.fissurae (Nyár.) W.Seitz, Feddes Repert. 80: 42 (1969); GBIF: https://www.gbif.org/species/7688050; WFO: https://list.worldfloraonline.org/wfo-0000517708; POWO: https://powo.science.kew.org/taxon/2619144-4
= *Aconitumflerovii* Steinb. in Komarov, Fl. USSR 7: 221, 730 (1937); GBIF: https://www.gbif.org/species/3925362; IPNI: https://www.ipni.org/n/707360-1; WFO: http://www.worldfloraonline.org/taxon/wfo-0000517265; POWO: https://powo.science.kew.org/taxon/707360-1
= *Aconitumhunyadense* Degen, Magyar Bot. Lapok 5: 196 (1906); GBIF: https://www.gbif.org/species/3924541; IPNI: https://www.ipni.org/n/707449-1; WFO: http://www.worldfloraonline.org/taxon/wfo-0000517401; POWO: https://powo.science.kew.org/taxon/707449-1; BHL: https://www.biodiversitylibrary.org/item/202161#page/600
= *Aconitumromanicum* Woł., Fl. Polon. Exsicc. no. 905. *; GBIF: https://www.gbif.org/species/8006246; IPNI: https://www.ipni.org/n/707774-1; WFO: http://www.worldfloraonline.org/taxon/wfo-0000517999; POWO: https://powo.science.kew.org/taxon/2619449-4; JACQ: https://www.jacq.org/detail.php?ID=389701; JSTOR Global Plants: https://plants.jstor.org/stable/10.5555/al.ap.specimen.bm000613645; JSTOR Global Plants: https://plants.jstor.org/stable/10.5555/al.ap.specimen.lw00126605
= Aconitumtatraesubsp.hunyadense (Degen) Soó, Feddes Repert. 83: 135 (1972); GBIF: https://www.gbif.org/species/3931258; IPNI: https://www.ipni.org/n/888821-1; WFO: http://www.worldfloraonline.org/taxon/wfo-0000518168; POWO: https://powo.science.kew.org/taxon/888821-1

##### Conservation status

In Ukraine – NT (Onyshchenko et al. 2022).

##### Distribution

Pancarpathian subendemic.

##### Notes

GBIF (https://www.gbif.org/species/10985587, accessed on 06.06.2023) incorrectly provides A.napellussubsp.fissurae amongst synonyms to A.firmumsubsp.skerisorae (Gáyer) Starm. Aconitumfirmumsubsp.skerisorae is an independent subspecies endemic to Transylvania (Starmühller 2000).

#### Aconitum degenii degenii

Gáyer, Magyar Bot. Lapok 5: 123 (1906)

7F790C7B-DBC9-5BA1-AEFF-C2EA53D6AF51

https://www.catalogueoflife.org/data/taxon/5FDH6

https://www.gbif.org/species/7276900

urn:lsid:ipni.org:names:707304-1

http://www.worldfloraonline.org/taxon/wfo-0000517153

https://powo.science.kew.org/taxon/77122903-1

https://species.wikimedia.org/wiki/Aconitum_degenii

https://europlusmed.org/cdm_dataportal/taxon/4bf508f8-0fb6-42b5-8ea2-8cbe48db894a

https://www.worldplants.de/world-plants-complete-list/complete-plant-list?name=Aconitum-degenii

= Aconitumdegeniif.craciunelense Gáyer, Magyar Bot. Lap. 5: 126 (1906); GBIF: https://www.gbif.org/species/12141980; POWO: https://powo.science.kew.org/taxon/3284590-4; BHL: https://www.biodiversitylibrary.org/item/202161#page/528
= *Aconitummolle* Rchb., Uebers. Gat. Aconitum: 47 (1819); GBIF: https://www.gbif.org/species/3926208; IPNI: https://www.ipni.org/n/707593-1; WFO: http://www.worldfloraonline.org/taxon/wfo-0000517656; POWO: https://powo.science.kew.org/taxon/707593-1
= *Aconitumpaniculatum* [unranked] b *czeremossicum* Zapał., Consp. Fl. Gal. Crit. 2: 220 (1908); GBIF: https://www.gbif.org/species/8037520; WFO: https://list.worldfloraonline.org/wfo-0000517836; POWO: https://powo.science.kew.org/taxon/2619293-4
= *Aconitumpaniculatum* [unranked] d *intermedium* Zapał., Consp. Fl. Gal. Crit. 2: 221 (1908); POWO: https://powo.science.kew.org/taxon/2619296-4
= Aconitumpaniculatumf.latiusculum Zapał., Consp. Fl. Gal. Crit. 2: 220 (1908); GBIF: https://www.gbif.org/species/12126565; POWO: https://powo.science.kew.org/taxon/2619299-4
= *Aconitumpaniculatum* [unranked] a percalabense Zapał., Consp. Fl. Gal. Crit. 2: 220 (1908); GBIF: https://www.gbif.org/species/7721719; POWO: https://powo.science.kew.org/taxon/2619303-4
= *Aconitumpaniculatum* [unranked] c *prutense* Zapał., Consp. Fl. Gal. Crit. 2: 221 (1908); GBIF: https://www.gbif.org/species/7793959; POWO: https://powo.science.kew.org/taxon/2619306-4; POWO: https://powo.science.kew.org/taxon/2619307-4
= *Aconitumpaniculatum* [unranked] c *prutensef.lobatum* Zapał., Consp. Fl. Gal. Crit. 2: 221 (1908)
= *Aconitumpaniculatum* [unranked] c *prutensef.subintermedium* Zapał., Consp. Fl. Gal. Crit. 2: 221 (1908)
= Aconitumpaniculatumf.tenuifissum Zapał., Consp. Fl. Gal. Crit. 2: 220 (1908); GBIF: https://www.gbif.org/species/8294225; POWO: https://powo.science.kew.org/taxon/2619301-4
= *Aconitumprutense* (Zapał.) Tzvelev, Bot. Zhurn. (Moscow & Leningrad) 81(12): 115 (1997) *; GBIF: https://www.gbif.org/species/3921802; IPNI: https://www.ipni.org/n/996847-1; WFO: http://www.worldfloraonline.org/taxon/wfo-0000517917; POWO: https://powo.science.kew.org/taxon/996847-1
– *Aconitumhebegynum* auct. fl. carpat., non DC. [p. p.] *
– *Aconitumpaniculatum* Lam., Fl. Fr. 3: 646 (1778) [p. p., nom. inval.] *; GBIF: https://www.gbif.org/species/7276809; IPNI: https://www.ipni.org/n/707675-1; WFO: http://www.worldfloraonline.org/taxon/wfo-0000517834; POWO: https://powo.science.kew.org/taxon/707675-1; BHL: https://www.biodiversitylibrary.org/page/10168296#page/648
– *Cammarumpaniculatum* (Arcang.) Fourr., Ann. Soc. Linn. Lyon sér. 2 16: 327 (1868) [p. p.]; GBIF: https://www.gbif.org/species/5616149; IPNI: https://www.ipni.org/n/709367-1; WFO: http://www.worldfloraonline.org/taxon/wfo-0000582912; POWO: https://powo.science.kew.org/taxon/709367-1; BHL: https://www.biodiversitylibrary.org/item/237401#page/399
– *Delphiniumpaniculatum* (Arcang.) E.H.L.Krause, Deutschl. Fl. (Sturm), ed. 2. 5: 234 (1901) [p. p.], non Host; GBIF: https://www.gbif.org/species/3929050; IPNI: https://www.ipni.org/n/710879-1; WFO: http://www.worldfloraonline.org/taxon/wfo-0000640512; POWO: https://powo.science.kew.org/taxon/710879-1; BHL: https://www.biodiversitylibrary.org/page/55366385#page/236

##### Conservation status

In Ukraine – LC (Onyshchenko et al. 2022). Global – LC (Mitka 2019).

##### Distribution

Pancarpathian endemic.

#### Aconitum lasiocarpum kotulae

(Pawł.) Starm. & Mitka, Acta Soc. Bot. Polon. 69(2): 150 (2000)

EBA09984-85B5-5FFB-8E8F-9803C49176F5

https://www.catalogueoflife.org/data/taxon/7J77P

https://www.gbif.org/species/3923695

urn:lsid:ipni.org:names:1017003-1

http://www.worldfloraonline.org/taxon/wfo-0000517522

https://powo.science.kew.org/taxon/1017003-1

https://europlusmed.org/cdm_dataportal/taxon/4c78b8c9-e199-41f4-8248-3c3be1940167

https://www.worldplants.de/world-plants-complete-list/complete-plant-list?name=Aconitum-lasiocarpum

≡ Aconitumvariegatumsubsp.kotulae Pawł., FI. Tatr 1: 275 (1956); GBIF: https://www.gbif.org/species/12058061; GBIF: https://www.gbif.org/species/7777045; WFO: https://list.worldfloraonline.org/wfo-0000518265; POWO: https://powo.science.kew.org/taxon/2619702-4
≡ Aconitumvariegatumf.kotulae (Pawł.) Skalický, Preslia 54(2): 119 (1982); GBIF: https://www.gbif.org/species/3930764; IPNI: https://www.ipni.org/n/920448-1; WFO: http://www.worldfloraonline.org/taxon/wfo-0000518266; POWO: https://powo.science.kew.org/taxon/920448-1
= *Aconitumbeskidense* (Zapał.) Gáyer, Magyar Bot. Lapok 10: 201 (1911); GBIF: https://www.gbif.org/species/7861353; WFO: https://list.worldfloraonline.org/wfo-0000516976; POWO: https://powo.science.kew.org/taxon/2618437-4; BHL: https://www.biodiversitylibrary.org/item/201840#page/233
= *Aconitumcammarum* [unranked] a beskidense Zapał., Consp. FI. Gal. Crit. 2: 215 (1908); GBIF: https://www.gbif.org/species/12167522; WFO: https://list.worldfloraonline.org/wfo-0000517030; POWO: https://powo.science.kew.org/taxon/2618487-4
= *Aconitumcammarum* [unranked] c *koscieliskanum* Zapał., Consp. Fl. Gallic. Crit., 2: 215 (1908); GBIF: https://www.gbif.org/species/7787241; WFO: https://list.worldfloraonline.org/wfo-0000517035; POWO: https://powo.science.kew.org/taxon/2618491-4
= Aconitumgracilesubsp.grosserratumf.beskidense (Zapał.) Grinț., FI. Rep. Pop. Rom. 2: 485 (1953); GBIF: https://www.gbif.org/species/11985202; GBIF: https://www.gbif.org/species/8257779; WFO: https://list.worldfloraonline.org/wfo-0000517322; POWO: https://powo.science.kew.org/taxon/2618771-4
= *Aconitumpaniculatum* [unanked] e *podolicum* Zapał., Consp. FI. Gal. Crit. 2: 221 (1908); GBIF: https://www.gbif.org/species/8236504; GBIF: https://www.gbif.org/species/8149503; WFO: https://list.worldfloraonline.org/wfo-0000517847; POWO: https://powo.science.kew.org/taxon/2619305-4
= *Aconitumpaniculatum* [unanked] e *podolicumf.latilobum* Zapał., Consp. FI. Gal. Crit. 2: 222 (1908); GBIF: https://www.gbif.org/species/12053893; GBIF: https://www.gbif.org/species/7668283; WFO: https://list.worldfloraonline.org/wfo-0000517840; POWO: https://powo.science.kew.org/taxon/2619298-4
= *Aconitumpodolicum* (Zapał.) Voroshylov, Bjul. Glav. Bot. Sada 158: 39 (1990) *; GBIF: https://www.gbif.org/species/3921913; IPNI: https://www.ipni.org/n/962363-1; WFO: http://www.worldfloraonline.org/taxon/wfo-0000517900; POWO: https://powo.science.kew.org/taxon/962363-1
– *Aconitumlasiocarpum* Rchb., Uebers. Gat. Aconitum.: 55 (1819) [p. p.]; GBIF: https://www.gbif.org/species/7277094; IPNI: https://www.ipni.org/n/707516-1; WFO: http://www.worldfloraonline.org/taxon/wfo-0000517521; POWO: https://powo.science.kew.org/taxon/707516-1

##### Conservation status

In Ukraine – VU (Onyshchenko et al. 2022). Global – NT (Novikov and Mitka 2019).

##### Distribution

Pancarpathian subendemic.

##### Notes

*Aconitumlasiocarpum* (Rchb.) Gáyer is listed in the Red Book of Ukraine as vulnerable species without delimitation of subspecies (Melnyk and Batochenko 2009, MEPNR of Ukraine 2021).

#### Aconitum lasiocarpum lasiocarpum

(Rchb.) Gáyer, Magyar Bot. Lapok 11: 199 (1911)

6A5C7F32-E56F-504E-AB26-2C0B28379164

https://www.catalogueoflife.org/data/taxon/5FDJF

https://www.gbif.org/species/7277095

urn:lsid:ipni.org:names:707516-1

http://www.worldfloraonline.org/taxon/wfo-0000517521

https://powo.science.kew.org/taxon/77227837-1

https://species.wikimedia.org/wiki/Aconitum_lasiocarpum

https://europlusmed.org/cdm_dataportal/taxon/ac18631e-47c1-4016-9e69-aaed03e08cbf

https://www.worldplants.de/world-plants-complete-list/complete-plant-list?name=Aconitum-lasiocarpum

≡ Aconitumnasutumvar.lasiocarpum Rchb., Illustr. Spec. Aconitum: Nr. 47, Tab. 9 (1823–1827); GBIF: https://www.gbif.org/species/7616758; WFO: https://list.worldfloraonline.org/wfo-0000517757; POWO: https://powo.science.kew.org/taxon/2619219-4
≡ Aconitumpaniculatumsubsp.lasiocarpum (Rchb.) Soó, Acta Bot. Hung. 5: 213 (1943); GBIF: https://www.gbif.org/species/8127169; WFO: https://list.worldfloraonline.org/wfo-0000517839; POWO: https://powo.science.kew.org/taxon/2619297-4
≡ Aconitumtoxicumsubsp.lasiocarpum (Rchb.) Grinț., FI. Rep. Pop. Rom. 2: 491 (1953); GBIF: https://www.gbif.org/species/8405975; WFO: https://list.worldfloraonline.org/wfo-0000518204; POWO: https://powo.science.kew.org/taxon/2619645-4
= *Aconitumdasycarpum* (Schur) Schur ex Gáyer, Magyar Bot. Lapok 10: 199 (1911); GBIF: https://www.gbif.org/species/8459344; GBIF: https://www.gbif.org/species/3926069; IPNI: https://www.ipni.org/n/707298-1; WFO: http://www.worldfloraonline.org/taxon/wfo-0000517143; POWO: https://powo.science.kew.org/taxon/707298-1; BHL: https://www.biodiversitylibrary.org/item/201840#page/231
= *Aconitumtoxicum* [unranked] a dasycarpum Schur, Enum. Pl. Transsilv.: 33 (1886); GBIF: https://www.gbif.org/species/7883173; WFO: https://list.worldfloraonline.org/wfo-0000518203; POWO: https://powo.science.kew.org/taxon/2619644-4; BHL: https://www.biodiversitylibrary.org/item/7364#page/53
= *Aconitumvagneri* Kern. ex Gáyer, Magyar Bot. Lapok 10: 199 (1911); GBIF: https://www.gbif.org/species/8665182; GBIF: https://www.gbif.org/species/3930829; IPNI: https://www.ipni.org/n/707928-1; WFO: http://www.worldfloraonline.org/taxon/wfo-0000518249; POWO: https://powo.science.kew.org/taxon/707928-1; BHL: https://www.biodiversitylibrary.org/item/201840#page/231
– *Aconitumlasiocarpum* Rchb., Uebers. Gat. Aconitum.: 55 (1819) [p. p.]; GBIF: https://www.gbif.org/species/7277094; IPNI: https://www.ipni.org/n/707516-1; WFO: http://www.worldfloraonline.org/taxon/wfo-0000517521; POWO: https://powo.science.kew.org/taxon/707516-1

##### Conservation status

In Ukraine – VU (Onyshchenko et al. 2022). Global – NT (Novikov and Mitka 2019).

##### Distribution

SE Carpathian endemic.

##### Notes

This vulnerable species is listed in the Red Book of Ukraine without clarification of the subspecies (Melnyk and Batochenko 2009, MEPNR of Ukraine 2021).

#### Aconitum moldavicum hosteanum

(Schur) Graebn. et P.Graebn., Syn. Mitteleur. Fl. 5(2): 725 (1929)

2BA646EC-F6E4-5A0C-A409-D044C989F0CD

https://www.gbif.org/species/8062401

urn:lsid:ipni.org:names:77249320-1

http://www.worldfloraonline.org/taxon/wfo-0000517652

https://powo.science.kew.org/taxon/77249320-1

https://europlusmed.org/cdm_dataportal/taxon/a8dc028d-0922-4f47-ba8f-1894176c0424

https://www.worldplants.de/world-plants-complete-list/complete-plant-list?name=Aconitum-lycoctonum

https://www.biodiversitylibrary.org/page/25296776#page/735

≡ *Aconitumhosteanum* Schur, Verh. Mitth. Siebenbürg. Vereins Naturwiss. Hermannstadt 2: 177 (1851) [nom. nudum] et Verh. Mitth. Siebenbürg. Vereins Naturwiss. Hermannstadt 3: 84 (1852) [nom. nudum] et Verh. Mitth. Siebenbürg. Vereins Naturwiss. Hermannstadt 4: 49 (1853) *; GBIF: https://www.gbif.org/species/3924597; IPNI: https://www.ipni.org/n/707441-1; IPNI: https://www.ipni.org/n/707442-1; WFO: https://list.worldfloraonline.org/wfo-0000517392; POWO: https://powo.science.kew.org/taxon/707442-1; BHL: https://www.biodiversitylibrary.org/item/42660#page/87; BHL: https://www.biodiversitylibrary.org/item/42660#page/534; BHL: https://www.biodiversitylibrary.org/item/42660#page/701; JACQ: https://www.jacq.org/detail.php?ID=351628; JACQ: https://www.jacq.org/detail.php?ID=351319; JSTOR Global Plants: https://plants.jstor.org/stable/10.5555/al.ap.specimen.lw00201808; JSTOR Global Plants: https://plants.jstor.org/stable/10.5555/al.ap.specimen.lw00150056
≡ *Aconitummoldavicum* [unranked] e *hosteanum* (Schur) Zapał., Consp. Fl. Gal. Crit. 2: 213 (1908)
= Aconitumhosteanumf.borbasii Gáyer, Magyar Bot. Lapok 8: 316 (1909); BHL: https://www.biodiversitylibrary.org/item/201903#page/342
= Aconitumhosteanumvar.geraniifolium Grinț. in Săvul., Fl. Rep. Pop. Rom. 2: 499, 678 (1953)
= Aconitummoldavicumvar.australef.dissectifolium (Zapał.) Grinț. in Săvul., Fl. Rep. Pop. Rom. 2: 497 (1953)
= Aconitummoldavicumvar.australef.fragile Grinț. in Săvul., Fl. Rep. Pop. Rom. 2: 496, 676 (1953)
= Aconitummoldavicumvar.australef.grandiflorum (Schur) Grinț. in Săvul., Fl. Rep. Pop. Rom. 2: 497 (1953)
= Aconitummoldavicumvar.australef.leopoliensis (Zapał.) Grinț. in Săvul., Fl. Rep. Pop. Rom. 2: 497 (1953)
= Aconitummoldavicumvar.australef.obtusidentatum Simonk. ex Gáyer, Magyar Bot. Lap. 8: 315 (1909); BHL: https://www.biodiversitylibrary.org/item/201903#page/341; JSTOR Global Plants: https://plants.jstor.org/stable/10.5555/al.ap.specimen.je00017686; JSTOR Global Plants: https://plants.jstor.org/stable/10.5555/al.ap.specimen.je00017691; JSTOR Global Plants: https://plants.jstor.org/stable/10.5555/al.ap.specimen.je00017689; JSTOR Global Plants: https://plants.jstor.org/stable/10.5555/al.ap.specimen.je00017685; JSTOR Global Plants: https://plants.jstor.org/stable/10.5555/al.ap.specimen.je00017687; JSTOR Global Plants: https://plants.jstor.org/stable/10.5555/al.ap.specimen.je00017688; JSTOR Global Plants: https://plants.jstor.org/stable/10.5555/al.ap.specimen.je00017690
= Aconitummoldavicumvar.australef.thyraicum (Błocki) Grinț. in Săvul., Fl. Rep. Pop. Rom. 2: 497 (1953)
= *Aconitummoldavicum* [unranked] a dissectifolium Zapał., Consp. Fl. Gal. Crit. 2: 212 (1908)
= *Aconitummoldavicum* [unranked] b *grandicassum* Zapał., Consp. Fl. Gal. Crit. 2: 212 (1908)
= *Aconitummoldavicum* [unranked] c *grandiflorum* Schur, Enum. Pl. Transsilv.: 32 (1866)
= *Aconitummoldavicum* [unranked] d *leopoliense* Zapał., Consp. Fl. Gal. Crit. 2: 213 (1908)
= *Aconitumthyraicum* Błocki, Allg. Bot. Z. Syst. 1: 59 (1895) *; GBIF: https://www.gbif.org/species/7986865; IPNI: https://www.ipni.org/n/707888-1; WFO: http://www.worldfloraonline.org/taxon/wfo-0000518189; POWO: https://powo.science.kew.org/taxon/2619630-4; BHL: https://www.biodiversitylibrary.org/item/38475#page/71; JSTOR Global Plants: https://plants.jstor.org/stable/10.5555/al.ap.specimen.l0821162; JSTOR Global Plants: https://plants.jstor.org/stable/10.5555/al.ap.specimen.bm000613637; JSTOR Global Plants: https://plants.jstor.org/stable/10.5555/al.ap.specimen.je00018562; JSTOR Global Plants: https://plants.jstor.org/stable/10.5555/al.ap.specimen.hbg508762; JSTOR Global Plants: https://plants.jstor.org/stable/10.5555/al.ap.specimen.je00018563
= *Aconitummoldavicum* [unranked] e *hosteanumf.czywczynense* Zapał., Consp. Fl. Gal. Crit. 2: 213 (1908)
= *Aconitummoldavicum* [unranked] e *hosteanumf.rodnense* Zapał., Consp. Fl. Gal. Crit. 2: 213 (1908)
– *Aconitummoldavicum* Hacq., Reis. Dac. Sarm. Karpathen 1: 169 (1790) et Hacq. ex Rchb., In: Übers. Gen. Acon.: 67 (1819) [p. p.]; GBIF: https://www.gbif.org/species/8058146; GBIF: https://www.gbif.org/species/7276954; IPNI: https://www.ipni.org/n/77319987-1; WFO: https://list.worldfloraonline.org/wfo-0000517651; POWO: https://powo.science.kew.org/taxon/77319987-1
– Aconitummoldavicumvar.australe (Rchb.) Grinț. in Săvul., Fl. Rep. Pop. Rom. 2: 496 (1953) [p. p.]
– *Delphiniummoldavicum* (Hacq.) Bránadza, Prodr. Fl. Rom.: 11 (1879) [p. p., nom. inval.]

##### Conservation status

In Ukraine – LC (Onyshchenko et al. 2022).

##### Distribution

Pancarpathian subendemic.

##### Notes

Following the Worldplants (https://www.worldplants.de/world-plants-complete-list/complete-plant-list?name=Aconitum-lycoctonum, accessed on 06.06.2023), CoL (https://www.catalogueoflife.org/data/taxon/5FDJJ, accessed on 06.06.2023) and GBIF (https://www.gbif.org/species/3923267, accessed on 06.06.2023), they provide outdated taxonomy for *A.moldavicum* Hacq. and consider it belonging to A.lycoctonumsubsp.moldavicum (Hacq.) Jalas. Such consideration is based, perhaps, on the research of Utelli et al. (2000), who showed the phylogenetic affinity of *A.moldavicum* and *A.lycoctonum* L. in Europe and proposed to delimit its morphs as subspecies. Mitka et al. (2013), Mitka et al. (2016) have further discussed and stressed this question in the context of the biogeography of the genus *Aconitum* L. in the Carpathians. Anatomical studies (Novikoff 2010) also showed that, besides the common features (well-developed differentiated two-layered lignified parenchymal ring and occurrence of peripheral vascular bundles in the stem), *A.lycoctonum* and *A.moldavicum* differ by the position of sclerenchymatous strands supporting the vascular bundles in their stems. In *A.moldavicum*, the parenchyma layer is present between the vascular bundles and sclerenchymatous strands, while, in *A.lycoctonum*, it is absent. Morphological variation allowing to delimit subspecies within *A.moldavicum* was not taken into account by Utelli et al. (2000), but was studied in detail by Mitka (2008). Hence, *A.moldavicum* is currently considered an independent species with a developed infraspecific structure (Mitka and Kozioł 2009, Novikov and Mitka 2020 and Novikov and Mitka 2020).

GBIF (https://www.gbif.org/species/11040564, accessed on 06.06.2023) has a technical mistake and provides the name A.moldavicumsubsp.nothoconfusum (Grin.) A.Novikov – it should be *A.moldavicum* nothosubsp. *confusum* (Grinƫ.) A.Novikov.

#### Aconitum moldavicum moldavicum

Hacq. ex Rchb., Uebers. Gat. Aconitum: 67 (1819)

1F9900B3-1F4C-54D5-91CC-7B9A5046D372

https://www.gbif.org/species/8058146

https://www.gbif.org/species/7276954

urn:lsid:ipni.org:names:77319987-1

http://www.worldfloraonline.org/taxon/wfo-0000517651

https://powo.science.kew.org/taxon/77227195-1

https://species.wikimedia.org/wiki/Aconitum_moldavicum

https://europlusmed.org/cdm_dataportal/taxon/ea0a74d3-376c-4931-82c2-63fd1f84fedb

https://www.worldplants.de/world-plants-complete-list/complete-plant-list?name=Aconitum-lycoctonum

≡ Aconitumlycoctonumsubsp.moldavicum (Hacq.) Jalas, Ann. Bot. Fenn. 22(3): 219 (1985); CoL: https://www.catalogueoflife.org/data/taxon/5FDJJ; GBIF: https://www.gbif.org/species/3923267; IPNI: https://www.ipni.org/n/924000-1; WFO: http://www.worldfloraonline.org/taxon/wfo-0000517604; POWO: https://powo.science.kew.org/taxon/924000-1; JACQ: https://je.jacq.org/JE00017685; JACQ: https://je.jacq.org/JE00017686; JACQ: https://je.jacq.org/JE00017687; JACQ: https://je.jacq.org/JE00017688; JACQ: https://je.jacq.org/JE00017689; JACQ: https://je.jacq.org/JE00017690; JACQ: https://je.jacq.org/JE00017691; JACQ: https://je.jacq.org/JE00017823; JACQ: https://je.jacq.org/JE00017824; JACQ: https://je.jacq.org/JE00017825; JACQ: https://je.jacq.org/JE00017826; JACQ: https://je.jacq.org/JE00017827; JACQ: https://je.jacq.org/JE00017828; JACQ: https://je.jacq.org/JE00017829; JACQ: https://je.jacq.org/JE00017830; JACQ: https://je.jacq.org/JE00018561; JACQ: https://je.jacq.org/JE00018562; JACQ: https://je.jacq.org/JE00018563
= *Aconitumlycoctonum* [unranked] β *caeruleum* Wahlenb., FI. Carp. Princip.: 163 (1814); GBIF: https://www.gbif.org/species/8350826; WFO: https://list.worldfloraonline.org/wfo-0000517594; POWO: https://powo.science.kew.org/taxon/2619024-4
= Aconitummoldavicumsubsp.hacquetianum Grinț., Cat. Sem. Grăd. Bot. Bucovin.: 6 (1945) [nom. nudum]
= Aconitummoldavicumvar.hacquetianum Grinț. in Săvul., Fl. Rep. Pop. Rom. 2: 496 (1953); JACQ: https://ere.jacq.org/ERE0005606
= Aconitummoldavicumvar.hacquetianumf.flexuosum Grinț. in Săvul., Fl. Rep. Pop. Rom. 2: 498, 677 (1953)
= Aconitummoldavicumvar.hacquetianumf.macrocassis Grinț. in Săvul., Fl. Rep. Pop. Rom. 2: 498, 677 (1953)
= Aconitummoldavicumvar.hacquetianumf.piliferum Grinț. in Săvul., Fl. Rep. Pop. Rom. 2: 498, 677 (1953); JACQ: https://ere.jacq.org/ERE0005607
= Aconitummoldavicumvar.rubicundum Borbás, Kárp. Egyl. Ėvk. 5: 247 (1886) et Oesterr. Bot. Z. 36: 318 (1886); BHL: https://www.biodiversitylibrary.org/item/91253#page/326
= Aconitummoldavicumf.stenanthum Gáyer, Magyar Bot. Lapok 6: 297 (1907); BHL: https://www.biodiversitylibrary.org/item/201813#page/321
= *Aconitumseptentrionale* Baumg., Enum. Stirp. Transsilv. 2: 98 (1816), non Koelle *
= *Aconitumtransilvanicum* Lerchenf. ex Schur., Verh. Mitth. Siebenbürg. Vereins Naturwiss. Hermannstadt 10: 165 (1859); GBIF: https://www.gbif.org/species/3931066; GBIF: https://www.gbif.org/species/8527174; IPNI: https://www.ipni.org/n/707900-1; WFO: https://list.worldfloraonline.org/wfo-0000518206; POWO: https://powo.science.kew.org/taxon/707900-1; BHL: https://www.biodiversitylibrary.org/item/42663#page/409
– *Aconitumcarpathicum* (DC.) Sagorski & Schneider, Fl. Centralkarpat.: 45 (1891) [p. p.]; GBIF: https://www.gbif.org/species/7741610; POWO: https://powo.science.kew.org/taxon/2618509-4
– *Aconitumfallacinum* Błocki, Allg. Bot. Z. Syst. 1: 117 (1895) [p. p.]; IPNI: https://www.ipni.org/n/707347-1; WFO: http://www.worldfloraonline.org/taxon/wfo-0000517234; POWO: https://powo.science.kew.org/taxon/2618686-4; BHL: https://www.biodiversitylibrary.org/item/38475#page/129; JACQ: https://hal.jacq.org/HAL0117457; JSTOR Global Plants: https://plants.jstor.org/stable/10.5555/al.ap.specimen.je00017829; JSTOR Global Plants: https://plants.jstor.org/stable/10.5555/al.ap.specimen.je00017826; JSTOR Global Plants: https://plants.jstor.org/stable/10.5555/al.ap.specimen.je00017825; JSTOR Global Plants: https://plants.jstor.org/stable/10.5555/al.ap.specimen.je00017823; JSTOR Global Plants: https://plants.jstor.org/stable/10.5555/al.ap.specimen.je00017827; JSTOR Global Plants: https://plants.jstor.org/stable/10.5555/al.ap.specimen.je00018561; JSTOR Global Plants: https://plants.jstor.org/stable/10.5555/al.ap.specimen.hal0117457; JSTOR Global Plants: https://plants.jstor.org/stable/10.5555/al.ap.specimen.bm000613638; JSTOR Global Plants: https://plants.jstor.org/stable/10.5555/al.ap.specimen.je00017830; JSTOR Global Plants: https://plants.jstor.org/stable/10.5555/al.ap.specimen.je00017828; JSTOR Global Plants: https://plants.jstor.org/stable/10.5555/al.ap.specimen.je00017824
– *Aconitumjacquinianum* Host, Fl. Austr. 2: 68 (1831) [quoad pl. carpat.]; GBIF: https://www.gbif.org/species/3924390; IPNI: https://www.ipni.org/n/707468-1; WFO: https://list.worldfloraonline.org/wfo-0000517422; POWO: https://powo.science.kew.org/taxon/707468-1
– Aconitumlycoctonumsubsp.carpaticum (DC.) Dostal, Květ. ČSR 2: 150 (1950) [p. p.]; GBIF: https://www.gbif.org/species/7448841; WFO: https://list.worldfloraonline.org/wfo-0000517596; POWO: https://powo.science.kew.org/taxon/2619026-4
– Aconitumlycoctonumvar.carpaticum (DC.) Ser., Mus. helv. d'hist. nat. 1: 136 (1822) [p. p.]; GBIF: https://www.gbif.org/species/8273319; WFO: https://list.worldfloraonline.org/wfo-0000517595; POWO: https://powo.science.kew.org/taxon/2619025-4
– *Aconitummoldavicum* Hacq., Reis. Dac. Sarm. Karpathen 1: 169 (1790) et Hacq. ex Rchb., Übers. Gen. Acon.: 67 (1819) [p. p. major]; GBIF: https://www.gbif.org/species/8058146; GBIF: https://www.gbif.org/species/7276954; IPNI: https://www.ipni.org/n/77319987-1; WFO: https://list.worldfloraonline.org/wfo-0000517651; POWO: https://powo.science.kew.org/taxon/77319987-1
– *Aconitummoldavicum* [unranked] c *parvicassum* Zapał., Consp. Fl. Gal. Crit. 2: 212 (1908) [p. p.]
– Aconitummoldavicumf.puberulum Zapał., Consp. Fl. Gal. Crit. 2: 212 (1908) [p. p.]
– *Aconitumseptentrionale* [unranked] β *carpaticum* DC., Syst. Nat. 1: 370 (1818) [p. p.]; GBIF: https://www.gbif.org/species/7520649; WFO: https://list.worldfloraonline.org/wfo-0000518062; POWO: https://powo.science.kew.org/taxon/2619506-4; BHL: https://www.biodiversitylibrary.org/item/127665#page/380
– *Delphiniummoldavicum* (Hacq.) Bránadza, Prodr. Fl. Rom.: 11 (1879) [p. p. major, nom. inval.]

##### Conservation status

In Ukraine – LC (Onyshchenko et al. 2022).

##### Distribution

Pancarpathian subendemic.

#### Ranunculus carpaticus

Herbich, Sel. Pl. Rar. Gallic.: 15 (1836), non Wahlenb. ex Nyman

9E63B9E5-DAC4-5A0B-897F-A2B1223EC38C

https://www.catalogueoflife.org/data/taxon/4RFXM

https://www.gbif.org/species/3921904

urn:lsid:ipni.org:names:712424-1

http://www.worldfloraonline.org/taxon/wfo-0000460647

https://powo.science.kew.org/taxon/712424-1

https://species.wikimedia.org/wiki/Ranunculus_carpaticus

https://europlusmed.org/cdm_dataportal/taxon/d8e20ade-0c13-4253-a130-e2f9c2f68b34

https://www.worldplants.de/world-plants-complete-list/complete-plant-list?name=Ranunculus-carpaticus

https://je.jacq.org/JE00021608

https://www.jacq.org/detail.php?ID=442598

https://www.jacq.org/detail.php?ID=442599

https://plants.jstor.org/stable/10.5555/al.ap.specimen.cher0200025

https://plants.jstor.org/stable/10.5555/al.ap.specimen.je00021608

https://plants.jstor.org/stable/10.5555/al.ap.specimen.cher0200024

= *Ranunculusaduncus* Schur, Enum. Pl. Transsilv. 16 (1866), non Gren. & Godr.; BHL: https://www.biodiversitylibrary.org/item/7364#page/36
= Ranunculuscarpaticusf.anomalus A.Nyár., Fl. Rep. Pop. Rom. 2: 620, 687 (1953)
= Ranunculuscarpaticusf.flabellatus A.Nyár., Fl. Rep. Pop. Rom. 2: 620, 687 (1953)
= Ranunculuscarpaticusf.plenus Zapał., Consp. Fl. Galic. Crit. 2: 274 (1908)
= Ranunculuscarpaticusf.pygmaeus Porcius, Phaner. Năsăud: 152 (1881)
= Ranunculuscarpaticusvar.rupicolus Zapał., Consp. Fl. Galic. Crit. 2: 274 (1908)
= *Ranunculusdentatus* (Baumg.) Freyn in A.Kern., Sched. Fl. Austro-Hung. 5: 47 (1888) *; GBIF: https://www.gbif.org/species/3930794; IPNI: https://www.ipni.org/n/712586-1; WFO: http://www.worldfloraonline.org/taxon/wfo-0000460732; POWO: https://powo.science.kew.org/taxon/712586-1
= *Ranunculusgouani* Baumg., Enum. Stirp. Transsilv. 2: 125 (1816), non alior
= *Ranunculuslerchenfeldianus* Schur, Verh. Mitth. Siebenbürg. Vereins Naturwiss. Hermannstadt 3: 84 (1852) [nom. nudum] et Verh. Mitth. Siebenbürg. Vereins Naturwiss. Hermannstadt 4: 14 (1853); GBIF: https://www.gbif.org/species/3924099; IPNI: https://www.ipni.org/n/713087-1; WFO: http://www.worldfloraonline.org/taxon/wfo-0000460869; POWO: https://powo.science.kew.org/taxon/713087-1; BHL: https://www.biodiversitylibrary.org/page/11525300#page/534; BHL: https://www.biodiversitylibrary.org/page/11525300#page/666; JACQ: https://www.jacq.org/detail.php?ID=362042; JACQ: https://www.jacq.org/detail.php?ID=362813; JACQ: https://www.jacq.org/detail.php?ID=362826; JACQ: https://www.jacq.org/detail.php?ID=362839; JACQ: https://www.jacq.org/detail.php?ID=362842; JSTOR Global Plants: https://plants.jstor.org/stable/10.5555/al.ap.specimen.mel2427577; JSTOR Global Plants: https://plants.jstor.org/stable/10.5555/al.ap.specimen.lw00203232a; JSTOR Global Plants: https://plants.jstor.org/stable/10.5555/al.ap.specimen.lw00203232b; JSTOR Global Plants: https://plants.jstor.org/stable/10.5555/al.ap.specimen.lw00203233; JSTOR Global Plants: https://plants.jstor.org/stable/10.5555/al.ap.specimen.lw00203234; JSTOR Global Plants: https://plants.jstor.org/stable/10.5555/al.ap.specimen.lw00203184
= *Ranunculusmontanus* Willd. [unranked] α *dentatus* Baumg., Enum. Stirp. Transsilv. 2: 124 (1816); GBIF: https://www.gbif.org/species/6710384; POWO: https://powo.science.kew.org/taxon/3298250-4
= *Ranunculuspormbachiensis* Lerchenf. ex Schur, Enum. Pl. Transsilv.: 16 (1866); BHL: https://www.biodiversitylibrary.org/item/7364#page/36
= *Ranunculusschurii* Fuss ex Schur, Enum. Pl. Transsilv.: 16 (1866); GBIF: https://www.gbif.org/species/8666188; GBIF: https://www.gbif.org/species/3925490; IPNI: https://www.ipni.org/n/713787-1; WFO: http://www.worldfloraonline.org/taxon/wfo-0000462193; POWO: https://powo.science.kew.org/taxon/713787-1; BHL: https://www.biodiversitylibrary.org/page/10544067#page/38
= *Ranunculustuberosus* Schur, Oesterr. Bot. Z. 11: 82 (1861) et Enum. Pl. Transsilv.: 16 (1866), non alior.; GBIF: https://www.gbif.org/species/7688016; IPNI: https://www.ipni.org/n/714032-1; POWO: https://powo.science.kew.org/taxon/714032-1; BHL: https://www.biodiversitylibrary.org/openurlmultiple.aspx?id=p28748883|p9224548; BHL: https://www.biodiversitylibrary.org/page/10544067#page/38
– *Ranunculusszurulensis* Lerchenf. ex Schur, Verh. Mitth. Siebenbürg. Vereins Naturwiss. Hermannstadt 4: 14 (1853) [p. p.]; GBIF: https://www.gbif.org/species/8534706; GBIF: https://www.gbif.org/species/3923469; IPNI: https://www.ipni.org/n/713932-1; WFO: http://www.worldfloraonline.org/taxon/wfo-0000462274; POWO: https://powo.science.kew.org/taxon/713932-1; BHL: https://www.biodiversitylibrary.org/page/11525300#page/666

##### Conservation status

In Ukraine – LC (Onyshchenko et al. 2022).

##### Distribution

SE Carpathian endemic.

##### Notes

Almost all databases accessed on 06.06.2023, including CoL (https://www.catalogueoflife.org/data/taxon/4RH2B), GBIF (https://www.gbif.org/species/7276759), POWO (https://powo.science.kew.org/taxon/713262-1), WFO (https://list.worldfloraonline.org/wfo-0000462989) and Worldplants (https://www.worldplants.de/world-plants-complete-list/complete-plant-list?name=Ranunculus-montanus) indicate *R.szurulensis* Lerchenf. ex Schur as a synonym for *R.montanus* Willd. However, Domin and Krajina (on some herbarium labels) indicated that *R.szurulensis* is a synonym for *R.carpaticus*. This requires further exploration, but at least in the sense of Domin and Krajina, *R.szurulensis* should be considered a partial synonym of *R.carpaticus*.

#### Ranunculus malinovskii

Elenevsky et Derv.-Sok., Novosti Sist. Vyssh. Rast. 23: 59 (1986)

94E52158-73E9-54E2-B43C-FBD9F829D7E3

https://www.catalogueoflife.org/data/taxon/4RGWJ

https://www.gbif.org/species/3922948

urn:lsid:ipni.org:names:931315-1

http://www.worldfloraonline.org/taxon/wfo-0000462988

https://powo.science.kew.org/taxon/931315-1

https://europlusmed.org/cdm_dataportal/taxon/c8c99844-6aae-47fe-9652-31b79a864157

https://www.worldplants.de/world-plants-complete-list/complete-plant-list?name=Ranunculus-malinovskii

https://www.jacq.org/detail.php?ID=388543

https://www.jacq.org/detail.php?ID=422905

https://plants.jstor.org/stable/10.5555/al.ap.specimen.lw00122218

https://plants.jstor.org/stable/10.5555/al.ap.specimen.lw00122216

= *Ranunculuskladnii* auct. fl. ucrain. carpat., non Schur *

##### Conservation status

In Ukraine – LC (Onyshchenko et al. 2022).

##### Distribution

SE Carpathian endemic.

##### Notes

Euro+Med (https://europlusmed.org/cdm_dataportal/taxon/c8c99844-6aae-47fe-9652-31b79a864157, accessed on 06.06.2023) considers *R.malinovskii* as a synonym for *R.acris* L. Indeed, *R.malinovskii* and *R.acris* are morphologically similar, but *R.malinovskii* differs by smaller habitus, developed rhizome, weak pubescence of the leaves and stem and longer beak of the fruits (Visjulina 1953, Jelenevsky and Derviz-Sokolova 1986, Chopyk and Fedoronchuk 2015).

Some plants from the higher altitudes in the Ukrainian Carpathians were identified as *R.kladnii* Shur. CoL (https://www.catalogueoflife.org/data/taxon/4RHQG, accessed on 06.06.2023), POWO (https://powo.science.kew.org/taxon/713807-1), WFO (https://list.worldfloraonline.org/wfo-0000462207, accessed on 06.06.2023), Euro+Med (https://europlusmed.org/cdm_dataportal/taxon/61b4424b-1bc9-4ee8-8cda-c5b9d6f5f7d8, accessed on 06.06.2023) and Worldplants (https://www.worldplants.de/world-plants-complete-list/complete-plant-list?name=Ranunculus-serbicus, accessed on 07.06.2023) synonymise *R.kladnii* with *R.serbicus* Vis. GBIF (https://www.gbif.org/species/7277719, accessed on 06.06.2023), instead, considers *R.kladnii* to be a synonym for Ranunculusacrissubsp.acris. However, Jelenevsky and Derviz-Sokolova (1986) pointed out that the mentioned plants from higher altitudes differ from those described by Schur as *R.kladnii*. Jelenevsky and Derviz-Sokolova (1986) also found these plants to be different from *R.acris* and *R.serbicus* and, as a result, proposed a new name – *R.malinovskii.* Hence, all specimens from the Ukrainian Carpathians, identified as *R.kladnii*, appeared to be *R.malinovskii* (Tzvelev 2001, Chopyk and Fedoronchuk 2015).

#### Saxifragales

202889E4-4671-5C8F-BB93-C326128FF074

#### Crassulaceae

64669CA4-08E2-5A64-A776-4CBEAF69128E

#### Sempervivum carpathicum carpathicum

Wettst. ex Prodan, Fl. Rep. Pop. Rom. 1: 530 (1923)

6B58DE3E-8092-5124-AE20-5884EEE435AD

https://www.gbif.org/species/8594037

https://www.gbif.org/species/7334507

urn:lsid:ipni.org:names:20007689-1

http://www.worldfloraonline.org/taxon/wfo-0000437104

https://powo.science.kew.org/taxon/77225188-1

https://species.wikimedia.org/wiki/Sempervivum_carpathicum_subsp._carpathicum

https://europlusmed.org/cdm_dataportal/taxon/dc0af6e4-2449-4717-a20f-c69eba919385

https://www.worldplants.de/world-plants-complete-list/complete-plant-list?name=Sempervivum-montanum

https://wu.jacq.org/WU0034206

≡ *Sempervivumcarpathicum* Wettst. in A.Kern., Sched. Fl. Exs. Austro-Hung. 10: 25. 1913, [nom. nudum] et Wettst. ex Prodan, Fl. Rom. 1: 530 (1923); GBIF: https://www.gbif.org/species/8594037; GBIF: https://www.gbif.org/species/7334507; IPNI: https://www.ipni.org/n/20007689-1; WFO: http://www.worldfloraonline.org/taxon/wfo-0000437104; POWO: https://powo.science.kew.org/taxon/20007689-1
≡ Sempervivummontanumsubsp.carpathicum (Wettst. ex Prodan) A.Berger in Engler & Prantl, Nat. Pflanzenfam., ed. 2 18a: 422 (1930); GBIF: https://www.gbif.org/species/7940262; IPNI: https://www.ipni.org/n/77143230-1; WFO: http://www.worldfloraonline.org/taxon/wfo-0000735970; POWO: https://powo.science.kew.org/taxon/77143230-1
≡ Sempervivummontanumsubsp.carpaticum Wettst. in Sched., Flora Exs. Austro-Hung. (1913) [nom. nudum]
≡ Sempervivummontanumsubsp.carpaticum Wettst. ex Hayek in Hegi, Ill. Fl. Mitt.-Eur. 4(2): 554 (1923) [nom. nudum]; CoL: https://www.catalogueoflife.org/data/taxon/5L5BR; GBIF: https://www.gbif.org/species/8493204
≡ Sempervivummontanumvar.carpathicum (Wettst. ex Prodan) Praeger, An account of the Sempervivum group: 46 (1932) [comb. inval.]
≡ Sempervivummontanumsubsp.eumontanumvar.carpathicum (Wettst. ex Prodan) Domin, Rozpr. Ceské Akad. Ved, Tr. 2, Vedy Mat. Prír. 42(29): 28 (1933); GBIF: https://www.gbif.org/species/7373462; WFO: https://list.worldfloraonline.org/wfo-0000735971; POWO: https://powo.science.kew.org/taxon/2871574-4
= Sempervivummontanumf.brachypetalum Domin, Rozpr. Ceské Akad. Ved, Tr. 2, Vedy Mat. Prír. 42(29): 28 (1933); GBIF: https://www.gbif.org/species/7561540; WFO: https://list.worldfloraonline.org/wfo-0000735972; POWO: https://powo.science.kew.org/taxon/2871575-4
= Sempervivummontanumf.congestum Domin, Rozpr. Ceské Akad. Ved, Tr. 2, Vedy Mat. Prír. 42(29): 28 (1933); GBIF: https://www.gbif.org/species/7627077; WFO: https://list.worldfloraonline.org/wfo-0000735975; POWO: https://powo.science.kew.org/taxon/2871578-4
= Sempervivummontanumvar.pallidum Wettst. ex Hayek in Hegi, Ill. Fl. Mitt.-Eur. 4(2): 554 (1923) [nom. inval.]
= Sempervivummontanumf.pallidum (Wettst. ex Hayek) Fiori, Nuov. Fl. Italia 1: 716 (1923)
= Sempervivummontanumf.pallidum (Wettst. ex Hayek) Domin, Rozpr. Ceské Akad. Ved, Tr. 2, Vedy Mat. Prír. 42(29): 28 (1933) [comb. illeg.]; GBIF: https://www.gbif.org/species/7872574; IPNI: https://www.ipni.org/n/77087400-1; WFO: http://www.worldfloraonline.org/taxon/wfo-0000735974; POWO: https://powo.science.kew.org/taxon/77087400-1
= Sempervivummontanumf.pallidum (Wettst. ex Hayek) Hadrava & Miklánek, Kaktusy (Brno) 43 (Special 1): 11 (2007) [nom. illeg.]; IPNI: https://www.ipni.org/n/77087401-1; POWO: https://powo.science.kew.org/taxon/77087401-1
= Sempervivummontanumf.neopallidum Hadrava & Miklánek, Kaktusy (Brno) 43 (Special 1): 11 (2007) [nom. illeg.]; GBIF: https://www.gbif.org/species/4199474; IPNI: https://www.ipni.org/n/77087402-1; WFO: https://list.worldfloraonline.org/wfo-0001325704; POWO: https://powo.science.kew.org/taxon/77087402-1
= Sempervivummontanumf.speciosum Domin, Rozpr. Ceské Akad. Ved, Tr. 2, Vedy Mat. Prír. 42(29): 28 (1933); GBIF: https://www.gbif.org/species/8138052; WFO: https://list.worldfloraonline.org/wfo-0000735973; POWO: https://powo.science.kew.org/taxon/2871576-4
= Sempervivummontanumf.stenophyllum Domin, Rozpr. Ceské Akad. Ved, Tr. 2, Vedy Mat. Prír. 42(29): 28 (1933); GBIF: https://www.gbif.org/species/8394796; WFO: https://list.worldfloraonline.org/wfo-0000735976; POWO: https://powo.science.kew.org/taxon/2871579-4
= Sempervivummontanumvar.pallidum Wettst. ex Schinz & R. Keller, Fl. Schweiz (Schinz), ed. 2. 2: 96 (1905); IPNI: https://www.ipni.org/n/77087399-1; WFO: http://www.worldfloraonline.org/taxon/wfo-0001400047; POWO: https://powo.science.kew.org/taxon/77087399-1
= Sempervivumwettsteiniisubsp.wettsteinii Letz, Vybrané Problémy Taxonomickej Diferenciácie rodov Sempervivum a Jovibarba v Európe, Thèse Bratislava: 184 (1998) [nom. invalid.] *
– *Sempervivumarachnoideum* auct. [e.g., G.Reuss], non L.
– *Sempervivumheterophyllum* Jáv., Magyar Fl.: 456 (1925), non Haszl.
– *Sempervivummontanum* L., Sp. Pl. 1: 465 (1753) [p. p., tantum quod plantas ucrain. carpat.], non alior *; GBIF: https://www.gbif.org/species/8164781; IPNI: https://www.ipni.org/n/276551-1; WFO: http://www.worldfloraonline.org/taxon/wfo-0000441784; POWO: https://powo.science.kew.org/taxon/276551-1; BHL: https://www.biodiversitylibrary.org/page/358484#page/477
– Sempervivummontanumsubsp.debile auct., non (Schott.) Dostál
– Sempervivummontanumsubsp.heterophyllum auct., non (Haszl.) Jáv. ex Soó
– Sempervivummontanumsubsp.montanum auct. [e.g., Pawłowski, Dostál, Lippert], non L.

##### Conservation status

In Ukraine – NT (Onyshchenko et al. 2022).

##### Distribution

Pancarpathian endemic.

##### Notes

The Red Book of Ukraine lists this species as *S.montanum* s.l. (Kobiv 2009, MEPNR of Ukraine 2021).

There are two subspecies within S.carpathicumWettst. ex Prodan –subsp.carpathicumandsubsp.heterophyllum (Hazsl.) Letz (occurs in Slovakia – Letz 2002). However, *S.carpathicum* is sometimes (e.g. in CoL – https://www.catalogueoflife.org/data/taxon/5L5BR, accessed on 06.06.2023) considered as a subspecies of *S.montanum* L. *Sempervivummontanum*, in general, has a wider distribution range and three (https://powo.science.kew.org/taxon/276551-1, accessed on 06.06.2023) to five (https://www.worldplants.de/world-plants-complete-list/complete-plant-list?name=Sempervivum-montanum, accessed on 07.06.2023) delimited subspecies (i.e. subsp. *montanum, subsp. burnatii* Wettst. ex Hayek, subsp. subsp. carpaticum Wettst. ex Hayek, subsp. rex Niederle and subsp. stiriacum (Wettst. ex Hayek) Hayek). Nevertheless, even in such a case, only S.montanumsubsp.carpaticum occurs in the Ukrainian Carpathians (Chopyk and Fedoronchuk 2015). Worldplants also mentions the presence of S.montanumsubsp.montanum for Ukraine, but no recent reports confirm this. Previous reports of S.montanumsubsp.montanum from Ukraine probably result from some mistaken taxonomic interpretation (Letz and Marhold 1998) of lowland plants of *S.monatnum* that also occur in the flora of Poland and Slovakia (Pawłowski 1956, Zahradníková 1985, Dostál 1989, Jalas 1999). Moreover, GBIF (ttps://www.gbif.org/species/7771274, https://www.gbif.org/species/8674343, accessed on 06.06.2023) synonymises *S.heterophyllum* Haszl. (≡ S.carpathicumsubsp.heterophyllum (Hazsl.) Letz) with S.carpathicumsubsp.carpathicum, which is not entirely correct. Only a part of S.carpathicumsubsp.heterophyllum (i.e. in the sense of Jávorka) can be treated as a synonym for S.carpathicumsubsp.carpathicum (Letz 2002).

#### Sempervivum globiferum preissianum

(Domin) M.Werner, Avonia 28(4): 191 (2011)

61E9B099-B715-5FC7-94F2-5DC810976D47

https://www.catalogueoflife.org/data/taxon/5L5BC

https://www.gbif.org/species/7943221

urn:lsid:ipni.org:names:77110762-1

http://www.worldfloraonline.org/taxon/wfo-0001361905

https://powo.science.kew.org/taxon/77110762-1

https://europlusmed.org/cdm_dataportal/taxon/13361222-617f-42b0-b280-edd556d997eb

https://www.worldplants.de/world-plants-complete-list/complete-plant-list?name=Sempervivum-globiferum

≡ *Jovibarbapreissiana* (Domin) Omelczuk & Chopik, Bot. Zhurn. 60(8): 1184 (1975) *; GBIF: https://www.gbif.org/species/9627483; IPNI: https://www.ipni.org/n/274240-1; WFO: http://www.worldfloraonline.org/taxon/wfo-0000355803; POWO: https://powo.science.kew.org/taxon/274240-1
≡ Jovibarbaglobiferasubsp.preissiana (Domin) Holub, Preslia 70(2): 106 (1998); GBIF: https://www.gbif.org/species/6441825; IPNI: https://www.ipni.org/n/1002586-1; WFO: http://www.worldfloraonline.org/taxon/wfo-0000355779; POWO: https://powo.science.kew.org/taxon/1002586-1
≡ Jovibarbaglobiferavar.preissiana (Domin) Hadrava & Miklánek, Kaktusy (Brno) 43 (Special 1): 28 (2007); GBIF: https://www.gbif.org/species/4201475; IPNI: https://www.ipni.org/n/77087405-1; WFO: http://www.worldfloraonline.org/taxon/wfo-0000509637; POWO: https://powo.science.kew.org/taxon/77087405-1
≡ Jovibarbahirtasubsp.preissiana (Domin) Soó, Acta Bot. Hung. 23: 380 (1977); GBIF: https://www.gbif.org/species/8264808
≡ Sempervivumhirtumsubsp.preissianum (Domin) Dostál, Květena ČSR: 537 (1948); GBIF: https://www.gbif.org/species/7440878
≡ *Sempervivumpreissianum* Domin, Bull. Internat. Acad. Sc., Prague, 33: 126 (1932) *; GBIF: https://www.gbif.org/species/4199066; IPNI: https://www.ipni.org/n/276602-1; WFO: http://www.worldfloraonline.org/taxon/wfo-0000441817; POWO: https://powo.science.kew.org/taxon/276602-1; JACQ: https://prc.jacq.org/PRC454424; JSTOR Global Plants: https://plants.jstor.org/stable/10.5555/al.ap.specimen.prc454422; JSTOR Global Plants: https://plants.jstor.org/stable/10.5555/al.ap.specimen.prc454424
≡ Sempervivumsoboliferumsubsp.preissianum (Domin) Pawłowska, Fl. Polska 7: 48, 294 (1955); GBIF: https://www.gbif.org/species/8342394; WFO: https://list.worldfloraonline.org/wfo-0001299919
= Jovibarbaglobiferavar.tatrensis (Domin) Konop & Bendak, Skalnicky, 1981(1): 33 (1981) [nom. illeg.]; GBIF: https://www.gbif.org/species/7776508; JACQ: https://prc.jacq.org/PRC454422
= Jovibarbahirtasubsp.tatrensis (Domin) Á.Löve & D.Löve, Bot. Not. 114: 53 (1961); GBIF: https://www.gbif.org/species/7520442; WFO: https://list.worldfloraonline.org/wfo-0001300750; POWO: https://powo.science.kew.org/taxon/2878711-4
= Jovibarbahirtavar.tatrense (Domin) Soó, Feddes Repert. 83(3): 174 (1972); GBIF: https://www.gbif.org/species/4201267; IPNI: https://www.ipni.org/n/878851-1; WFO: https://list.worldfloraonline.org/wfo-0000355810; POWO: https://powo.science.kew.org/taxon/878851-1
= Jovibarbahirtavar.tatrensis (Dom.) Konop & Bendak, Skalnicky 1981(1): 33 (1981) [nom. illeg.]; GBIF: https://www.gbif.org/species/4201255; IPNI: https://www.ipni.org/n/920004-1
= Sempervivumhirtumf.glabrescens Sabr., Oesterr. Bot. Z. 32: 378 (1882); GBIF: https://www.gbif.org/species/4200162; IPNI: https://www.ipni.org/n/51013624-1; WFO: https://list.worldfloraonline.org/wfo-0001299917; POWO: https://powo.science.kew.org/taxon/3242423-4; BHL: https://www.biodiversitylibrary.org/page/9607775#page/394
= Sempervivumhirtumsubsp.glabrescens (Sabr.) Jáv., Magyar Fl. 2: 458 (1924); GBIF: https://www.gbif.org/species/11028494; GBIF: https://www.gbif.org/species/8816122; WFO: https://list.worldfloraonline.org/wfo-0001299957
= Sempervivumhirtumsubsp.tatrense (Domin) Dostál, Květena ČSR: 537 (1948); GBIF: https://www.gbif.org/species/7844009
= Sempervivumsoboliferumsubsp.preissianumf.minus Domin ex Pawłowska, Fl. Polska 7: 48 (1955)
= Sempervivumsoboliferumsubsp.preissianumvar.tatrense (Domin) Pawłowska, Fl. Polska 7: 48, 294 (1955); GBIF: https://www.gbif.org/species/7850718
= *Sempervivumtatrense* Domin, Rozpr. České Akad. Věd, Tř. 2, Vědy Mat. Přír. 42/29: 20–21 (1933); GBIF: https://www.gbif.org/species/4198415; IPNI: https://www.ipni.org/n/276680-1; WFO: http://www.worldfloraonline.org/taxon/wfo-0000441748; POWO: https://powo.science.kew.org/taxon/276680-1
– Jovibarbaglobiferasubsp.hirta (L.) J. Parn., Bot. J. Lin. Soc. 103(3): 219 (1990) [p. p., tantum quod plantas ucrain. carpat.]; GBIF: https://www.gbif.org/species/4201494; IPNI: https://www.ipni.org/n/956373-1; WFO: http://www.worldfloraonline.org/taxon/wfo-0000355771; POWO: https://powo.science.kew.org/taxon/956373-1
– *Jovibarbasobolifera* (Sims) Opiz, Seznam: 54 (1852) [p. p., tantum quod plantas ucrain. carpat.] *; GBIF: https://www.gbif.org/species/8990928; IPNI: https://www.ipni.org/n/274241-1; WFO: http://www.worldfloraonline.org/taxon/wfo-0000355792; POWO: https://powo.science.kew.org/taxon/274241-1
– *Sempervivumsoboliferum* Sims, Bot. Mag. 35: t. 1457 (1812) [p. p., tantum quod plantas ucrain. carpat.], non Fleisch. & Lindem. *; GBIF: https://www.gbif.org/species/4198581; IPNI: https://www.ipni.org/n/276661-1; WFO: http://www.worldfloraonline.org/taxon/wfo-0000441418; POWO: https://powo.science.kew.org/taxon/276661-1; BHL: https://www.biodiversitylibrary.org/item/14321#page/114

##### Conservation status

In Ukraine – NT (Onyshchenko et al. 2022).

##### Distribution

Pancarpathian subendemic.

##### Notes

*Jovibarba* Opiz. is often synonymised with *Sempervivum* L., but is sometimes considered an independent genus (Chopyk and Fedoronchuk 2015, Kliment et al. 2016, Mirek et al. 2020). In Ukraine, this genus is traditionally recognised as *Jovibarba.* Here, two geographically well-separated *Jovibarba* species occur – lowland *J.sobolifera* Opiz and high-mountainous *J.preissiana* (Domin) Omelczuk et Chopik. Both species are rare and listed in the Red Book of Ukraine (Andriyenko et al. 2009, Chorney 2009b, MEPNR of Ukraine 2021). In addition, *J.heuffelii* (Schott) Á.Löve & D.Löve is sometimes mistakenly mentioned for the Ukrainian Carpathians – this species occurs in Romania, but was never discovered in the Ukrainian Carpathians (Bialt 2001, Chopyk and Fedoronchuk 2015).

CoL (https://www.catalogueoflife.org/data/taxon/5L5BC, accessed on 07.06.2023), GBIF (https://www.gbif.org/species/7943221, accessed on 06.06.2023) and Worldplants (https://www.worldplants.de/world-plants-complete-list/complete-plant-list?name=Sempervivum-globiferum, accessed on 07.06.2023), amongst synonyms of S.globiferumsubsp.preissianum, mentioned J.hirtasubsp.preissiana (Domin) Holub, which is, perhaps, a technical mistake. Holub (1998) did not apply such a combination, but instead used a combination J.globiferasubsp.preissiana (Domin) Holub. It is also interesting that Omelczuk-Mjakushko and Chopik are often mentioned as the authors of *J.preissiana*. This is not a principal mistake, but a result of a complicated publication case. Omelczuk-Mjakushko and Chopik (1975) are, indeed, the authors of the paper where the species is published. However, in the species protologue, near the new name on page 1184, they provided the maiden name of the first author (i.e. Omelczuk). Therefore, the proper authority of this species should be provided as Omelczuk & Chopik.

#### Saxifragaceae

8482FBB5-5D7A-585F-8119-5697806DD2AE

#### Chrysosplenium alpinum

Schur, Verh. Mitth. Siebenbürg. Vereins Naturwiss. Hermannstadt 3(6): 86 (1852) et Verh. Mitth. Siebenbürg. Vereins Naturwiss. Hermannstadt 10: 133 (1859)

C861F363-CB49-5CF2-AFB8-DFCFA4D91B15

https://www.catalogueoflife.org/data/taxon/5YTN5

https://www.gbif.org/species/5567560

urn:lsid:ipni.org:names:790542-1

http://www.worldfloraonline.org/taxon/wfo-0000603880

https://powo.science.kew.org/taxon/790542-1

https://europlusmed.org/cdm_dataportal/taxon/4cb54abb-d957-4541-8621-b7cc55069df5

https://www.worldplants.de/world-plants-complete-list/complete-plant-list?name=Chrysosplenium-alpinum

https://www.biodiversitylibrary.org/item/42660#page/536

https://www.biodiversitylibrary.org/item/42663#page/377

https://w.jacq.org/W0046043

https://w.jacq.org/W0046044

https://w.jacq.org/W0046045

https://w.jacq.org/W0046046

https://w.jacq.org/W18890086003

≡ Chrysospleniumoppositifoliumvar.alpinum Schur, Verh. Mitth. Siebenbürg. Vereins Naturwiss. Hermannstadt, 4(8): 28 (1853) et Enum. Pl. Transsilv.: 241 (1866) *; GBIF: https://www.gbif.org/species/7964184; WFO: https://list.worldfloraonline.org/wfo-0000604048; POWO: https://powo.science.kew.org/taxon/2720224-4; BHL: https://www.biodiversitylibrary.org/item/42660#page/914; BHL: https://www.biodiversitylibrary.org/item/7364#page/261
= *Chrysospleniumglaciale* Fuss, Fl. Transs.: 247 (1866) *; GBIF: https://www.gbif.org/species/5567435; IPNI: https://www.ipni.org/n/790594-1; WFO: http://www.worldfloraonline.org/taxon/wfo-0000603976; POWO: https://powo.science.kew.org/taxon/790594-1
= Chrysospleniumoppositifoliumvar.rosulare (Schot) Schott ex Engl., Nat. Pflanzenfam. ed. 2, 18a: 165 (1930); GBIF: https://www.gbif.org/species/8660071; GBIF: https://www.gbif.org/species/8009567; WFO: http://www.worldfloraonline.org/taxon/wfo-0000604049; POWO: https://powo.science.kew.org/taxon/2720225-4
= *Chrysospleniumrosulare* Schott ex Maxim., Gartenflora 6: 115 (1857) [nom. nudum] et Bull. Acad. Imp. Sci. Saint-Pétersbourg 23: 345 (1877); GBIF: https://www.gbif.org/species/8560914; GBIF: https://www.gbif.org/species/5567837; IPNI: https://www.ipni.org/n/790655-1; WFO: http://www.worldfloraonline.org/taxon/wfo-0000604087; POWO: https://powo.science.kew.org/taxon/790655-1; BHL: https://www.biodiversitylibrary.org/page/40083306#page/153; BHL: https://www.biodiversitylibrary.org/page/5354877#page/189
= *Chrysospleniumtranssilvanicum* Schur, Verh. Mitth. Siebenbürg. Vereins Naturwiss. Hermannstadt 4(8): 28 (1853) et Enum. Pl. Transsilv.: 241 (1866); GBIF: https://www.gbif.org/species/5567792; IPNI: https://www.ipni.org/n/790679-1; WFO: http://www.worldfloraonline.org/taxon/wfo-0000604115; POWO: https://powo.science.kew.org/taxon/790679-1; BHL: https://www.biodiversitylibrary.org/item/42660#page/914; BHL: https://www.biodiversitylibrary.org/item/7364#page/261
– *Chrysospleniumoppositifolium* auct. fl. roman. et ucrain. [e.g., Baumg., Enum. Stirp. Transsilv. 1: 338, Nr. 699 (1816)], non L. *

##### Conservation status

In Ukraine – LC (Onyshchenko et al. 2022).

##### Distribution

SE Capathian endemic.

##### Notes

*Chrysospleniumalpinum* and *C.oppositifolium* L. are two closely-related species that are sometimes synonymised (e.g. Maximowicz (1877), Răvăruţ (1956)).

*Chrysospleniumalpinum* plants are glabrous with entire or almost entire leaves (occur in Romanian and Ukrainian Carpathians), while *C.oppositifolium* plants are pubescent at least in their base and have distinctly dentate leaves (occurring in western and central Europe, but not in Romania or Ukraine – Hrouda and Šourková (1992)). The affinity of these two species resulted in their misinterpretation and inevitable confusion. For example, GBIF provides *C.glaciale* Fuss (https://www.gbif.org/species/5567435, accessed on 06.06.2023) amongst the synonyms of *C.oppositifolium* (https://www.gbif.org/species/7526486, accessed on 06.06.2023). Perhaps, this synonymy resulted from Maximowicz’s (1877) observations. Similarly, GBIF provides *C.rosulare* Schott ex Maxim. (https://www.gbif.org/species/8560914, https://www.gbif.org/species/5567837, accessed on 06.06.2023) amongst synonyms to *C.oppositifolium.* This is because Maximowicz (1877), on page 345, indicated that *C.rosulare, C.alpinum* and *C.glaciale*, are synonyms for *C.oppositifolium.* Maximowicz (1877) noted that Transylvanian plants slightly differ, but concluded that this difference is taxonomically unimportant. In the original protologue of *C.glaciale*, Fuss (1866), on page 247, indeed provided *C.oppositifolium* as a synonym for *C.glaciale*, but he considered *C.oppositifolium* in the sense of Baumgarten, not in the original sense of Linnaeus. In turn, Baumgarten (1816a) on page 338, mentioned glabrous plants with slightly dentate leaves from Romania (i.e. *C.alpinum*, not *C.oppositifolium*). Hence, after analysis of the original protologues, it looks like both species, *C.glaciale* and *C.rosulare*, should be interpreted as synonyms of *C.alpinum*.

## Analysis

### Experience in working with taxonomy-related databases

The work on the current checklist has been a part of the inventory of endemics distributed in the flora of the Ukrainian Carpathians (Novikoff and Hurdu 2015, Novikov and Sup-Novikova 2022a). During the inventory, creating a working list of taxa and their most frequently applied synonyms was necessary because herbarium specimens could be stored under different names. Later, the initial list was updated following the recent taxonomy and extended with other synonyms, including rare ones from old publications. It was found that different taxonomic databases (e.g. Worldplants, Euro+Med and POWO) have different visions of the structure and status of certain taxa, sometimes providing controversial data. Hence, the need to appeal to original protologues and monographic studies arose.

Considering that the data were prepared specially to be deposited in GBIF, the GBIF backbone taxonomy (Grosjean 2019) was the main focus. Moreover, each taxonomic record in GBIF has hyperlinks to principal taxonomic databases, including IPNI, Tropicos, POWO, WFO and CoL. The only exception are the databases Wikispecies and Worldplants – they are not directly crosslinked with GBIF; however, Worldplants is applied as a source of taxonomic data by CoL. Hence, GBIF seems to be the most comprehensive aggregator for gathering all taxonomic information. Moreover, GBIF uses for its backbone taxonomy checklist datasets published directly in GBIF through ChecklistBank. As a result, surprisingly, many rare taxonomic citations were present in GBIF and absent in other specialised taxonomic databases. Therefore, the GBIF backbone taxonomy has been chosen as a starting point for the exploration.

All databases were artificially subdivided on several types during the work regarding their primary focus listed below. It is worth noting that most databases combine the signs of different types.

1) **Nomenclatural** – the databases providing valuable nomenclatural information, including the taxonomic name, author(s) and details about the place of the protologue’s publication without clarification of relationships between listed taxa (i.e. IPNI)

2) **Taxonomic** – the databases providing more or less detailed information about taxa nomenclature, their synonymy and systematics (e.g. CoL, POWO, WFO, Wikispecies, Euro+Med, Worldplants). Such databases often offer extra data on the taxa distribution, type material, treatment etc.

3) **Biogeographic** – the databases primarily provide data on the taxa distribution, but also gather other information, including synonymy and systematics (i.e. GBIF).

4) **Virtual herbaria** – the databases hosting images of the herbarium material, including the type material and related data (e.g. JACQ and JSTOR Global Plants).

5) **Publication repositories** – the databases containing scanned publications with protologues and taxonomic treatments (e.g. Biodiversity Heritage Library (BHL), Biodiversity Literature Repository (BLR), Plazi, GBIF). With virtual herbaria, such databases are beneficial during the nomenclatural work because they provide access to principal taxonomic data.

The taxonomic databases were used to construct an initial working checklist and clarify the systematics of investigated taxa. Later, nomenclatural and biogeographic databases, as well as virtual herbaria and publication repositories, were used to test and develop the constructed checklist.

Despite numerous taxonomic-related databases, none of them is exceptional and exhaustive. Below are provided some pros and cons of the databases that were mostly used during the work on the current checklist.

**1) CoL**

**Pros**: CoL focuses on constructing a standardised and comprehensive checklist of the entire biota and is useful for global exploration. It provides persistent IDs for the valid names, information about authors and publication of the taxa. CoL predominantly uses Worldplants as a source for taxa validation and systematics. There is also minor information on the distribution of the listed taxa and their vernacular names. CoL is curated by a large number of specilaists in the taxonomy of the particular organism group.

**Cons**: CoL does not provide persistent IDs for synonyms and has no separate databases containing details about the authors and standard publications. It provides only simplified synonymy without clarification on whether it is a homotypic or heterotypic synonym. It has no maps visualising the distribution of taxa. It has no direct links to other databases. Additionally, the time lag of introducing new taxa or nomenclatural changes to CoL can be too long, sometimes a year or more.

**2) GBIF**

**Pros**: GBIF primarily aims to gather data on the distribution of living organisms and uses its own taxonomic backbone for systematics purposes. As a result, provided data are mostly related to reported occurrences. It allows us to build precise distribution maps and provides some other metrics facilities. GBIF gathers the data from different sources, which results in the presence of some unique poorly-known synonyms. It also contains other taxonomy-related data, including information about type materials, vernacular names, treatments etc. GBIF provides its own unique IDs for all taxa, including synonyms. Besides this, it provides a comprehensive list of cross-linked IDs applied by other databases.

**Cons**: Due to using different sources to construct the taxonomic backbone, there are some duplicated records (e.g. with some minor orthographic differences). GBIF has no separate databases about authors and publications where taxa were published, except from the taxonomic treatments extracted and provided by Plazi.

**3) IPNI**

**Pros**: IPNI is a fundamental nomenclatural database used as a starting point for validating taxon names, authors’ and publications’ abbreviations and searching for protologues. IPNI provides stable persistent identifiers (LSIDs) for all taxa, publications and authors.

**Cons**: IPNI provides links to POWO, WFO and BHL databases for certain taxa. However, it does not offer any interpretation of relationships between taxa. Therefore, detecting whether a taxon name is valid or a synonym is impossible. It also does not provide details on the principal chorology of taxa, which is sometimes useful for nomenclatural and taxonomic explorations. Besides this, IPNI has some ‘dead’ LSIDs listed in other databases, but suspended for some reason by IPNI. There are some duplicated records.

**4) WFO**

**Pros**: WFO is focused on constructing a taxonomic checklist and classification of the world flora, largely based on POWO. It provides its own unique IDs for all listed taxa, including synonyms. It is interlinked with IPNI and some other databases, including BOLD and GBIF.

**Cons**: It has a visually unfriendly interface with an overuse of bold fonts. Some IDs are integrated into plain text, making them tricky to find. Each taxon has two different pages (within the list and stand-alone taxonomic page – for example, https://wfoplantlist.org/plant-list/taxon/wfo-0000788843-2023-06 and http://www.worldfloraonline.org/taxon/wfo-0000788843), which is confusing. There is not even basic information about the distribution of the listed taxa. There are some duplicated records.

**5) POWO**

**Pros**: POWO is one of the most used databases during the construction of the current checklist. It has a user-friendly interface and provides the most important information about listed taxa, including extended synonymy with the indication of homotypic and heterotypic synonyms, distribution details on the level of countries with the indication of native and introduced ranges, plant photos, links to digitised herbarium materials, list of related publications and other sources and links to some other databases (e.g. IPNI). POWO also applies LSIDs like IPNI.

**Cons**: The only disadvantage of POWO is the absence of independent databases dealing with authors and publications, which IPNI substitutes. There are some duplicated records.

**6) Wikispecies**

**Pros**: Some rare and unique names are represented that are not listed in other databases, which makes Wikispecies necessary to check. Classification of certain groups is often provided by narrow specialists. There are usually photos of listed plants. There are often provided vernacular names.

**Cons**: Wikispecies has numerous limitations due to its construction. In particular, the absence of persistent IDs, providing data as plain text, application of unusual abbreviations etc. Wikispecies is constructed mostly by enthusiasts, so many taxa are absent in this database.

**7) Euro+Med**

**Pros**: Some exclusive synonymy and systematics resulted from elaborating certain taxa by narrow specialists. Good maps with detailed information about data sources. Clustered synonymy with differentiation of homotypic and heterotypic synonyms. For some taxa, Euro+Med provides vernacular names in languages from the distribution range.

**Cons**: Some data, especially regarding the systematics of problematic taxa, are outdated. The database covers a limited geographical range, while some represented taxa have distribution extended out of this range. Unfriendly pop-up taxa list. Application of long URNs instead of short unique persistent IDs and absence of such IDs for synonyms.

**8) Worldplants**

**Pros**: Stand-alone database providing an alternative vision on plant systematics, which often differs from those proposed by POWO, Euro+Med and GBIF. This database offers detailed and precise nomenclature and systematics operatively updated following the newly-published data. It contains numerous rare synonyms from regional floras that are mostly absent in other databases. It also provides quite detailed information about taxa distribution in different countries.

**Cons**: The absence of persistent IDs and stable links makes it hard to cite certain taxon. The synonyms are listed as plain text without clarification of their type. Applied abbreviations for the authors and publications often differ from those used by IPNI and other databases. The old-fashion interface does not attract.

**9) BHL**

**Pros**: The principal repository of old printed materials related to biodiversity, providing a unique opportunity to work directly with protologues. BHL provides DOIs for some of the publications. It also offers persistent IDs for all content, allowing links to a certain page from the publication. It automatically searches and displays the taxa mentioned on the selected page. Allows us to download the content freely.

**Cons**: Slightly complicated interface with a somewhat unfriendly searching procedure.

**10) JACQ**

**Pros**: One of the virtual herbaria; extremely useful for studies related to the Carpathian Region. Provides free access to scanned herbarium vouchers (including the type material) with high resolution and to the data parsed from the herbarium labels. For some entries, JACQ provides persistent IDs.

**Cons**: Not all entries are supported by scans.

**11) JSTOR Global Plants**

**Pros**: Another virtual herbarium, which focuses mostly on gathering the images of type material and providing both scans and data parsed from the labels. It offers stable links similar to DOIs.

**Cons**: Paid access.

**12) PLAZI TreatmentBank**

**Pros**: Provides direct access to parsed taxonomic treatments and mentions of taxa in the publications and grants UUIDs for these records.

**Cons**: Undeveloped interface. Complicated search procedure. Absence of the treatments for most of the analysed taxa.

### Issues revealed during the work with databases

Besides the inconsistency in nomenclatural and taxonomic visions, when different taxa are considered to be independent or merged as synonyms (e.g. *Aconitummoldavicum* Hacq. and *A.lycoctonum* L.) or considered at different taxonomic ranks (e.g. *Koeleriatranssilvanica* Schur versus Koeleriamacranthasubsp.transsilvanica (Schur) A.Nyár.) by different data providers, several other issues were detected and resolved viz.:

1) Some taxa have two or more **duplicated checklist records**. For example, in GBIF, there are duplicated checklist records for *Campanulamicrophylla* Kit. ex Schult. (https://www.gbif.org/species/5411213 and https://www.gbif.org/species/7654241, accessed on 06.06.2023), *Thesiumserratum* Kit. ex Schult. (https://www.gbif.org/species/7614045 and https://www.gbif.org/species/7390879, accessed on 06.06.2023), *Alsinepauciflora* Kit. ex Nyman (https://www.gbif.org/species/8455786 and https://www.gbif.org/species/3807842, accessed on 06.06.2023) and many other species. Such issues, in most cases, including other databases, result from data aggregation from different sources that can provide data of different quality. Providing the same data, but containing even minor differences (mistakes or technical errors) or incomplete data can result in their automatic interpretation as independent records. Therefore, it is necessary to revise and catch such duplicated records manually.

2) Some of the records in IPNI (e.g. Centaureamollisf.maramarosiensis Jáv. – https://www.ipni.org/n/50909566-1, accessed on 23.07.2023) are **suppressed for unclear reasons**, while the assigned LSIDs are still in use in other databases (e.g. https://powo.science.kew.org/taxon/50909566-1, accessed on 23.07.2023) and provided nomenclatural information is correct.

3) Some taxonomic records provide **incomplete and/or incorrect authorship** for taxa. For example, all databases provide for *Jovibarbapreissiana* authorship (Domin) Omel'chuk-Myakushko & Chopik (https://www.gbif.org/species/9627483, accessed on 06.06.2023), but it should be (Domin) Omelczuk & Chopik (this is clearly indicated in the original protologue of the species and also recognised in the IPNI authors database (https://www.ipni.org/n/274240-1, accessed on 06.06.2023), which bases its abbreviations on the respective TDWG). For *Koeleriatenuipes* and its homonyms, all databases display Domin as an author of the basionym. However, Ujhelyi (1965) 191) pointed out that it is Schur and provided the correct name – *Koeleriatenuipes* (Schur) Ujhelyi. Another example, GBIF provides the record for *Lathyrustranssilvanicus* Fritsch (https://www.gbif.org/species/8329194, accessed on 06.06.2023), while it should be *Lathyrustranssilvanicus* (Spreng.) Fritsch. Some similar authorship issues were also revealed and fixed while elaborating on the current checklist.

4) Some taxonomic records provide **missing, incomplete and/or incorrect protologue data.** For example, in GBIF, pages are not indicated for protologues of Campanulanapuligeraf.longisepala (Nyár.) Morariu (https://www.gbif.org/species/8397712, accessed on 06.06.2023), Campanularotundifoliavar.grandiflora J.A.Knapp (https://www.gbif.org/species/7764236, accessed on 06.06.2023) and many other taxa. Protologue data are missing for *Minuartiaoxypetala* (Woł.) Kulczyński (https://www.gbif.org/species/7504529, accessed on 06.06.2023), Minuartiavernasubsp.oxypetala (Woł.) G.Halliday (https://www.gbif.org/species/8333640, accessed on 06.06.2023), *Genistaoligosperma* (Andrae) Simonk. (https://www.gbif.org/species/5347633, accessed on 06.06.2023) and many other taxa. For some taxa, GBIF provides empty taxonomic records with missing protologue data, for example, for Campanulanapuligeravar.alpiniformis Nyár. ex Morariu (https://www.gbif.org/species/8554545, accessed on 06.06.2023).

5) Occasionally taxonomic records provide **not the first published protologue**. For example, the name *Dianthusmicrochelus* B.S.Williams (https://www.gbif.org/species/3810687, https://powo.science.kew.org/taxon/153597-1, accessed on 06.06.2023) has been first published in 1890 (not in 1891, as indicated in World Plants, GBIF and POWO). Similarly, the name *Trifoliumsarosiense* Hazsl. (https://www.gbif.org/species/5358815, https://powo.science.kew.org/taxon/523672-1, accessed on 06.06.2023) has been first published in 1864 (not in 1867). Such dating mistakes occur mostly due to the inaccessibility of many old publications, especially periodicals that were published locally. In some cases, such mistakes had resulted from publication tardiness – before many journals published their volumes in the consequent year (e.g. the last volume from 1912 could be published in 1913). Sporadically, such dating mistakes result due to the use of re-prints instead of original publications. Fortunately, currently, BHL and other virtual libraries provide access to more and more rarities allowing the detection of such dating issues and discovering the first publications with the original protologues of many taxa.

6) For many taxa, all elaborated databases provide an **incomplete list of synonyms**. Some taxa, especially those published in old local periodicals and monographs, are missing from the databases. In particular, there are often missing taxa published by Zapałowicz (1906b) in the “Conspectus florae Galiciae criticus” – for example, many infraspecific taxa of *Alsinezarencznyi* Zapał. Additionally, there are some missing taxa published in “Flora Reipublicae Populare România” (Săvulescu 1952) – for example, infraspecific taxa of *Aconitumcallibotryon* Rchb. GBIF has no taxonomic record about the name *Melandriumzawadzkii* (Herbich) A. Braun, which is often applied as an alternative name for *Silenezawadzkii* Herbich (https://www.gbif.org/species/5587094, accessed on 06.06.2023) in the Ukrainian herbaria. World Plants, CoL and GBIF completely miss the data on Scabiosalucidasubsp.barbata Nyár., its homonyms and infraspecific derivates.

7) In some cases, the **taxonomic rank is indicated incorrectly**. For example, GBIF provides a taxonomic record for Campanulapolymorphaf.reflectans Hruby (https://www.gbif.org/species/5410039, accessed on 06.06.2023). However, this taxon has been described as a subform and, hence, the correct citation should be Campanulapolymorphasubf.reflectans Hruby (initially described as Campanulapolymorphavar.typicaf.lepidasubf.reflectans – Hruby (1930)). Similarly, GBIF mistakenly indicates the rank of subspecies for Aconitumkoelleanumvar.firmum (Rchb.) Rchb. (https://www.gbif.org/species/12133197, accessed on 06.06.2023), Aconitumpaniculatumf.latilobum Zapał. (https://www.gbif.org/species/12053893, accessed on 06.06.2023) and many other *Aconitum* L. taxa.

8) The **lack of synonymic interlinkage** for existing taxonomic records has been observed in some cases. For example, in GBIF, there is a taxonomic record for *Campanulastenophylla* (Schur) Witasek (https://www.gbif.org/species/7633288, accessed on 06.06.2023), but it is not linked to the record of the valid taxon C.tatraesubsp.tatrae (https://www.gbif.org/species/7222073, accessed on 06.06.2023). Similarly, the taxonomic record of *Trifoliumsarosiense* Hazsl. ex Neilr. (https://www.gbif.org/species/8013811, accessed on 06.06.2023) is not linked to the parental record of *Trifoliumsarosiense* Hazsl. (https://www.gbif.org/species/5358815, accessed on 06.06.2023).

9) Many databases **omit orthographical variants** that often appear in taxonomy. For example, GBIF provides the only variant *Minuartiazarecznyi* (Zapał.) Klokov (https://www.gbif.org/species/7267413, accessed on 06.06.2023), while it is often written as *Minuartiazarencznii*. In this checklist, such orthographical variants are considered.

10) The **uneven approach in building the nomenclatural backbone**. Amongst all databases, only IPNI has an ultimative structure comprising three main nomenclatural elements (i.e. the name of the taxon, the author(s) of the taxon and the publishing source) as independent datasets and providing unique urns to each of these elements. For example, *Campanulacarpatica* has a LSID names:140068-1 (https://www.ipni.org/urn:lsid:ipni.org:names:140068-1, accessed on 06.06.2023), its author, Nicolaus Jacquin, has its own LSID – authors:12576-1 (https://www.ipni.org/urn:lsid:ipni.org:authors:12576-1, accessed on 06.06.2023) and the publication with the protologue, Hortus Botanicus Vindobonensis, has its own LSID – publications:3465-2 (https://www.ipni.org/urn:lsid:ipni.org:publications:3465-2, accessed on 06.06.2023). This is extremely useful because, sometimes, it is not clear where the taxon was published. By browsing other taxa of the same author, it is possible to locate the protologue. Such an operation is impossible without independent datasets with taxa names, authors and publications. Moreover, besides the urns, IPNI proposes standard abbreviations for the authors and publications, which makes it more accessible to routine work with nomenclatural data. Unfortunately, all other databases provide unique ids (if any) only for the taxa and do not allow navigating amongst the authors and their publications, which limits their application for nomenclatural work.

11) The **lack or excessive length of unique IDs for listed taxa**. Most of the elaborated databases provide unique IDs for the hosted taxa. In particular, POWO applies IPNI's LSIDs (https://powo.science.kew.org/taxon/urn:lsid:ipni.org:names:77221836-1, accessed on 06.06.2023). While CoL, GBIF and WFO have their own short persistent IDs (e.g. https://www.catalogueoflife.org/data/taxon/3KXBD or https://list.worldfloraonline.org/wfo-0000745368, accessed on 06.06.2023), Euro+Med also applies persistent IDs in the form of UUIDs (e.g. https://europlusmed.org/cdm_dataportal/taxon/616ef7e8-6c89-4d43-8f5b-b52b0bb164b0, accessed on 06.06.2023). The Wikispecies simply applies URLs (e.g. https://species.wikimedia.org/wiki/Heracleum_carpaticum, accessed on 06.06.2023). Regardless of the ID type, most databases provide IDs both to valid taxa and their synonyms and build independent pages for them, allowing cross-linkage and precise citation of each of the taxa (valid or synonymic). Unfortunately, Euro+Med, Worldplants and Wikispecies have limitations in operation with synonyms. In Euro+Med, synonyms also have unique ids, but they have no independent pages and, therefore, it is not possible to dirrectly navigate to them – they are only highlighted in the list of synonyms and generated links are extremely long (e.g. https://europlusmed.org/cdm_dataportal/taxon/43877abc-2ef9-4cbf-afaf-d18e92f3cf63/synonymy?highlite=676063a0-c78d-433b-9c76-efdfb15a2d15&acceptedFor=676063a0-c78d-433b-9c76-efdfb15a2d15#676063a0-c78d-433b-9c76-efdfb15a2d15, accessed on 06.06.2023). In Worldplants and Wikispecies, only valid taxa have their own URLs and synonyms are simply listed as a plain text (e.g. https://species.wikimedia.org/wiki/Scilla_kladnii, accessed on 06.06.2023). Moreover, in Worldplants, even valid species are bulked together within the same page of the genus, while the ids are temporarily generated, which makes it impossible to provide a permanent link to certain species.

### General recommendations to taxonomy-related databases

Considering my experience in working with and combining data from different taxonomic-related databases, I can outline a few following recommendations for these databases, which, in general, correspond to FAIR principles (Wilkinson et al. 2016, GO FAIR 2023). These recommendations aim to increase the speed and quality of automatic or semi-automatic data processing and data reusability by individual researchers.

1) Use **persistent and resolvable persistent IDs for all taxon names**, including synonymic ones. Applying unique persistent IDs assures the ability to easily recombine and interlink the names following new taxonomic visions and particular nomenclatural purposes.

2) Assign **unique persistent IDs to all elements of the taxon name**, including name, authority and publication source. This allows us to navigate between different elements of the taxon name quickly, find the original publications and interlink them, providing surplus analysis on the value of certain publication sources.

3) Use of the **standard abbreviations for the taxon authors and at least principal publications sources**. Since the taxon authors and nomenclatural citations are frequently abbreviated in different ways, sometimes it is extremely hard to findi the original publication for further nomenclatural exploration. This is especially evident in work with rare old publications and journal series that have changed their name several times. Together with providing unique ids, the application of standardised abbreviations allows us to correctly navigate amongst the authors and publications.

4) Providing at least **basic data on the distribution** of specified taxon helps a lot in preliminary decisions during the initial explorations. The databases with maps of the distribution or with the list of countries where the taxon occurs are very helpful in building the initial lists for further nomenclatural and taxonomic investigations because they allow us to exclude the taxa that are out of the area of interest and, at the same time, locate some obviously problematic taxa requiring special attention.

5) Providing the **links to the original publications with protologues** is crucial because it speeds the taxonomic revision process and assures correct and unambiguous elaboration of the original material by different investigators. Providing links to other related databases could be also helpful. However, different databases develop with different intensities and support, so extensive cross-linkage can result in the appearance of dead links that should be additionally maintained.

6) Providing the **history or the log of the changes** occurring with taxa names would help to understand which issue was detected and for which reason the name has been suspended or modified. At the moment, such an option is partly realised only in the WFO database.

7) Providing the **bibliographic reference to the works containing taxa descriptions** (with an option to download it in RIS/ BibTex format or at least displayed as plain text following one of the citation styles like APA) would be helpful in two ways – (a) *practical*, since the researchers will not waste their time on searching and formatting proper citations in case of need to provide in-text references with analysis of the original publications; (b) *respective*, since providing the full citations for taxa names (all or at least recently published) will increase the citation rate of nomenclatural and taxonomic works that are generally significantly unacknowledged (Valdecasas et al. 2000, Haszprunar 2011, Pyke 2014). Unfortunately, at the moment such an option is not realised in any of the used databases.

## Supplementary Material

3389BB57-56F7-53EB-BD20-80C29DE6ADB8 10.3897/BDJ.11.e103921.suppl1 Supplementary material 1 An alphabetic index of endemic species and infraspecific taxa of vascular plants distributed in the Ukrainian Carpathians Data type Checklist Brief description This is an alphabetically ordered checklist of endemic vascular plants distributed in the Ukrainian Carpathians. File: oo_866643.docx https://binary.pensoft.net/file/866643 Andriy Novikov

15F10DBE-A266-5496-B31D-90A1AC65FE9D 10.3897/BDJ.11.e103921.suppl2 Supplementary material 2 Clustered synonymic checklist of endemic species and infraspecific of vascular plants taxa distributed in the Ukrainian Carpathians Data type Checklist Brief description This is a hierarchically ordered (with clustered homotypic synonyms) checklist of endemic vascular plants distributed in the Ukrainian Carpathians. File: oo_866642.docx https://binary.pensoft.net/file/866642 Andriy Novikov

938B05A2-2BD4-5B2A-90B4-9FB186459E13 10.3897/BDJ.11.e103921.suppl3 Supplementary material 3 Revealed accessions to molecular data on the investigated species Data type molecular data Brief description This table contains all accessions to molecular data regarding investigated species and revealed using the ENA and BOLD facilities. File: oo_873626.xlsx https://binary.pensoft.net/file/873626 Andriy Novikov
