# Supplementary material for: An annotated nomenclatural checklist of endemic vascular plants distributed in the Ukrainian Carpathians
Source: Biodivers Data J. 2023 Aug 11;11:e103921. doi: 10.3897/BDJ.11.e103921 (PMC10848708; doi:10.3897/BDJ.11.e103921)
Supplement: Supplementary material 1 — An alphabetic index of endemic species and infraspecific taxa of vascular plants distributed in the Ukrainian Carpathians [file bdj-11-e103921-s001.docx]

**Supplement A. An alphabetic index of endemic species and infraspecific taxa of vascular plants distributed in the Ukrainian Carpathians**

*Achillea atrata* Baumg., non L. = ***Achillea oxyloba* subsp. *schurii*** (Sch.Bip.) Heimerl

*Achillea dacica* Simonk. = ***Achillea oxyloba* subsp. *schurii*** (Sch.Bip.) Heimerl

***Achillea oxyloba*** subsp. ***schurii*** (Sch.Bip.) Heimerl

*Achillea schurii* f. *pleiocephala* Bommüller = ***Achillea oxyloba* subsp. *schurii*** (Sch.Bip.) Heimerl

*Achillea schurii* Sch.Bip.* *≡* ***Achillea oxyloba* subsp. *schurii*** (Sch.Bip.) Heimerl

*Achillea schurii* var. *dacica* (Simonk.) Prodan & Nyár. = ***Achillea oxyloba* subsp. *schurii*** (Sch.Bip.) Heimerl

*Achillea schurii* var. *polycephala* (Schur) Prodan & Nyár. = ***Achillea oxyloba* subsp. *schurii*** (Sch.Bip.) Heimerl

*Aconitum bernhardianum* Rchb. – ***Aconitum bucovinense*** Zapał.

*Aconitum beskidense* (Zapał.) Gáyer – ***Aconitum lasiocarpum*** subsp. ***kotulae*** (Pawł.) Starm. & Mitka

***Aconitum bucovinense*** Zapał.*

*Aconitum bucovinense* f. *orthotricha* Gáyer = ***Aconitum bucovinense*** Zapał.

*Aconitum callibotryon* subsp. *bucovinense* (Zapał.) Grinţ. ≡ ***Aconitum bucovinense*** Zapał.

*Aconitum callibotryon* subsp. *bucovinense* f. *altum* Grinţ. = ***Aconitum bucovinense*** Zapał.

*Aconitum callibotryon* subsp. *bucovinense* f. *densum* Grinţ. = ***Aconitum bucovinense*** Zapał.

*Aconitum callibotryon* subsp. *bucovinense* f. *glaberrimum* Grinţ. = ***Aconitum bucovinense*** Zapał.

*Aconitum callibotryon* subsp. *bucovinense* f. *laxum* Grinţ. = ***Aconitum bucovinense*** Zapał.

*Aconitum callibotryon* subsp. *bucovinense* f. *pilosum* Grinţ. = ***Aconitum bucovinense*** Zapał.

*Aconitum callibotryon* subsp. *bucovinense* f. *pyramidatum* Grinţ. = ***Aconitum bucovinense*** Zapał.

*Aconitum callibotryon* subsp. *rigidum* (Rchb.) Grinţ. = ***Aconitum bucovinense*** Zapał.

*Aconitum callibotryon* subsp. *rigidum* f. *glabrum* Grinţ. = ***Aconitum bucovinense*** Zapał.

*Aconitum callibotryon* subsp. *rigidum* f. *pubescens* Grinţ. = ***Aconitum bucovinense*** Zapał.

*Aconitum cammarum* [unranked] a *beskidense* Zapał. – ***Aconitum lasiocarpum*** subsp. ***kotulae*** (Pawł.) Starm. & Mitka

*Aconitum cammarum* [unranked] c *koscieliskanum* Zapał. – ***Aconitum lasiocarpum*** subsp. ***kotulae*** (Pawł.) Starm. & Mitka

*Aconitum carpaticum* (DC.) Sagorski & Schneider [p.p.] – ***Aconitum moldavicum*** subsp. ***moldavicum*** Hacq. ex Rchb.

*Aconitum commutatum* Rchb. = ***Aconitum bucovinense*** Zapał.

*Aconitum dasycarpum* (Schur) Schur ex Gáyer = ***Aconitum lasiocarpum*** subsp. ***lasiocarpum*** (Rchb.) Gáyer

*Aconitum degenii* f. *craciunelense* Gáyer = ***Aconitum degenii*** subsp. ***degenii*** Gáyer

***Aconitum degenii*** subsp. ***degenii*** Gáyer *

*Aconitum fallacinum* Błocki [p.p.] – ***Aconitum moldavicum*** subsp. ***moldavicum*** Hacq. ex Rchb.

*Aconitum firmum* f. *rigidum* (Rchb.) Gáyer = ***Aconitum bucovinense*** Zapał.

*Aconitum firmum* subsp. *bucovinense* (Zapał.) Aschers. & Graebn. ≡ ***Aconitum bucovinense*** Zapał.

***Aconitum firmum*** subsp. ***firmum*** Rchb.*

***Aconitum firmum*** subsp. ***fissurae*** Nyár.

*Aconitum flerovii* Steinb. in Komarov = ***Aconitum firmum*** subsp. ***fissurae*** Nyár.

*Aconitum gracile* subsp. *grosserratum* f. *beskidense* (Zapał.) Grinț. – ***Aconitum lasiocarpum*** subsp. ***kotulae*** (Pawł.) Starm. & Mitka

*Aconitum hebegynum* auct. fl. carpat., non DC. [p.p.] * – ***Aconitum degenii*** subsp. ***degenii*** Gáyer

*Aconitum hosteanum* f. *borbasii* Gáyer *=* ***Aconitum moldavicum*** subsp. ***hosteanum*** (Schur) Graebn. & P.Graebn.

*Aconitum hosteanum* Schur * *≡* ***Aconitum moldavicum*** subsp. ***hosteanum*** (Schur) Graebn. & P.Graebn.

*Aconitum hosteanum* var. *geraniifolium* Grinț. in Săvul. = ***Aconitum moldavicum*** subsp. ***hosteanum*** (Schur) Graebn. & P.Graebn.

*Aconitum hunyadense* Degen = ***Aconitum firmum*** subsp. ***fissurae*** Nyár.

*Aconitum jacquinianum* Host [quoad pl.carpat.] – ***Aconitum moldavicum*** subsp. ***moldavicum*** Hacq. ex Rchb.

*Aconitum koelleanum* var. *firmum* (Rchb.) Rchb. ≡ ***Aconitum firmum*** subsp. ***firmum*** Rchb.

*Aconitum laetum* [unranked] β *rigidum* Rchb. = ***Aconitum bucovinense*** Zapał.

*Aconitum lasiocarpum* Rchb. [p.p.] – ***Aconitum lasiocarpum*** subsp. ***lasiocarpum*** (Rchb.) Gáyer

*Aconitum lasiocarpum* Rchb. [p.p.] – ***Aconitum lasiocarpum*** subsp. ***kotulae*** (Pawł.) Starm. & Mitka

***Aconitum lasiocarpum*** subsp. ***kotulae*** (Pawł.) Starm. & Mitka *

***Aconitum lasiocarpum*** subsp. ***lasiocarpum*** (Rchb.) Gáyer *

*Aconitum lycoctonum* subsp. *moldavicum* (Hacq.) Jalas *≡* ***Aconitum moldavicum*** subsp. ***moldavicum*** Hacq. ex Rchb.

*Aconitum lycoctonum* [unranked] β *caeruleum* Wahlenb. = ***Aconitum moldavicum*** subsp. ***moldavicum*** Hacq. ex Rchb.

*Aconitum lycoctonum* subsp. *carpaticum* (DC.) Dostal [p.p.] – ***Aconitum moldavicum*** subsp. ***moldavicum*** Hacq. ex Rchb.

*Aconitum lycoctonum* var. *carpaticum* (DC.) Ser. [p.p.] – ***Aconitum moldavicum*** subsp. ***moldavicum*** Hacq. ex Rchb.

*Aconitum moldavicum* Hacq. [p.p.major] – ***Aconitum moldavicum*** subsp. ***moldavicum*** Hacq. ex Rchb.

*Aconitum moldavicum* Hacq. [p.p.] – ***Aconitum moldavicum*** subsp. ***hosteanum*** (Schur) Graebn. & P.Graebn.

*Aconitum moldavicum* Hacq. ex Rchb. [p.p.major] – ***Aconitum moldavicum*** subsp. ***moldavicum*** Hacq. ex Rchb.

*Aconitum moldavicum* Hacq. ex Rchb. [p.p.] – ***Aconitum moldavicum*** subsp. ***hosteanum*** (Schur) Graebn. & P.Graebn.

*Aconitum moldavicum* [unranked] a *dissectifolium* Zapał. = ***Aconitum moldavicum*** subsp. ***hosteanum*** (Schur) Graebn. & P.Graebn.

*Aconitum moldavicum* [unranked] b *grandicassum* Zapał. = ***Aconitum moldavicum*** subsp. ***hosteanum*** (Schur) Graebn. & P.Graebn.

*Aconitum moldavicum* [unranked] c *grandiflorum* Schur *=* ***Aconitum moldavicum*** subsp. ***hosteanum*** (Schur) Graebn. & P.Graebn.

*Aconitum moldavicum* [unranked] c *parvicassum* Zapał. [p.p.] – ***Aconitum moldavicum*** subsp. ***moldavicum*** Hacq. ex Rchb.

*Aconitum moldavicum* [unranked] d *leopoliense* Zapał. = ***Aconitum moldavicum*** subsp. ***hosteanum*** (Schur) Graebn. & P.Graebn.

*Aconitum moldavicum* [unranked] e *hosteanum* (Schur) Zapał. ≡ ***Aconitum moldavicum*** subsp. ***hosteanum*** (Schur) Graebn. & P.Graebn.

*Aconitum moldavicum* [unranked] e *hosteanum* f. *czywczynense* Zapał. = ***Aconitum moldavicum*** subsp. ***hosteanum*** (Schur) Graebn. & P.Graebn.

*Aconitum moldavicum* [unranked] e *hosteanum* f. *rodnense* Zapał. = ***Aconitum moldavicum*** subsp. ***hosteanum*** (Schur) Graebn. & P.Graebn.

*Aconitum moldavicum* f. *puberulum* Zapał. [p.p.] – ***Aconitum moldavicum*** subsp. ***moldavicum*** Hacq. ex Rchb.

*Aconitum moldavicum* f. *stenanthum* Gáyer = ***Aconitum moldavicum*** subsp. ***moldavicum*** Hacq. ex Rchb.

*Aconitum moldavicum* subsp. *hacquetianum* Grinț. [nom. nudum] = ***Aconitum moldavicum*** subsp. ***moldavicum*** Hacq. ex Rchb.

***Aconitum moldavicum*** subsp. ***hosteanum*** (Schur) Graebn. & P.Graebn.*

***Aconitum moldavicum*** subsp. ***moldavicum*** Hacq. ex Rchb.*

*Aconitum moldavicum* var. *australe* (Rchb.) Grinț. in Săvul. [p.p.] – ***Aconitum moldavicum*** subsp. ***hosteanum*** (Schur) Graebn. & P.Graebn.

*Aconitum moldavicum* var. *australe* f. *dissectifolium* (Zapał.) Grinț. in Săvul. = ***Aconitum moldavicum*** subsp. ***hosteanum*** (Schur) Graebn. & P.Graebn.

*Aconitum moldavicum* var. *australe* f. *fragile* Grinț. in Săvul. = ***Aconitum moldavicum*** subsp. ***hosteanum*** (Schur) Graebn. & P.Graebn.

*Aconitum moldavicum* var. *australe* f. *grandiflorum* (Schur) Grinț. in Săvul. = ***Aconitum moldavicum*** subsp. ***hosteanum*** (Schur) Graebn. & P.Graebn.

*Aconitum moldavicum* var. *australe* f. *leopoliensis* (Zapał.) Grinț. in Săvul. = ***Aconitum moldavicum*** subsp. ***hosteanum*** (Schur) Graebn. & P.Graebn.

*Aconitum moldavicum* var. *australe* f. *obtusidentatum* Simonk. ex Gáyer *=* ***Aconitum moldavicum*** subsp. ***hosteanum*** (Schur) Graebn. & P.Graebn.

*Aconitum moldavicum* var. *australe* f. *thyraicum* (Błocki) Grinț. in Săvul. = ***Aconitum moldavicum*** subsp. ***hosteanum*** (Schur) Graebn. & P.Graebn.

*Aconitum moldavicum* var. *hacquetianum* f. *flexuosum* Grinț. in Săvul. = ***Aconitum moldavicum*** subsp. ***moldavicum*** Hacq. ex Rchb.

*Aconitum moldavicum* var. *hacquetianum* f. *macrocassis* Grinț. in Săvul. = ***Aconitum moldavicum*** subsp. ***moldavicum*** Hacq. ex Rchb.

*Aconitum moldavicum* var. *hacquetianum* f. *piliferum* Grinț. in Săvul. = ***Aconitum moldavicum*** subsp. ***moldavicum*** Hacq. ex Rchb.

*Aconitum moldavicum* var. *hacquetianum* Grinț. in Săvul. = ***Aconitum moldavicum*** subsp. ***moldavicum*** Hacq. ex Rchb.

*Aconitum moldavicum* var. *rubicundum* Borbás = ***Aconitum moldavicum*** subsp. ***moldavicum*** Hacq. ex Rchb.

*Aconitum molle* Rchb. = ***Aconitum degenii*** subsp. ***degenii*** Gáyer

*Aconitum napellus* f. *commutatum* (Rchb.) Gáyer in G.Hegi = ***Aconitum bucovinense*** Zapał.

*Aconitum napellus* subsp. *firmum* (Rchb.) Gáyer in G.Hegi ≡ ***Aconitum firmum*** subsp. ***firmum*** Rchb.

*Aconitum napellus* subsp. *fissurae* (Nyár.) W.Seitz ≡ ***Aconitum firmum*** subsp. ***fissurae*** Nyár.

*Aconitum napellus* [unranked] b *subtatrense* = ***Aconitum firmum*** subsp. ***firmum*** Rchb.

*Aconitum napellus* [unranked] b *subtatrense* f. *abnorme* Zapał. = ***Aconitum firmum*** subsp. ***firmum*** Rchb.

*Aconitum napellus* [unranked] b *subtatrense* f. *latisectum* Zapał. = ***Aconitum firmum*** subsp. ***firmum*** Rchb.

*Aconitum napellus* [unranked] b *subtatrense* f. *subtatrense* Zapał. = ***Aconitum firmum*** subsp. ***firmum*** Rchb.

*Aconitum napellus* [unranked] d *carpaticum* f. *carpaticum* Zapał. = ***Aconitum firmum*** subsp. ***firmum*** Rchb.

*Aconitum napellus* [unranked] e *babiogorense* f. *babiogorense* Zapał. = ***Aconitum firmum*** subsp. ***firmum*** Rchb.

*Aconitum napellus* [unranked] e *babiogorense* f. *subfissum* Zapał. = ***Aconitum firmum*** subsp. ***firmum*** Rchb.

*Aconitum napellus* [unranked] e *babiogorense* Zapał. = ***Aconitum firmum*** subsp. ***firmum*** Rchb.

*Aconitum napellus* [unranked] g *tatrense* Zapał. = ***Aconitum firmum*** subsp. ***firmum*** Rchb.

*Aconitum napellus* var. *firmum* (Rchb.) Pawł. ≡ ***Aconitum firmum*** subsp. ***firmum*** Rchb.

*Aconitum nasutum* var. *lasiocarpum* Rchb. ≡ ***Aconitum lasiocarpum*** subsp. ***lasiocarpum*** (Rchb.) Gáyer

*Aconitum palmatifidum* Rchb. [p.p.] – ***Aconitum firmum*** subsp. ***firmum*** Rchb.

*Aconitum paniculatum* [unanked] e *podolicum* f. *latilobum* Zapał. = ***Aconitum lasiocarpum*** subsp. ***kotulae*** (Pawł.) Starm. & Mitka

*Aconitum paniculatum* [unanked] e *podolicum* Zapał. = ***Aconitum lasiocarpum*** subsp. ***kotulae*** (Pawł.) Starm. & Mitka

*Aconitum paniculatum* [unranked] a *percalabense* Zapał. = ***Aconitum degenii*** subsp. ***degenii*** Gáyer

*Aconitum paniculatum* [unranked] b *czeremossicum* Zapał. = ***Aconitum degenii*** subsp. ***degenii*** Gáyer

*Aconitum paniculatum* [unranked] c *prutense* f. *lobatum* Zapał. = ***Aconitum degenii*** subsp. ***degenii*** Gáyer

*Aconitum paniculatum* [unranked] c *prutense* f. *subintermedium* Zapał. = ***Aconitum degenii*** subsp. ***degenii*** Gáyer

*Aconitum paniculatum* [unranked] c *prutense* Zapał. = ***Aconitum degenii*** subsp. ***degenii*** Gáyer

*Aconitum paniculatum* [unranked] d *intermedium* Zapał. = ***Aconitum degenii*** subsp. ***degenii*** Gáyer

*Aconitum paniculatum* f. *latiusculum* Zapał. = ***Aconitum degenii*** subsp. ***degenii*** Gáyer

*Aconitum paniculatum* f. *tenuifissum* Zapał. = ***Aconitum degenii*** subsp. ***degenii*** Gáyer

*Aconitum paniculatum* Lam. [p.p., nom. inval.] * – ***Aconitum degenii*** subsp. ***degenii*** Gáyer

*Aconitum paniculatum* subsp. *lasiocarpum* (Rchb.) Soó *≡* ***Aconitum lasiocarpum*** subsp. ***lasiocarpum*** (Rchb.) Gáyer

*Aconitum podolicum* (Zapał.) Voroshylov * = ***Aconitum lasiocarpum*** subsp. ***kotulae*** (Pawł.) Starm. & Mitka

*Aconitum prutense* (Zapał.) Tzvelev = ***Aconitum degenii*** subsp. ***degenii*** Gáyer

*Aconitum romanicum* Woł.* = ***Aconitum firmum*** subsp. ***fissurae*** Nyár.

*Aconitum septentrionale* [unranked] β *carpathicum* DC. [p.p.] – ***Aconitum moldavicum*** subsp. ***moldavicum*** Hacq. ex Rchb.

*Aconitum septentrionale* Baumg., non Koelle = ***Aconitum moldavicum*** subsp. ***moldavicum*** Hacq. ex Rchb.

*Aconitum skerisorae* auct [e.g., Seitz, Soó], non Gáyer * – ***Aconitum firmum*** subsp. ***firmum*** Rchb.

*Aconitum tatrae* Borbás. in Pallas [p.p.] – ***Aconitum firmum*** subsp. ***firmum*** Rchb.

*Aconitum tatrae* subsp. *hunyadense* (Degen) Soó = ***Aconitum firmum*** subsp. ***fissurae*** Nyár.

*Aconitum tauricum* auct. fl. carpat., non Wulfen – ***Aconitum firmum*** subsp. ***firmum*** Rchb.

*Aconitum thyraicum* Błocki * *=* ***Aconitum moldavicum*** subsp. ***hosteanum*** (Schur) Graebn. & P.Graebn.

*Aconitum toxicum* [unranked] a *dasycarpum* Schur = ***Aconitum lasiocarpum*** subsp. ***lasiocarpum*** (Rchb.) Gáyer

*Aconitum toxicum* subsp. *lasiocarpum* (Rchb.) Grinț. ≡ ***Aconitum lasiocarpum*** subsp. ***lasiocarpum*** (Rchb.) Gáyer

*Aconitum transilvanicum* Lerchenf. ex Schur. = ***Aconitum moldavicum*** subsp. ***moldavicum*** Hacq. ex Rchb.

*Aconitum vagneri* Kern. ex Gáyer = ***Aconitum lasiocarpum*** subsp. ***lasiocarpum*** (Rchb.) Gáyer

*Aconitum variegatum* f. *kotulae* (Pawł.) Skalický *≡* ***Aconitum lasiocarpum*** subsp. ***kotulae*** (Pawł.) Starm. & Mitka

*Aconitum variegatum* subsp. *kotulae* Pawł. ≡ ***Aconitum lasiocarpum*** subsp. ***kotulae*** (Pawł.) Starm. & Mitka

*Alopecurus brachystachyus* auct. [e.g., Janka], non M.Bieb. – ***Alopecurus pratensis*** subsp. ***laguriformis*** (Schur) Tzvelev

*Alopecurus laguriformis* [unranked] a *abbreviatus* Schur = ***Alopecurus pratensis*** subsp. ***laguriformis*** (Schur) Tzvelev

*Alopecurus laguriformis* [unranked] b *elongatus* Schur = ***Alopecurus pratensis*** subsp. ***laguriformis*** (Schur) Tzvelev

*Alopecurus laguriformis* Schur [nom. nudum] * *≡* ***Alopecurus pratensis*** subsp. ***laguriformis*** (Schur) Tzvelev

*Alopecurus laguriformis* Schur ex Gris. ≡ ***Alopecurus pratensis*** subsp. ***laguriformis*** (Schur) Tzvelev

***Alopecurus pratensis*** subsp. ***laguriformis*** (Schur) Tzvelev

*Alopecurus transsilvanicus* Schur = ***Alopecurus pratensis*** subsp. ***laguriformis*** (Schur) Tzvelev

*Alsine gerardii* auct. flora carpat., non Willd. – ***Sabulina pauciflora*** (Kit.) A.Novikov, *comb. nov.*

*Alsine oxypetala* Woł. ≡ ***Sabulina oxypetala*** (Woł.) Mosyakin & Fedor.

*Alsine pauciflora* Kit. ex Nyman *≡* ***Sabulina pauciflora*** (Kit.) A.Novikov, *comb. nov.*

*Alsine verna* [unranked] a. *zarencnyi* (Zapał.) Hermann = ***Sabulina pauciflora*** (Kit.) A.Novikov, *comb. nov.*

*Alsine verna* [unranked] *oxypetala* Zapał. [nom. inval. ?] *≡* ***Sabulina oxypetala*** (Woł.) Mosyakin & Fedor.

*Alsine verna* [unranked] δ *carpatica* Porcius = ***Sabulina pauciflora*** (Kit.) A.Novikov, *comb. nov.*

*Alsine verna* auct. fl. carpat., non (L.) Wahlenb. nec Bartl * – ***Sabulina pauciflora*** (Kit.) A.Novikov, *comb. nov.*

*Alsine verna* Knapp [p.p.], non Wahlenb.s.str. – ***Sabulina pauciflora*** (Kit.) A.Novikov, *comb. nov.*

*Alsine zarencznyi* [unranked] c *oxypetala* f. *acutissima* Zapał. = ***Sabulina oxypetala*** (Woł.) Mosyakin & Fedor.

*Alsine zarencznyi* [unranked] c *oxypetala* f. *micropetala* Zapał. = ***Sabulina oxypetala*** (Woł.) Mosyakin & Fedor.

*Alsine zarencznyi* var. *divestita* Zapał. = ***Sabulina pauciflora*** (Kit.) A.Novikov, *comb. nov.*

*Alsine zarencznyi* var. *neglecta* f. *ramificans* Zapał. = ***Sabulina oxypetala*** (Woł.) Mosyakin & Fedor.

*Alsine zarencznyi* var. *neglecta* f. *subcaespitosa* Zapał. = ***Sabulina oxypetala*** (Woł.) Mosyakin & Fedor.

*Alsine zarencznyi* var. *neglecta* f. *subcolorata* Zapał. = ***Sabulina oxypetala*** (Woł.) Mosyakin & Fedor.

*Alsine zarencznyi* var. *neglecta* Zapał. = ***Sabulina oxypetala*** (Woł.) Mosyakin & Fedor.

*Alsine zarencznyi* var. *oxypetala* Woł. ≡ ***Sabulina oxypetala*** (Woł.) Mosyakin & Fedor.

*Alsine zarencznyi* var. *pseudogerardiana* Zapał. = ***Sabulina pauciflora*** (Kit.) A.Novikov, *comb. nov.*

*Alsine zarencznyi* var. *zarencznyi* f. *bryophila* Zapał. = ***Sabulina pauciflora*** (Kit.) A.Novikov, *comb. nov.*

*Alsine zarencznyi* var. *zarencznyi* f. *minima* Zapał. = ***Sabulina pauciflora*** (Kit.) A.Novikov, *comb. nov.*

*Alsine zarencznyi* var. *zarencznyi* f. *paucicaulis* Zapał. = ***Sabulina pauciflora*** (Kit.) A.Novikov, *comb. nov.*

*Alsine zarencznyi* var. *zarencznyi* f. *subpurpuea* Zapał. = ***Sabulina pauciflora*** (Kit.) A.Novikov, *comb. nov.*

*Alsine zarencznyi* var. *zarencznyi* f. *supraglandulosa* Zapał. = ***Sabulina pauciflora*** (Kit.) A.Novikov, *comb. nov.*

*Alsine zarencznyi* Zapał. [excl. var. c] = ***Sabulina pauciflora*** (Kit.) A.Novikov, *comb. nov.*

*Amphigenes carpathica* (F.Dietr.) Janka *≡* ***Festuca carpatica*** F.Dietr.

*Amphigenes carpathica* (F.Dietr.) Janka ex Hack. ≡ ***Festuca carpatica*** F.Dietr.

*Amphigenes nutans* (Wahlenb.) Janka = ***Festuca carpatica*** F.Dietr.

*Antennaria alpina* auct fl. carpat. [e.g., Baumg.; Schur], non (L.) Gaertn. – ***Antennaria carpatica*** subsp. ***carpatica*** (Wahlenb.) Bluff & Fingerh.

*Antennaria alpina* Ledeb. [p.p., tantum quod plantas ucrain. carpat.], non (L.) Gaertn. – ***Antennaria carpatica*** subsp. ***carpatica*** (Wahlenb.) Bluff & Fingerh.

*Antennaria carpatica* (Wahlenb.) Hook. ≡ ***Antennaria carpatica*** subsp. ***carpatica*** (Wahlenb.) Bluff & Fingerh.

*Antennaria carpatica* (Wahlenb.) R.Br. [nom. inval.] * *≡* ***Antennaria carpatica*** subsp. ***carpatica*** (Wahlenb.) Bluff & Fingerh.

*Antennaria carpatica* (Wahlenb.) Hook. in Bluff & Fingerh. ≡ ***Antennaria carpatica*** subsp. ***carpatica*** (Wahlenb.) Bluff & Fingerh.

***Antennaria carpatica*** subsp. ***carpatica*** (Wahlenb.) Bluff & Fingerh.*

*Antennaria carpatica* Trautv. [p.p.] – ***Antennaria carpatica*** subsp. ***carpatica*** (Wahlenb.) Bluff & Fingerh.

*Anthemis alpina* Baumg., non L. = ***Achillea oxyloba* subsp. *schurii*** (Sch.Bip.) Heimerl

*Anthemis caespitosa* Herbich = ***Achillea oxyloba* subsp. *schurii*** (Sch.Bip.) Heimerl

*Anthemis oxyloba* Schur = ***Achillea oxyloba* subsp. *schurii*** (Sch.Bip.) Heimerl

*Anthemis pseudo-atrata* Schur ex Schur = ***Achillea oxyloba* subsp. *schurii*** (Sch.Bip.) Heimerl

*Anthemis schurii* Sch.Bip. ex Heimerl [nom. nudum] *≡* ***Achillea oxyloba* subsp. *schurii*** (Sch.Bip.) Heimerl

*Anthemis schurii* Sch.Bip. ≡ ***Achillea oxyloba* subsp. *schurii*** (Sch.Bip.) Heimerl

*Anthemis tenuifolia* (Schur) Schur. [nom. inval.], non *Achillea tenuifolia* Lam. ≡ ***Achillea oxyloba* subsp. *schurii*** (Sch.Bip.) Heimerl

Anthemis tenuifolia [unranked] a simplex monocephala Schur. ≡ ***Achillea oxyloba* subsp. *schurii*** (Sch.Bip.) Heimerl

Anthemis tenuifolia [unranked] b ramosa polycephala Schur. *≡* ***Achillea oxyloba* subsp. *schurii*** (Sch.Bip.) Heimerl

Anthemis tenuifolia [unranked] c pilosa minima polaris Schur. *≡* ***Achillea oxyloba* subsp. *schurii*** (Sch.Bip.) Heimerl

*Apargia aurea* Baumg., non (L.) F. W.Schmidt, non *Leontodon aureum* L., nec *Ceracium aureum* Schur. – ***Scorzoneroides pseudotaraxaci*** (Schur) Holub

*Apargia taraxaci* Wahlenb., non Willd. – ***Scorzoneroides pseudotaraxaci*** (Schur) Holub

***Arabidopsis neglecta*** (Schult.) O’Kane & Al-Shehbaz

*Arabis floribunda* Schur = ***Arabidopsis neglecta*** (Schult.) O’Kane & Al-Shehbaz

*Arabis glareosa* Schur = ***Arabidopsis neglecta*** (Schult.) O’Kane & Al-Shehbaz

*Arabis neglecta* Schult.* *≡* ***Arabidopsis neglecta*** (Schult.) O’Kane & Al-Shehbaz

*Arabis transsilvanica* Schur = ***Arabidopsis neglecta*** (Schult.) O’Kane & Al-Shehbaz

*Arenaria gerardii* auct. fl. carpat., non Willd. – ***Sabulina pauciflora*** (Kit.) A.Novikov, *comb. nov.*

*Arenaria pauciflora* Kit., non Prodan ≡ ***Sabulina pauciflora*** (Kit.) A.Novikov, *comb. nov.*

*Armeria elongata* auct., non (Hoffm.) Koch – ***Armeria pocutica*** Pawł.

*Armeria maritima* subsp. *elongata* auct., non (Hoffm.) Bonnier – ***Armeria pocutica*** Pawł.

***Armeria pocutica*** Pawł.

*Armeria vulgaris* auct., non Willd. – ***Armeria pocutica*** Pawł.

*Arnica scorpioides* Baumg., non alior – ***Doronicum carpaticum*** (Griseb. & Schenk) Nyman

*Aronicum barcense* Simonk. = ***Doronicum carpaticum*** (Griseb. & Schenk) Nyman

*Aronicum carpathicum* (Griseb. & Schenk) Fuss *≡* ***Doronicum carpaticum*** (Griseb. & Schenk) Nyman

*Aronicum carpathicum* (Griseb. & Schenk) Schur [ortho. var.] * *≡* ***Doronicum carpaticum*** (Griseb. & Schenk) Nyman

*Aronicum carpaticum* (Griseb. & Schenk) Schur * *≡* ***Doronicum carpaticum*** (Griseb. & Schenk) Nyman

*Aronicum carpaticum* [unranked] a *polyphyllum* Schur = ***Doronicum carpaticum*** (Griseb. & Schenk) Nyman

*Aronicum latifolium* Schur [nom. nudum], non Rchb. = ***Doronicum carpaticum*** (Griseb. & Schenk) Nyman

*Aronicum scorpioides* var. *carpaticum* Griseb. & Schenk in Wiegm. ≡ ***Doronicum carpaticum*** (Griseb. & Schenk) Nyman

*Asterocephalus* *lucidus* [unranked] a.*alpicolus* Schur = ***Scabiosa lucida*** subsp. ***barbata*** Nyár.

*Asterocephalus lucidus* [unranked] b.*subalpinus* Schur = ***Scabiosa lucida*** subsp. ***barbata*** Nyár.

*Avena carpatica* auct. [e.g., Błocki ex herb.], non Host – ***Trisetum fuscum*** (Kit. ex Schult.) Schult. in Roem. & Schult.

*Avena ciliaris* Kit. ex Schult. = ***Trisetum fuscum*** (Kit. ex Schult.) Schult. in Roem. & Schult.

*Avena fusca* Kit. ex Schult., non Ard. ≡ ***Trisetum fuscum*** (Kit. ex Schult.) Schult. in Roem. & Schult.

*Cammarum paniculatum* (Arcang.) Fourr. [p.p.] – ***Aconitum degenii*** subsp. ***degenii*** Gáyer

*Campanula arcuata* Schur = ***Campanula serrata*** (Kit. ex Schult.) Hendrych

*Campanula carnica* auct. fl. transsilv., non Schiede – ***Campanula tatrae* subsp. *tatrae*** Borbás

*Campanula carpatica* [unranked] *alba* (Voss) J.R.Duncan & V.C.Davies = ***Campanula carpatica*** Jacq., non *C. carpatha* Halácsy

*Campanula carpatica* [unranked] b *subpilosa* Schur = ***Campanula carpatica*** Jacq., non *C. carpatha* Halácsy

*Campanula carpatica* [unranked] *pelviformis* Froebel ex André = ***Campanula carpatica*** Jacq., non *C. carpatha* Halácsy

*Campanula carpatica* [unranked] *riverslea* J.R.Duncan & V.C.Davies = ***Campanula carpatica*** Jacq., non *C. carpatha* Halácsy

*Campanula carpatica* Baumg. ex Schur [nom. inval.] = ***Campanula carpatica*** Jacq., non *C. carpatha* Halácsy

*Campanula carpatica* L. ex Schur [nom. inval.] = ***Campanula carpatica*** Jacq., non *C. carpatha* Halácsy

*Campanula carpatica* f. *dasycarpa* (Schur) Tacik = ***Campanula carpatica*** Jacq., non *C. carpatha* Halácsy

*Campanula carpatica* f. *subpilosa* (Schur) Tacik = ***Campanula carpatica*** Jacq., non *C. carpatha* Halácsy

***Campanula carpatica*** Jacq., non *C. carpatha* Halácsy *

*Campanula* *carpatica* subsp. *turbinata* (Schott, Nyman & Kotschy) Nyman = ***Campanula carpatica*** Jacq., non *C. carpatha* Halácsy

*Campanula carpatica* var. *brachyphylla* Morariu = ***Campanula carpatica*** Jacq., non *C. carpatha* Halácsy

*Campanula carpatica* var. *dasycarpa* Schur = ***Campanula carpatica*** Jacq., non *C. carpatha* Halácsy

*Campanula carpatica* var. *grandiflora* Schur = ***Campanula carpatica*** Jacq., non *C. carpatha* Halácsy

*Campanula carpatica* var. *hemisphaerica* Schur = ***Campanula carpatica*** Jacq., non *C. carpatha* Halácsy

*Campanula carpatica* var. *hendersonii* (C.Wolley Dod) W.T.Mill. [hort.] – ***Campanula carpatica*** Jacq., non *C. carpatha* Halácsy

*Campanula carpatica* var. *longifolia* f. *parviflora* Săvul. ex Morariu & Nyár. = ***Campanula carpatica*** Jacq., non *C. carpatha* Halácsy

*Campanula carpatica* var. *longifolia* Morariu = ***Campanula carpatica*** Jacq., non *C. carpatha* Halácsy

*Campanula carpatica* var. *oreophila* Schur = ***Campanula carpatica*** Jacq., non *C. carpatha* Halácsy

*Campanula carpatica* var. *porrecta* f. *minor* Morariu = ***Campanula carpatica*** Jacq., non *C. carpatha* Halácsy

*Campanula carpatica* var. *porrecta* Morariu = ***Campanula carpatica*** Jacq., non *C. carpatha* Halácsy

*Campanula carpatica* var. *schuriana* Săvul. ex Morariu & Nyár. = ***Campanula carpatica*** Jacq., non *C. carpatha* Halácsy

*Campanula carpatica* var. *subdasycarpa* Morariu & Nyár. = ***Campanula carpatica*** Jacq., non *C. carpatha* Halácsy

*Campanula carpatica* var. *transsilvanica* Schur, non *Campanula transsilvanica* Schur ex Andrae = ***Campanula carpatica*** Jacq., non *C. carpatha* Halácsy

*Campanula carpatica* var. *tomentosa* Kotschy = ***Campanula carpatica*** Jacq., non *C. carpatha* Halácsy

*Campanula carpatica* var. *turbinata* (Schott, Nyman & Kotschy) Fuss = ***Campanula carpatica*** Jacq., non *C. carpatha* Halácsy

*Campanula carpatica* var. *turbinata* (Schott, Nyman & Kotschy) Nichols = ***Campanula carpatica*** Jacq., non *C. carpatha* Halácsy

*Campanula* *carpatica* var. *turbinata* f. *rotundata* Morariu = ***Campanula carpatica*** Jacq., non *C. carpatha* Halácsy

*Campanula consanguinea* Simonk. [p.p.], non Schott – ***Campanula tatrae* subsp. *tatrae*** Borbás

*Campanula cordifolia* Vuk., non K.Koch *≡* ***Campanula carpatica*** Jacq., non *C. carpatha* Halácsy

*Campanula dasycarpa* Fuss. ex Schur, non Kit. ex Schult. = ***Campanula carpatica*** Jacq., non *C. carpatha* Halácsy

*Campanula dasycarpa* Schur ex Schur [nom. illeg.], non Kit. ex Schult. = ***Campanula carpatica*** Jacq., non *C. carpatha* Halácsy

*Campanula fergusonii* A.M.Ferguson [pro hybr., hort] – ***Campanula carpatica*** Jacq., non *C. carpatha* Halácsy

*Campanula hendersonii* C.Wolley Dod [hort.] – ***Campanula carpatica*** Jacq., non *C. carpatha* Halácsy

*Campanula hornungiana* Schur = ***Campanula serrata*** (Kit. ex Schult.) Hendrych

*Campanula kitaibeliana* Roem. & Schult. = ***Campanula serrata*** (Kit. ex Schult.) Hendrych

***Campanula kladniana*** (Schur) Witasek *

*Campanula kladniana* (Schur) Witasek [p.p.min., non sensu Schur orig.] – ***Campanula tatrae* subsp. *tatrae*** Borbás

*Campanula kladniana* subsp. *polymorpha* Witasek = ***Campanula tatrae* subsp. *tatrae*** Borbás

*Campanula kladniana* subsp. *stenophylla* (Schur) Witasek = ***Campanula tatrae* subsp. *tatrae*** Borbás

*Campanula kladniana* var. *polymorpha* (Witasek) Pawł. = ***Campanula tatrae* subsp. *tatrae*** Borbás

*Campanula lanceolata* Neilr., non alior = ***Campanula serrata*** (Kit. ex Schult.) Hendrych

*Campanula lanceolata* subsp. *arcuata* (Schur) Simonk. = ***Campanula serrata*** (Kit. ex Schult.) Hendrych

*Campanula lanceolata* var. *hornungiana* (Schur) Simonk. = ***Campanula serrata*** (Kit. ex Schult.) Hendrych

*Campanula lancifolia* Schur [nom. illeg.] sensu Błocki, non Witasek – ***Campanula serrata*** (Kit. ex Schult.) Hendrych

*Campanula linifolia* auct. [e.g., Wahlenb.], non Jacq. – ***Campanula tatrae* subsp. *tatrae*** Borbás

*Campanula microphylla* Kit. ex Schult., non Cav. [nom. illeg.] = ***Campanula serrata*** (Kit. ex Schult.) Hendrych

*Campanula napuligera* f. *albiflora* Raclaru [nom. nudum] = ***Campanula serrata*** (Kit. ex Schult.) Hendrych

*Campanula napuligera* f. *angustifrons* Hruby = ***Campanula serrata*** (Kit. ex Schult.) Hendrych

*Campanula napuligera* f. *arcuata* (Schur) Hruby = ***Campanula serrata*** (Kit. ex Schult.) Hendrych

*Campanula napuligera* f. *genuina* Hruby = ***Campanula serrata*** (Kit. ex Schult.) Hendrych

*Campanula napuligera* f. *glabrescens* Hruby = ***Campanula serrata*** (Kit. ex Schult.) Hendrych

*Campanula napuligera* f. *humilis* Hruby = ***Campanula serrata*** (Kit. ex Schult.) Hendrych

*Campanula napuligera* f. in*termedia* Hruby = ***Campanula serrata*** (Kit. ex Schult.) Hendrych

*Campanula napuligera* f. *latifrons* Hruby = ***Campanula serrata*** (Kit. ex Schult.) Hendrych

*Campanula napuligera* f. *longisepala* (Nyár.) Morariu = ***Campanula serrata*** (Kit. ex Schult.) Hendrych

*Campanula napuligera* f. *minima* (Săvul.) Morariu = ***Campanula serrata*** (Kit. ex Schult.) Hendrych

*Campanula napuligera* f. *parvula* Morariu = ***Campanula serrata*** (Kit. ex Schult.) Hendrych

*Campanula napuligera* f. *robusta* Hruby = ***Campanula serrata*** (Kit. ex Schult.) Hendrych

*Campanula napuligera* f. *savulescui* Morariu = ***Campanula serrata*** (Kit. ex Schult.) Hendrych

*Campanula napuligera* f. *scheuzeriformis* (Nyár.) Morariu = ***Campanula serrata*** (Kit. ex Schult.) Hendrych

*Campanula napuligera* f. *semiamplexicaulis* (Vladescu & Săvul.) Morariu = ***Campanula serrata*** (Kit. ex Schult.) Hendrych

*Campanula napuligera* f. *setulosa* Morariu = ***Campanula serrata*** (Kit. ex Schult.) Hendrych

*Campanula napuligera* f. *simplex* Hruby = ***Campanula serrata*** (Kit. ex Schult.) Hendrych

*Campanula napuligera* f. *stenophylloides* Nyár. = ***Campanula serrata*** (Kit. ex Schult.) Hendrych

*Campanula napuligera* Schur * = ***Campanula serrata*** (Kit. ex Schult.) Hendrych

*Campanula napuligera* subf. *angustifrons* Hruby = ***Campanula serrata*** (Kit. ex Schult.) Hendrych

*Campanula napuligera* subf. *brachyantha* Hruby = ***Campanula serrata*** (Kit. ex Schult.) Hendrych

*Campanula napuligera* subf. *latifrons* Hruby = ***Campanula serrata*** (Kit. ex Schult.) Hendrych

*Campanula napuligera* subf. *tenella* Hruby = ***Campanula serrata*** (Kit. ex Schult.) Hendrych

*Campanula napuligera* var. *alpiniformis* Nyár. ex Morariu = ***Campanula serrata*** (Kit. ex Schult.) Hendrych

*Campanula napuligera* var. *elatior* (Săvul.) Morariu = ***Campanula serrata*** (Kit. ex Schult.) Hendrych

*Campanula napuligera* var. *hirsuta* Hruby = ***Campanula serrata*** (Kit. ex Schult.) Hendrych

*Campanula napuligera* var. *hornungiana* (Schur) Morariu = ***Campanula serrata*** (Kit. ex Schult.) Hendrych

*Campanula napuligera* var. *longisepala* Nyár. = ***Campanula serrata*** (Kit. ex Schult.) Hendrych

*Campanula napuligera* var. *redux* (Schott, Nyman & Kotschy) Hruby [nom. inval.] = ***Campanula serrata*** (Kit. ex Schult.) Hendrych

*Campanula napuligera* var. *redux* (Schott, Nyman & Kotschy) Nyman = ***Campanula serrata*** (Kit. ex Schult.) Hendrych

*Campanula napuligera* var. *savulescui* Morariu = ***Campanula serrata*** (Kit. ex Schult.) Hendrych

*Campanula napuligera* var. *scheuzeriformis* Nyár. = ***Campanula serrata*** (Kit. ex Schult.) Hendrych

*Campanula napuligera* var. *stenophylloides* (Nyár.) Morariu = ***Campanula serrata*** (Kit. ex Schult.) Hendrych

*Campanula napuligera* var. *stricta* Hruby = ***Campanula serrata*** (Kit. ex Schult.) Hendrych

*Campanula napuligera* var. *transsilvanica* (Săvul.) Morariu = ***Campanula serrata*** (Kit. ex Schult.) Hendrych

*Campanula napuligera* var. *umbrosa* Hruby = ***Campanula serrata*** (Kit. ex Schult.) Hendrych

*Campanula napuligera*.var. *arcuata* (Schur) Morariu = ***Campanula serrata*** (Kit. ex Schult.) Hendrych

*Campanula oreophila* Schur ex Schur = ***Campanula carpatica*** Jacq., non *C. carpatha* Halácsy

*Campanula polymorpha* (Witasek) Prain, non Banks & Sol. ex A.DC.* = ***Campanula tatrae* subsp. *tatrae*** Borbás

*Campanula polymorpha* f. *pseudolanceolata* (Pant.) Hruby = ***Campanula serrata*** (Kit. ex Schult.) Hendrych

*Campanula polymorpha* f. *sciaphila* Hruby [nom.et des. inval.] – ***Campanula tatrae* subsp. *tatrae*** Borbás

*Campanula polymorpha* var. in*tercedens* f. *angustifolia* Hruby = ***Campanula tatrae* subsp. *tatrae*** Borbás

*Campanula polymorpha* var. in*tercedens* f. ex*igua* Hruby = ***Campanula tatrae* subsp. *tatrae*** Borbás

*Campanula polymorpha* var. in*tercedens* f. *latifolia* Hruby = ***Campanula tatrae* subsp. *tatrae*** Borbás

*Campanula polymorpha* var. in*tercedens* f. *reflectans* Hruby = ***Campanula tatrae* subsp. *tatrae*** Borbás

*Campanula polymorpha* var. in*tercedens* f. *umbrosa* Hruby = ***Campanula tatrae* subsp. *tatrae*** Borbás

*Campanula polymorpha* var. in*tercedens* Hruby, non *C.witasekiana* var. in*tercedens* Hruby = ***Campanula tatrae* subsp. *tatrae*** Borbás

*Campanula polymorpha* var. *lepida* Nyár. ex Hruby = ***Campanula tatrae* subsp. *tatrae*** Borbás

*Campanula polymorpha* var. *pluriflora* Nyár. ex Hruby = ***Campanula tatrae* subsp. *tatrae*** Borbás

*Campanula polymorpha* var. *praticola* f. *hirta* (Nyár.) Hruby = ***Campanula tatrae* subsp. *tatrae*** Borbás

*Campanula polymorpha* var. *praticola* f. *pluriflora* (Nyár.) Hruby = ***Campanula tatrae* subsp. *tatrae*** Borbás

*Campanula polymorpha* var. *praticola* Hruby, non *C.witasekiana* var. *praticola* Hruby = ***Campanula tatrae* subsp. *tatrae*** Borbás

*Campanula polymorpha* var. *stenophylla* (Schur) Hruby = ***Campanula tatrae* subsp. *tatrae*** Borbás

*Campanula polymorpha* var. *stenophylla* f. *brachyphylla* Hruby = ***Campanula tatrae* subsp. *tatrae*** Borbás

*Campanula polymorpha* var. *stenophylla* f. *genuina* Hruby = ***Campanula tatrae* subsp. *tatrae*** Borbás

*Campanula polymorpha* var. *stenophylla* f. *gracilis* Hruby = ***Campanula tatrae* subsp. *tatrae*** Borbás

*Campanula polymorpha* var. *typica* f. *fasciculata* Nyár. ex Hruby = ***Campanula tatrae* subsp. *tatrae*** Borbás

*Campanula polymorpha* var. *typica* f. *fasciculata* subf. *deltoidea* Hruby = ***Campanula tatrae* subsp. *tatrae*** Borbás

*Campanula polymorpha* var. *typica* f. *kladnianioides* Nyárady ex Hruby = ***Campanula tatrae* subsp. *tatrae*** Borbás

*Campanula polymorpha* var. *typica* f. *latifolia* Hruby = ***Campanula tatrae* subsp. *tatrae*** Borbás

*Campanula polymorpha* var. *typica* f. *latifolia* subf. *umbrosa* Hruby = ***Campanula tatrae* subsp. *tatrae*** Borbás

*Campanula polymorpha* var. *typica* f. *lepida* (Nyár.) Hruby = ***Campanula tatrae* subsp. *tatrae*** Borbás

*Campanula polymorpha* var. *typica* f. *lepida* subf. *reflectans* Hruby = ***Campanula tatrae* subsp. *tatrae*** Borbás

*Campanula polymorpha* var. *typica* f. *saxiphila* Hruby = ***Campanula tatrae* subsp. *tatrae*** Borbás

*Campanula polymorpha* var. *typica* f. *saxiphila* subf. *reflectans* Hruby = ***Campanula tatrae* subsp. *tatrae*** Borbás

*Campanula polymorpha* var. *typica* Hruby = ***Campanula tatrae* subsp. *tatrae*** Borbás

*Campanula pseudocarpatica* Schur = ***Campanula carpatica*** Jacq., non *C. carpatha* Halácsy

*Campanula pseudolanceolata* f. *albiflora* Săvul. = ***Campanula serrata*** (Kit. ex Schult.) Hendrych

*Campanula pseudolanceolata* f. *elatior* Săvul. = ***Campanula serrata*** (Kit. ex Schult.) Hendrych

*Campanula pseudolanceolata* f. *minima* Săvul. = ***Campanula serrata*** (Kit. ex Schult.) Hendrych

*Campanula pseudolanceolata* f. *transsilvanica* Săvul. = ***Campanula serrata*** (Kit. ex Schult.) Hendrych

*Campanula pseudolanceolata* f. *umbraticola* Săvul. = ***Campanula serrata*** (Kit. ex Schult.) Hendrych

*Campanula pseudolanceolata* Pant.* = ***Campanula serrata*** (Kit. ex Schult.) Hendrych

*Campanula pseudolanceolata* Pant. ex A.Kern. = ***Campanula serrata*** (Kit. ex Schult.) Hendrych

*Campanula pseudolanceolata* subsp. *semiamplexicaulis* Vladescu & Săvul. = ***Campanula serrata*** (Kit. ex Schult.) Hendrych

*Campanula pseudolanceolata* var. *arcuata* (Schur) Porcius = ***Campanula serrata*** (Kit. ex Schult.) Hendrych

*Campanula pseudolanceolata* var. *hornungiana* (Schur) Porcius = ***Campanula serrata*** (Kit. ex Schult.) Hendrych

*Campanula pseudolanceolata* var. *porcii* Săvul. = ***Campanula serrata*** (Kit. ex Schult.) Hendrych

*Campanula pusilla* auct. fl. ucrain. carpat., non Haenke – ***Campanula tatrae* subsp. *tatrae*** Borbás

*Campanula redux* Schott, Nyman & Kotschy = ***Campanula serrata*** (Kit. ex Schult.) Hendrych

*Campanula reniformis* Schur = ***Campanula carpatica*** Jacq., non *C. carpatha* Halácsy

*Campanula rhomboidalis* subsp. *pseudolanceolata* (Pant.) Nyman = ***Campanula serrata*** (Kit. ex Schult.) Hendrych

*Campanula rhomboidalis* var. *angustifolia* Neilr. = ***Campanula serrata*** (Kit. ex Schult.) Hendrych

*Campanula rhomboidea* [unranked] β *foliis ovato-oblongis* Wahlenberg, non L. = ***Campanula serrata*** (Kit. ex Schult.) Hendrych

*Campanula rotundifolia* L. [p.p. minor, tantum quod plantas ucrain. carpat.], non alior * – ***Campanula tatrae* subsp. *tatrae*** Borbás

*Campanula rotundifolia* var. *arcuata* (Schur) Nyman = ***Campanula serrata*** (Kit. ex Schult.) Hendrych

*Campanula rotundifolia* var. *dentata* Schur, non N.Coleman = ***Campanula serrata*** (Kit. ex Schult.) Hendrych

*Campanula rotundifolia* var. *grandiflora* J.A.Knapp [nom. illeg.], non alior = ***Campanula serrata*** (Kit. ex Schult.) Hendrych

*Campanula rotundifolia* subsp. *kladniana* (Schur) Tacik in Pawłowski & Jasiewicz *≡* ***Campanula kladniana*** (Schur) Witasek

*Campanula rotundifolia* subsp. *polymorpha* (Witasek) Tacik in Jasiewicz = ***Campanula tatrae* subsp. *tatrae*** Borbás

*Campanula rotundifolia* var. *alpina* Schur [nom. illeg.], non Tuck. = ***Campanula serrata*** (Kit. ex Schult.) Hendrych

*Campanula scheuchzeri* [unranked] β *dacica* Porcius = ***Campanula tatrae* subsp. *tatrae*** Borbás

*Campanula scheuchzeri* auct. [e.g., Reuss, Sagorski & Schneider], non Vill.* – ***Campanula tatrae* subsp. *tatrae*** Borbás

*Campanula scheuchzeri* var. *dacica* Porcius = ***Campanula tatrae* subsp. *tatrae*** Borbás

*Campanula scheuchzeri* var. *kladniana* Schur *≡* ***Campanula kladniana*** (Schur) Witasek

*Campanula scheuchzeri* var. *stenophylla* Schur = ***Campanula tatrae* subsp. *tatrae*** Borbás

***Campanula serrata*** (Kit. ex Schult.) Hendrych *

*Campanula serrata* var. *elatior* (Săvul.) Tasenkevych [nom.provis.et inval., ex herb. LWS] * – ***Campanula serrata*** (Kit. ex Schult.) Hendrych

*Campanula serrata* var. *elatior* f. *latifrons* Hruby [comb. inval. ex herb. CHER] * – ***Campanula serrata*** (Kit. ex Schult.) Hendrych

*Campanula serrata* var. *hornungiana* (Schur.) Tasenkevych [nom.provis.et inval., ex herb. LWS] – ***Campanula serrata*** (Kit. ex Schult.) Hendrych

*Campanula stenophylla* (Schur) Prain [nom. inval.] = ***Campanula tatrae* subsp. *tatrae*** Borbás

*Campanula stenophylla* (Schur) Witasek, non Boiss. & Heldr. = ***Campanula tatrae* subsp. *tatrae*** Borbás

***Campanula tatrae* subsp. *tatrae*** Borbás*

Campanula trans[s]ilvanica Schur (1859), non *C. transsilvanica* Schur ex Andrae (1855) nec Schur (1866) – ***Campanula carpatica*** Jacq., non *C. carpatha* Halácsy

*Campanula turbinata* Schott, Nyman & Kotschy = ***Campanula carpatica*** Jacq., non *C. carpatha* Halácsy

*Campanula turbinata* f. *alba* Voss = ***Campanula carpatica*** Jacq., non *C. carpatha* Halácsy

*Campanula turbinata* f. *lilacina* Voss = ***Campanula carpatica*** Jacq., non *C. carpatha* Halácsy

*Campanula turbinata* f. *pelviformis* (Froebel ex André) Voss = ***Campanula carpatica*** Jacq., non *C. carpatha* Halácsy

*Cardamine enneaphyllos* Turcz. [nom. inval., ex herb.KW], non (L.) Crantz ex Crantz * – ***Arabidopsis neglecta*** (Schult.) O’Kane & Al-Shehbaz

***Cardamine glanduligera*** O.Schwarz

*Cardamine glandulosa* (Waldst. & Kit.) Schmalh. [nom. illeg.], non Blanco * ≡ ***Cardamine glanduligera*** O.Schwarz

*Cardaminopsis neglecta* (Schult.) Hayek * *≡* ***Arabidopsis neglecta*** (Schult.) O’Kane & Al-Shehbaz

*Cardaminopsis neglecta* subsp. *glareosa* (Schur) Soó = ***Arabidopsis neglecta*** (Schult.) O’Kane & Al-Shehbaz

*Centaurea carpatica* (Porcius) Formánek *≡* ***Centaurea phrygia*** subsp. ***carpatica*** (Porcius) Dostál

*Centaurea carpatica* (Porcius) Porcius * *≡* ***Centaurea phrygia*** subsp. ***carpatica*** (Porcius) Dostál

*Centaurea carpatica* (Porcius) Wagner ≡ ***Centaurea phrygia*** subsp. ***carpatica*** (Porcius) Dostál

***Centaurea maramarosiensis*** (Jáv.) Czerep.*

*Centaurea mollis* f. *maramarosiensis* Jáv. ≡ ***Centaurea maramarosiensis*** (Jáv.) Czerep.

*Centaurea mollis* f. *ramosa* Czakó in Jáv., non *Centaurea ramosa* (Gugler) Hayek = ***Centaurea maramarosiensis*** (Jáv.) Czerep.

*Centaurea mollis* subsp. *marmarosiensis* (Jáv.) Soó *≡* ***Centaurea maramarosiensis*** (Jáv.) Czerep.

*Centaurea montana* subsp. *maramarosiensis* (Jáv.) Soják *≡* ***Centaurea maramarosiensis*** (Jáv.) Czerep.

*Centaurea montana* subsp. *mollis* (Waldst. & Kit.) Gugler sensu Katina – ***Centaurea phrygia*** subsp. ***carpatica*** (Porcius) Dostál

*Centaurea montana* subsp. *mollis* var. *ramosa* (Czakó) Dostál, non *Centaurea ramosa* (Gugler) Hayek = ***Centaurea maramarosiensis*** (Jáv.) Czerep.

*Centaurea montana* subsp. *mollis* var. *typica* f. *maramarosiensis* (Jáv.) Dostál *≡* ***Centaurea maramarosiensis*** (Jáv.) Czerep.

***Centaurea phrygia*** subsp. ***carpatica*** (Porcius) Dostál

*Centaurea plumosa* var. *carpatica* Porcius [nom. inval.] *≡* ***Centaurea phrygia*** subsp. ***carpatica*** (Porcius) Dostál

*Centaurea plumosa* β [unranked] *polycephala* Porcius *=* ***Centaurea phrygia*** subsp. ***carpatica*** (Porcius) Dostál

*Centaurea pseudophrygia* f. in*tercedens* subf. *carpatica* (Porcius) Gugler *≡* ***Centaurea phrygia*** subsp. ***carpatica*** (Porcius) Dostál

*Centaurea rodnensis* Simonk.* *=* ***Centaurea phrygia*** subsp. ***carpatica*** (Porcius) Dostál

*Chamaezelum carpaticum* (Wahlenb.) Link *≡* ***Antennaria carpatica*** subsp. ***carpatica*** (Wahlenb.) Bluff & Fingerh.

*Chrysanthemum montanum* Csató, non alior – ***Leucanthemum rotundifolium*** (Waldst. & Kit. in Willd.) DC., non Opiz

*Chrysanthemum rotundifolium* Waldst. & Kit.* *≡* ***Leucanthemum rotundifolium*** (Waldst. & Kit. in Willd.) DC., non Opiz

*Chrysanthemum rotundifolium* Waldst. & Kit. in Willd. ≡ ***Leucanthemum rotundifolium*** (Waldst. & Kit. in Willd.) DC., non Opiz

***Chrysosplenium alpinum*** Schur *

*Chrysosplenium glaciale* Fuss * = ***Chrysosplenium alpinum*** Schur

*Chrysosplenium oppositifolium* auct. fl. roman.et ucrain. [e.g., Baumg.], non L.* – ***Chrysosplenium alpinum*** Schur

*Chrysosplenium oppositifolium* var. *alpinum* Schur * *≡* ***Chrysosplenium alpinum*** Schur

*Chrysosplenium oppositifolium* var. rosulare (Schot) Schott ex Engl. = ***Chrysosplenium alpinum*** Schur

*Chrysosplenium rosulare* Schott ex Maxim. = ***Chrysosplenium alpinum*** Schur

*Chrysosplenium transsilvanicum* Schur = ***Chrysosplenium alpinum*** Schur

*Ciminalis dshimilensis* subsp. *laciniata* (Kit. ex Kanitz) Zuev *≡* ***Gentiana laciniata*** Kit. ex Kanitz

*Colobachne gerardi* Schur, non Link. – ***Alopecurus pratensis*** subsp. ***laguriformis*** (Schur) Tzvelev

*Crociris iridiflora* (Heuff. ex Rchb.) Schur = ***Crocus banaticus*** J.Gay, non Heuff.

*Crociris speciosa* (Host) Schur = ***Crocus banaticus*** J.Gay, non Heuff.

***Crocus banaticus*** J.Gay, non Heuff. *

*Crocus byzantinus* Ker Gawl. [p.p.] – ***Crocus banaticus*** J.Gay, non Heuff.

*Crocus herbertianus* Körn. = ***Crocus banaticus*** J.Gay, non Heuff.

*Crocus iridiflorus* Heuff. ex Rchb. = ***Crocus banaticus*** J.Gay, non Heuff.

*Crocus nudiflorus* Schult., [nom. illeg.], non alior = ***Crocus banaticus*** J.Gay, non Heuff.

*Crocus speciosus* Baumg., non alior = ***Crocus banaticus*** J.Gay, non Heuff.

Crocus speciosus (Baumg.) Host, non alior [nom. illeg.] = ***Crocus banaticus*** J.Gay, non Heuff.

*Crocus speciosus* var. *transsylvanicus* Hooker = ***Crocus banaticus*** J.Gay, non Heuff.

*Crucifera novemfolia* E.H.L.Krause ≡ ***Cardamine glanduligera*** O.Schwarz

*Cyanus maramarosiensis* (Jáv.) Dostál *≡* ***Centaurea maramarosiensis*** (Jáv.) Czerep.

*Cyanus mollis* subsp. *marmarosiensis* (Jáv.) Soó [nom.et.des. invalid] *≡* ***Centaurea maramarosiensis*** (Jáv.) Czerep.

*Cyanus montanus* subsp. *maramarosiensis* (Jáv.) Soják *≡* ***Centaurea maramarosiensis*** (Jáv.) Czerep.

*Delphinium moldavicum* (Hacq.) Bránadza [p.p.major, nom. inval.] *–* ***Aconitum moldavicum*** subsp. ***moldavicum*** Hacq. ex Rchb.

*Delphinium moldavicum* (Hacq.) Bránadza [p.p., nom. inval.] *–* ***Aconitum moldavicum*** subsp. ***hosteanum*** (Schur) Graebn. & P.Graebn.

*Delphinium paniculatum* (Arcang.) E.H.L.Krause [p.p.], non Host – ***Aconitum degenii*** subsp. ***degenii*** Gáyer

*Dentaria enneaphyllos* auct. flora ucrain. carpat., non L. [ex herb. LWS] * – ***Arabidopsis neglecta*** (Schult.) O’Kane & Al-Shehbaz

*Dentaria glandulosa* Waldst. & Kit.* ≡ ***Cardamine glanduligera*** O.Schwarz

*Dianthus acicularis* Schur, non Fisch. ex Ledeb. = ***Dianthus spiculifolius*** Schur

*Dianthus brachyanthus* Schur, non Boiss. = ***Dianthus spiculifolius*** Schur

*Dianthus carpathicus* Borbás, non Woł. [nom. inval.] = ***Dianthus spiculifolius*** Schur

*Dianthus hungaricus* (Andrae) Simonk., non alior = ***Dianthus spiculifolius*** Schur

*Dianthus kitaibelii* Janka subsp. *spiculifolius* (Schur) Novák *≡* ***Dianthus spiculifolius*** Schur

*Dianthus microche[i]lus* B.S.Williams = ***Dianthus spiculifolius*** Schur

*Dianthus microche[i]lus* B.S.Williams ex Wettst. = ***Dianthus spiculifolius*** Schur

*Dianthus petraeus* Janka, non Waldst. & Kit. nec M.Bieb. = ***Dianthus spiculifolius*** Schur

*Dianthus petraeus* Kerner, [nom. nudum], non Waldst. & Kit. nec M.Bieb. = ***Dianthus spiculifolius*** Schur

*Dianthus petraeus* subsp. *spiculifolius* (Schur) Ciocârlan *≡* ***Dianthus spiculifolius*** Schur

*Dianthus plumarius* Baumg.et auct. transsilv., non L. nec Gunnerus = ***Dianthus spiculifolius*** Schur

*Dianthus plumarius* subsp. *spiculifolius* (Schur) Baksay *≡* ***Dianthus spiculifolius*** Schur

*Dianthus plumarius* var. *erythrocalyx* Schott ex Simonk. = ***Dianthus spiculifolius*** Schur

*Dianthus plumarius* var. *hungaricus* Andrae, non alior = ***Dianthus spiculifolius*** Schur

*Dianthus serotinus* Barth, [nom. nudum], non Waldst. & Kit. = ***Dianthus spiculifolius*** Schur

*Dianthus serotinus* Salzer, [nom. nudum], non Waldst. & Kit. = ***Dianthus spiculifolius*** Schur

*Dianthus spiculifolius* f. *petraeiformis* Novák = ***Dianthus spiculifolius*** Schur

***Dianthus spiculifolius*** Schur

***Doronicum carpaticum*** (Griseb. & Schenk) Nyman *

*Doronicum carpaticum* var. *barcense* (Simonk.) Borbás = ***Doronicum carpaticum*** (Griseb. & Schenk) Nyman

*Doronicum columnae* subsp. *carpaticum* (Griseb. & Schenk) Sóo *≡* ***Doronicum carpaticum*** (Griseb. & Schenk) Nyman

*Doronicum cordatum* var. *asperum* Borbás = ***Doronicum carpaticum*** (Griseb. & Schenk) Nyman

*Doronicum grandiflorum* subsp. *carpaticum* (Griseb. & A.Schenk) Rouy *≡* ***Doronicum carpaticum*** (Griseb. & Schenk) Nyman

*Doronicum orientale* Kotschy [nom. nudum], non alior = ***Doronicum carpaticum*** (Griseb. & Schenk) Nyman

*Doronicum pardalianches* Heuff. , non alior = ***Doronicum carpaticum*** (Griseb. & Schenk) Nyman

*Elisanthe zawadskii* (Herbich) Klokov * *≡* ***Silene zawadzkii*** Herbich

*Elisanthe zawadzkii* (Herbich) Fuss *≡* ***Silene zawadzkii*** Herbich

*Erysimum baumgartenianum* Jáv., non Schur = ***Erysimum witmannii*** subsp. ***transsilvanicum*** (Schur) P.W.Ball

Erysimum cheiranthus Herbich, non alior – ***Erysimum witmannii*** subsp. ***transsilvanicum*** (Schur) P.W.Ball

*Erysimum czetzianum* Schur ex Jáv. = ***Erysimum witmannii*** subsp. ***transsilvanicum*** (Schur) P.W.Ball

*Erysimum czetzianum* Schur = ***Erysimum witmannii*** subsp. ***transsilvanicum*** (Schur) P.W.Ball

*Erysimum neglectum* (Schult.) Kuntze *≡* ***Arabidopsis neglecta*** (Schult.) O’Kane & Al-Shehbaz

*Erysimum odoratum* Baumg. [p.p.], non Ehr. – ***Erysimum witmannii*** subsp. ***transsilvanicum*** (Schur) P.W.Ball

Erysimum pannonicum auct. carpat., non Crantz – ***Erysimum witmannii*** subsp. ***transsilvanicum*** (Schur) P.W.Ball

Erysimum pannonicum f. viridis Simonk. = ***Erysimum witmannii*** subsp. ***transsilvanicum*** (Schur) P.W.Ball

*Erysimum pumilum* var*. transilvanica* Schur = ***Erysimum witmannii*** subsp. ***transsilvanicum*** (Schur) P.W.Ball

*Erysimum transsilvanicum* f. *czetzianum* (Schur) Nyár. = ***Erysimum witmannii*** subsp. ***transsilvanicum*** (Schur) P.W.Ball

*Erysimum transsilvanicum* f. *luxurians* Nyár. = ***Erysimum witmannii*** subsp. ***transsilvanicum*** (Schur) P.W.Ball

*Erysimum transsilvanicum* f. *rarifolium* Nyár. = ***Erysimum witmannii*** subsp. ***transsilvanicum*** (Schur) P.W.Ball

*Erysimum transsilvanicum* Schur * *≡* ***Erysimum witmannii*** subsp. ***transsilvanicum*** (Schur) P.W.Ball

*Erysimum wahlenbergii* Simonk., non Asch. & Engl. – ***Erysimum witmannii*** subsp. ***transsilvanicum*** (Schur) P.W.Ball

*Erysimum witmannii* auct. flora ucrain. carpat.* – ***Erysimum witmannii*** subsp. ***transsilvanicum*** (Schur) P.W.Ball

*Erysimum witmannii* Grec. [p.p.], non Zaw. – ***Erysimum witmannii*** subsp. ***transsilvanicum*** (Schur) P.W.Ball

*Erysimum witmannii* subsp. *czetzianum* (Schur) Zapał. [ex herb. Mądalski, nom. inval. ?] = ***Erysimum witmannii*** subsp. ***transsilvanicum*** (Schur) P.W.Ball

***Erysimum witmannii*** subsp. ***transsilvanicum*** (Schur) P.W.Ball

*Erysimum witmannii* var. *czetziano* Nyár. = ***Erysimum witmannii*** subsp. ***transsilvanicum*** (Schur) P.W.Ball

*Erysimum witmannii* var. *czetzianum* (Schur) Borza = ***Erysimum witmannii*** subsp. ***transsilvanicum*** (Schur) P.W.Ball

*Euphrasia minima* subsp. *tatrae* (Wettst.) Hayek in Hegi * *≡* ***Euphrasia tatrae*** Wettst.

*Euphrasia minima* var. *carpathica* Freyn in Sagorski & Schneider, non *Euphrasia carpatica* Zapał. = ***Euphrasia tatrae*** Wettst.

*Euphrasia minima* var. *tatrae* (Wettst.) Pawł. ≡ ***Euphrasia tatrae*** Wettst.

*Euphrasia minima* var. *tatrae* f. *glandulifera* (Wettst.) Răvăruţ = ***Euphrasia tatrae*** Wettst.

*Euphrasia officinalis* [unranked] δ *alpestris* Freyn, non Günther, Grab. & Wimm. = ***Euphrasia tatrae*** Wettst.

*Euphrasia tatrae* f. *glandulifera* Wettst. = ***Euphrasia tatrae*** Wettst.

*Euphrasia tatrae* subsp. *glandulifera* (Wettst.) Staszk. = ***Euphrasia tatrae*** Wettst.

***Euphrasia tatrae*** Wettst.*

*Festuca amethystina* [p.p., tantum quod plantas ucrain. carpat.] * – ***Festuca amethystina*** subsp. ***orientalis*** Krajina, non alior

*Festuca amethystina* [unranked] a *marmarossica* Zapał. [ortho. var.] * = ***Festuca amethystina*** subsp. ***orientalis*** Krajina, non alior

*Festuca amethystina* [unranked] a *marmarossiensis* f. *doamnensis* Zapał. = ***Festuca amethystina*** subsp. ***orientalis*** Krajina, non alior

*Festuca amethystina* [unranked] a *marmarossiensis* Zapał. = ***Festuca amethystina*** subsp. ***orientalis*** Krajina, non alior

*Festuca amethystina* f. *pauciflora* A.Nyár. & Nyár. = ***Festuca amethystina*** subsp. ***orientalis*** Krajina, non alior

*Festuca amethystina* subsp. *amethystina* var. *amethystina* f. *marmarossica* (Zapał.) Beldie = ***Festuca amethystina*** subsp. ***orientalis*** Krajina, non alior

*Festuca amethystina* subsp. *amethystina* var. *amethystina* f. *pauciflora* (A.Nyár. & Nyár.) Beldie = ***Festuca amethystina*** subsp. ***orientalis*** Krajina, non alior

*Festuca amethystina* subsp. in*armata* (Schur) Krajina = ***Festuca amethystina*** subsp. ***orientalis*** Krajina, non alior

***Festuca amethystina*** subsp. ***orientalis*** Krajina, non alior *

***Festuca carpatica*** F.Dietr.*

*Festuca carpatica* f. *elatior* Krajina = ***Festuca carpatica*** F.Dietr.

*Festuca carpatica* f. *pseudolaxa* (Schur) Jáv. = ***Festuca carpatica*** F.Dietr.

*Festuca carpatica* f. *subflavescens* Zapał. = ***Festuca carpatica*** F.Dietr.

*Festuca carpatica* var. *bucegica* (Krajina) Beldie = ***Festuca carpatica*** F.Dietr.

*Festuca carpatica* var. *carpatica* f. *subflavescens* (Zapał.) Beldie = ***Festuca carpatica*** F.Dietr.

*Festuca carpatica* var. *carpatica* f. *umbrosa* Beldie = ***Festuca carpatica*** F.Dietr.

*Festuca dimorpha* Janka, non Guss. = ***Festuca carpatica*** F.Dietr.

*Festuca heterophylla* var. in*armata* Schur ex Schur = ***Festuca amethystina*** subsp. ***orientalis*** Krajina, non alior

*Festuca heterophylla* var. *setifolia* Schur ex Schur = ***Festuca amethystina*** subsp. ***orientalis*** Krajina, non alior

*Festuca inarmata* Schur * = ***Festuca amethystina*** subsp. ***orientalis*** Krajina, non alior

*Festuca laxa* Schur, non Host = ***Festuca carpatica*** F.Dietr.

Festuca minor Schur, non St.-Lag. = **Festuca versicolor** subsp. **versicolor** Tausch

*Festuca nutans* Wahlenb., non Host nec Moench = ***Festuca carpatica*** F.Dietr.

*Festuca porcii* f. *hirsuta* (A.Nyár.) Beldie = ***Festuca porcii*** Hack.

*Festuca porcii* f. *longiaristata* Krajina = ***Festuca porcii*** Hack.

*Festuca porcii* f. *vestita* (Hack.) Krajina = ***Festuca porcii*** Hack.

***Festuca porcii*** Hack.*

*Festuca porcii* var. *hirsuta* A.Nyár. = ***Festuca porcii*** Hack.

*Festuca porcii* var. *vestita* Hack. ex Zapał. = ***Festuca porcii*** Hack.

*Festuca pseudolaxa* Schur = ***Festuca carpatica*** F.Dietr.

*Festuca pseudonutans* Schur = ***Festuca carpatica*** F.Dietr.

*Festuca pulchella* subsp. *scheuchzeriformis* var. *bucegica* Krajina = ***Festuca carpatica*** F.Dietr.

*Festuca scheuchzeriformis* Schur = ***Festuca carpatica*** F.Dietr.

*Festuca varia* [unranked] *flavescens* Zapał., non Gaudin = ***Festuca versicolor*** subsp. ***versicolor*** Tausch, non J.Presl ex Kunth

*Festuca varia* [unranked] *giewontica* Zapał. = ***Festuca versicolor*** subsp. ***versicolor*** Tausch, non J.Presl ex Kunth

*Festuca varia* f. *acuminata* Sagorski & Schneider, non (Gaudin) Bolzon = ***Festuca versicolor*** subsp. ***versicolor*** Tausch, non J.Presl ex Kunth

*Festuca varia* f. *pallidula* auct., non Hack. – ***Festuca versicolor*** subsp. ***versicolor*** Tausch, non J.Presl ex Kunth

*Festuca varia* subsp. *pumila* [unranked] *spiculis* *flavescentibus* Gaudin ex Hack. in Sagorski & Schneider = ***Festuca versicolor*** subsp. ***versicolor*** Tausch, non J.Presl ex Kunth

*Festuca varia* var. *scopariaeformis* Kotula = ***Festuca versicolor*** subsp. ***versicolor*** Tausch, non J.Presl ex Kunth

*Festuca versicolor* subsp. *eu-versicolor* var. *genuina* subvar. *rodnensis* Krajina = ***Festuca versicolor*** subsp. ***versicolor*** Tausch, non J.Presl ex Kunth

*Festuca versicolor* subsp. *eu-versicolor* var. *genuina* subvar. *rodnensis* f. *minor* Krajina = ***Festuca versicolor*** subsp. ***versicolor*** Tausch, non J.Presl ex Kunth

*Festuca versicolor* subsp. *eu-versicolor* var. *genuina* subvar. *rodnensis* f. *typica* Krajina = ***Festuca versicolor*** subsp. ***versicolor*** Tausch, non J.Presl ex Kunth

*Festuca versicolor* subsp. *eu-versicolor* var. *genuina* subvar. *transsilvanica* Krajina = ***Festuca versicolor*** subsp. ***versicolor*** Tausch, non J.Presl ex Kunth

*Festuca versicolor* subsp. *eu-versicolor* var. *genuina* subvar. *transsilvanica* f. *Kotschyi* Krajina = ***Festuca versicolor*** subsp. ***versicolor*** Tausch, non J.Presl ex Kunth

*Festuca versicolor* subsp. *eu-versicolor* var. *genuina* subvar. *transsilvanica* f. *pallens* Krajina = ***Festuca versicolor*** subsp. ***versicolor*** Tausch, non J.Presl ex Kunth

*Festuca versicolor* subsp. *eu-versicolor* var. *genuina* subvar. *transsilvanica* f. *typica* Krajina = ***Festuca versicolor*** subsp. ***versicolor*** Tausch, non J.Presl ex Kunth

*Festuca versicolor* subsp. *eu-versicolor* var. *genuina* subvar. *vulgaris* Krajina = ***Festuca versicolor*** subsp. ***versicolor*** Tausch, non J.Presl ex Kunth

*Festuca versicolor* subsp. *eu-versicolor* var. *genuina* subvar. *vulgaris* f. *chrysantha* Krajina = ***Festuca versicolor*** subsp. ***versicolor*** Tausch, non J.Presl ex Kunth

*Festuca versicolor* subsp. *eu-versicolor* var. *genuina* subvar. *vulgaris* f. *curvala* Krajina = ***Festuca versicolor*** subsp. ***versicolor*** Tausch, non J.Presl ex Kunth

*Festuca versicolor* subsp. *eu-versicolor* var. *genuina* subvar. *vulgaris* f. *debilis* Krajina = ***Festuca versicolor*** subsp. ***versicolor*** Tausch, non J.Presl ex Kunth

*Festuca versicolor* subsp. *eu-versicolor* var. *genuina* subvar. *vulgaris* f. *giewontica* (Zapał.) Krajina = ***Festuca versicolor*** subsp. ***versicolor*** Tausch, non J.Presl ex Kunth

*Festuca versicolor* subsp. *eu-versicolor* var. *genuina* subvar. *vulgaris* f. *glaucophylla* Krajina = ***Festuca versicolor*** subsp. ***versicolor*** Tausch, non J.Presl ex Kunth

*Festuca versicolor* subsp. *eu-versicolor* var. *genuina* subvar. *vulgaris* f. *mutica* Krajina = ***Festuca versicolor*** subsp. ***versicolor*** Tausch, non J.Presl ex Kunth

*Festuca versicolor* subsp. *eu-versicolor* var. *genuina* subvar. *vulgaris* f. *robustior* Krajina = ***Festuca versicolor*** subsp. ***versicolor*** Tausch, non J.Presl ex Kunth

*Festuca versicolor* subsp. *eu-versicolor* var. *genuina* subvar. *vulgaris* f. *scopariaeformis* (Kotula) Krajina = ***Festuca versicolor*** subsp. ***versicolor*** Tausch, non J.Presl ex Kunth

*Festuca versicolor* subsp. *eu-versicolor* var. *genuina* subvar. *vulgaris* f. *typica* Krajina = ***Festuca versicolor*** subsp. ***versicolor*** Tausch, non J.Presl ex Kunth

*Festuca versicolor* subsp. *eu-versicolor* var. *genuina* subvar. *vulgaris* f. *zapalowiczii* Krajina = ***Festuca versicolor*** subsp. ***versicolor*** Tausch, non J.Presl ex Kunth

*Festuca versicolor* subsp. *pseudosulcata* Krajina, non Drobow = ***Festuca versicolor*** subsp. ***versicolor*** Tausch, non J.Presl ex Kunth

*Festuca versicolor* subsp. *versicolor* Tausch ex Kraj., non J.Presl ex Kunth *≡* ***Festuca versicolor*** subsp. ***versicolor*** Tausch, non J.Presl ex Kunth

***Festuca versicolor*** subsp. ***versicolor*** Tausch, non J.Presl ex Kunth *

*Festuca versicolor* var. *minor* (Schur) Krajina = ***Festuca versicolor*** subsp. ***versicolor*** Tausch, non J.Presl ex Kunth

*Festuca versicolor* var. *versicolor* f. *chrysantha* (Krajina) Beldie = ***Festuca versicolor*** subsp. ***versicolor*** Tausch, non J.Presl ex Kunth

*Festuca versicolor* var. *versicolor* f. *debilis* (Krajina) Beldie = ***Festuca versicolor*** subsp. ***versicolor*** Tausch, non J.Presl ex Kunth

***Galium album*** subsp. ***suberectum*** (Klokov) Michálk.

*Galium erectum* auct. fl. ucrain. carpat., non Huds. – ***Galium album*** subsp. ***suberectum*** (Klokov) Michálk.

*Galium erectum* subsp. *suberectum* (Klokov) Kobiv et al. [nom. illeg.] *≡* ***Galium album*** subsp. ***suberectum*** (Klokov) Michálk.

*Galium mollugo* subsp. *erectum* (Huds.) Syme sensu Kucowa in Pawł. [p.p.] – ***Galium album*** subsp. ***suberectum*** (Klokov) Michálk.

*Galium mollugo* subsp. *erectum* f. *longifolium* Kucowa in Pawł. = ***Galium album*** subsp. ***suberectum*** (Klokov) Michálk.

*Galium suberectum* Klokov * *≡* ***Galium album*** subsp. ***suberectum*** (Klokov) Michálk.

***Galium transcarpaticum*** Stojko & Tasenk.*

*Genista alpicola* Schur = ***Genista tinctoria*** subsp. ***oligosperma*** (Andrae) Soó

*Genista oligosperma* (Andrae) Simonk.* *≡* ***Genista tinctoria*** subsp. ***oligosperma*** (Andrae) Soó

*Genista oligosperma* f. *alpicola* (Schur) Morariu = ***Genista tinctoria*** subsp. ***oligosperma*** (Andrae) Soó

*Genista oligosperma* f. *ghisae* Pawłowski = ***Genista tinctoria*** subsp. ***oligosperma*** (Andrae) Soó

*Genista procumbens* Baumg. ex Fuss [nom. inval.], non alior – ***Genista tinctoria*** subsp. ***oligosperma*** (Andrae) Soó

*Genista rupestris* Schur * = ***Genista tinctoria*** subsp. ***oligosperma*** (Andrae) Soó

*Genista sigeriana* Fuss = ***Genista tinctoria*** subsp. ***oligosperma*** (Andrae) Soó

*Genista tinctoria* subsp. *oligosperma* (Andrae) Malinovsky [comb. invalid.] *≡* ***Genista tinctoria*** subsp. ***oligosperma*** (Andrae) Soó

***Genista tinctoria*** subsp. ***oligosperma*** (Andrae) Soó

*Genista tinctoria* var. *oligosperma* Andrae *≡* ***Genista tinctoria*** subsp. ***oligosperma*** (Andrae) Soó

*Genista tinctoria* var. *prostrata* auct., non Bab. = ***Genista tinctoria*** subsp. ***oligosperma*** (Andrae) Soó

***Gentiana laciniata*** Kit. ex Kanitz *

*Gentiana pyrenaica* auct. fl. ucrain. carpat., non L.* – ***Gentiana laciniata*** Kit. ex Kanitz

*Gentiana pyrenaica* var. *laciniata* (Kit. ex Kanitz) Jáv.* *≡* ***Gentiana laciniata*** Kit. ex Kanitz

*Gentiana vagneriana* Janka = ***Gentiana laciniata*** Kit. ex Kanitz

Gentiana wagneri Janka [nom. inval.; ortho. var.] = ***Gentiana laciniata*** Kit. ex Kanitz

*Gnaphalium alpinum* Willd., non L. [p.p., tantum quod plantas ucrain. carpat.] – ***Antennaria carpatica*** subsp. ***carpatica*** (Wahlenb.) Bluff & Fingerh.

*Gnaphalium carpaticum* Wahlenb. ≡ ***Antennaria carpatica*** subsp. ***carpatica*** (Wahlenb.) Bluff & Fingerh.

*Gnaphalium wahlenbergii* Sieber ex Steud. = ***Antennaria carpatica*** subsp. ***carpatica*** (Wahlenb.) Bluff & Fingerh.

***Gymnadenia carpatica*** (Zapał.) Teppner & E.Klein

*Gymnadenia nigra* auct. fl. ucrain. carpat., non (L.) Rchb.f. – ***Gymnadenia carpatica*** (Zapał.) Teppner & E.Klein

*Heracleum alpinum* Baumg. = ***Heracleum carpaticum*** Porcius

*Heracleum alpinum* subsp. *palmatum* (Baumg.) Briquet, non Crantz nec Rchb. = ***Heracleum sphondylium*** subsp. ***transsilvanicum*** (Schur) Brummitt

*Heracleum carpaticum* f. *alpinum* (Baumg.) Borza = ***Heracleum carpaticum*** Porcius

*Heracleum carpaticum* f. *palmatifidum* Jáv. = ***Heracleum carpaticum*** Porcius

*Heracleum carpaticum* f. *porcii* Pax = ***Heracleum carpaticum*** Porcius

*Heracleum carpaticum* f. *typicum* Nyár & Todor = ***Heracleum carpaticum*** Porcius

***Heracleum carpaticum*** Porcius *

*Heracleum carpaticum* var. *aconitifolium* M.Pop. & Chrshan. [ex herb., nom. inval.], non Woronow – ***Heracleum carpaticum*** Porcius

*Heracleum palmatum* Baumg.* = ***Heracleum sphondylium*** subsp. ***transsilvanicum*** (Schur) Brummitt

*Heracleum palmatum* subsp. *transsilvanicum* (Schur) Nyman *≡* ***Heracleum sphondylium*** subsp. ***transsilvanicum*** (Schur) Brummitt

*Heracleum pollinianum* Nyman [p.p., tantum quod plantas ucrain. carpat.], non Bertol. – ***Heracleum carpaticum*** Porcius

*Heracleum simplicifolium* Herb. [p.p., tantum quod plantas ucrain. carpat.] – ***Heracleum carpaticum*** Porcius

*Heracleum simplicifolium* Herb. [p.p., tantum quod plantas ucrain. carpat.] – ***Heracleum sphondylium*** subsp. ***transsilvanicum*** (Schur) Brummitt

*Heracleum simplicifolium* Herb. ex Nyman [p.p., tantum quod plantas ucrain. carpat.] – ***Heracleum carpaticum*** Porcius

*Heracleum simplicifolium* Herb. ex Nyman sensu Borza [p.p.] – ***Heracleum carpaticum*** Porcius

*Heracleum sphondylium* subsp. *carpaticum* (Porcius) Soó *≡* ***Heracleum carpaticum*** Porcius

***Heracleum sphondylium*** subsp. ***transsilvanicum*** (Schur) Brummitt

*Heracleum sphondylium* subsp. *transsilvanicum* (Schur) Thellung [nom. invalid.] *≡* ***Heracleum sphondylium*** subsp. ***transsilvanicum*** (Schur) Brummitt

*Heracleum transsilvanicum* Schur *≡* ***Heracleum sphondylium*** subsp. ***transsilvanicum*** (Schur) Brummitt

*Jacea carpatica* (Porcius) Soják *≡* ***Centaurea phrygia*** subsp. ***carpatica*** (Porcius) Dostál

*Jacea phrygia* (L.) Soják subsp. *carpatica* (Porcius) Dostál *≡* ***Centaurea phrygia*** subsp. ***carpatica*** (Porcius) Dostál

*Jovibarba globifera* subsp. *hirta* (L.) J.Parn. [p.p., tantum quod plantas ucrain. carpat.] – ***Sempervivum globiferum*** subsp. ***preissianum*** (Domin) M.Werner

*Jovibarba globifera* subsp. *preissiana* (Domin) Holub *≡* ***Sempervivum globiferum*** subsp. ***preissianum*** (Domin) M.Werner

*Jovibarba globifera* var. *preissiana* (Domin) Hadrava & Miklánek *≡* ***Sempervivum globiferum*** subsp. ***preissianum*** (Domin) M.Werner

*Jovibarba hirta* subsp. *preissiana* (Domin) Soó *≡* ***Sempervivum globiferum*** subsp. ***preissianum*** (Domin) M.Werner

*Jovibarba hirta* subsp. *tatrensis* (Domin) Á.Löve & D.Löve = ***Sempervivum globiferum*** subsp. ***preissianum*** (Domin) M.Werner

*Jovibarba globifera* var. *tatrensis* (Domin) Konop & Bendak [nom. illeg.] = ***Sempervivum globiferum*** subsp. ***preissianum*** (Domin) M.Werner

*Jovibarba hirta* var. *tatrense* (Domin) Soó = ***Sempervivum globiferum*** subsp. ***preissianum*** (Domin) M.Werner

Jovibarba hirta var. tatrensis (Dom.) Konop & Bendak [nom. illeg.] = ***Sempervivum globiferum*** subsp. ***preissianum*** (Domin) M.Werner

*Jovibarba preissiana* (Domin) Omelczuk & Chopik * *≡* ***Sempervivum globiferum*** subsp. ***preissianum*** (Domin) M.Werner

*Jovibarba sobolifera* (Sims) Opiz [p.p., tantum quod plantas ucrain. carpat.] * – ***Sempervivum globiferum*** subsp. ***preissianum*** (Domin) M.Werner

*Juncus alpinopilosus* Chaix [p.p., tantum quod plantas carpat.] – ***Luzula alpinopilosa*** subsp. ***obscura*** S.E.Fröhner

*Juncus spadiceus* [unranked] β *glabratus* Wahlbg., non Hoppe nec Host = ***Luzula alpinopilosa*** subsp. ***obscura*** S.E.Fröhner

*Juncus spadiceus* All. [nom. invalid., p.p., tantum quod plantas carpat.], non alior – ***Luzula alpinopilosa*** subsp. ***obscura*** S.E.Fröhner

Koeleria colorata (Heuff.) Nyár. ex Degen [p.p.] – ***Koeleria transsilvanica*** Schur, non Barth.

*Koeleria cristata* [unranked] d) *transsilvanica* (Schur) K.Richt. ≡ ***Koeleria transsilvanica*** Schur, non Barth.*

*Koeleria cristata* [unranked] *foliis vaginisque glabris* Andrä [ex herb., nom. nudum] – ***Koeleria transsilvanica*** Schur, non Barth.

*Koeleria cristata* subsp. *ciliata* var. *transsilvanica* (Schur) Asch. & Graebn. ≡ ***Koeleria transsilvanica*** Schur, non Barth.

Koeleria cristata var. colorata Heuff. [p.p.] – ***Koeleria transsilvanica*** Schur, non Barth.

*Koeleria cristata* var. *glabra* Kotschy [ex herb., nom. nudum], non alior. – ***Koeleria transsilvanica*** Schur, non Barth.

*Koeleria gracilis* f. *colorata* (Heuff.) Domin [p.p.] – ***Koeleria transsilvanica*** Schur, non Barth.

*Koeleria gracilis* Pers [p.p., ex herb.], non Guss. – ***Koeleria transsilvanica*** Schur, non Barth.

*Koeleria gracilis* subsp. *transsilvanica* (Schur) Domin *≡* ***Koeleria transsilvanica*** Schur, non Barth.

*Koeleria gracilis* var. *colorata* (Heuff.) Domin, non alior [p.p.] – ***Koeleria transsilvanica*** Schur, non Barth.

*Koeleria gracilis* var. *rohlenae* Domin = ***Koeleria transsilvanica*** Schur, non Barth.

*Koeleria gracilis* var. *transsilvanica* (Schur) Jáv. ≡ ***Koeleria transsilvanica*** Schur, non Barth.

*Koeleria gracilis* var. *typica* Domin = ***Koeleria transsilvanica*** Schur, non Barth.

*Koeleria macrantha* var. *colorata* (Heuff.) Ghisa [p.p.] – ***Koeleria transsilvanica*** Schur, non Barth.

*Koeleria macrantha* subsp. *transsilvanica* (Schur) A.Nyár. [nom. nudum ?] *≡* ***Koeleria transsilvanica*** Schur, non Barth.

*Koeleria macrantha* subsp. *transsilvanica* (Schur) Beldie [nom. illeg.] *≡* ***Koeleria transsilvanica*** Schur, non Barth.

*Koeleria setacea* DC. sensu Nyman – ***Koeleria transsilvanica*** Schur, non Barth.

*Koeleria tenuipes* (Schur) Ujhelyi = ***Koeleria transsilvanica*** Schur, non Barth.

***Koeleria transsilvanica*** Schur, non Barth.

*Koeleria transsilvanica* [unranked] a *tenuipes alpestris* Schur = ***Koeleria transsilvanica*** Schur, non Barth.

*Koeleria transsilvanica* [unranked] b *tenuipes alpestris* Schur = ***Koeleria transsilvanica*** Schur, non Barth.

*Koeleria transsilvanica* subsp. *tenuipes* (Schur) Soó = ***Koeleria transsilvanica*** Schur, non Barth.

*Koeleria transsilvanica* var. *tenuipes* (Schur) Domin = ***Koeleria transsilvanica*** Schur, non Barth.

*Koeleria transsilvanica* var. *tenuipes* f. *discolor* Degen ex Domin = ***Koeleria transsilvanica*** Schur, non Barth.

*Lathyrus laevigatus* subsp. *transsylvanicus* (Spreng.) Breistr. ≡ ***Lathyrus transsilvanicus*** (Spreng.) Rchb.f.

*Lathyrus linnaei* f. *transsilvanicus* (Spreng.) Rouy in Rouy & Foucad *≡* ***Lathyrus transsilvanicus*** (Spreng.) Rchb.f.

*Lathyrus luteus* [unranked] a *transsilvanicus* (Spreng.) Ascherson & Graebn. ≡ ***Lathyrus transsilvanicus*** (Spreng.) Rchb.f.

*Lathyrus luteus* [unranked] c *transsylvanicus* (Spreng.) Beck in Rchb. ≡ ***Lathyrus transsilvanicus*** (Spreng.) Rchb.f.

*Lathyrus luteus* subsp. *transsylvanicus* (Spreng.) Dostal *≡* ***Lathyrus transsilvanicus*** (Spreng.) Rchb.f.

*Lathyrus transsilvanicus* (Spreng.) R.M.Fritsch [nom. inval.] *≡* ***Lathyrus transsilvanicus*** (Spreng.) Rchb.f.

***Lathyrus transsilvanicus*** (Spreng.) Rchb.f. *

*Lathyrus transsilvanicus* f. *trichocarpus* Borbás in Nyár. = ***Lathyrus transsilvanicus*** (Spreng.) Rchb.f.

*Leontodon clavatus* Sagorski & Schneider * *=* ***Scorzoneroides pseudotaraxaci*** (Schur) Holub

*Leontodon medius* Simonk., non *Apargia media* Host *=* ***Scorzoneroides pseudotaraxaci*** (Schur) Holub

*Leontodon montanus* subsp. *pseudotaraxaci* (Schur) Finch & P.D.Sell *≡* ***Scorzoneroides pseudotaraxaci*** (Schur) Holub

*Leontodon pseudotaraxaci* Schur * *≡* ***Scorzoneroides pseudotaraxaci*** (Schur) Holub

Leontodon pyrenaeus R.Uechtr. [nom. illeg], non Gouan – ***Scorzoneroides pseudotaraxaci*** (Schur) Holub

Leontodon pyrenaicus Hoborski [nom. illeg], non Gouan – ***Scorzoneroides pseudotaraxaci*** (Schur) Holub

*Leontodon taraxaci* auct., non (L.) Loisel. – ***Scorzoneroides pseudotaraxaci*** (Schur) Holub

*Leontodon taraxaci* R.Uechtr. [nom. illeg], non Loisel. – ***Scorzoneroides pseudotaraxaci*** (Schur) Holub

*Leontodon taraxaci* var. *tatricus* Kotula *=* ***Scorzoneroides pseudotaraxaci*** (Schur) Holub

*Leontodon tatricis* (Kotula) Woł. [ortho. var.] = ***Scorzoneroides pseudotaraxaci*** (Schur) Holub

*Leontodon tatricus* (Kotula) Woł. = ***Scorzoneroides pseudotaraxaci*** (Schur) Holub

*Leucanthemum rotundifolium* (Waldst. & Kit. in Willd.) Baumg. ≡ ***Leucanthemum rotundifolium*** (Waldst. & Kit. in Willd.) DC., non Opiz

***Leucanthemum rotundifolium*** (Waldst. & Kit. in Willd.) DC., non Opiz *

*Leucanthemum rotundifolium* (Waldst. & Kit. in Willd.) Schur *≡* ***Leucanthemum rotundifolium*** (Waldst. & Kit. in Willd.) DC., non Opiz

*Leucanthemum waldsteinii* (Sch.Bip.) Pouzar = ***Leucanthemum rotundifolium*** (Waldst. & Kit. in Willd.) DC., non Opiz

*Leucopoa carpatica* (F.Dietr.) H.Scholz *≡* ***Festuca carpatica*** F.Dietr.

*Linum alpinum* auct. fl. transsilv., non L. – ***Linum extraaxillare*** Kit. ex Rochel

*Linum extraaxillare* Kit. ≡ ***Linum extraaxillare*** Kit. ex Rochel

***Linum extraaxillare*** Kit. ex Rochel *

*Linum montanum* auct. fl. transsilv., non Schleich. – ***Linum extraaxillare*** Kit. ex Rochel

*Linum perenne* subsp. ex*traaxillare* (Kit. ex Rochel) Nyman *≡* ***Linum extraaxillare*** Kit. ex Rochel

*Luzula alpinopilosa* (Chaix) Breistr. [p.p., tantum quod plantas carpat.] * – ***Luzula alpinopilosa*** subsp. ***obscura*** S.E.Fröhner

***Luzula alpinopilosa*** subsp. ***obscura*** S.E.Fröhner

*Luzula carpatica* Kitt. ex Kanitz = ***Luzula alpinopilosa*** subsp. ***obscura*** S.E.Fröhner

*Luzula obscura* (S.E.Fröhner) Novikov *≡* ***Luzula alpinopilosa*** subsp. ***obscura*** S.E.Fröhner

*Luzula spadicea* (All.) Lam. & DC. [p.p., tantum quod plantas carpat.] * – ***Luzula alpinopilosa*** subsp. ***obscura*** S.E.Fröhner

*Luzula spadicea* [unranked] *carpatica* (Kitt. ex Kanitz) Asch. & Graebn. = ***Luzula alpinopilosa*** subsp. ***obscura*** S.E.Fröhner

*Luzula spadicea* f. *carpatica* (Kitt. ex Kanitz) I.Grinț. = ***Luzula alpinopilosa*** subsp. ***obscura*** S.E.Fröhner

*Luzula spadicea* var. *carpatica* (Kitt. ex Kanitz) Nyman = ***Luzula alpinopilosa*** subsp. ***obscura*** S.E.Fröhner

*Matricaria rotundifolia* (Waldst. & Kit. in Willd.) Poir. ≡ ***Leucanthemum rotundifolium*** (Waldst. & Kit. in Willd.) DC., non Opiz

*Melampyrum herbichii* subsp. *csatoi* (Soó) Soó = ***Melampyrum saxosum*** Baumg.

*Melampyrum* *herbichii* subsp. *woloszczakii* Jasiewicz = ***Melampyrum saxosum*** Baumg.

*Melampyrum herbichii* Woł. = ***Melampyrum saxosum*** Baumg.

*Melampyrum pictum* Herbich [nom inval., ex herb LWS] * – ***Melampyrum saxosum*** Baumg.

*Melampyrum saxosum* [unranked] *baumgartenii* Soó ex Jáv. = ***Melampyrum saxosum*** Baumg.

*Melampyrum saxosum* [unranked] *javorkae* Soó ex Jáv. = ***Melampyrum saxosum*** Baumg.

***Melampyrum saxosum*** Baumg.*

*Melampyrum* saxosum subsp. *baumgartenii* (Soó) Soó = ***Melampyrum saxosum*** Baumg.

*Melampyrum saxosum* subsp. *javorkae* (Soó) Soó = ***Melampyrum saxosum*** Baumg.

*Melampyrum saxosum* var. *baumgartenii* (Soó) Nyár. = ***Melampyrum saxosum*** Baumg.

*Melampyrum saxosum* var. *javorkae* (Soó) Nyár. = ***Melampyrum saxosum*** Baumg.

*Melampyrum saxosum* var. *typicum* Nyár. = ***Melampyrum saxosum*** Baumg.

*Melampyrum sylvaticum* [unranked] *M.saxosum* (Baumg.) Nyman *≡* ***Melampyrum saxosum*** Baumg.

*Melampyrum sylvaticum* [unranked] α *pictum* Herbich = ***Melampyrum saxosum*** Baumg.

*Melampyrum sylvaticum* f. *csatoi* Soó = ***Melampyrum saxosum*** Baumg.

*Melampyrum sylvaticum* Simonk. [p.p.], non L. – ***Melampyrum saxosum*** Baumg.

*Melampyrum sylvaticum* subsp. *moeszianum* Soó = ***Melampyrum saxosum*** Baumg.

*Melampyrum sylvaticum* subsp. *saxosum* (Baumg.) G.Beauvis.* *≡* ***Melampyrum saxosum*** Baumg.

*Melampyrum sylvaticum* subsp. *saxosum* var. *herbichii* (Woł.) G.Beauvis. = ***Melampyrum saxosum*** Baumg.

*Melampyrum sylvaticum* subsp. *saxosum* var. *pictum* subvar. *eu-pictum* G.Beauvis. = ***Melampyrum saxosum*** Baumg.

*Melampyrum sylvaticum* subsp. *saxosum* var. *pictum* subvar. *eu-saxosum* G.Beauvis. = ***Melampyrum saxosum*** Baumg.

*Melampyrum sylvaticum* subsp. *saxosum* var. β *pictum* (Herbich) G.Beauvis. = ***Melampyrum saxosum*** Baumg.

*Melampyrum sylvaticum* var. β *saxosum* Willkomm = ***Melampyrum saxosum*** Baumg.

*Melandrium zawadskii* (Herbich) A.Braun [nom. nudum] * *≡* ***Silene zawadzkii*** Herbich

*Minuartia gerardii* auct. fl. carpat., non (Willd.) Hayek * – ***Sabulina pauciflora*** (Kit.) A.Novikov, *comb. nov.*

*Minuartia oxypetala* (Woł.) Kulczyński * *≡* ***Sabulina oxypetala*** (Woł.) Mosyakin & Fedor.

*Minuartia pauciflora* (Kit.) Dvořaková * *≡* ***Sabulina pauciflora*** (Kit.) A.Novikov, *comb. nov.*

*Minuartia verna* [unranked] B *attica* [unranked] *oxypetala* Graebn. in Asch. & Graebn. ≡ ***Sabulina oxypetala*** (Woł.) Mosyakin & Fedor.

*Minuartia verna* [unranked] α *caespitosa* (Ehrn.) Graebn. in Asch. & Graebn. sensu Tovt [ex herb.UU] * – ***Sabulina pauciflora*** (Kit.) A.Novikov, *comb. nov.*

*Minuartia verna* auct. flora carpat., non (L.) Hiern * - ***Sabulina pauciflora*** (Kit.) A.Novikov, *comb. nov.*

*Minuartia verna* Kulczyński, non (L.) Hiern – ***Sabulina pauciflora*** (Kit.) A.Novikov, *comb. nov.*

*Minuartia verna* subsp. *gerardii* (Willd.) Graebn. in Asch. & Graebn. [p.p., tantum quod plantas carpat.], non *Sabulina verna* subsp. *gerardii* (Willd.) Dillenb. s.str.* – ***Sabulina pauciflora*** (Kit.) A.Novikov, *comb. nov.*

*Minuartia verna* subsp. *gerardii* [unranked] b. *carpatica* (Porcius) Graebn. in Asch. & Graebn.* = ***Sabulina pauciflora*** (Kit.) A.Novikov, *comb. nov.*

*Minuartia verna* subsp. *oxypetala* (Woł.) G.Halliday *≡* ***Sabulina oxypetala*** (Woł.) Mosyakin & Fedor.

*Minuartia verna* var. *gerardi* Kulczyński, non Schinz. & Keller – ***Sabulina pauciflora*** (Kit.) A.Novikov, *comb. nov.*

*Minuartia verna* var. *oxypetala* (Woł.) Prodan *≡* ***Sabulina oxypetala*** (Woł.) Mosyakin & Fedor.

*Minuartia verna* var. *oxypetala* f. *micropetala* (Zapał.) Prodan = ***Sabulina oxypetala*** (Woł.) Mosyakin & Fedor.

*Minuartia zarecznyi* (Zapał.) Klokov = ***Sabulina pauciflora*** (Kit.) A.Novikov, *comb. nov.*

*Minuartia zarecznyi* var. *divestita* (Zapał.) Tzvelev = ***Sabulina pauciflora*** (Kit.) A.Novikov, *comb. nov.*

*Minuartia zarencznii* (Zapał.) Klokov [ortho. var.] = ***Sabulina pauciflora*** (Kit.) A.Novikov, *comb. nov.*

*Minuartia zarencznii* auct. [i.e., Chopyk 1976], non (Zapał.) Klokov * – ***Sabulina oxypetala*** (Woł.) Mosyakin & Fedor.

*Neocodon carpaticus* (Jacq.) Kolak. & Serdyuk *≡* ***Campanula carpatica*** Jacq., non *C. carpatha* Halácsy

*Nigritella angustifolia* var. *carpatica* Zapał. *≡* ***Gymnadenia carpatica*** (Zapał.) Teppner & E.Klein

*Nigritella carpatica* (Zapał.) Teppner, E.Klein & Zag.* *≡* ***Gymnadenia carpatica*** (Zapał.) Teppner & E.Klein

*Nigritella nigra* auct. fl. ucrain. carpat., non (L.) Rchb.f. * – ***Gymnadenia carpatica*** (Zapał.) Teppner & E.Klein

*Nigritella nigra* subsp. *carpatica* (Zapał.) H.Baumann & R.Lorenz *≡* ***Gymnadenia carpatica*** (Zapał.) Teppner & E.Klein

*Nigritella nigra* var. *carpatica* (Zapał.) Pawł. ≡ ***Gymnadenia carpatica*** (Zapał.) Teppner & E.Klein

*Nigritella rubra* f. *carpatica* (Zapał.) Soó *≡* ***Gymnadenia carpatica*** (Zapał.) Teppner & E.Klein

*Noccaea dacica* (Heuff.) F.K.Mey. ≡ ***Noccaea dacica*** subsp. ***dacica*** (Heuff.) F.K.Mey

***Noccaea dacica*** subsp. ***dacica*** (Heuff.) F.K.Mey

*Orobus laevigatus* Baumg., non Waldst. & Kit. – ***Lathyrus transsilvanicus*** (Spreng.) Rchb.f.

*Orobus luteus* subsp. *transsylvanicus* (Spreng.) Nyman [nom. et. des. inval.] *≡* ***Lathyrus transsilvanicus*** (Spreng.) Rchb.f.

*Orobus transsylvanicus* Spreng. ≡ ***Lathyrus transsilvanicus*** (Spreng.) Rchb.f.

*Pastinaca palmata* (Baumg.) Calest. = ***Heracleum sphondylium*** subsp. ***transsilvanicum*** (Schur) Brummitt

*Phyteuma atropurpureum* Schur [nom. nudum], non Hoppe ≡ ***Phyteuma vagneri*** A.Kern in Vágner

*Phyteuma betonicaefolium* Baumg. et auct. transsilv., non Vill. [nom. nudum ?] – ***Phyteuma vagneri*** A.Kern in Vágner

*Phyteuma halleri* auct. transsilv., non All. – ***Phyteuma vagneri*** A.Kern in Vágner

*Phyteuma michelii* Sternh., non alior = ***Phyteuma vagneri*** A.Kern in Vágner

*Phyteuma nigrum* auct. [e.g., Baumg.], non Schmalh. – ***Phyteuma vagneri*** A.Kern in Vágner

*Phyteuma nigrum* var. *atropurpureum* Schur ≡ ***Phyteuma vagneri*** A.Kern in Vágner

*Phyteuma ovatum* auct. [e.g., Baumg.], non Schmalh. – ***Phyteuma vagneri*** A.Kern in Vágner

*Phyteuma spicatum* Baumg., non L. nec Lapeyr. = ***Phyteuma tetramerium*** Schur

*Phyteuma spicatum* Nyman [nom. illeg.], non L. nec Lapeyr. *≡* ***Phyteuma tetramerium*** Schur

Phyteuma spicatum var. tetramerum (Schur) Nyman sensu auct. multipl. [nom. nudum] *–* ***Phyteuma tetramerium*** Schur

*Phyteuma spiciforme* Rochel [nom. nudum] * *≡* ***Phyteuma vagneri*** A.Kern in Vágner

*Phyteuma spiciforme* Rochel ex Domin & Podp. = ***Phyteuma vagneri*** A.Kern in Vágner

***Phyteuma tetramerium*** Schur

*Phyteuma tetramerum* Schur * [ortho. var.] *≡* ***Phyteuma tetramerium*** Schur

***Phyteuma vagneri*** A.Kern in Vágner *

*Phyteuma vagneri* A.Kern. ≡ ***Phyteuma vagneri*** A.Kern in Vágner

*Phyteuma vagneri* f. *alpinum* Rich.Schulz = ***Phyteuma vagneri*** A.Kern in Vágner

*Phyteuma vagneri* f. *brevibracteatum* Rich.Schulz = ***Phyteuma vagneri*** A.Kern in Vágner

*Phyteuma vagneri* f. *grossidentatum* Rich.Schulz = ***Phyteuma vagneri*** A.Kern in Vágner

*Phyteuma vagneri* f. *latibracteatum* Rich.Schulz = ***Phyteuma vagneri*** A.Kern in Vágner

*Phyteuma vagneri* var. *pallida* Porcius = ***Phyteuma vagneri*** A.Kern in Vágner

*Plantago alpina* Vill. sensu Rochel – ***Plantago atrata*** subsp. ***carpatica*** (Pilg.) Soó

*Plantago alpina* Vill. sensu Schur, non alior – ***Plantago atrata*** subsp. ***carpatica*** (Pilg.) Soó

*Plantago atrata* Hoppe [p.p., tantum quod plantas ucrain. carpat.] * – ***Plantago atrata*** subsp. ***carpatica*** (Pilg.) Soó

*Plantago atrata* subsp. *atrata* var. *carpathica* (Pilg.) Pilg. ≡ ***Plantago atrata*** subsp. ***carpatica*** (Pilg.) Soó

*Plantago atrata* subsp. *atrata* var. *carpathica* subvar. *rigidior* (Pilg.) Pilg. = ***Plantago atrata*** subsp. ***carpatica*** (Pilg.) Soó

*Plantago atrata* subsp. *atrata* var. *carpathica* subvar. *vestita* (Pilg.) Pilg. = ***Plantago atrata*** subsp. ***carpatica*** (Pilg.) Soó

*Plantago atrata* subsp. *carpathica* f. *vestita* (Pilg.) Soó = ***Plantago atrata*** subsp. ***carpatica*** (Pilg.) Soó

***Plantago atrata*** subsp. ***carpatica*** (Pilg.) Soó *

*Plantago atrata* var. *carpathica* f. *vestita* (Pilg.) Borza = ***Plantago atrata*** subsp. ***carpatica*** (Pilg.) Soó

*Plantago lanceolata* [unranked] β *alpestris* Wahlenb. = ***Plantago atrata*** subsp. ***carpatica*** (Pilg.) Soó

*Plantago montana* [unranked] *alpestre* Wahlenb. = ***Plantago atrata*** subsp. ***carpatica*** (Pilg.) Soó

*Plantago montana* Lam. sensu Schur, non alior – ***Plantago atrata*** subsp. ***carpatica*** (Pilg.) Soó

*Plantago montana* subsp. *atrata* var. *carpathica* Pilg. ≡ ***Plantago atrata*** subsp. ***carpatica*** (Pilg.) Soó

*Plantago montana* subsp. *atrata* var. *carpathica* subvar. *rigidior* Pilg. = ***Plantago atrata*** subsp. ***carpatica*** (Pilg.) Soó

*Plantago montana* subsp. *atrata* var. *carpathica* subvar. *vestita* Pilg. = ***Plantago atrata*** subsp. ***carpatica*** (Pilg.) Soó

*Plantago montana* subsp. *carpatica* (Pilg.) Soó ex Balázs *≡* ***Plantago atrata*** subsp. ***carpatica*** (Pilg.) Soó

*Plantago montana* subsp. *carpatica* subvar. *rigidior* (Pilg.) Balázs = ***Plantago atrata*** subsp. ***carpatica*** (Pilg.) Soó

*Plantago montana* subsp. *carpatica* subvar. *vestita* (Pilg.) Balázs = ***Plantago atrata*** subsp. ***carpatica*** (Pilg.) Soó

*Plantago saxatilis* M.Bieb. [p.p.] * – ***Plantago atrata*** subsp. ***carpatica*** (Pilg.) Soó

*Poa breazensis* Nyár. = ***Poa granitica*** subsp. ***disparillis*** (Nyár.) Nyár.

*Poa anceps* Rehmann, non G.Forst. ≡ ***Poa rehmannii*** (Asch. & Graebn.) Woł.

*Poa balfourii* auct. fl. ucrain. carpat., non Parn.* – ***Poa carpatica*** subsp. ***carpatica*** (V.Jirásek) Bernátová, Májovský, Kliment & Topercer

*Poa balfourii* f. *carpatica* Zapał. = ***Poa carpatica*** subsp. ***carpatica*** (V.Jirásek) Bernátová, Májovský, Kliment & Topercer

*Poa caesia* [unranked] d) *rehmanni* K.Richt. ≡ ***Poa rehmannii*** (Asch. & Graebn.) Woł.

*Poa carpatica* (V.Jirásek) Chopik *≡* ***Poa carpatica*** subsp. ***carpatica*** (V.Jirásek) Bernátová, Májovský, Kliment & Topercer

***Poa carpatica*** subsp. ***carpatica*** (V.Jirásek) Bernátová, Májovský, Kliment & Topercer

*Poa cenisia* [unranked] b *pietrosuana* Zapał. = ***Poa granitica*** subsp. ***disparillis*** (Nyár.) Nyár.

*Poa cenisia* All. [p.p., tantum quod plantas ucrain. carpat.] * – ***Poa granitica*** subsp. ***disparillis*** (Nyár.) Nyár.

*Poa cenisia* subsp. *granitica* var. *disparillis* (Nyár.) Nyár. & Borza *≡* ***Poa granitica*** subsp. ***disparillis*** (Nyár.) Nyár.

*Poa deylii* Chrtek & V.Jirásek * *=* ***Poa granitica*** subsp. ***disparillis*** (Nyár.) Nyár.

*Poa deylii* var. *deylii* f. *breazensis* (Nyár.) Ghișa & Beldie *=* ***Poa granitica*** subsp. ***disparillis*** (Nyár.) Nyár.

*Poa deylii* var. *deylii* f. *pietrosuana* (Zapał.) Ghișa & Beldie *=* ***Poa granitica*** subsp. ***disparillis*** (Nyár.) Nyár.

*Poa deylii* var. *deylii* f. *subgranitica* (Nyár.) Ghișa & Beldie *=* ***Poa granitica*** subsp. ***disparillis*** (Nyár.) Nyár.

*Poa deylii* subsp. *retezatensis* (A.Nyár.) Chrtek *=* ***Poa granitica*** subsp. ***disparillis*** (Nyár.) Nyár.

*Poa deylii* var. *retezatensis* (A.Nyár.) Ghișa & Beldie *=* ***Poa granitica*** subsp. ***disparillis*** (Nyár.) Nyár.

*Poa granitica* Braun-Blanq. [p.p., tantum quod plantas ucrain. carpat.] * – ***Poa granitica*** subsp. ***disparillis*** (Nyár.) Nyár.

***Poa granitica*** subsp. ***disparillis*** (Nyár.) Nyár. *

*Poa granitica* subsp. *disparillis* var. *subgranitica* Nyár. = ***Poa granitica*** subsp. ***disparillis*** (Nyár.) Nyár.

*Poa granitica* subsp. *granitica* Braun-Blanq. sensu Tasenkevich [non sensu orig., ex herb. LWS] * – ***Poa granitica*** subsp. ***disparillis*** (Nyár.) Nyár.

*Poa granitica* subsp. *retezatensis* A.Nyár. = ***Poa granitica*** subsp. ***disparillis*** (Nyár.) Nyár.

*Poa granitica* subsp. *subcarpatica* (V.Jirásek) Fodor *=* ***Poa granitica*** subsp. ***disparillis*** (Nyár.) Nyár.

*Poa granitica* var. *disparillis* f. *pietrosuana* (Zapał.) Nyár. = ***Poa granitica*** subsp. ***disparillis*** (Nyár.) Nyár.

*Poa granitica* var. *disparillis* Nyár. ≡ ***Poa granitica*** subsp. ***disparillis*** (Nyár.) Nyár.

*Poa granitica* var. *subcarpatica* V.Jirásek *=* ***Poa granitica*** subsp. ***disparillis*** (Nyár.) Nyár.

*Poa granitica* var. *typica* f. *deminuta* Nyár. = ***Poa granitica*** subsp. ***disparillis*** (Nyár.) Nyár.

*Poa granitica* var. *typica* Nyár. = ***Poa granitica*** subsp. ***disparillis*** (Nyár.) Nyár.

Poa janczewskii auct., non Zapał. [tantum quod plantas ucrain. carpat., alp.et subalp. altitud. solum] – ***Poa carpatica*** subsp. ***carpatica*** (V.Jirásek) Bernátová, Májovský, Kliment & Topercer

*Poa nemoralis* subsp. *carpatica* f. *minoriformis* V.Jirásek = ***Poa carpatica*** subsp. ***carpatica*** (V.Jirásek) Bernátová, Májovský, Kliment & Topercer

*Poa nemoralis* subsp. *carpatica* V.Jirásek * *≡* ***Poa carpatica*** subsp. ***carpatica*** (V.Jirásek) Bernátová, Májovský, Kliment & Topercer

*Poa nemoralis* subsp. *montana* auct., non (Gaudin) Chrtek & V.Jirásek – ***Poa carpatica*** subsp. ***carpatica*** (V.Jirásek) Bernátová, Májovský, Kliment & Topercer

*Poa nemoralis* subsp. *nemoralis* var. *carpatica* (V.Jirásek) Soó *≡* ***Poa carpatica*** subsp. ***carpatica*** (V.Jirásek) Bernátová, Májovský, Kliment & Topercer

*Poa nemoralis* subsp. *rehmannii* Asch. & Graebn. ≡ ***Poa rehmannii*** (Asch. & Graebn.) Woł.

*Poa nemoralis* var. *montana* auct. fl. ucrain. carpat., non Gaudin * – ***Poa carpatica*** subsp. ***carpatica*** (V.Jirásek) Bernátová, Májovský, Kliment & Topercer

***Poa pannonica*** subsp. ***scabra*** (Asch.) Soó

*Poa perscabra* Holub = ***Poa pannonica*** subsp. ***scabra*** (Asch.) Soó

*Poa pratensis* var. *scabra* (Asch.) Asch. & Graebn. *≡* ***Poa pannonica*** subsp. ***scabra*** (Asch.) Soó

***Poa rehmannii*** (Asch. & Graebn.) Woł.*

*Poa rehmannii* (Asch. & Graebn.) K.Richt. [nom. nudum] *≡* ***Poa rehmannii*** (Asch. & Graebn.) Woł.

*Poa rehmannii* Asch. & Gürke sensu Woł. [nom. confus., ex herb. LWS] * *–* ***Poa rehmannii*** (Asch. & Graebn.) Woł.

*Poa scabra* Asch., non Ehrh.* *≡* ***Poa pannonica*** subsp. ***scabra*** (Asch.) Soó

*Poa scabra* Kit. ex Steud. [nom. nudum], non Ehrh. ≡ ***Poa pannonica*** subsp. ***scabra*** (Asch.) Soó

*Poa sterilis* Kerner, non M.Bieb. = ***Poa pannonica*** subsp. ***scabra*** (Asch.) Soó

*Poa sterilis* subsp. *eu-sterilis* var. *scabra* (Asch.) Asch. & Graebn., non alior *≡* ***Poa pannonica*** subsp. ***scabra*** (Asch.) Soó

*Ptarmica oxyloba* Schur, non *Achillea oxyloba* (DC.) Sch.Bip. = ***Achillea oxyloba* subsp. *schurii*** (Sch.Bip.) Heimerl

*Ptarmica pseudo-atrata* Schur ex Schur = ***Achillea oxyloba* subsp. *schurii*** (Sch.Bip.) Heimerl

*Ptarmica schurii* Sch.Bip. ≡ ***Achillea oxyloba* subsp. *schurii*** (Sch.Bip.) Heimerl

*Ptarmica tenuifolia* (Schur) Schur, non *Achillea tenuifolia* Lam.* *≡* ***Achillea oxyloba* subsp. *schurii*** (Sch.Bip.) Heimerl

Ptarmica tenuifolia [unranked] a macrocephala Schur, non alior *≡* ***Achillea oxyloba* subsp. *schurii*** (Sch.Bip.) Heimerl

*Ptarmica tenuifolia* [unranked] b *polycephala* (Schur) Schur = ***Achillea oxyloba* subsp. *schurii*** (Sch.Bip.) Heimerl

*Pulmonaria angustifolia* Kern. [p.p., quoad plantas marmaros. et rodn.], non L. – ***Pulmonaria filarszkyana*** Jáv.

*Pulmonaria dacica* (Simonk.) Porcius [p.p., nom et des. invalid] – ***Pulmonaria filarszkyana*** Jáv.

*Pulmonaria dacica* (Simonk.) Simonk. [p.p.] * – ***Pulmonaria filarszkyana*** Jáv.

***Pulmonaria filarszkyana*** Jáv.*

*Pulmonaria rubra* subsp. *filarszkyana* (Jáv.) Domin *≡* ***Pulmonaria filarszkyana*** Jáv.

*Pulmonaria rubra* var. *dacica* Simonk. [p.p.] – ***Pulmonaria filarszkyana*** Jáv.

*Pulmonaria rubra* var. *filarszkyana* (Jáv.) Guşul.* *≡* ***Pulmonaria filarszkyana*** Jáv.

*Pyrethrum waldsteinii* (Sch.Bip.) Janka = ***Leucanthemum rotundifolium*** (Waldst. & Kit. in Willd.) DC., non Opiz

***Pyrola carpatica*** Holub & Křísa

*Pyrola intermedia* auct., non Schleich. ex Arcang. – ***Pyrola carpatica*** Holub & Křísa

*Pyrola intermedia* Schleich. sensu Szafer in Kulczyński & Pawłowski [nom. illeg.], non Schleich. ex Arcang.

*Pyrola rotundifolia* [unranked] *arenaria* Scheele sensu Jáv. – ***Pyrola carpatica*** Holub & Křísa

*Pyrola rotundifolia* subsp. *carpatica* (Holub & Křísa) Váczy & Beldie *≡* ***Pyrola carpatica*** Holub & Křísa

*Pyrola rotundifolia* subsp. in*termedia* (Alef.) Wohlfahrt in W.D.J.Koch [p.p., tantum quod plantas carpat.] *–* ***Pyrola carpatica*** Holub & Křísa

*Pyrola rotundifolia* subsp. in*termedia* (Schleich.) Dostál [p.p., tantum quod plantas carpat., excl. var. *arenaria* Koch; nom. illeg.] *–* ***Pyrola carpatica*** Holub & Křísa

*Ranunculus aduncus* Schur, non Gren. & Godr. = ***Ranunculus carpaticus*** Herbich, non Wahlenb. ex Nyman

*Ranunculus carpaticus* f. *anomalus* A.Nyár. = ***Ranunculus carpaticus*** Herbich, non Wahlenb. ex Nyman

*Ranunculus carpaticus* f. *flabellatus* A.Nyár. = ***Ranunculus carpaticus*** Herbich, non Wahlenb. ex Nyman

*Ranunculus carpaticus* f. *plenus* Zapał. = ***Ranunculus carpaticus*** Herbich, non Wahlenb. ex Nyman

*Ranunculus carpaticus* f. *pygmaeus* Porcius = ***Ranunculus carpaticus*** Herbich, non Wahlenb. ex Nyman

***Ranunculus carpaticus*** Herbich, non Wahlenb. ex Nyman *

*Ranunculus carpaticus* var. *rupicolus* Zapał. = ***Ranunculus carpaticus*** Herbich, non Wahlenb. ex Nyman

*Ranunculus dentatus* (Baumg.) Freyn in A.Kern.* = ***Ranunculus carpaticus*** Herbich, non Wahlenb. ex Nyman

*Ranunculus gouani* Baumg., non alior = ***Ranunculus carpaticus*** Herbich, non Wahlenb. ex Nyman

*Ranunculus kladnii* auct. fl. ucrain. carpat., non Schur * = ***Ranunculus malinovskii*** Jelenevsky & Derv.-Sok.

*Ranunculus lerchenfeldianus* Schur = ***Ranunculus carpaticus*** Herbich, non Wahlenb. ex Nyman

***Ranunculus malinovskii*** Jelenevsky & Derv.-Sok.

*Ranunculus montanus* Willd. [unranked] α *dentatus* Baumg. = ***Ranunculus carpaticus*** Herbich, non Wahlenb. ex Nyman

*Ranunculus pormbachiensis* Lerchenf. ex Schur = ***Ranunculus carpaticus*** Herbich, non Wahlenb. ex Nyman

*Ranunculus schurii* Fuss ex Schur = ***Ranunculus carpaticus*** Herbich, non Wahlenb. ex Nyman

*Ranunculus szurulensis* Lerchenf. ex Schur [p.p.] – ***Ranunculus carpaticus*** Herbich, non Wahlenb. ex Nyman

*Ranunculus tuberosus* Schur, non alior. = ***Ranunculus carpaticus*** Herbich, non Wahlenb. ex Nyman

*Sabulina gerardii* auct. fl. carpat., non (Willd.) Rchb. – ***Sabulina pauciflora*** (Kit.) A.Novikov, *comb. nov.*

***Sabulina oxypetala*** (Woł.) Mosyakin & Fedor.

***Sabulina pauciflora*** (Kit.) A.Novikov, *comb. nov.*

*Sabulina verna* subsp. *gerardii* auct. fl. carpat., non (Willd.) Dillenb. – ***Sabulina pauciflora*** (Kit.) A.Novikov, *comb. nov.*

*Sabulina verna* subsp. *oxypetala* (Woł.) Dillenb. & Kadereit *≡* ***Sabulina oxypetala*** (Woł.) Mosyakin & Fedor.

***Salix kitaibeliana*** Willd.*

*Salix retusa* [unranked] b *serrulata* Roch. = ***Salix kitaibeliana*** Willd.

*Salix retusa* [unranked] β *major* W.D.J.Koch [nom. superfl.] = ***Salix kitaibeliana*** Willd.

*Salix retusa* [unranked] γ *kitaibeliana* (Willd.) Rchb. ≡ ***Salix kitaibeliana*** Willd.

*Salix retusa* f. *kitaibeliana* (Willd.) Rouy * *≡* ***Salix kitaibeliana*** Willd.

*Salix retusa* subsp. *kitaibeliana* (Willd.) Jáv.* *≡* ***Salix kitaibeliana*** Willd.

*Salix retusa* var. *major* Rchb. = ***Salix kitaibeliana*** Willd.

Salix retusa var. serrulata Roch. ex Rchb. = ***Salix kitaibeliana*** Willd.

*Saussurea alata* Porcius & Czetz, non DC. = ***Saussurea porcii*** Degen

*Saussurea parviflora* auct., non (Poir.) DC. – ***Saussurea porcii*** Degen

***Saussurea porcii*** Degen *

*Saussurea serrata* auct. Transsilv., non DC. – ***Saussurea porcii*** Degen

*Saussurea serrata* Janka, non DC. = ***Saussurea porcii*** Degen

*Scabiosa barbata* Nyár. ex Chopyk & Fedoronchuk [des. et nom. invalid.] * *≡* ***Scabiosa lucida*** subsp. ***barbata*** Nyár.

*Scabiosa columbaria* subsp. *lucida* var. *subalpina* (Brügger) Braun-Blanq. = ***Scabiosa lucida*** subsp. ***barbata*** Nyár.

*Scabiosa columbaria* subsp. *subalpina* (Brügger) Killias = ***Scabiosa lucida*** subsp. ***barbata*** Nyár.

*Scabiosa columbaria* subsp. *subalpina* Brügger [nom. nudum] = ***Scabiosa lucida*** subsp. ***barbata*** Nyár.

*Scabiosa lucida* f. *elata* Nyár. = ***Scabiosa lucida*** subsp. ***barbata*** Nyár.

*Scabiosa lucida* f. *hirticaulis* Nyár. = ***Scabiosa lucida*** subsp. ***barbata*** Nyár.

*Scabiosa lucida* f. *perramosa* Nyár. = ***Scabiosa lucida*** subsp. ***barbata*** Nyár.

*Scabiosa lucida* f. *scaposa* Nyár. = ***Scabiosa lucida*** subsp. ***barbata*** Nyár.

*Scabiosa lucida* subsp. *barbata* f. *alpicola* (Schur) Prodan = ***Scabiosa lucida*** subsp. ***barbata*** Nyár.

*Scabiosa lucida* subsp. *barbata* f. *hirticaulis* (Nyár.) Prodan = ***Scabiosa lucida*** subsp. ***barbata*** Nyár.

*Scabiosa lucida* subsp. *barbata* f. *perramosa* (Nyár.) Prodan = ***Scabiosa lucida*** subsp. ***barbata*** Nyár.

*Scabiosa lucida* subsp. *barbata* f. *subalpina* (Schur) Prodan = ***Scabiosa lucida*** subsp. ***barbata*** Nyár.

***Scabiosa lucida*** subsp. ***barbata*** Nyár.*

*Scabiosa lucida* subsp. *lucida* Vill. sensu Tasenkevych [non sensu orig.] – ***Scabiosa lucida*** subsp. ***barbata*** Nyár.

*Scabiosa lucida* var. *subalpina* (Brügger) Hayek & Hegi = ***Scabiosa lucida*** subsp. ***barbata*** Nyár.

*Scabiosa lucida* Vill. [p.p., tantum quod plantas ucrain. carpat.], non W.T.Aiton * – ***Scabiosa lucida*** subsp. ***barbata*** Nyár.

*Scabiosa opaca* Klokov * = ***Scabiosa lucida*** subsp. ***barbata*** Nyár.

*Scabiosa pseudobanatica* subsp. *barbata* (Nyár.) Chrtek ***Scabiosa lucida*** subsp. ***barbata*** Nyár.

*Scabiosa subalpina* Brügger = ***Scabiosa lucida*** subsp. ***barbata*** Nyár.

*Scilla alpina* Schur = ***Scilla kladnii*** Schur

*Scilla bifolia* L. [p.p., tantum quod plantas ucrain. carpat.], non alior * – ***Scilla kladnii*** Schur

*Scilla bifolia* subsp. *alpina* (Schur) Nyman = ***Scilla kladnii*** Schur

*Scilla bifolia* subsp. *nivalis* (Boiss.) K.Richt. sensu Fodor [non sensu orig.] – ***Scilla kladnii*** Schur

*Scilla bifolia* subsp. *subtriphylla* (Schur) Domin = ***Scilla kladnii*** Schur

*Scilla bifolia* var. *alpina* (Schur) C.Zahariadi in Nyár. = ***Scilla kladnii*** Schur

*Scilla bifolia* var. *kladnii* (Schur) Nyman *≡* ***Scilla kladnii*** Schur

*Scilla bifolia* var. *nivalis* auct. fl. carpat, non Baker – ***Scilla kladnii*** Schur

*Scilla bifolia* var. *subtriphylla* (Schur) T.Simon = ***Scilla kladnii*** Schur

*Scilla bifolia* β [unranked] *gracillima* Grecescu, non alior = ***Scilla kladnii*** Schur

***Scilla kladnii*** Schur *

*Scilla praecox* auct. fl. carpat, non Willd. – ***Scilla kladnii*** Schur

*Scilla subtriphylla* Schur * = ***Scilla kladnii*** Schur

*Scilla trifolia* Schur = ***Scilla kladnii*** Schur

*Scorzoneroides montana* (Lam.) J.Holub subsp. *pseudotaraxaci* [des. et nom. inval.] *≡* ***Scorzoneroides pseudotaraxaci*** (Schur) Holub

***Scorzoneroides pseudotaraxaci*** (Schur) Holub

*Sempervivum arachnoideum* auct. [e.g., G.Reuss], non L. – ***Sempervivum carpathicum*** subsp. ***carpathicum*** Wettst. ex Prodan

***Sempervivum carpathicum*** subsp. ***carpathicum*** Wettst. ex Prodan

*Sempervivum carpathicum* Wettst. ex Prodan ≡ ***Sempervivum carpathicum*** subsp. ***carpathicum*** Wettst. ex Prodan

*Sempervivum carpathicum* Wettst. in A.Kern. [nom. nudum] ≡ ***Sempervivum carpathicum*** subsp. ***carpathicum*** Wettst. ex Prodan

***Sempervivum globiferum*** subsp. ***preissianum*** (Domin) M.Werner

*Sempervivum heterophyllum* Jáv., non Haszl. – ***Sempervivum carpathicum*** subsp. ***carpathicum*** Wettst. ex Prodan

*Sempervivum hirtum* f. *glabrescens* Sabr. = ***Sempervivum globiferum*** subsp. ***preissianum*** (Domin) M.Werner

*Sempervivum hirtum* subsp. *glabrescens* (Sabr.) Jáv. = ***Sempervivum globiferum*** subsp. ***preissianum*** (Domin) M.Werner

*Sempervivum hirtum* subsp. *preissianum* (Domin) Dostál *≡* ***Sempervivum globiferum*** subsp. ***preissianum*** (Domin) M.Werner

*Sempervivum hirtum* subsp. *tatrense* (Domin) Dostál = ***Sempervivum globiferum*** subsp. ***preissianum*** (Domin) M.Werner

*Sempervivum montanum* f. *brachypetalum* Domin = ***Sempervivum carpathicum*** subsp. ***carpathicum*** Wettst. ex Prodan

*Sempervivum montanum* f. *congestum* Domin = ***Sempervivum carpathicum*** subsp. ***carpathicum*** Wettst. ex Prodan

*Sempervivum montanum* f. *neopallidum* Hadrava & Miklánek [nom. illeg.] = ***Sempervivum carpathicum*** subsp. ***carpathicum*** Wettst. ex Prodan

*Sempervivum montanum* f. *pallidum* (Wettst. ex Hayek) Domin [comb.illeg.] = ***Sempervivum carpathicum*** subsp. ***carpathicum*** Wettst. ex Prodan

*Sempervivum montanum* f. *pallidum* (Wettst. ex Hayek) Fiori = ***Sempervivum carpathicum*** subsp. ***carpathicum*** Wettst. ex Prodan

Sempervivum montanum f. pallidum (Wettst. ex Hayek) Hadrava & Miklánek [nom. illeg.] = ***Sempervivum carpathicum*** subsp. ***carpathicum*** Wettst. ex Prodan

*Sempervivum montanum* f. *speciosum* Domin = ***Sempervivum carpathicum*** subsp. ***carpathicum*** Wettst. ex Prodan

*Sempervivum montanum* f. *stenophyllum* Domin = ***Sempervivum carpathicum*** subsp. ***carpathicum*** Wettst. ex Prodan

*Sempervivum montanum* L. [p.p., tantum quod plantas ucrain. carpat.], non alior – ***Sempervivum carpathicum*** subsp. ***carpathicum*** Wettst. ex Prodan

*Sempervivum montanum* subsp. *carpathicum* (Wettst. ex Prodan) A.Berger in Engler & Prantl ≡ ***Sempervivum carpathicum*** subsp. ***carpathicum*** Wettst. ex Prodan

*Sempervivum montanum* subsp. *carpaticum* Wettst. ex Hayek in Hegi [nom. nudum] ≡ ***Sempervivum carpathicum*** subsp. ***carpathicum*** Wettst. ex Prodan

*Sempervivum montanum* subsp. *carpaticum* Wettst. in Sched. [nom. nudum] ≡ ***Sempervivum carpathicum*** subsp. ***carpathicum*** Wettst. ex Prodan

*Sempervivum montanum* subsp. *debile* auct., non (Schott.) Dostál – ***Sempervivum carpathicum*** subsp. ***carpathicum*** Wettst. ex Prodan

*Sempervivum montanum* subsp. *eumontanum* var. *carpathicum* (Wettst. ex Prodan) Domin ≡ ***Sempervivum carpathicum*** subsp. ***carpathicum*** Wettst. ex Prodan

*Sempervivum montanum* subsp. *heterophyllum* auct., non (Haszl.) Jáv. ex Soó – ***Sempervivum carpathicum*** subsp. ***carpathicum*** Wettst. ex Prodan

*Sempervivum montanum* subsp. montanum auct. [e.g., Pawłowski, Dostál, Lippert], non L. – ***Sempervivum carpathicum*** subsp. ***carpathicum*** Wettst. ex Prodan

*Sempervivum montanum* var. *carpathicum* (Wettst. ex Prodan) Praeger [comb. inval.] ≡ ***Sempervivum carpathicum*** subsp. ***carpathicum*** Wettst. ex Prodan

*Sempervivum montanum* var. *pallidum* Wettst. ex Hayek in Hegi [nom. inval.] = ***Sempervivum carpathicum*** subsp. ***carpathicum*** Wettst. ex Prodan

*Sempervivum montanum* var. *pallidum* Wettst. ex Schinz & R.Keller = ***Sempervivum carpathicum*** subsp. ***carpathicum*** Wettst. ex Prodan

*Sempervivum preissianum* Domin * *≡* ***Sempervivum globiferum*** subsp. ***preissianum*** (Domin) M.Werner

*Sempervivum soboliferum* Sims [p.p., tantum quod plantas ucrain. carpat.], non Fleisch. & Lindem.* – ***Sempervivum globiferum*** subsp. ***preissianum*** (Domin) M.Werner

*Sempervivum soboliferum* subsp. *preissianum* (Domin) Pawłowska *≡* ***Sempervivum globiferum*** subsp. ***preissianum*** (Domin) M.Werner = ***Sempervivum globiferum*** subsp. ***preissianum*** (Domin) M.Werner

*Sempervivum soboliferum* subsp. *preissianum* f. *minus* Domin ex Pawłowska = ***Sempervivum globiferum*** subsp. ***preissianum*** (Domin) M.Werner

*Sempervivum soboliferum* subsp. *preissianum* var. *tatrense* (Domin) Pawłowska = ***Sempervivum globiferum*** subsp. ***preissianum*** (Domin) M.Werner

*Sempervivum tatrense* Domin = ***Sempervivum globiferum*** subsp. ***preissianum*** (Domin) M.Werner

*Sempervivum wettsteinii* subsp. *wettsteinii* Letz [nom. invalid.] = ***Sempervivum carpathicum*** subsp. ***carpathicum*** Wettst. ex Prodan

***Senecio hercynicus*** subsp. ***ucranicus*** (Hodálová) Greuter

*Senecio ucranicus* Hodálová, non Besser.* *≡* ***Senecio hercynicus*** subsp. ***ucranicus*** (Hodálová) Greuter

***Sesleria bielzii*** Schur *

*Sesleria caerulea* [unranked] a *interrupta* Schur = ***Sesleria heufleriana*** subsp. ***heufleriana*** Schur

*Sesleria caerulea* [unranked] b *prorepens* Schur = ***Sesleria heufleriana*** subsp. ***heufleriana*** Schur

*Sesleria caerulea* [unranked] c *praelonga* Schur = ***Sesleria heufleriana*** subsp. ***heufleriana*** Schur

*Sesleria caerulea* Baumg. [p.p.], non (L.) Ard. – ***Sesleria heufleriana*** subsp. ***heufleriana*** Schur

*Sesleria caerulea* Janka, non Ard. = ***Sesleria bielzii*** Schur

*Sesleria caerulea* Scap. sensu Rehman [nom. confus. ex herb. LWS] * – ***Sesleria bielzii*** Schur

*Sesleria caerulea* var. *transilvanica* (Schur) Jáv. = ***Sesleria heufleriana*** subsp. ***heufleriana*** Schur

*Sesleria capitata* (Schur) Schur = ***Sesleria bielzii*** Schur

*Sesleria coerulans* f. *pseudorigida* (Schur) Beldie = ***Sesleria bielzii*** Schur

*Sesleria coerulans* Friv. [p.p., tantum quod plantas ucrain. carpat.] * – ***Sesleria bielzii*** Schur

*Sesleria coerulans* subsp. *bielzii* (Schur) Gergely & Beldie * *≡* ***Sesleria bielzii*** Schur

*Sesleria coerulans* var. *borsae* Deyl = ***Sesleria bielzii*** Schur

*Sesleria haynaldiana* [unanked] g *pseudorigida* Schur = ***Sesleria bielzii*** Schur

*Sesleria heufleriana* [unranked] a *polydactyla* Schur = ***Sesleria heufleriana*** subsp. ***heufleriana*** Schur

*Sesleria heufleriana* [unranked] a *praelonga* Schur = ***Sesleria heufleriana*** subsp. ***heufleriana*** Schur

*Sesleria heufleriana* [unranked] c *elongata* Schur, non Host = ***Sesleria heufleriana*** subsp. ***heufleriana*** Schur

*Sesleria heufleriana* [unranked] b *digitata* Schur = ***Sesleria heufleriana*** subsp. ***heufleriana*** Schur

*Sesleria heufleriana* f. in*terrupta* (Schur) Soó = ***Sesleria heufleriana*** subsp. ***heufleriana*** Schur

*Sesleria heufleriana* f. *praelonga* (Schur) Gergely & Beldie = ***Sesleria heufleriana*** subsp. ***heufleriana*** Schur

*Sesleria heufleriana* f. *prorepens* (Schur) Soó = ***Sesleria heufleriana*** subsp. ***heufleriana*** Schur

*Sesleria heufleriana* Schur * – ***Sesleria heufleriana*** subsp. ***heufleriana*** Schur

*Sesleria heufleriana* Schur ex Błocki [nom. inval.] *≡* ***Sesleria heufleriana*** subsp. ***heufleriana*** Schur

***Sesleria heufleriana*** subsp. ***heufleriana*** Schur

*Sesleria heufleriana* var. *insignis* Schur = ***Sesleria heufleriana*** subsp. ***heufleriana*** Schur

*Sesleria nitida* Heldr. ex Nyman [nom. illeg.], non Ten. = ***Sesleria heufleriana*** subsp. ***heufleriana*** Schur

*Sesleria prorepens* Schur ex Schur = ***Sesleria heufleriana*** subsp. ***heufleriana*** Schur

*Sesleria pseudorigida* Schur = ***Sesleria bielzii*** Schur

*Sesleria rigida* [unranked] a *capitata* Schur = ***Sesleria bielzii*** Schur

*Sesleria rigida* [unranked] b *ovoidea* Schur = ***Sesleria bielzii*** Schur

*Sesleria rigida* [unranked] β *bielzii* (Schur) Heuff. ≡ ***Sesleria bielzii*** Schur

*Sesleria rigida* Griseb., non Heuff. ex Rchb. = ***Sesleria bielzii*** Schur

*Sesleria robusta* Pávai [nom. nudum], non Schott et al. = ***Sesleria heufleriana*** subsp. ***heufleriana*** Schur

*Sesleria transilvanica* Schur = ***Sesleria heufleriana*** subsp. ***heufleriana*** Schur

*Silenanthe zawadzkii* (Herbich) Griseb. & Schenk *≡* ***Silene zawadzkii*** Herbich

*Silene dubia* Herbich, non alior *≡* ***Silene nutans* subsp. *dubia*** (Herbich) Zapał.

*Silene dubia* Herbich ex Rohrb., non alior * *≡* ***Silene nutans* subsp. *dubia*** (Herbich) Zapał.

*Silene dubia* var. *glabriuscula* (Zapał.) Guşul. = ***Silene nutans* subsp. *dubia*** (Herbich) Zapał.

*Silene dubia* var. *hormuzakii* f. *acaulis* Guşul. [monster forma] = ***Silene nutans* subsp. *dubia*** (Herbich) Zapał.

*Silene dubia* var. *hormuzakii* f. *apricorum* (Zapał.) Graebn. in Asch. & Graebn.emend. Guşul. = ***Silene nutans* subsp. *dubia*** (Herbich) Zapał.

*Silene dubia* var. *hormuzakii* f. *herbichii* (Zapał.) Graebn. in Asch. & Graebn.emend. Guşul. = ***Silene nutans* subsp. *dubia*** (Herbich) Zapał.

*Silene dubia* var. *hormuzakii* f. *kelemenensis* (Zapał.) Graebn. in Asch. & Graebn.emend. Guşul. = ***Silene nutans* subsp. *dubia*** (Herbich) Zapał.

*Silene dubia* var. *hormuzakii* f. *lilacina* (Zapał.) Guşul. = ***Silene nutans* subsp. *dubia*** (Herbich) Zapał.

*Silene dubia* var. *hormuzakii* f. *robustior* (Schur) Graebn. in Asch. & Graebn.emend. Guşul. = ***Silene nutans* subsp. *dubia*** (Herbich) Zapał.

*Silene dubia* var. *hormuzakii* Guşul. = ***Silene nutans* subsp. *dubia*** (Herbich) Zapał.

*Silene nutans* [unanked] β *transsilvanica* Grec. = ***Silene nutans* subsp. *dubia*** (Herbich) Zapał.

*Silene nutans* [unranked] c.*glabriuscula* Zapał. = ***Silene nutans* subsp. *dubia*** (Herbich) Zapał.

***Silene nutans* subsp. *dubia*** (Herbich) Zapał.*

*Silene nutans* subsp. *dubia* [unranked] a *kelemenensis* f. *lilacina* Zapał. = ***Silene nutans* subsp. *dubia*** (Herbich) Zapał.

*Silene nutans* subsp. *dubia* [unranked] a *kelemenensis* Zapał. = ***Silene nutans* subsp. *dubia*** (Herbich) Zapał.

*Silene nutans* subsp. *dubia* [unranked] b *herbichii* Zapał. = ***Silene nutans* subsp. *dubia*** (Herbich) Zapał.

*Silene nutans* subsp. *dubia* var. *dubia* f. *apricorum* Zapał. = ***Silene nutans* subsp. *dubia*** (Herbich) Zapał.

*Silene nutans* subsp. *dubia* var. *dubia* f. *luxuriosa* Zapał. = ***Silene nutans* subsp. *dubia*** (Herbich) Zapał.

*Silene nutans* subsp. *dubia* var. *dubia* f. *tenuis* Zapał. = ***Silene nutans* subsp. *dubia*** (Herbich) Zapał.

*Silene nutans* var. *dubia* (Herbich) Zapał. ≡ ***Silene nutans* subsp. *dubia*** (Herbich) Zapał.

Silene saxatilis [unranked] a *racemosa* Schur = ***Silene nutans* subsp. *dubia*** (Herbich) Zapał.

*Silene saxatilis* [unranked] a *robustior* Schur = ***Silene nutans* subsp. *dubia*** (Herbich) Zapał.

*Silene saxatilis* Schur, non Sims nec M.Bieb. – ***Silene nutans* subsp. *dubia*** (Herbich) Zapał.

*Silene transsilvanica* Schur = ***Silene nutans* subsp. *dubia*** (Herbich) Zapał.

*Silene transsilvanica* var. *angustifolia* Hormuz. = ***Silene nutans* subsp. *dubia*** (Herbich) Zapał.

***Silene zawadzkii*** Herbich *

Soldanella alpina [unranked] minor Clus. [p.p.] – ***Soldanella hungarica*** Simonk. [s.str.et s.l.]

*Soldanella alpina* [unranked] a *minor* Schur [p. p., nom. illeg.], non Seringe * – ***Soldanella hungarica*** Simonk. [s.str.et s.l.]

*Soldanella alpina* [unranked] α *major* Neilr.* *≡* ***Soldanella major*** (Neilr.) Vierh. in Urban & Graebn., emend. Zhang & Kadereit – ***Soldanella hungarica*** Simonk. [s.l.]

*Soldanella alpina* [unranked] β *minor* (Clus.) Neilr. [p. p.], non Seringe – ***Soldanella hungarica*** Simonk. [s.str.et s.l.]

*Soldanella alpina* var. *hungarica* (Simonk.) Stojanoff & Stefanoff *≡* ***Soldanella hungarica*** Simonk. [s.str.et s.l.]

*Soldanella alpina* var. *vulgaris* Seringe [p.p., nom. inval.] – ***Soldanella major*** (Neilr.) Vierh. in Urban & Graebn., emend. Zhang & Kadereit – ***Soldanella hungarica*** Simonk. [s.l.]

*Soldanella haretii* Grinţ. = ***Soldanella marmarossiensis*** Klášt., emend. Zhang & Kadereit – ***Soldanella hungarica*** Simonk. [s.l.]

***Soldanella hungarica*** Simonk. [s.str.] * = ***Soldanella hungarica*** Simonk. [s.l.]

*Soldanella hungarica* subsp. *hungarica* Simonk. [s.str.] *≡* ***Soldanella hungarica*** Simonk. [s.str.] * = ***Soldanella hungarica*** Simonk. [s.l.]

*Soldanella hungarica* subsp. *major* (Neilr.) Pawłowska [p.p.] * = ***Soldanella major*** (Neilr.) Vierh. in Urban & Graebn., emend. Zhang & Kadereit – ***Soldanella hungarica*** Simonk. [s.l.]

*Soldanella hungarica* var. *minor* (Schur) Vierh. in Hannig & Winkler [p.p.] – ***Soldanella hungarica*** Simonk. [s.str.] = ***Soldanella hungarica*** Simonk. [s.l.]

***Soldanella major*** (Neilr.) Vierh. in Urban & Graebn., emend. Zhang & Kadereit * = ***Soldanella hungarica*** Simonk. [s.l.]

*Soldanella major* f. *haretii* (Grinţ.) Guşul. in Morariu, Nyár. & Guşul. = ***Soldanella marmarossiensis*** Klášt., emend. Zhang & Kadereit – ***Soldanella hungarica*** Simonk. [s.l.]

*Soldanella major* f. *macrocarpa* Morariu in Morariu, Nyár. & Guşul. [p.p., nom. inval.] – ***Soldanella hungarica*** Simonk. [s.str.] = ***Soldanella hungarica*** Simonk. [s.l.]

*Soldanella major* f. *parviflora* Morariu in Morariu, Nyár. & Guşul. [nom. inval.] = ***Soldanella hungarica*** Simonk. [s. str. et s.l.]

*Soldanella major* f. *purpureifolia* R.Rös. [nom. inval.] = ***Soldanella hungarica*** Simonk. [s.str. et s.l.]

*Soldanella major* subsp. *margittaniana* Fodor [nom. nudum, ex herb. UU] * – ***Soldanella major*** (Neilr.) Vierh. in Urban & Graebn., emend. Zhang & Kadereit – ***Soldanella hungarica*** Simonk. [s.l.]

*Soldanella major* Vierh. f. *hungarica* (Simonk.) Jáv. [p.p.] *–* ***Soldanella hungarica*** Simonk. [s.str.] = ***Soldanella hungarica*** Simonk. [s.l.]

***Soldanella marmarossiensis*** Klášt., emend. Zhang & Kadereit ***** – ***Soldanella hungarica*** Simonk. [s.l.]

*Soldanella montana* subsp. *faceta* A.Kress [p.p.] – ***Soldanella marmarossiensis*** Klášt., emend. Zhang & Kadereit – ***Soldanella hungarica*** Simonk. [s.l.]

*Soldanella montana* subsp. *hungarica* var. *major* (Neilr.) Lüdi in Hegi *≡* ***Soldanella major*** (Neilr.) Vierh. in Urban & Graebn., emend. Zhang & Kadereit – ***Soldanella hungarica*** Simonk. [s.l.]

*Soldanella montana* subsp. *hungarica* var. *marmarossiensis* (Klášt.) Fodor [p.p.] – ***Soldanella marmarossiensis*** Klášt., emend. Zhang & Kadereit – ***Soldanella hungarica*** Simonk. [s.l.]

*Soldanella montana* subsp. *hungarica* var. *minor* (Schur) Vierch. [nom. inval., ex herb. LWS] * – ***Soldanella hungarica*** Simonk. [s.str.] = ***Soldanella hungarica*** Simonk. [s.l.]

*Soldanella montana* subsp. *hungarica* (Simonk.) Lüdi in Hegi *≡* ***Soldanella hungarica*** Simonk. [s.str.et s.l.]

*Soldanella montana* var. *hungarica* (Simonk.) Grinţ. ≡ ***Soldanella hungarica*** Simonk. [s.str.et s.l.]

*Soldanella montana* var. *hungarica* f. *minor* (Schur) G.Kozij [nom. inval., ex herb. LWS] * – ***Soldanella hungarica*** Simonk. [s.str.] = ***Soldanella hungarica*** Simonk. [s.l.]

*Soldanella montana* var. *minor* (Schur) Borbás [p.p.] – ***Soldanella hungarica*** Simonk. [s.str.] = ***Soldanella hungarica*** Simonk. [s.l.]

*Soldanella montana* var. *repanda* Grinţ. = ***Soldanella marmarossiensis*** Klášt., emend. Zhang & Kadereit – ***Soldanella hungarica*** Simonk. [s.l.]

*Soldanella pseudomontana* F.K.Meyer = ***Soldanella hungarica*** Simonk. [s.str. et s.l.]

*Soldanella richteri* subsp. *marmarossiensis* (Klášt.) Niederle *≡* ***Soldanella marmarossiensis*** Klášt., emend. Zhang & Kadereit – ***Soldanella hungarica*** Simonk. [s.l.]

*Soldanella stiriaca* F.K.Meyer [nom. inval., superfl.] = ***Soldanella major*** (Neilr.) Vierh. in Urban & Graebn., emend. Zhang & Kadereit – ***Soldanella hungarica*** Simonk. [s.l.]

Swertia perennis L. [p. p. minor] – ***Swertia punctata*** Baumg.

*Swertia perennis* M.Bieb. ex Boiss., non L. = ***Swertia punctata*** Baumg.

*Swertia perennis* subsp. *punctata* (Baumg.) Ciocârlan, non *S.dichotoma* var. *punctata* T.N.Ho & J.X.Yang *≡* ***Swertia punctata*** Baumg.

***Swertia punctata*** Baumg.*

*Swertia stigmantha* K.Koch = ***Swertia punctata*** Baumg.

*Symphytum cordatum* M.Bieb. [nom. inval.] *–* ***Symphytum cordatum*** Waldst. & Kit. ex Willd., non M.Bieb.

***Symphytum cordatum*** Waldst. & Kit. ex Willd., non M.Bieb.*

*Symphytum cordatum* Waldst. & Kit., non M.Bieb. ≡ ***Symphytum cordatum*** Waldst. & Kit. ex Willd., non M.Bieb.

*Symphytum cordifolium* Baumg.* *=* ***Symphytum cordatum*** Waldst. & Kit. ex Willd., non M.Bieb.

*Symphytum pannonicum* Pers.* *=* ***Symphytum cordatum*** Waldst. & Kit. ex Willd., non M.Bieb.

*Syringa henryi* var. ex*imia* Rehder = ***Syringa josikaea*** J.Jacq. ex Rchb.f.

*Syringa josikaea* [unranked] *eximia* hort. ex Beissner, Schelle & Zabel = ***Syringa josikaea*** J.Jacq. ex Rchb.f.

*Syringa josikaea* [unranked] *pallida* hort. ex Beissner, Schelle & Zabel = ***Syringa josikaea*** J.Jacq. ex Rchb.f.

*Syringa josikaea* [unranked] *rubra* hort. ex Beissner, Schelle & Zabel = ***Syringa josikaea*** J.Jacq. ex Rchb.f.

*Syringa josikaea* [unranked] *zabeli* hort. ex Beissner, Schelle & Zabel = ***Syringa josikaea*** J.Jacq. ex Rchb.f.

*Syringa josikaea* f. *monstrosa* Jägger ex Morariu = ***Syringa josikaea*** J.Jacq. ex Rchb.f.

*Syringa josikaea* f. *pallida* Jägger ex Morariu = ***Syringa josikaea*** J.Jacq. ex Rchb.f.

*Syringa josikaea* f. *rosea* Miemetz ex Morariu = ***Syringa josikaea*** J.Jacq. ex Rchb.f.

*Syringa josikaea* f. *rubra* hort. ex Morariu = ***Syringa josikaea*** J.Jacq. ex Rchb.f.

*Syringa josikaea* f. *simia* Froebel ex Morariu = ***Syringa josikaea*** J.Jacq. ex Rchb.f.

*Syringa josikaea* f. *zabelii* Froebel ex Morariu = ***Syringa josikaea*** J.Jacq. ex Rchb.f.

***Syringa josikaea*** J.Jacq. ex Rchb.f. *

*Syringa josikaea* var. ex*imia* Froebel ex Olbrich = ***Syringa josikaea*** J.Jacq. ex Rchb.f.

*Syringa prunifolia* Kit. ex Lingelsh. = ***Syringa josikaea*** J.Jacq. ex Rchb.f.

*Syringa vincetoxifolia* Baumg. ex Steud. = ***Syringa josikaea*** J.Jacq. ex Rchb.f.

*Tanacetum rotundifolium* (Waldst. & Kit. in Willd.) Simonk.* *≡* ***Leucanthemum rotundifolium*** (Waldst. & Kit. in Willd.) DC., non Opiz

*Tanacetum waldsteinii* Sch.Bip. = ***Leucanthemum rotundifolium*** (Waldst. & Kit. in Willd.) DC., non Opiz

*Tanacetum waldsteinii* var. *ramosum* Ilse &.Fritze = ***Leucanthemum rotundifolium*** (Waldst. & Kit. in Willd.) DC., non Opiz

Thesium serratum Kit. ex D.Dietr., Syn. Pl. [D. Dietrich] 1: 878 (1839) ≡ ***Campanula serrata*** (Kit. ex Schult.) Hendrych

*Thesium serratum* Kit. ex Schult. ≡ ***Campanula serrata*** (Kit. ex Schult.) Hendrych

*Thlaspi alpestre* auct. [e.g., Schur, Fuss., Baumg.], non L. – ***Noccaea dacica*** subsp. ***dacica*** (Heuff.) F.K.Mey

*Thlaspi commutatum* Rochel, non Reiche = ***Noccaea dacica*** subsp. ***dacica*** (Heuff.) F.K.Mey

*Thlaspi corongianum* Czetz ex Nyman [ortho. var.] = ***Noccaea dacica*** subsp. ***dacica*** (Heuff.) F.K.Mey

*Thlaspi dacicum* [unranked] β *rodnense* Porcius = ***Noccaea dacica*** subsp. ***dacica*** (Heuff.) F.K.Mey

*Thlaspi dacicum* [unranked] β *transsilvanicum* Porcius = ***Noccaea dacica*** subsp. ***dacica*** (Heuff.) F.K.Mey

*Thlaspi dacicum* Heuff. * *≡* ***Noccaea dacica*** subsp. ***dacica*** (Heuff.) F.K.Mey

*Thlaspi korongianum* Czetz ex Nyman = ***Noccaea dacica*** subsp. ***dacica*** (Heuff.) F.K.Mey

*Thlaspi rotundifolium* auct. fl. transsilv., non Gaud – ***Noccaea dacica*** subsp. ***dacica*** (Heuff.) F.K.Mey

*Thlaspi trojagense* f. *abbreviatum* Zapał. = ***Noccaea dacica*** subsp. ***dacica*** (Heuff.) F.K.Mey

*Thlaspi trojagense* Zapał. = ***Noccaea dacica*** subsp. ***dacica*** (Heuff.) F.K.Mey

***Thymus alternans*** Klokov *

*Thymus carpathicus* auct fl. ucrain. carpat., non Čelak.* – ***Thymus pulcherrimus*** subsp. ***pulcherrimus*** Schur

*Thymus chamaedrys* subsp. *pulcherrimus* (Schur) Simonk. *≡* ***Thymus pulcherrimus*** subsp. ***pulcherrimus*** Schur

*Thymus circumcinctus* Klokov * = ***Thymus pulcherrimus*** subsp. ***pulcherrimus*** Schur

*Thymus glabrescens* auct., non Willd. – ***Thymus alternans*** Klokov

*Thymus marschallianus* auct., non Willd.* – ***Thymus alternans*** Klokov

*Thymus montanus* auct., non Waldst. & Kit. – ***Thymus pulcherrimus*** subsp. ***pulcherrimus*** Schur

*Thymus pulcherrimus* f. *beldiei* Guşul. in Săvul. [nom. invalid.] = ***Thymus pulcherrimus*** subsp. ***pulcherrimus*** Schur

*Thymus pulcherrimus* f. *oreades* (Lyka) Guşul. in Săvul. = ***Thymus pulcherrimus*** subsp. ***pulcherrimus*** Schur

*Thymus pulcherrimus* Schur [s. str.] * *≡* ***Thymus pulcherrimus*** subsp. ***pulcherrimus*** Schur

***Thymus pulcherrimus*** subsp. ***pulcherrimus*** Schur

*Thymus pulcherrimus* var. *oreades* (Lyka) Borza = ***Thymus pulcherrimus*** subsp. ***pulcherrimus*** Schur

*Thymus roegneri* K.Koch, [p.p., tantum quod plantas ucrain. carpat.] * – ***Thymus alternans*** Klokov

*Thymus rotundifolius* Schur, non alior = ***Thymus pulcherrimus*** subsp. ***pulcherrimus*** Schur

*Thymus serpyllum* f. *margittaianus* auct., non Lyka in Jáv. – ***Thymus alternans*** Klokov

*Thymus serpyllum* f. *oreades* Lyka ex Jáv. = ***Thymus pulcherrimus*** subsp. ***pulcherrimus*** Schur

*Thymus serpyllum* subsp. *pulcherrimus* (Schur) Lyka in Jáv. *≡* ***Thymus pulcherrimus*** subsp. ***pulcherrimus*** Schur

*Thymus serpyllum* var. *pulcherrimus* (Schur) Nyman *≡* ***Thymus pulcherrimus*** subsp. ***pulcherrimus*** Schur

Thymus serpyllum var. roegneri (K.Koch) Nyman [p. p., tantum quod plantas ucrain. carpat.] – ***Thymus alternans*** Klokov

*Thymus sudeticus* Opiz ex Borbás [p.p., tantum quod plantas ucrain. carpat.] * – ***Thymus pulcherrimus*** subsp. ***pulcherrimus*** Schur

*Thymus sudeticus* Opiz ex Rchb. [p.p., tantum quod plantas ucrain. carpat.] * – ***Thymus pulcherrimus*** subsp. ***pulcherrimus*** Schur

*Trifolium banaticum* (Heuff.) Májovský = ***Trifolium sarosiense*** Hazsl.

*Trifolium flexuosum* subsp. *sarosiense* (Hazsl.) Gibelli & Belli *≡* ***Trifolium sarosiense*** Hazsl.

*Trifolium medium* [unranked] e *humile* Schur = ***Trifolium sarosiense*** Hazsl.

*Trifolium medium* subsp. *banaticum* (Heuff.) Hendrych = ***Trifolium sarosiense*** Hazsl.

*Trifolium medium* subsp. *sarosiense* (Hazsl.) Simonk.* *≡* ***Trifolium sarosiense*** Hazsl.

*Trifolium medium* var. *banaticum* Heuff. = ***Trifolium sarosiense*** Hazsl.

*Trifolium medium* var. *sarosiense* (Hazsl.) A.Nyár. in Săvul. ≡ ***Trifolium sarosiense*** Hazsl.

*Trifolium medium* var. *sarosiense* f. *bracteolatum* A.Nyár. in Săvul. = ***Trifolium sarosiense*** Hazsl.

*Trifolium medium* var. *sarosiense* f. *eciliatum* A.Nyár. in Săvul. = ***Trifolium sarosiense*** Hazsl.

***Trifolium sarosiense*** Hazsl.

*Trifolium sarosiense* Hazsl. ex Neilr. ≡ ***Trifolium sarosiense*** Hazsl.

*Trifolium sarosiense* subsp. *banaticum* (Heuff.) Holub = ***Trifolium sarosiense*** Hazsl.

*Trisetaria carpatica* auct. fl. carpat., non (Host) Baumg – ***Trisetum fuscum*** (Kit. ex Schult.) Schult. in Roem. & Schult.

*Trisetaria fusca* (Kit. ex Schult.) Banfi & Soldano *≡* ***Trisetum fuscum*** (Kit. ex Schult.) Schult. in Roem. & Schult.

*Trisetum carpathicum* auct., non (Host) Roem. & Schult.* – ***Trisetum fuscum*** (Kit. ex Schult.) Schult. in Roem. & Schult.

*Trisetum ciliare* (Kit. ex Schult.) Domin * = ***Trisetum fuscum*** (Kit. ex Schult.) Schult. in Roem. & Schult.

*Trisetum flavescens* [unranked] c *carpaticum* f. *majus* Zapał., non Asch. & Graebn. = ***Trisetum fuscum*** (Kit. ex Schult.) Schult. in Roem. & Schult.

*Trisetum flavescens* subsp. *fuscum* (Kit. ex Schult.) Hack. ≡ ***Trisetum fuscum*** (Kit. ex Schult.) Schult. in Roem. & Schult.

***Trisetum fuscum*** (Kit. ex Schult.) Schult. in Roem. & Schult.*

*Trisetum tenue* Baumg. ex Steud. [nom. illeg., pro syn. T. transylvanicum Steud.], non Leers – ***Trisetum fuscum*** (Kit. ex Schult.) Schult. in Roem. & Schult.

*Trisetum transylvanicum* Steud., non Schur = ***Trisetum fuscum*** (Kit. ex Schult.) Schult. in Roem. & Schult.

*Trisetum varium* var. *violaceum* Schur = ***Trisetum fuscum*** (Kit. ex Schult.) Schult. in Roem. & Schult.

*Tryphane gerardi* auct. fl. carpat., non (Willd.) Rchb. – ***Sabulina pauciflora*** (Kit.) A.Novikov, *comb. nov.*

*Viola declinata* [unranked] b *montana* Schur = ***Viola declinata*** Waldst. & Kit.

*Viola declinata* var. *knechtelii* Grec. = ***Viola declinata*** Waldst. & Kit.

*Viola declinata* var. *major* (Roch.) Grec. = ***Viola declinata*** Waldst. & Kit.

***Viola declinata*** Waldst. & Kit.*

*Viola gracilis* Rchb., non alior = ***Viola declinata*** Waldst. & Kit.

*Viola mutabilis* [unranked] b *intermedia* Roch. [nom. nudum] = ***Viola declinata*** Waldst. & Kit.

*Viola mutabilis* [unranked] e *major* Roch. [nom. nudum] = ***Viola declinata*** Waldst. & Kit.
