# Supplementary material for: An annotated nomenclatural checklist of endemic vascular plants distributed in the Ukrainian Carpathians
Source: Biodivers Data J. 2023 Aug 11;11:e103921. doi: 10.3897/BDJ.11.e103921 (PMC10848708; doi:10.3897/BDJ.11.e103921)
Supplement: Supplementary material 2 — Clustered synonymic checklist of endemic species and infraspecific of vascular plants taxa distributed in the Ukrainian Carpathians [file bdj-11-e103921-s002.docx]

**Supplement B. Clustered synonymic checklist of endemic species and infraspecific taxa of vascular plants distributed in the Ukrainian Carpathians**

***Scilla kladnii* Schur, Enum. Pl. Transsilv.: 668 (1866)**

≡ Scilla bifolia var. kladnii (Schur) Nyman, Consp. Fl. Eur.: 730 (1882)

= Scilla alpina Schur, Verh. Mitth. Siebenbürg. Vereins Naturwiss. Hermannstadt 3: 90 (1852)

≡ Scilla bifolia subsp. alpina (Schur) Nyman, Consp. Fl. Eur.: 730 (1882)

≡ Scilla bifolia var. alpina (Schur) C.Zahariadi in Nyár., Fl. Rep. Pop. Rom. 11: 314 (1966)

= Scilla bifolia β [unranked] gracillima Grecescu, Consp. Fl. Rom.: 565 (1898), non alior

= Scilla subtriphylla Schur, Enum. Pl. Transsilv.: 668 (1866) *

≡ Scilla bifolia subsp. subtriphylla (Schur) Domin, Preslia 13–15: 19 (1936)

≡ Scilla bifolia var. subtriphylla (Schur) T.Simon, Ann. Biol. Univ. Debrecen. n.s., 1: 154 (1950)

= Scilla trifolia Schur, Enum. Pl. Transsilv.: 668 (1866)

– Scilla bifolia L., Sp. Pl. 1: 309 (1753) [p. p., tantum quod plantas ucrain. carpat.], non alior *

– Scilla bifolia var. nivalis auct. fl. carpat, non Baker

– Scilla bifolia subsp. nivalis (Boiss.) K.Richt., Pl. Eur., 1: 220 (1890) sensu Fodor [non sensu orig.]

– Scilla praecox auct. fl. carpat, non Willd.

# Crocus banaticus J.Gay, Bull. Sci. Nat. Géol. (Bull. Férussac), 25: 220, Nr. 178 (1831), non Heuff.

= Crocus herbertianus Körn., Index Seminum (B, Berolinensis) 1854 (App.): 15 (1855)

= Crocus iridiflorus Heuff. ex Rchb., Ic. Fl. Germ. 9: 10, figs. 802, 803 (1847)

≡ *Crociris iridiflora* (Heuff. ex Rchb.) Schur, Verh. Mitth. Siebenbürg. Vereins Naturwiss. Hermannstadt 4: 73 (1853)

= Crocus nudiflorus Schult., Oestr. Fl., ed. 2, 1: 101 (1814) [nom. illeg.], non alior

= Crocus speciosus Baumg., Enum. Stirp. Transsilv. 1: 60 (1816), non alior

= Crocus speciosus (Baumg.) Host, Fl. Austr. 1: 43 (1827), non alior [nom. illeg.]

≡ Crociris speciosa (Host) Schur, Verh. Mitth. Siebenbürg. Vereins Naturwiss. Hermannstadt 4: 73 (1853)

= Crocus speciosus var. transsylvanicus Hooker, Curt. Bot. Mag. 67: t. 3861 (1840–1841)

– Crocus byzantinus Ker Gawl., Bot. Mag. 28: t. 1111 (1808) [p. p.]

# Gymnadenia carpatica (Zapał.) Teppner et E.Klein, Phyton (Horn) 38 (1): 221 (1998)

≡ Nigritella angustifolia var. carpatica Zapał., Consp. Fl. Gallic. Crit. 1: 215 (1906)

≡ Nigritella rubra f. carpatica (Zapał.) Soó, Repert. Sp. Nov. Regni Veg. 24: 33 (1927)

≡ Nigritella nigra var. carpatica (Zapał.) Pawł., Bull. Int. Acad. Polon. Sci., Cl. Sci. Math., Sér. B 1, Bot. 1947: 85, 96 (1947)

≡ Nigritella carpatica (Zapał.) Teppner, E.Klein & Zag., Phyton (Horn) 34: 171 (1994) *

≡ Nigritella nigra subsp. carpatica (Zapał.) H.Baumann & R.Lorenz, J. Eur. Orch. 37: 717 (2005)

– Gymnadenia nigra auct. fl. ucrain. carpat., non (L.) Rchb.f.

– Nigritella nigra auct. fl. ucrain. carpat., non (L.) Rchb.f. *

### **Luzula alpinopilosa subsp. obscura S.E.Fröhner, Preslia 40: 426 (1968)**

≡ Luzula obscura (S.E.Fröhner) Novikov, Byull. Moskovsk. Obshch. Isp. Prir., Otd. Biol. 95(6): 66 (1990)

= Luzula carpatica Kitt. ex Kanitz, Linnaea 32: 327 (1863)

≡ Luzula spadicea var. carpatica (Kitt. ex Kanitz) Nyman, Consp. Fl. Eur., Suppl. 2: 314 (1890)

≡ Luzula spadicea [unranked] carpatica (Kitt. ex Kanitz) Asch. & Graebn., Syn. Mitteleur. Fl. 2(2): 513 (1904)

≡ Luzula spadicea f. carpatica (Kitt. ex Kanitz) I.Grinț., Fl. Rep. Soc. Rom. 11: 594 (1966)

= Juncus spadiceus [unranked] β glabratus Wahlbg., Fl. Carp. Princip.: 102 (1814), non Hoppe nec Host

– Juncus alpinopilosus Chaix, Hist. Pl. Dauphiné (Villars) 1: 318 (1786) [p. p., tantum quod plantas carpat.]

– Juncus spadiceus All., Fl. Pedem. 2: 216 (1785) [nom. invalid., p. p., tantum quod plantas carpat.], non alior

– Luzula alpinopilosa (Chaix) Breistr., Bull. Soc. Sci. Dauph. 61: 609 (1947) [p. p., tantum quod plantas carpat.] *

– Luzula spadicea (All.) Lam. & DC., Fl. Franc. [de Candolle & Lamarck], ed. 3. 3: 159 (1805) [p. p., tantum quod plantas carpat.] *

# Alopecurus pratensis subsp. laguriformis (Schur) Tzvelev, Novosti Sist. Vyssh. Rast. 8: 19 (1971)

≡ Alopecurus laguriformis Schur, Verh. Siebenb. Ver. Naturw. 1: (1850) 182 [nom. nudum] et Schur ex Gris., Iter. Hung: 362 (1852) *

= Alopecurus laguriformis [unranked] a abbreviatus Schur, Oesterr. Bot. Z. 9: 13 (1859)

= Alopecurus laguriformis [unranked] b elongatus Schur, Oesterr. Bot. Z. 9: 13 (1859) et Enum. Pl. Transsilv.: 727 (1866)

= Alopecurus transsilvanicus Schur, Enum. Pl. Transsilv.: 727 (1866)

– Alopecurus brachystachyus auct. [e.g., Janka], non M.Bieb.

– Colobachne gerardi Schur, Enum. Pl. Transsilv.: 728 (1866), non Link.

# Festuca amethystina subsp. orientalis Krajina, Acta Bot. Bohem. 9: 214 (1930), non alior

= Festuca amethystina [unranked] a marmarossica Zapał., Consp. Fl. Galic. Crit. 1: 65 (1906) [ortho. var.] *

≡ Festuca amethystina [unranked] a marmarossiensis Zapał., Consp. Fl. Galic. Crit. 3: 230 (1911)

≡ Festuca amethystina subsp. amethystina var. amethystina f. marmarossica (Zapał.) Beldie, Fl. Rep. Pop. Soc. Rom. 12: 557 (1972)

= Festuca amethystina [unranked] a marmarossiensis f. doamnensis Zapał., Consp. Fl. Galic. Crit. 3: 230 (1911)

= Festuca amethystina f. pauciflora A.Nyár. & Nyár., Studii Cercet. Biol. Ser. Bot. 16(2): 109 (1964)

≡ Festuca amethystina subsp. amethystina var. amethystina f. pauciflora (A.Nyár. & Nyár.) Beldie, Fl. Rep. Soc. Rom. 12: 557 (1972)

= Festuca heterophylla var. setifolia Schur ex Schur, Enum. Pl. Transsilv.: 792 (1866)

= Festuca inarmata Schur, Verh. Mitth. Siebenbürg. Vereins Naturwiss. Hermannstadt 10: 177 (1859) *

≡ Festuca heterophylla var. inarmata Schur ex Schur, Enum. Pl. Transsilv.: 792 (1866)

≡ Festuca amethystina subsp. inarmata (Schur) Krajina, Veröff. Geobot. Inst. Rübel Zürich 10: 29 (1933)

– Festuca amethystina L., Sp. Pl. 1: 74 (1753) [p. p., tantum quod plantas ucrain. carpat.] *

# Festuca carpatica F.Dietr., Nachtr. Vollst. Lex. Gärtn. 3: 333 (1817)

≡ Amphigenes carpathica (F.Dietr.) Janka, Linnaea 30(5): 619 (1859) & Janka ex Hack., Monogr. Fest. Eur.: 187 (1882)

≡ Leucopoa carpatica (F.Dietr.) H.Scholz, Willdenowia 35: 242 (2005)

= Festuca carpatica var. carpatica f. umbrosa Beldie, Fl. şi veg. Bucegi: 329 (1967)

= Festuca carpatica f. elatior Krajina, Rozpr. Wydz. Mat.-Przyr. Akad. Umiejetn., Dzial B, Nauki Biol. 9: 219 (1930)

= Festuca carpatica f. subflavescens Zapał., Bull. Int. Acad. Sci. Cracovie, Cl. Sci. Math. 4B: 184. (1904)

≡ Festuca carpatica var. carpatica f. subflavescens (Zapał.) Beldie, Fl. Rep. Soc. Rom. 12: 484 (1972)

= Festuca dimorpha Janka, Oesterr. Bot. Z. 16: 101 (1866), non Guss.

= Festuca laxa Schur, Verh. Mitth. Siebenbürg. Vereins Naturwiss. Hermannstadt 10: 177 (1859), non Host

= Festuca nutans Wahlenb., Fl. Carpat. Princ.: 28 (1814), non Host nec Moench

≡ Amphigenes nutans (Wahlenb.) Janka, Linnaea 30(5): 619 (1859)

= Festuca pseudolaxa Schur, Oesterr. Bot. Z. 8: 22 (1858)

≡ Festuca carpatica f. pseudolaxa (Schur) Jáv., Magyar Bot. Lapok 10: 266 (1911)

= Festuca pseudonutans Schur, Enum. Pl. Transsilv.: 796 (1866)

= Festuca pulchella subsp. scheuchzeriformis var. bucegica Krajina, Veröff. Geobot. Inst. ETH Stiftung Rübel Zürich 10: 52 (1933)

≡ Festuca carpatica var. bucegica (Krajina) Beldie, Fl. şi veg. Bucegi: 330 (1967)

= Festuca scheuchzeriformis Schur, Enum. Pl. Transsilv.: 796 (1866)

# Festuca porcii Hack., Bot. Centralbl. 2(8): 407 (1881)

= Festuca porcii var. hirsuta A.Nyár., Not. Bot. Cluj 2: 83 (1966)

≡ Festuca porcii f. hirsuta (A.Nyár.) Beldie, Fl. Rep. Soc. Rom. 12: 524 (1972)

= Festuca porcii f. longiaristata Krajina, Veröff. Geobot. Inst. ETH Stiftung Rübel Zürich 10: 32 (1933)

= Festuca porcii var. vestita Hack. ex Zapał., Consp. Fl. Galic. Crit. 1: 67 (1906)

≡ Festuca porcii f. vestita (Hack.) Krajina, Veröff. Geobot. Inst. ETH Stiftung Rübel Zürich 10: 31 (1933)

# Festuca versicolor subsp. versicolor Tausch, Flora 4(2): 559 (1821) et Tausch ex Kraj., Publ. Fac. Sc. Univ. Charles, Prague 106: 25 (1930), non J.Presl ex Kunth

= Festuca minor Schur, Enum. Pl. Transsilv.: 795 (1866), non St.-Lag.

≡ Festuca versicolor var. minor (Schur) Krajina, Veröff. Geobot. Inst. ETH Stiftung Rübel Zürich 10: 40 (1933)

= Festuca varia f. acuminata Sagorski & Schneider, Fl. Centralkarp. 2: 554 (1891), non (Gaudin) Bolzon

= Festuca varia [unranked] giewontica Zapał., Consp. Fl. Galic. Crit. 1: 71 (1906)

≡ Festuca versicolor subsp. eu-versicolor var. genuina subvar. vulgaris f. giewontica (Zapał.) Krajina, Spisy Přír. Fak. Karlovy Univ. 106: 33 (1930)

= Festuca varia [unranked] flavescens Zapał., Consp. Fl. Galic. Crit. 1: 70 (1906), non Gaudin

= Festuca varia subsp. pumila [unranked] spiculis flavescentibus Gaudin ex Hack. in Sagorski & Schneider, Fl. Centralkarp. 2: 554 (1891)

= Festuca varia var. scopariaeformis Kotula, Rozmieszczenie roślin naczyniowych w Tatrach: 456 (1890)

≡ Festuca versicolor subsp. eu-versicolor var. genuina subvar. vulgaris f. scopariaeformis (Kotula) Krajina, Spisy Přír. Fak. Karlovy Univ. 106: 32 (1930)

= Festuca versicolor var. versicolor f. chrysantha (Krajina) Beldie, Fl. Rep. Soc. Rom. 12: 491 (1972)

= Festuca versicolor var. versicolor f. debilis (Krajina) Beldie, Fl. Rep. Soc. Rom. 12: 491 (1972)

= Festuca versicolor subsp. eu-versicolor var. genuina subvar. rodnensis Krajina, Spisy Přír. Fak. Karlovy Univ. 106: 37 (1930)

= Festuca versicolor subsp. eu-versicolor var. genuina subvar. rodnensis f. minor Krajina, Spisy Přír. Fak. Karlovy Univ. 106: 38 (1930)

= Festuca versicolor subsp. eu-versicolor var. genuina subvar. rodnensis f. typica Krajina, Spisy Přír. Fak. Karlovy Univ. 106: 38 (1930)

= Festuca versicolor subsp. eu-versicolor var. genuina subvar. transsilvanica Krajina, Spisy Přír. Fak. Karlovy Univ. 106: 38 (1930)

= Festuca versicolor subsp. eu-versicolor var. genuina subvar. transsilvanica f. Kotschyi Krajina, Spisy Přír. Fak. Karlovy Univ. 106: 39 (1930)

= Festuca versicolor subsp. eu-versicolor var. genuina subvar. transsilvanica f. pallens Krajina, Spisy Přír. Fak. Karlovy Univ. 106: 39 (1930)

= Festuca versicolor subsp. eu-versicolor var. genuina subvar. transsilvanica f. typica Krajina, Spisy Přír. Fak. Karlovy Univ. 106: 39 (1930)

= Festuca versicolor subsp. eu-versicolor var. genuina subvar. vulgaris Krajina, Spisy Přír. Fak. Karlovy Univ. 106: 31 (1930)

= Festuca versicolor subsp. eu-versicolor var. genuina subvar. vulgaris f. chrysantha Krajina, Spisy Přír. Fak. Karlovy Univ. 106: 32 (1930)

= Festuca versicolor subsp. eu-versicolor var. genuina subvar. vulgaris f. curvala Krajina, Spisy Přír. Fak. Karlovy Univ. 106: 32 (1930)

= Festuca versicolor subsp. eu-versicolor var. genuina subvar. vulgaris f. debilis Krajina, Spisy Přír. Fak. Karlovy Univ. 106: 33 (1930)

= Festuca versicolor subsp. eu-versicolor var. genuina subvar. vulgaris f. glaucophylla Krajina, Spisy Přír. Fak. Karlovy Univ.: 33 (1930)

= Festuca versicolor subsp. eu-versicolor var. genuina subvar. vulgaris f. mutica Krajina, Spisy Přír. Fak. Karlovy Univ. 106: 33 (1930)

= Festuca versicolor subsp. eu-versicolor var. genuina subvar. vulgaris f. robustior Krajina, Spisy Přír. Fak. Karlovy Univ. 106: 32 (1930)

= Festuca versicolor subsp. eu-versicolor var. genuina subvar. vulgaris f. typica Krajina, Spisy Přír. Fak. Karlovy Univ. 106: 32 (1930)

= Festuca versicolor subsp. eu-versicolor var. genuina subvar. vulgaris f. zapalowiczii Krajina, Spisy Přír. Fak. Karlovy Univ. 106: 33 (1930)

= Festuca versicolor subsp. pseudosulcata Krajina, Spisy Přír. Fak. Karlovy Univ. 106: 43 (1930), non Drobow

– Festuca varia f. pallidula auct., non Hack.

# Koeleria transsilvanica Schur, Oesterr. Bot. Wochenbl. 7: 313 (1857), non Barth.

≡ Koeleria cristata [unranked] d) transsilvanica (Schur) K.Richt., Pl. Eur. 1: 75 (1890)

≡ Koeleria cristata subsp. ciliata var. transsilvanica (Schur) Asch. & Graebn., Syn. Mitteleur. Fl. 2(1): 358 (1900)

≡ Koeleria gracilis subsp. transsilvanica (Schur) Domin, Monographie d. Gattung Koeleria, Biblioth. Bot. 14(65): 239 (1907) et Flora Romaniae Exsiceatae

≡ Koeleria gracilis var. transsilvanica (Schur) Jáv., Magyar Fl.: 87 (1925)

≡ Koeleria macrantha subsp. transsilvanica (Schur) A.Nyár. (1965) [nom. nudum ?]

≡ Koeleria macrantha subsp. transsilvanica (Schur) Beldie, Fl. Rom. Det.: 342 (1977) [nom. illeg.]

= Koeleria gracilis var. rohlenae Domin, Biblioth. Bot. 65: 193 (1907)

= Koeleria gracilis var. typica Domin, Biblioth. Bot. 65: 230 (1907)

= Koeleria transsilvanica var. tenuipes f. discolor Degen ex Domin, Magyar. Bot. Lap. 3: 259 (1904) et Bibl. Bot. 14: 240 (1907)

= Koeleria transsilvanica [unranked] b tenuipes alpestris Schur, Oesterr. Bot. Wochenbl. 7: 313 (1857)

≡ Koeleria transsilvanica [unranked] a tenuipes alpestris Schur, Enum. Pl. Transsilv.: 750 (1866)

≡ Koeleria transsilvanica var. tenuipes (Schur) Domin, Magyar Bot. Lapok 3: 259 (1904)

≡ Koeleria tenuipes (Schur) Ujhelyi, Ann. Hist.-Nat. Mus. Natl. Hung. 57: 191 (1965)

≡ Koeleria transsilvanica subsp. tenuipes (Schur) Soó, Acta Bot. Acad. Sci. Hung. 17(1–2): 122 (1972)

– Koeleria gracilis Pers, Syn. Pl. [Persoon] 1: 97 (1805) [p. p., ex herb.], non Guss.

– Koeleria cristata var. colorata Heuff., Verh. K.K. Zool.-Bot. Ges. Wien 8: 228 (1858) [p. p.]

≡ Koeleria gracilis var. colorata (Heuff.) Domin, Magyar Bot. Lapok 3: 268 (1904) [p. p.], non alior

≡ Koeleria gracilis f. colorata (Heuff.) Domin, Biblioth. Bot. 65: 232 (1907) [p. p.]

≡ Koeleria colorata (Heuff.) Nyár. ex Degen, Gramina Hungarica: nr 368 (1911) [p. p.]

≡ *Koeleria macrantha* var. *colorata* (Heuff.) Ghisa, Fl. Rep. Soc. Rom. 12: 237 (1972) [p. p.]

– Koeleria cristata var. glabra Kotschy [ex herb., nom. nudum], non alior.

– Koeleria cristata [unranked] foliis vaginisque glabris Andrä [ex herb., nom. nudum]

– Koeleria setacea DC. sensu Nyman, Consp. Fl. Eur.: 816 (1878–1882)

# Poa carpatica subsp. carpatica (V.Jirásek) Bernátová, Májovský, Kliment & Topercer, Biologia (Bratislava), Sect. Bot. 61(4): 389-390 (2006)

≡ Poa nemoralis subsp. carpatica V.Jirásek, Veda Prir. 15: 207 (1934) *

≡ Poa carpatica (V.Jirásek) Chopik, Visokogirna Fl. Ukrains’k. Karpat: 174 (1976)

≡ Poa nemoralis subsp. nemoralis var. carpatica (V.Jirásek) Soó, Acta Bot. Acad. Sci. Hung. 17(1–2): 118 (1972)

= Poa balfourii f. carpatica Zapał., Spraw. Komis. Fizjogr. 39: 33 (1906)

= Poa nemoralis subsp. carpatica f. minoriformis V.Jirásek, Věda Přír. 15: 208 (1934)

– Poa balfourii auct. fl. ucrain. carpat., non Parn. *

– Poa janczewskii auct., non Zapał. [tantum quod plantas ucrain. carpat., alp. et subalp. altitud. solum]

– Poa nemoralis subsp. montana auct., non (Gaudin) Chrtek & V.Jirásek

– Poa nemoralis var. montana auct. fl. ucrain. carpat., non Gaudin *

# Poa granitica subsp. disparillis (Nyár.) Nyár., Rev. Roumaine Biol., Sér. Bot. 10: 355 (1965)

≡ Poa granitica var. disparillis Nyár., Veröff. Geobot. Inst. Rübel Zürich 10: 173 (1933)

≡ Poa cenisia subsp. granitica var. disparillis (Nyár.) Nyár. & Borza, Consp. Fl. Roman. 1: 16 (1947)

= Poa breazensis Nyár., Veröff. Geobot. Inst. Rübel Zürich 10: 173 (1933)

≡ Poa deylii var. deylii f. breazensis (Nyár.) Ghișa & Beldie, Fl. Rep. Soc. Rom. 12: 399 (1972)

= Poa cenisia [unranked] b pietrosuana Zapał., Consp. Fl. Gallic. Crit. 6: 227 (1911) *

≡ Poa deylii var. deylii f. pietrosuana (Zapał.) Ghișa & Beldie, Fl. Rep. Soc. Rom. 12: 399 (1972)

= Poa deylii Chrtek & V.Jirásek, Feddes Repert. Spec. Nov. Regni Veg. 69: 177 (1964) *

= Poa granitica subsp. retezatensis A.Nyár., Rev. Roumaine Biol., Sér. Bot. 10: 356 (1965)

≡ Poa deylii subsp. retezatensis (A.Nyár.) Chrtek, Oesterr. Bot. Z., 115 (4–5): 424 (1968)

≡ Poa deylii var. retezatensis (A.Nyár.) Ghișa & Beldie, Fl. Rep. Soc. Rom. 12: 399 (1972)

= Poa granitica var. disparillis f. pietrosuana (Zapał.) Nyár., Veröff. Geobot. Inst. Rübel Zürich 10: 173 (1933)

= Poa granitica subsp. disparillis var. subgranitica Nyár., Rev. Roumaine Biol., Sér. Bot. 10: 355 (1965)

≡ Poa deylii var. deylii f. subgranitica (Nyár.) Ghișa & Beldie, Fl. Rep. Soc. Rom. 12: 399 (1972)

= Poa granitica var. subcarpatica V.Jirásek, Vest. Král. Ceské Spol. Náuk 1935: 11 (1936)

≡ Poa granitica subsp. subcarpatica (V.Jirásek) Fodor, Flora Zakarpattia: 182 (1974)

= Poa granitica var. typica Nyár., Veröff. Geobot. Inst. Rübel Zürich 10: 171–172 (1933)

= Poa granitica var. typica f. deminuta Nyár., Veröff. Geobot. Inst. Rübel Zürich 10: 172 (1933)

– Poa cenisia All., Auct. Fl. Pedem.: 40 (1789) [p. p., tantum quod plantas ucrain. carpat.]

– Poa granitica Braun-Blanq., Arch. Bot., Caen, Bull. 3: 46 (1929) [p. p., tantum quod plantas ucrain. carpat.] *

– Poa granitica subsp. granitica Braun-Blanq., Arch. Bot., Caen, Bull. 3: 46 (1929) sensu Tasenkevich [non sensu orig, ex herb. LWS] *

# Poa pannonica subsp. scabra (Asch.) Soó, Acta Bot. Acad. Sci. Hung. 5: 483 (1959)

≡ Poa scabra Kit. ex Steud., Nomencl. Bot. [Steudel], ed. 2. 2: 362 et Linnaea, 32: 311 (1863) [nom. nudum], non Ehrh. *

≡ Poa scabra Asch., Verh. K.K. Zool.-Bot. Ges. Wien 17: 568 (1867), non Ehrh.

≡ Poa pratensis var. scabra (Asch.) Asch. & Graebn., Syn. Mitteleur. Fl. 2(1): 414 (1900)

≡ Poa sterilis subsp. eu-sterilis var. scabra (Asch.) Asch. & Graebn., Syn. Mitteleur. Fl. 2(1): 414 (1900), non alior

= Poa perscabra Holub, Folia Geobot. Phytotax. 18(2): 204 (1983)

= Poa sterilis Kerner, Oesterr. Bot. Z. 14: 85 (1864), non M.Bieb.

# Poa rehmannii (Asch. et Graebn.) Woł., Fl. Polon. Exs., 10–11: Nr 1020 (1904)

≡ Poa caesia [unranked] d) rehmanni K.Richt., Pl. Eur. 1: 83 (1890)

≡ Poa nemoralis subsp. rehmannii Asch. & Graebn., Syn. Mitteleur. Fl. 2(1): 412 (1900)

≡ Poa rehmannii (Asch. & Graebn.) K.Richt., Pl. Eur. 1: 83 (1890) [nom. nudum]

= Poa anceps Rehmann, Spraw. Komis. Fizjogr. 7: 5 (1873), non G.Forst.

– Poa rehmannii Asch. & Gürke sensu Woł. [nom. confus., ex herb. LWS]

# Sesleria bielzii Schur, Verh. Mitth. Siebenbürg. Vereins Naturwiss. Hermannstadt 1: 109 (1850) et Verh. Mitth. Siebenbürg. Vereins Naturwiss. Hermannstadt 4: 84 (1853)

≡ Sesleria rigida [unranked] β bielzii (Schur) Heuff., Enum. Pl. Banat.: 227 (1858)

≡ Sesleria coerulans subsp. bielzii (Schur) Gergely & Beldie, Fl. Rep. Soc. Rom. 12: 223 (1972) *

= Sesleria caerulea Janka, Linnaea 30: 615 (1859), non Ard.

= Sesleria coerulans var. borsae Deyl, Opera Bot. Čechina 3: 139 (1946)

= Sesleria haynaldiana [unanked] g pseudorigida Schur, Verh. K.K. Zool.-Bot. Ges. Wien 6: 209 (1856)

≡ Sesleria pseudorigida Schur, Enum. Pl. Transsilv.: 745 (1866)

≡ Sesleria coerulans f. pseudorigida (Schur) Beldie, Bul. Şt. Acad. R.P.R. 2(5): 248 (1950)

= Sesleria rigida Griseb., Arch. Naturgesch. (Berlin) 18(1): 361 (1852), non Heuff. ex Rchb.

= Sesleria rigida [unranked] a capitata Schur, Verh. K.K. Zool.-Bot. Ges. Wien 6: 201 (1856)

≡ Sesleria capitata (Schur) Schur, Enum. Pl. Transsilv.: 743 (1866)

= Sesleria rigida [unranked] b ovoidea Schur, Verh. K.K. Zool.-Bot. Ges. Wien 6: 201 (1856)

– Sesleria caerulea Scap. sensu Rehman [nom. confus. ex herb. LWS] *

– Sesleria coerulans Friv., Flora 19(2): 438 (1836) [p. p., tantum quod plantas ucrain. carpat.] *

# Sesleria heufleriana subsp. heufleriana Schur, Verh. Mitth. Siebenbürg. Vereins Naturwiss. Hermannstadt 4: 84 (1853) et Verh. Zool.-Bot. Ges. Wien 6: 203 (1856)

≡ Sesleria heufleriana Schur, Verh. Mitth. Siebenbürg. Vereins Naturwiss. Hermannstadt 4: 84 (1853) et Verh. Zool.-Bot. Ges. Wien 6: 203 (1856)

≡ Sesleria heufleriana Schur ex Błocki, Oesterr. Bot. Z. 39: 155 (1889) [nom. inval.] *

= Sesleria caerulea [unranked] a interrupta Schur, Enum. Pl. Transsilv.: 743 (1866)

≡ Sesleria heufleriana f. interrupta (Schur) Soó, Acta Bot. Acad. Sci. Hung. 17(1–2): 119 (1972)

= Sesleria caerulea [unranked] b prorepens Schur, Enum. Pl. Transsilv.: 743 (1866)

≡ Sesleria heufleriana f. prorepens (Schur) Soó, Acta Bot. Acad. Sci. Hung. 17(1–2): 119 (1972)

≡ Sesleria prorepens Schur ex Schur, Enum. Pl. Transsilv.: 743 (1866)

= Sesleria caerulea [unranked] c praelonga Schur, Enum. Pl. Transsilv.: 743 (1866)

= Sesleria caerulea var. transilvanica (Schur) Jáv., Magyar Fl. 1: 84 (1924)

= Sesleria heufleriana [unranked] a praelonga Schur, Enum. Pl. Transsilv.: 744 (1866)

≡ Sesleria heufleriana f. praelonga (Schur) Gergely & Beldie, Fl. Rep. Soc. Rom. 12: 224 (1972)

= Sesleria heufleriana [unranked] b digitata Schur, Verh. Zool.-Bot. Ges. Wien 6: 204 (1856) et Enum. Pl. Transsilv.: 744 (1866)

= Sesleria heufleriana [unranked] c elongata Schur, Verh. Zool. -Bot. Ges. Wien 6: 204 (1856), non Host

= Sesleria heufleriana var. insignis Schur, Verh. Mitth. Siebenbürg. Vereins Naturwiss. Hermannstadt 4: 84 (1853)

= Sesleria heufleriana [unranked] a polydactyla Schur, Verh. Zool.-Bot. Ges. Wien 6: 204 (1856)

= Sesleria nitida Heldr. ex Nyman, Consp. Fl. Eur. 4: 796 (1882) [nom. illeg.], non Ten.

= Sesleria robusta Pávai, Oesterr. Bot. Z. 12: 214 (1862) [nom. nudum], non Schott et al.

= Sesleria transilvanica Schur, Verh. Zool.-Bot. Vereins Wien 6: 205 (1856) et Enum. Pl. Transsilv.: 745 (1866)

– Sesleria caerulea Baumg., Enum. Stirp. Transsilv. 3: 228, Nr 2013 (1816) [p. p.], non (L.) Ard.

# Trisetum fuscum (Kit. ex Schult.) Schult. in Roem. et Schult., Syst. Veg. 2: 664 (1817)

≡ Avena fusca Kit. ex Schult., Oesterr. Fl. ed. 2, 1: 268 (1814), non Ard.

≡ Trisetum flavescens subsp. fuscum (Kit. ex Schult.) Hack., Magyar Bot. Lap. 2: 111 (1903)

≡ Trisetaria fusca (Kit. ex Schult.) Banfi & Soldano, Atti Soc. Ital. Sci. Nat. Mus. Civico Storia Nat. Milano 135(2): 383 (1996)

= Avena ciliaris Kit. ex Schult., Oesterr. Fl. ed. 2, 1: 268 (1814)

≡ Trisetum ciliare (Kit. ex Schult.) Domin, Preslia 13-15: 41 (1935) *

= Trisetum flavescens [unranked] c carpaticum f. majus Zapał., Rozpr. Wydz. Mat.-Przyr. Akad. Umiejetn., Dzial B, Nauki Biol. 4: 108 (1904) et Consp. Fl. Galic. Crit. 1: 35 (1906), non Asch. & Graebn.

= Trisetum transylvanicum Steud., Syn. Pl. Glumac. 1(3): 226 (1855), non Schur

= Trisetum varium var. violaceum Schur, Oesterr. Bot. Z. 10: 75 (1860)

– Avena carpatica auct. [e.g., Błocki ex herb.], non Host

– Trisetaria carpatica auct. fl. carpat., non (Host) Baumg

– Trisetum carpathicum auct., non (Host) Roem. & Schult. *

– Trisetum tenue Baumg. ex Steud., Syn. Pl. Glumac. 1(3): 226 (1854) [nom. illeg., pro syn. T. transylvanicum Steud.], non Leers

# Heracleum carpaticum Porcius, Magyar Növénytani Lapok 2: 25 (1878) et Fl. Naséud.: 144 (1881)

≡ Heracleum sphondylium subsp. carpaticum (Porcius) Soó, Acta Bot. Acad. Sci. Hung. 23(3–4): 380 (1978)

= Heracleum alpinum Baumg., Enum. Stirp. Transsilv. 1: 215 (1816), non alior

≡ Heracleum carpaticum f. alpinum (Baumg.) Borza, Consp. Fl. Rom. 2: 204 (1949)

= Heracleum carpaticum f. palmatifidum Jáv., Magyar Bot. Lapok 9: 162 (1910)

= Heracleum carpaticum f. porcii Pax, Grundz. Pfl. Karp. 2: 70 (1908)

= Heracleum carpaticum f. typicum Nyár & Todor, Fl. Rep. Pop. Roman. 6: 625, 660 (1958)

– Heracleum carpaticum var. aconitifolium M.Pop. & Chrshan. [ex herb., nom. inval.], non Woronow

– Heracleum pollinianum Nyman, Consp. Fl. Eur. 2: 289 (1879) [p. p., tantum quod plantas ucrain. carpat.], non Bertol.

– Heracleum simplicifolium Herb. ex Nyman sensu Borza

– Heracleum simplicifolium Herb., Fl. Bucov.: 302 (1859) et Herb. ex Nyman, Consp. Fl. Eur. 2: 289 (1879) [p. p., tantum quod plantas ucrain. carpat.]

# Heracleum sphondylium subsp. transsilvanicum (Schur) Brummitt, Feddes Repert. 79: 65 (1968)

≡ Heracleum transsilvanicum Schur, Enum. Pl. Transsilv.: 267 (1866)

≡ Heracleum palmatum subsp. transsilvanicum (Schur) Nyman, Consp. Fl. Eur. 2: 289 (1879)

≡ Heracleum sphondylium subsp. transsilvanicum (Schur) Thellung, Oesterr. Bot. Z. 73: 211 (1924) [nom. invalid.]

= Heracleum palmatum Baumg., Enum. Stirp. Transsilv. 1: 215 (1816), non Crantz nec Rchb. *

≡ Pastinaca palmata (Baumg.) Calest., Webbia 1: 245 (1905)

≡ Heracleum alpinum subsp. palmatum (Baumg.) Briquet, Candollea 2: 16 (1924), non Crantz nec Rchb.

– Heracleum simplicifolium Herb., Fl. Bucov.: 302 (1859) et Herb. ex Nyman, Consp. Fl. Eur. 2: 289 (1879) [p. p., tantum quod plantas ucrain. carpat.]

# Achillea oxyloba subsp. schurii (Sch.Bip.) Heimerl, Denkschr. Kaiserl. Akad. Wiss., Wien. Math.-Naturwiss. Kl. 48: 137 (1884)

≡ Achillea schurii Sch.Bip., Oesterr. Bot. Wochenbl. 6: 300 (1856) *

≡ Ptarmica schurii Sch.Bip., Oesterr. Bot. Wochenbl. 6: 300 (1856)

≡ Anthemis schurii Sch.Bip., Oesterr. Bot. Wochenbl. 6: 300 (1856) et Sch.Bip. ex Heimerl, Denkschr. Acad. Wien 48: 137 (1884) [nom. nudum]

≡ Anthemis tenuifolia (Schur) Schur., Verh. Siebenb. Ver. Naturw. 2: 171 (1851) [nom. inval.], non Achillea tenuifolia Lam.

≡ Ptarmica tenuifolia (Schur) Schur, Enum. Pl. Transsilv.: 327 (1866), non Achillea tenuifolia Lam. *

= Achillea atrata Baumg., Enum. Stirp. Transsilv. 3: 141 (1816), non L.

= Achillea dacica Simonk., Termesz. Füzet. 10: 181 (1886) et Enum. Fl. Transsilv.: 317 (1886)

≡ Achillea schurii var. dacica (Simonk.) Prodan & Nyár., Fl. Rep. Pop. Rom. 9: 369 (1964)

= Achillea schurii f. pleiocephala Bommüller, Mitt. Thüringischen Bot. Vereins 30: 56 (1913)

= Anthemis alpina Baumg., Enum. Stirp. Transsilv. 3: 145 (1816), non alior

= Anthemis caespitosa Herbich, Flora 40: 509 (1857)

= Anthemis oxyloba Schur, Enum. Pl. Transsilv.: 884 (1866), non Achillea oxyloba (DC.) Sch.Bip.

≡ Ptarmica oxyloba Schur, Enum. Pl. Transsilv.: 326 (1866), non DC., non Achillea oxyloba (DC.) Sch.Bip.

= Anthemis pseudo-atrata Schur ex Schur, Enum. Pl. Transsilv.: 327 (1866)

≡ Ptarmica pseudo-atrata Schur ex Schur, Enum. Pl. Transsilv.: 327 (1866)

= Anthemis tenuifolia [unranked] a simplex monocephala Schur., Verh. Siebenb. Ver. Naturw. 4: 40 (1851)

= Anthemis tenuifolia [unranked] b ramosa polycephala Schur., Verh. Siebenb. Ver. Naturw. 4: 40 (1851)

≡ Ptarmica tenuifolia [unranked] b polycephala (Schur) Schur, Enum. Pl. Transsilv.: 327 (1866)

≡ Achillea schurii var. polycephala (Schur) Prodan & Nyár., Fl. Rep. Pop. Rom. 9: 369 (1964)

= Anthemis tenuifolia [unranked] c pilosa minima polaris Schur., Verh. Siebenb. Ver. Naturw. 4: 40 (1851)

= Ptarmica tenuifolia [unranked] a macrocephala Schur, Enum. Pl. Transsilv.: 327 (1866), non alior

# Antennaria carpatica subsp. carpatica (Wahlenb.) Hook. in Bluff et Fingerh., Comp. Fl. German. 2: 348 (1825)

≡ Gnaphalium carpathicum Wahlenb., Fl. Carpat. Princ.: 258, tab. 3, 260 (1814) et Fl. Suec., ed. 2, 2: 515 (1833)

≡ Antennaria carpatica (Wahlenb.) R.Br., Trans. Linn. Soc. London 12: 123 (1818) [nom. inval.] *

≡ Antennaria carpatica (Wahlenb.) Hook. in Bluff & Fingerh., Comp. Fl. German. 2: 348 (1825)

≡ Chamaezelum carpaticum (Wahlenb.) Link, Handbuch Erkennung nutz. häufigsten vorkomm. Gewachse 1: 719 (1829)

≡ Antennaria carpatica (Wahlenb.) Hook., Fl. Bor.-Amer. 1(suppl.): 329 (1834)

≡ Antennaria carpatica (Wahlenb.) Trautv., Acta Horti Petropolitani 6(1): 24 (1879)

= Gnaphalium wahlenbergii Sieber ex Steud., Nomencl. Bot., ed. 2. 1: 696 (1841)

– Antennaria alpina Ledeb., Fl. Ross. 2(2): 612 (1845–1846) [p. p., tantum quod plantas ucrain. carpat.], non (L.) Gaertn.

– Antennaria alpina auct fl. carpat. [e.g., Baumg.; Schur], non (L.) Gaertn.

– Gnaphalium alpinum Willd., Sp. Pl., ed. 4 3(3): 1883 (1803), non L. [p.p., tantum quod plantas ucrain. carpat.]

# Centaurea maramarosiensis (Jáv.) Czerep., Bot. Mater. Gerb. Bot. Inst. Komarova Akad. Nauk SSSR. 20: 395 (1960)

≡ Centaurea mollis f. maramarosiensis Jáv., Magyar Fl. 3: 1170 (1925)

≡ Centaurea montana subsp. mollis var. typica f. maramarosiensis (Jáv.) Dostál, Acta Bot. Bohem. 10: 69 (1931)

≡ Centaurea mollis subsp. marmarosiensis (Jáv.) Soó, Acta Bot. Acad. Sci. Hung. 13: 309 (1967)

≡ Centaurea montana subsp. maramarosiensis (Jáv.) Soják, Čas. Nár. Mus., Odd. Přír. 140(3–4): 131 (1972)

≡ Cyanus montanus subsp. maramarosiensis (Jáv.) Soják, Čas. Nár. Mus., Odd. Přír. 140 (3–4): 131 (1972)

≡ Cyanus maramarosiensis (Jáv.) Dostál, Folia Mus. Rerum Nat. Bohemiae Occid., Bot. 21: 14 (1984)

≡ Cyanus mollis subsp. marmarosiensis (Jáv.) Soó [nom. et. des. invalid ?]

= Centaurea mollis f. ramosa Czakó in Jáv., Magyar Fl. 3: 1170 (1925), non Centaurea ramosa (Gugler) Hayek

≡ Centaurea montana subsp. mollis var. ramosa (Czakó) Dostál, Acta Bot. Bohem. 10: 69 (1931), non Centaurea ramosa (Gugler) Hayek

# Centaurea phrygia subsp. carpatica (Porcius) Dostál, Bot. J. Linn. Soc. 71(3): 207 (1976)

≡ Centaurea plumosa var. carpatica Porcius, Enum. Pl. Phanerogam. Distr. Quondam Naszódiensis: 34 (1878) [nom. inval.]

≡ Centaurea carpatica (Porcius) Porcius, Magyar Növényt. Lapok 9: 128 (1885) *

≡ Centaurea carpatica (Porcius) Formánek, Oesterr. Bot. Z. 37: 153 (1887)

≡ Centaurea pseudophrygia f. intercedens subf. carpatica (Porcius) Gugler, Ann. Hist.-Nat. Musei Nat. Hungarici 6: 92 (1908)

≡ Centaurea carpatica (Porcius) Wagner, Cent. Hung.: 157 (1910)

≡ Jacea carpatica (Porcius) Soják, Čas. Nár. Mus., Odd. Přír. 140(3–4): 132 (1972)

≡ Jacea phrygia subsp. carpatica (Porcius) Dostál, Folia Mus. Rer. Nat. Bohem. Occid., Bot. 21: 14 (1984)

= Centaurea plumosa β [unranked] polycephala Porcius, Enum. Pl. Phanerogam. Distr. Quondam Naszódiensis: 34 (1878)

= Centaurea rodnensis Simonk., Enum. Fl. Transsilv.: 620 (1886) *

– Centaurea montana subsp. mollis (Waldst. & Kit.) Gugler, Ann. Hist.-Nat. Mus. Natl. Hung. 6: 104 (1907) sensu Katina

# Doronicum carpaticum (Griseb. et Schenk) Nyman, Syll. Fl. Eur. suppl.: 1 (1865)

≡ Aronicum scorpioides var. carpaticum Griseb. & Schenk in Wiegm., Arch. Naturgesch. 18(1): 342 (1852)

≡ Aronicum carpathicum (Griseb. & Schenk) Fuss, Progr. Gymn. Hermannstadt: 12 (1854)

≡ Aronicum carpaticum (Griseb. & Schenk) Schur, Bot. Rundr.: 71 (1853) et Verh. Siebenb. Ver. Naturw. 10: 137 (1859) *

≡ Aronicum carpathicum (Griseb. & Schenk) Schur, Bot. Rundr.: 71 (1853) et Verh. Siebenb. Ver. Naturw. 10: 137 (1859) [ortho. var.] *

≡ Doronicum grandiflorum subsp. carpaticum (Griseb. & A. Schenk) Rouy, Rev. Bot. Syst. Geogr. Bot. 1: 53 (1903)

≡ Doronicum columnae subsp. carpaticum (Griseb. & Schenk) Sóo, Scripta Bot. Mus. Transsilv. 3(3–5): 10 (1944)

= Aronicum barcense Simonk., Enum. Fl. Transsilv.: 322 (1886)

≡ Doronicum carpaticum var. barcense (Simonk.) Borbás, Termr. Füz. 19: 219 (1896)

= Aronicum carpaticum [unranked] a polyphyllum Schur, Enum. Pl. Transsilv.: 341 (1866)

= Aronicum latifolium Schur, Verh. Mitth. Siebenbürg. Vereins Naturwiss. Hermannstadt 2: 171 (1851) [nom. nudum], non Rchb.

= Doronicum cordatum var. asperum Borbás, Oesterr. Bot. Z. 28: 311 (1878)

= Doronicum pardalianches Heuff., [Enum. Pl. Banat. Temes.] Verh. K.K. Zool.-Bot. Ges. Wien 8: 137 (1858), non alior

= Doronicum orientale Kotschy, Verh. Zool.-Bot. Vereins Wien 3: 140 (1853) [nom. nudum], non alior

– Arnica scorpioides Baumg., Enum. Stirp. Transsilv. 3: 135 (1816), non alior

# Leucanthemum rotundifolium (Waldst. et Kit. in Willd.) DC., Prodr. 6: 46 (1838), non Opiz

≡ Chrysanthemum rotundifolium Waldst. & Kit. in Willd., Sp. Pl. 3(3): 2144 (1803) et Waldst. & Kit., Descr. Icon. Pl. Rar. Hung. 3: 262, t. 236 (1812)*

≡ Matricaria rotundifolia (Waldst. & Kit. in Willd.) Poir., Encycl. [J. Lamarck et al.] Suppl. 3.: 608 (1814)

≡ Leucanthemum rotundifolium (Waldst. & Kit. in Willd.) Baumg., Enum. Stirp. Transsilv. 3: 107 (1817)

≡ Leucanthemum rotundifolium (Waldst. & Kit. in Willd.) Schur, Enum. Pl. Transsilv.: 339 (1866)

≡ Tanacetum rotundifolium (Waldst. & Kit. in Willd.) Simonk., Enum. Fl. Transsilv.: 313 (1886), non DC. *

= Tanacetum waldsteinii Sch.Bip., Tanaceteen: 35 (1844)

≡ Pyrethrum waldsteinii (Sch.Bip.) Janka, Bot. Jahresber. (Just) 4: 1062 (1878)

≡ Leucanthemum waldsteinii (Sch.Bip.) Pouzar, Preslia 47: 158 (1975)

= Tanacetum waldsteinii var. ramosum Ilse &. Fritze, Verh. K.K. Zool.-Bot. Ges. Wien 20: 488 (1870)

– Chrysanthemum montanum Csató, Erd. Muz. [Az Erdélyi Múzeum-Egylet Évkönyveiben] 4: 82 (1868), non alior

# Saussurea porcii Degen, Magyar Bot. Lapok 3: 311 (1904)

= Saussurea alata Porcius & Czetz, Transilvania 15–16: 118 (1881), non DC.

= Saussurea serrata Janka, Oesterr. Bot. Z. 8: 200 (1858), non DC.

– Saussurea parviflora auct., non (Poir.) DC.

– Saussurea serrata auct. Transsilv., non DC.

# Scorzoneroides pseudotaraxaci (Schur) Holub, Folia Geobot. Phytotax. 12: 307 (1977)

≡ Leontodon pseudotaraxaci Schur, Enum. Pl. Transsilv.: 357 (1866) *

≡ Leontodon montanus subsp. pseudotaraxaci (Schur) Finch & P.D.Sell, Bot. J. Linn. Soc. 71: 242 (1976)

≡ Scorzoneroides montana (Lam.) J.Holub subsp. pseudotaraxaci [des. et nom. inval.]

= Leontodon clavatus Sagorski & Schneider, Fl. Centralkarpath. 2: 254 (1890–1891) *

= Leontodon medius Simonk., Enum. Fl. Transsilv.: 352 (1886) et Bot. Centralbl. 49: 268 (1892), non Apargia media Host

= Leontodon taraxaci var. tatricus Kotula, Distr. Pl. Mont. Tatr.: 356 (1890)

≡ *Leontodon tatricis* (Kotula) Woł., Fl. Pol. Exs.: 545 (1897) [ortho. var.]

≡ Leontodon tatricus (Kotula) Woł., Fl. Pol. Exs.: 545 (1897)

– Apargia aurea Baumg., Enum. Stirp. Transsilv. 3: 16 (1816), non (L.) F.W.Schmidt, non Leontodon aureum L., nec Ceracium aureum Schur.

– *Apargia taraxaci* Wahlenb., Fl. Carpat. Princ.: 235 (1814), non Willd.

– Leontodon taraxaci auct., non (L.) Loisel.

– *Leontodon taraxaci* R.Uechtr., Oesterr. Bot. Z. 14: 386 (1864) [nom. illeg], non Loisel.

– Leontodon pyrenaeus R.Uechtr., Oesterr. Bot. Wochenbl. 7: 370 (1857) [nom. illeg], non Gouan

– Leontodon pyrenaicus Hoborski, Oesterr. Bot. Wochenbl. 3: 19 (1853) [nom. illeg], non Gouan

# Senecio hercynicus subsp. ucranicus (Hodálová) Greuter, Willdenowia 33: 247 (2003)

≡ Senecio ucranicus Hodálová, Folia Geobot. 34 (3): 334 (1999), non Besser. *

# Campanula carpatica Jacq., Hort. Bot. Vindob. 1: 22, tab. 57 (1770), non C. carpatha Halácsy

≡ Campanula cordifolia Vuk., Linnaea 26(3): 328 (1854), non K.Koch

≡ Neocodon carpaticus (Jacq.) Kolak. & Serdyuk, Zametki Sist. Geogr. Rast. 40: 28 (1984)

= Campanula carpatica [unranked] alba (Voss) J.R.Duncan & V.C.Davies, Nursery Cat. (Duncan & Davies) 1925: 17 (1925)

= Campanula carpatica var. brachyphylla Morariu, Fl. Rep. Pop. Rom. 9: 76, 960 (1964)

= Campanula carpatica var. dasycarpa Schur, Enum. Pl. Transsilv.: 440 (1866)

≡ *Campanula dasycarpa* Fuss. ex Schur, Enum. Pl. Transsilv.: 440 (1866), non Kit. ex Schult.

≡ Campanula dasycarpa Schur ex Schur, Enum. Pl. Transsilv.: 440 (1866) [nom. illeg.], non Kit. ex Schult.

≡ Campanula carpatica f. dasycarpa (Schur) Tacik, Fl. Polska 12: 84 (1971)

= Campanula carpatica var. grandiflora Schur, Enum. Pl. Transsilv.: 440 (1866)

= Campanula carpatica var. hemisphaerica Schur, Enum. Pl. Transsilv.: 440 (1866)

= Campanula carpatica var. longifolia Morariu, Fl. Rep. Pop. Rom. 9: 79, 960 (1964)

= Campanula carpatica var. longifolia f. parviflora Săvul. ex Morariu & Nyár., Fl. Rep. Pop. Rom. 9: 79, 960 (1964)

= Campanula carpatica var. oreophila Schur, Enum. Pl. Transsilv.: 440 (1866)

= Campanula carpatica [unranked] pelviformis Froebel ex André, Rev. Hort. (Paris) 54: 509 (1882)

≡ Campanula turbinata f. pelviformis (Froebel ex André) Voss, Vilm. Blumengärtn. ed. 3, 1: 570 (1894)

= Campanula carpatica [unranked] riverslea J.R.Duncan & V.C.Davies, Nursery Cat. (Duncan & Davies) 1925: 17 (1925)

= Campanula carpatica var. porrecta Morariu, Fl. Rep. Pop. Rom. 9: 76, 959 (1964)

= Campanula carpatica var. porrecta f. minor Morariu, Fl. Rep. Pop. Rom. 9: 76, 959 (1964)

= Campanula carpatica var. schuriana Săvul. ex Morariu & Nyár., Fl. Rep. Pop. Rom. 9: 76, 960 (1964)

= Campanula carpatica var. subdasycarpa Morariu & Nyár., Fl. Rep. Pop. Rom. 9: 79, 960 (1964)

= Campanula carpatica [unranked] b subpilosa Schur, Enum. Pl. Transsilv.: 440 (1866)

≡ Campanula carpatica f. subpilosa (Schur) Tacik, Fl. Polska 12: 84 (1971)

*= Campanula carpatica* var. *transsilvanica* Schur, Bot. Rundreise [unpublished work]: 108 (1853) [nom. illeg.] et Verh. Mitth. Siebenbürg. Vereins Naturwiss. Hermannstadt 10: 174 (1859), non *Campanula transsilvanica* Schur ex Andrae

= Campanula carpatica var. turbinata f. rotundata Morariu, Fl. Rep. Pop. Rom. 9: 79, 960 (1964)

= Campanula carpatica var. tomentosa Kotschy, Verh. Zool.-Bot. Ges. Wien 3: 140 (1853)

= Campanula carpatica Baumg. ex Schur, Enum. Pl. Transsilv.: 440 (1866) [nom. inval.]

= Campanula carpatica L. ex Schur, Enum. Pl. Transsilv.: 440 (1866) [nom. inval.]

= Campanula oreophila Schur ex Schur, Enum. Pl. Transsilv.: 441 (1866)

= Campanula pseudocarpatica Schur, Enum. Pl. Transsilv.: 441 (1866)

= Campanula reniformis Schur, Enum. Pl. Transsilv.: 440 (1866)

= Campanula turbinata Schott, Nyman & Kotschy, Analect. Bot.: 14 (1854)

= Campanula turbinata f. alba Voss, Vilm. Blumengärtn. ed. 3, 1: 570 (1894)

= Campanula turbinata f. lilacina Voss, Vilm. Blumengärtn. ed. 3, 1: 570 (1894)

≡ Campanula carpatica var. turbinata (Schott, Nyman & Kotschy) Fuss, Fl. Transsilv. Exc.: 420 (1866)

≡ Campanula carpatica subsp. turbinata (Schott, Nyman & Kotschy) Nyman, Consp. Fl. Eur. 482 (1879)

≡ Campanula carpatica var. turbinata (Schott, Nyman & Kotschy) Nichols, Garden (London, 1871–1927) 45: 171 (1893)

– Campanula fergusonii A.M.Ferguson, Rev. Hort. 76: 557 (1904) [pro hybr., hort]

– Campanula hendersonii C.Wolley Dod, Gard. Chron. n.s., 18: 502 (1882) [hort.]

≡ Campanula carpatica var. hendersonii (C.Wolley Dod) W.T.Mill., Cycl. Amer. Hort. 231 (1900) [hort.]

– Campanula trans[s]ilvanica Schur, Verh. Mitth. Siebenbürg. Vereins Naturwiss. Hermannstadt 10: 174 (1859), non *C. transsilvanica* Schur ex Andrae (1855) nec Schur (1866)

# Campanula kladniana (Schur) Witasek, Abh. Zool.-Bot. Ges. Wien 1: 39 (1902)

≡ Campanula scheuchzeri var. kladniana Schur, Enum. Pl. Transsilv.: 443 (1866)

≡ Campanula rotundifolia subsp. kladniana (Schur) Tacik in Pawł. & Jasiewicz, Fl. Polska 12: 76 (1971)

# Campanula serrata (Kit. ex Schult.) Hendrych, Taxon 11: 123 (1962)

≡ Thesium serratum Kit. ex Schult., Oesterr. Fl. ed. 2, 1: 437 (1814)

≡ Thesium serratum Kit. ex D.Dietr., Syn. Pl. [D. Dietrich] 1: 878 (1839)

= Campanula arcuata Schur, Verh. Mitth. Siebenbürg. Vereins Naturwiss. Hermannstadt 10: 138 (1859)

≡ Campanula rotundifolia var. arcuata (Schur) Nyman, Consp. Fl. Eur.: 479 (1879)

≡ Campanula lanceolata subsp. arcuata (Schur) Simonk., Enum. Fl. Transsilv.: 385 (1887)

≡ Campanula pseudolanceolata var. arcuata (Schur) Porcius, Analele Acad. Romane ser. 2, 14: 196 (1893)

≡ Campanula napuligera f. arcuata (Schur) Hruby, Magyar Bot. Lapok 29: 217 (1930)

≡ Campanula napuligera. var. arcuata (Schur) Morariu, Fl. Rep. Pop. Rom. 9: 93 (1964)

= Campanula hornungiana Schur, Enum. Pl. Transsilv.: 442 (1866)

≡ Campanula pseudolanceolata var. hornungiana (Schur) Porcius, Anal. Acad. Rom., Ser. 2 14: 202 (1893)

≡ Campanula lanceolata var. hornungiana (Schur) Simonk., Enum. Fl. Transsilv.: 385 (1887)

≡ Campanula napuligera var. hornungiana (Schur) Morariu, Fl. Rep. Pop. Rom. 9: 97 (1964)

= Campanula kitaibeliana Roem. & Schult., Syst. Veg., ed. 15 bis 5: 90 (1819)

= Campanula lanceolata Neilr., Aufzählung Ungarn Slavonien Gefässpflanzen: 145 (1866), non alior

= Campanula microphylla Kit. ex Schult., Oesterr. Fl. ed. 2: 400 (1814), non Cav. [nom. illeg.]

= Campanula napuligera Schur, Enum. Pl. Transsilv.: 444 (1866) *

= Campanula napuligera f. albiflora Raclaru, Analele Univ. Bucureşti, Biol. Veg. 22: 125 (1973) [nom. nudum]

= Campanula napuligera var. alpiniformis Nyár. ex Morariu, Fl. Rep. Pop. Rom. 9: 96 (1964)

= Campanula napuligera f. angustifrons Hruby, Magyar Bot. Lapok 29: 217 (1930)

= Campanula napuligera subf. angustifrons Hruby, Magyar Bot. Lapok 29: 217 (1930)

= Campanula napuligera subf. brachyantha Hruby, Magyar Bot. Lapok 29: 220 (1930)

= Campanula napuligera f. genuina Hruby, Magyar Bot. Lapok 29: 220 (1930)

= Campanula napuligera f. glabrescens Hruby, Magyar Bot. Lapok 29: 218 (1930)

= Campanula napuligera var. hirsuta Hruby, Magyar Bot. Lapok 29: 220 (1930)

= Campanula napuligera f. humilis Hruby, Magyar Bot. Lapok 29: 218 (1930)

= Campanula napuligera f. intermedia Hruby, Magyar Bot. Lapok 29: 218 (1930)

= Campanula napuligera f. latifrons Hruby, Magyar Bot. Lapok, 29: 216 (1930)

= Campanula napuligera subf. latifrons Hruby, Magyar Bot. Lapok 29: 218 (1930)

= Campanula napuligera var. longisepala Nyár., Bul. Grad. Bot. Univ. Cluj 14: 95 (1934)

≡ Campanula napuligera f. longisepala (Nyár.) Morariu, Fl. Rep. Pop. Rom. 9: 101 (1964)

= Campanula napuligera f. parvula Morariu, Fl. Rep. Pop. Rom. 9: 96 (1964)

= Campanula napuligera f. robusta Hruby, Magyar Bot. Lapok 29: 220 (1930)

= Campanula napuligera f. savulescui Morariu, Fl. Rep. Pop. Rom. 9: 97 (1964)

= Campanula napuligera var. savulescui Morariu, Fl. Rep. Pop. Rom. 9: 96 (1964)

= Campanula napuligera var. scheuzeriformis Nyár., Bul. Grad. Bot. Univ. Cluj 14: 95 (1934)

≡ Campanula napuligera f. scheuzeriformis (Nyár.) Morariu, Fl. Rep. Pop. Rom. 9: 98. (1964)

= Campanula napuligera f. setulosa Morariu, Fl. Rep. Pop. Rom. 9: 97 (1964)

= Campanula napuligera f. simplex Hruby, Magyar Bot. Lapok 29: 216 (1930)

= Campanula napuligera f. stenophylloides Nyár., Bul. Grad. Bot. Univ. Cluj 14: 96 (1934)

≡ Campanula napuligera var. stenophylloides (Nyár.) Morariu, Fl. Rep. Pop. Rom. 9: 98 (1964)

= Campanula napuligera var. stricta Hruby, Magyar Bot. Lapok 29: 216 (1930)

= Campanula napuligera subf. tenella Hruby, Magyar Bot. Lapok 29: 220 (1930)

= Campanula napuligera var. umbrosa Hruby, Magyar Bot. Lapok 29: 218 (1930)

= Campanula pseudolanceolata Pant., Magyar Növényt. Lapok 6: 162 (1882) et Pant. ex. A.Kern., Sched. Fl. Exs. Austro-Hung. [Kerner] 9: 37 (1902) *

≡ Campanula rhomboidalis subsp. pseudolanceolata (Pant.) Nyman, Consp. Fl. Eur. Suppl. 2: 208 (1889)

≡ Campanula polymorpha f. pseudolanceolata (Pant.) Hruby, Magyar Bot. Lapok 29: 203 (1930)

= Campanula pseudolanceolata f. albiflora Săvul., Stud. Sp. Campanula: 81 (1916)

= Campanula pseudolanceolata f. elatior Săvul., Stud. Sp. Campanula: 78 (1916)

≡ Campanula napuligera var. elatior (Săvul.) Morariu, Fl. Rep. Pop. Rom. 9: 94 (1964)

= Campanula pseudolanceolata f. minima Săvul., Stud. Sp. Campanula: 81 (1916)

≡ Campanula napuligera f. minima (Săvul.) Morariu, Fl. Rep. Pop. Rom. 9: 97 (1964)

= Campanula pseudolanceolata var. porcii Săvul., Stud. Sp. Campanula: 84 (1916)

= Campanula pseudolanceolata subsp. semiamplexicaulis Vladescu & Săvul., Stud. Sp. Campanula: 86 (1916)

≡ Campanula napuligera f. semiamplexicaulis (Vladescu & Săvul.) Morariu, Fl. Rep. Pop. Rom. 9: 98 (1964)

= Campanula pseudolanceolata f. transsilvanica Săvul., Stud. Sp. Campanula: 78 (1916)

≡ Campanula napuligera var. transsilvanica (Săvul.) Morariu, Fl. Rep. Pop. Rom. 9: 93 (1964)

= Campanula pseudolanceolata f. umbraticola Săvul., Stud. Sp. Campanula: 78 (1916)

= Campanula redux Schott, Nyman & Kotschy, Analect. Bot.: 9 (1854)

≡ Campanula napuligera var. redux (Schott, Nyman & Kotschy) Nyman, Consp. Fl. Eur.: 479 (1879)

≡ Campanula napuligera var. redux (Schott, Nyman & Kotschy) Hruby, Magyar Bot. Lapok 29: 219 (1930) [nom. inval.]

= Campanula rhomboidalis var. angustifolia Neilr., Aufz. Ungarn Slavon. Gefässpfl.: 145 (1866)

= Campanula rhomboidea [unranked] β foliis ovato-oblongis Wahlenberg, Fl. Carpat.: 60 (1814), non L.

= Campanula rotundifolia var. alpina Schur, Enum. Pl. Transsilv.: 444 (1866) [nom. illeg.], non Tuck.

= Campanula rotundifolia var. dentata Schur, Enum. Pl. Transsilv.: 444 (1866), non N.Coleman

= Campanula rotundifolia var. grandiflora J.A.Knapp, Pfl. Galiz.: 173 (1872) [nom. illeg.], non alior

– Campanula serrata var. elatior (Săvul.) Tasenkevych [nom. provis. et inval., ex herb. LWS]

– Campanula serrata var. elatior f. latifrons Hruby [comb. inval. ex herb. CHER] *

– Campanula serrata var. hornungiana (Schur.) Tasenkevych [nom. provis. et inval., ex herb. LWS]

– Campanula lancifolia Schur, Enum. Pl. Transsilv.: 445 (1866) [nom. illeg.] sensu Błocki, non Witasek

# Campanula tatrae subsp. tatrae Borbás, Magyar Bot. Lapok 1: 319 (1902)

= Campanula kladniana subsp. polymorpha Witasek, Magyar Bot. Lapok 5: 239 (1906)

≡ Campanula polymorpha (Witasek) Prain, Index Kew. Suppl. 4: 35 (1913), non Banks & Sol. ex A.DC. *

≡ Campanula kladniana var. polymorpha (Witasek) Pawł., Acta Soc. Bot. Pol. 1: 5 (1923)

≡ Campanula rotundifolia subsp. polymorpha (Witasek) Tacik in Jasiewicz, Monogr. Bot. 20: 254 (1965)

= Campanula polymorpha var. intercedens Hruby, Magyar Bot. Lapok 29: 199 (1930), non C. witasekiana var. intercedens Hruby

= Campanula polymorpha var. intercedens f. angustifolia Hruby, Magyar Bot. Lapok 29: 200 (1930)

= Campanula polymorpha var. intercedens f. exigua Hruby, Magyar Bot. Lapok 29: 200 (1930)

= Campanula polymorpha var. intercedens f. latifolia Hruby, Magyar Bot. Lapok 29: 199 (1930)

= Campanula polymorpha var. intercedens f. reflectans Hruby, Magyar Bot. Lapok 29: 200 (1930)

= Campanula polymorpha var. intercedens f. umbrosa Hruby, Magyar Bot. Lapok 29: 200 (1930)

= Campanula polymorpha var. lepida Nyár. ex Hruby, Magyar Bot. Lapok 29: 201 (1930)

≡ Campanula polymorpha var. typica f. lepida (Nyár.) Hruby, Magyar Bot. Lapok 29: 202 (1930)

= Campanula polymorpha var. pluriflora Nyár. ex Hruby, Magyar Bot. Lapok 29: 198 (1930)

≡ Campanula polymorpha var. praticola f. pluriflora (Nyár.) Hruby, Magyar Bot. Lapok 29: 199 (1930)

= Campanula polymorpha var. praticola Hruby, Magyar Bot. Lapok 29: 198 (1930), non C. witasekiana var. praticola Hruby

= Campanula polymorpha var. praticola f. hirta (Nyár.) Hruby, Magyar Bot. Lapok 29: 199 (1930)

= Campanula polymorpha var. stenophylla f. brachyphylla Hruby, Magyar Bot. Lapok 29: 206 (1930)

= Campanula polymorpha var. stenophylla f. genuina Hruby, Magyar Bot. Lapok 29: 206 (1930)

= Campanula polymorpha var. stenophylla f. gracilis Hruby, Magyar Bot. Lapok 29: 206 (1930)

= Campanula polymorpha var. typica Hruby, Magyar Bot. Lapok 29: 198, 201 (1930)

= Campanula polymorpha var. typica f. fasciculata Nyár. ex Hruby, Magyar Bot. Lapok 29: 202 (1930)

= Campanula polymorpha var. typica f. fasciculata subf. deltoidea Hruby, Magyar Bot. Lapok 29: 203 (1930)

= Campanula polymorpha var. typica f. kladnianioides Nyárady ex Hruby, Magyar Bot. Lapok 29: 201 (1930)

= Campanula polymorpha var. typica f. latifolia Hruby, Magyar Bot. Lapok 29: 201 (1930)

= Campanula polymorpha var. typica f. latifolia subf. umbrosa Hruby, Magyar Bot. Lapok 29: 202 (1930)

= Campanula polymorpha var. typica f. lepida subf. reflectans Hruby, Magyar Bot. Lapok 29: 202 (1930)

= Campanula polymorpha var. typica f. saxiphila Hruby, Magyar Bot. Lapok 29: 204 (1930)

= Campanula polymorpha var. typica f. saxiphila subf. reflectans Hruby, Magyar Bot. Lapok 29: 205 (1930)

= Campanula scheuchzeri [unranked] β dacica Porcius, Enum. Pl. Phan. Naszód.: 37 (1878)

≡ Campanula scheuchzeri var. dacica Porcius, Fl. Naseud.: 98 (1885)

= Campanula scheuchzeri var. stenophylla Schur, Enum. Pl. Transsilv.: 443 (1866)

≡ Campanula kladniana subsp. stenophylla (Schur) Witasek, Magyar Bot. Lapok 5: 238 (1906)

≡ Campanula stenophylla (Schur) Witasek, Magyar Bot. Lapok 5: 238 (1906), non Boiss. & Heldr.

≡ Campanula stenophylla (Schur) Prain, Index Kew. Suppl. 4: 35 (1913) [nom. inval.]

≡ Campanula polymorpha var. stenophylla (Schur) Hruby, Magyar Bot. Lapok 29: 205 (1930)

– Campanula carnica auct. fl. transsilv., non Schiede

– Campanula consanguinea Simonk., Enum. Fl. Transsilv.: 385 (1886) [p. p.], non Schott

– Campanula kladniana (Schur) Witasek, Abh. Zool.-Bot. Ges. Wien 1: 39 (1902) [p. p. min., non sensu Schur orig.]

– Campanula linifolia auct. [e.g., Wahlenb.], non Jacq.

– Campanula polymorpha f. sciaphila Hruby, Magyar Bot. Lapok 29: 204 (1930) [nom. et des. inval.]

– Campanula rotundifolia L., Sp. Pl. 1: 163 (1753) [p. p. minor, tantum quod plantas ucrain. carpat.], non alior *

– Campanula pusilla auct. fl. ucrain. carpat., non Haenke

– Campanula scheuchzeri auct. [e.g., Reuss, Května Slov.: 278 (1853); Sagorski & Schneider, Fl. Centralkarp.: 369 (1891)], non Vill. *

# Phyteuma tetramerium Schur, Verh. Mitth. Siebenbürg. Vereins Naturwiss. Hermannstadt 4: 47 (1853)

≡ Phyteuma tetramerum Schur, Verh. Mitth. Siebenbürg. Vereins Naturwiss. Hermannstadt 4: 47 (1853) [ortho. var.]

= Phyteuma spicatum Baumg., Enum. Stirp. Transsilv. 1: 158 (1816), non L. nec Lapeyr.

= Phyteuma spicatum Nyman, Consp. Fl. Eur.: 484 (1879) [nom. illeg.], non L. nec Lapeyr.

– Phyteuma spicatum var. tetramerum (Schur) Nyman sensu auct. multipl. [nom. nudum]

# Phyteuma vagneri A.Kern in Vágner, Máram. Növ.: 192 (1875) et A.Kern., Sched. Fl. Exs. Austro-Hung. [Kerner] 3: 107 (1884)

≡ Phyteuma atropurpureum Schur, Verh. Mitth. Siebenbürg. Vereins Naturwiss. Hermannstadt 3: 88 (1852) [nom. nudum], non Hoppe

≡ Phyteuma nigrum var. atropurpureum Schur, Enum. Pl. Transsilv.: 430 (1866)

≡ Phyteuma spiciforme Rochel, Bot. Reise Banat: 69 (1838) [nom. nudum] et Rochel ex Domin & Podp., Klic Ulpne Kvet. Rep. Ceskoslov.: 542 (1928)

= Phyteuma michelii Sternh., Fl. Sieb.: 20 (1846), non alior

= Phyteuma vagneri f. alpinum Rich. Schulz, Monogr. Phyteuma: 79 (1904)

= Phyteuma vagneri f. brevibracteatum Rich. Schulz, Monogr. Phyteuma: 78 (1904)

= Phyteuma vagneri f. grossidentatum Rich. Schulz, Monogr. Phyteuma: 78 (1904)

= Phyteuma vagneri f. latibracteatum Rich. Schulz, Monogr. Phyteuma: 78 (1904)

= Phyteuma vagneri var. pallida Porcius, Enum. Pl. Phanerogam. Distr. Quondam Naszódiensis: 37 (1878)

– Phyteuma betonicaefolium Baumg., Mant.: 16 (1846) et auct. transsilv., non Vill. [nom. nudum?]

– Phyteuma halleri auct. transsilv., non All.

– Phyteuma nigrum auct. [e.g., Baumg.], non Schmalh.

– Phyteuma ovatum auct. [e.g., Baumg.], non Schmalh.

# Pulmonaria filarszkyana Jáv., Bot. Közlem. 15: 52 (1916)

≡ Pulmonaria rubra var. filarszkyana (Jáv.) Guşul., Bul. Fac. St. Cern. 3: 330 (1929) *

≡ Pulmonaria rubra subsp. filarszkyana (Jáv.) Domin, Preslia 13-15: 175, in adnot. (1935)

– Pulmonaria angustifolia Kern., Monogr. Pulm.: 9 (1878) [p. p., quoad plantas marmaros. et rodn.], non L.

– Pulmonaria dacica (Simonk.) Simonk., Enum. Fl. Transsilv.: 406 (1886) [p. p.] *

– Pulmonaria dacica (Simonk.) Porcius [p. p., nom et des. invalid]

– Pulmonaria rubra var. dacica Simonk., Math. Termeszettud. Közlem. 15: 583 (1878) [p. p.]

# Symphytum cordatum Waldst. et Kit. ex Willd., Neue Schriften Ges. Naturf. Freunde Berlin 2: 121 (1799), non M.Bieb.

≡ Symphytum cordatum Waldst. & Kit., Descr. Icon. Pl. Hung. 1: 6, t. 7 (1799–1802), non M.Bieb.

= Symphytum cordifolium Baumg., Enum. Stirp. Transsilv. 1: 126 (1816) *

= Symphytum pannonicum Pers., Syn. Pl. [Persoon] 1: 161 (1805) *

– Symphytum cordatum M.Bieb., Fl. Taur.-Caucas. 1: 130 (1808) [p. p., nom. inval.]

# Arabidopsis neglecta (Schult.) O'Kane et Al-Shehbaz, Novon 7(3): 326 (1997)

≡ Arabis neglecta Schult., Oesterr. Fl., ed. 2: 248 (1814) *

≡ Erysimum neglectum (Schult.) Kuntze, Revis. Gen. Pl. 2: 933 (1891)

≡ Cardaminopsis neglecta (Schult.) Hayek, Fl. Steiermark 1: 480 (1908) *

= Arabis transsilvanica Schur, Enum. Pl. Transsilv.: 43 (1866)

= Arabis floribunda Schur, Enum. Pl. Transsilv.: 44 (1866)

= Arabis glareosa Schur, Verh. Mitth. Siebenbürg. Vereins Naturwiss. Hermannstadt 1: 106 (1850) et Verh. Mitth. Siebenbürg. Vereins Naturwiss. Hermannstadt 4: 59 (1853)

≡ Cardaminopsis neglecta subsp. glareosa (Schur) Soó, Acta Bot. Acad. Sci. Hung. 16 (3–4): 371 (1971)

– Cardamine enneaphyllos Turcz. [nom. inval., ex herb. KW], non (L.) Crantz ex Crantz

– Dentaria enneaphyllos auct. flora ucrain. carpat., non L. [ex herb. LWS] *

# Cardamine glanduligera O.Schwarz, Repert. Spec. Nov. Regni Veg. 46: 188 (1939)

≡ Dentaria glandulosa Waldst. & Kit., Descr. Icon. Pl. Hung. 3: 302, t. 272 (1801)

≡ Cardamine glandulosa (Waldst. & Kit.) Schmalh., Fl. Sredn. Yuzhn. Rossii 1: 50 (1895) [nom. illeg.], non Blanco

≡ Crucifera novemfolia E.H.L.Krause, Deutschl. Fl. (Sturm), ed. 2 6: 118 (1902)

# Erysimum witmannii subsp. transsilvanicum (Schur) P.W.Ball, Feddes Repert. 69: 151 (1964)

≡ Erysimum transsilvanicum Schur, Enum. Pl. Transsilv.: 57 (1866) *

= Erysimum baumgartenianum Jáv., Magyar Bot. Lapok 11: 30 (1912), non Schur

= Erysimum czetzianum Schur, Enum. Pl. Transsilv.: 57 (1866)

≡ Erysimum czetzianum Shur ex Jáv., Magyar Bot. Lapok 11: 29 (1912)

≡ Erysimum witmannii subsp. czetzianum (Schur) Zapał. [ex herb. Mądalski, nom. inval. ?]

≡ Erysimum witmannii var. czetzianum (Schur) Borza, Bul. Grăd. Bot. Cluj 26: (1946)

≡ Erysimum transsilvanicum f. czetzianum (Schur) Nyár., Fl. Rep. Pop. Roman. 3: 177 (1955)

= Erysimum pannonicum f. viridis Simonk., Termesz. Fuzet. 5: 55 (1881)

= Erysimum pumilum var. transilvanica Schur, Verh. Mitth. Siebenburg. Vereins Naturwiss. Hermannstadt 10: 143 (1859)

= Erysimum transsilvanicum f. luxurians Nyár., Fl. Rep. Pop. Roman. 3: 177, 640 (1955)

= Erysimum transsilvanicum f. rarifolium Nyár., Fl. Rep. Pop. Roman. 3: 177, 640 (1955)

= Erysimum witmannii var. czetziano Nyár., Fl. Rep. Pop. Roman. 3: 174 (1955)

– Erysimum cheiranthus Herbich, Fl. Bucovina: 354 (1859), non alior

– Erysimum pannonicum auct. carpat., non Crantz

– Erysimum odoratum Baumg., Enum. Stirp. Transsilv. 2: 262 (1816) [p. p.], non Ehr.

– Erysimum wahlenbergii Simonk., Enum. Fl. Transsilv.: 85 (1886), non Asch. & Engl.

– Erysimum witmannii auct. flora ucrain. carpat. et Grec., Consp. Fl. Rom.: 61 (1891) [p. p.], non Zaw. *

# Noccaea dacica subsp. dacica (Heuff.) F.K.Mey, Feddes Repert. 84(5-6): 464 (1973)

≡ Thlaspi dacicum Heuff., Oesterr. Bot. Z. 8: 26 (1858) et Verh. K.K. Zool.-Bot. Ges. Wien 8 (Abh.): 61 (1858) *

≡ Noccaea dacica (Heuff.) F.K.Mey., Feddes Repert. 84(5-6): 464 (1973)

= Thlaspi dacicum [unranked] β rodnense Porcius, Enum. Pl. Phanerogam. Distr. Quondam Naszódiensis: 7 (1878)

= Thlaspi dacicum [unranked] β transsilvanicum Porcius, Fl. Năsăud: 169 (1881)

= Thlaspi commutatum Rochel, Bot. Reise Banat: 83 (1838), non Reiche

= Thlaspi korongianum Czetz ex Nyman, Syll. Suppl.: 37 (1865)

≡ Thlaspi corongianum Czetz ex Nyman, Consp. Fl. Eur. 1: 63 (1878) [ortho. var.]

= Thlaspi trojagense Zapał., Rozprawy Wydziału Mat.-Przyrod. Akad. Um., Dział B. Nauki Biol. 13: 316, 317 (1913)

= Thlaspi trojagense f. abbreviatum Zapał., Rozprawy Wydziału Mat.-Przyrod. Akad. Um., Dział B. Nauki Biol. 13: 317 (1913)

– Thlaspi alpestre auct. [e.g., Schur, Fuss., Baumg.], non L.

– Thlaspi rotundifolium auct. fl. transsilv., non Gaud

# Dianthus spiculifolius Schur, Enum. Pl. Transsilv.: 98 (1866), non Borbás

≡ Dianthus kitaibelii subsp. spiculifolius (Schur) Novák, Sborník Klubu Přírodověd. v Praze. Sv. 4. 1914–1920, Č. 4: 23(1) (1922) et Věst. Král. české spol. nauk. Tř. mat.-přírod. 1923(11): 30 (1924)

≡ Dianthus plumarius subsp. spiculifolius (Schur) Baksay, Symposia Biol. Hung. 12: 153 (1972)

≡ Dianthus petraeus subsp. spiculifolius (Schur) Ciocârlan, Illustr. Fl. Rom.: 217-223 (2000)

= Dianthus acicularis Schur, Enum. Pl. Transsilv.: 98 (1866), non Fisch. ex Ledeb.

= Dianthus brachyanthus Schur, Enum. Pl. Transsilv.: 96 (1866), non Boiss.

= Dianthus carpathicus Borbás, Termesz. Fuzet. 12: 44 (1889), non Woł. [nom. inval.]

= Dianthus microche[i]lus B.S.Williams, Pinks Centr. Eur.: 37 (1890) et B.S.Williams ex Wettst., Oesterr. Bot. Z. 41: 176 (1891)

= Dianthus petraeus Janka, Bot. Közlem. 12: 187 (1913), non Waldst. & Kit. nec M.Bieb.

= Dianthus petraeus Kerner, Oesterr. Bot. Z. 18: 18, 126 (1868) [nom. nudum], non Waldst. & Kit. nec M.Bieb.

= Dianthus plumarius Baumg., Enum. Stirp. Transsilv. 1: 390 (1816) et auct. transsilv., non L. nec Gunnerus

= Dianthus plumarius var. erythrocalyx Schott ex Simonk., Enum. Fl. Transsilv.: 121 (1886)

= Dianthus plumarius var. hungaricus Andrae, Bot. Zeitung 11: 436 (1853), non alior

≡ Dianthus hungaricus (Andrae) Simonk., Enum. Fl. Transsilv.: 121 (1886), non alior

= Dianthus serotinus Barth, Verh. Mitth. Siebenbürg. Vereins Naturwiss. Hermannstadt 19: 144 (1868) [nom. nudum], non Waldst. & Kit.

= Dianthus serotinus Salzer, Verh. Mitth. Siebenbürg. Vereins Naturwiss. Hermannstadt 15: 50 (1864) [nom. nudum], non Waldst. & Kit.

= Dianthus spiculifolius f. petraeiformis Novák, Sborník Klubu Přírodověd. v Praze. Sv. 4. 1914–1920, Č. 4: 25(3) (1922)

# Sabulina oxypetala (Woł.) Mosyakin & Fedor., Phytotaxa 231(1): 96 (2015)

≡ Alsine oxypetala Woł., Spraw. Komis. Fizjograf. 22(2): 214 (1888)

*≡* Alsine verna [unranked] oxypetala Zapał. [nom. inval. ?]

*≡* Alsine zarencznyi var. oxypetala Woł., Sprawozd. Kom. Fizjograf. 21(2): 111–139 (1887) [?]

*≡* Minuartia verna [unranked] B attica [unranked] oxypetala Graebn. in Asch. & Graebn., Syn. Mitteleur. Fl. 5(1): 745 (1918)

*≡* Minuartia oxypetala (Woł.) Kulczyński, Fl. Polska 2: 231 (1921) *

*≡* Minuartia verna var. oxypetala (Woł.) Prodan, Fl. Rep. Pop. Rom. 2: 86 (1953)

*≡* Minuartia verna subsp. oxypetala (Woł.) G.Halliday, Feddes Repert. 69: 13 (1964)

*≡* Sabulina verna subsp. oxypetala (Woł.) Dillenb. & Kadereit, Taxon 63(1): 88 (2014)

= Alsine zarencznyi var. neglecta Zapał., Consp. Fl. Galic. Crit. 3: 26 (1911)

= Alsine zarencznyi var. neglecta f. ramificans Zapał., Consp. Fl. Galic. Crit. 3: 27 (1911)

= Alsine zarencznyi var. neglecta f. subcaespitosa Zapał., Consp. Fl. Galic. Crit. 3: 27 (1911)

= Alsine zarencznyi var. neglecta f. subcolorata Zapał., Consp. Fl. Galic. Crit. 3: 27 (1911)

= Alsine zarencznyi [unranked] c oxypetala f. acutissima Zapał., Consp. Fl. Galic. Crit. 3: 27 (1911)

= Alsine zarencznyi [unranked] c oxypetala f. micropetala Zapał., Consp. Fl. Galic. Crit. 3: 28 (1911)

*≡* Minuartia verna var. oxypetala f. micropetala (Zapał.) Prodan, Fl. Rep. Pop. Rom. 2: 86 (1953)

– Minuartia zarencznii auct. [i.e., Chopyk 1976], non (Zapał.) Klokov

**Sabulina pauciflora (Kit.) A.Novikov, *comb. nov.***

*≡* Arenaria pauciflora Kit., Linnaea 32(4–5): 510 (1864), non Prodan

*≡* Alsine pauciflora Kit. ex Nyman, Consp. Fl. Eur. 1: 119 (1878)

*≡* Minuartia pauciflora (Kit.) Dvořaková, Preslia 75(4): 350 (2003) *

= Alsine verna [unranked] δ carpatica Porcius, Enum. Pl. Phanerogam. Distr. Quondam Naszódiensis: 11 (1878) et Anal. Acad. Rom.: 54 (1893)

*≡* Minuartia verna subsp. gerardii [unranked] b. carpatica (Porcius) Graebn. in Asch. & Graebn., Syn. Mitteleur. Fl. 5(1): 749 (1918) *

= Alsine zarencznyi Zapał., Bull. Int. Acad. Sci. Cracovie, Cl. Sci. Math. 1910(3B): 168 (1910) et Consp. Fl. Galic. Crit. 3: 25 (1911) [excl. var. c]

*≡* Alsine verna [unranked] a. zarencnyi (Zapał.) Hermann, Fl. Deutschl. Fennoskand.: 185 (1912)

*≡* Minuartia zarecznyi (Zapał.) Klokov, Fl. UkrSSR 4: 480 (1952) *

*≡* Minuartia zarencznii (Zapał.) Klokov, Fl. UkrSSR 4: 480 (1952) [ortho. var.]

= Alsine zarencznyi var. divestita Zapał., Consp. Fl. Galic. Crit. 3: 27 (1911)

*≡* Minuartia zarecznyi var. divestita (Zapał.) Tzvelev, Bot. Zhurn. 87(3): 125 (2002)

= Alsine zarencznyi var. pseudogerardiana Zapał., Consp. Fl. Galic. Crit. 3: 28 (1911)

= Alsine zarencznyi var. zarencznyi f. bryophila Zapał., Consp. Fl. Galic. Crit. 3: 26 (1911)

= Alsine zarencznyi var. zarencznyi f. minima Zapał., Consp. Fl. Galic. Crit. 3: 26 (1911)

= Alsine zarencznyi var. zarencznyi f. paucicaulis Zapał., Consp. Fl. Galic. Crit. 3: 26 (1911)

= Alsine zarencznyi var. zarencznyi f. subpurpuea Zapał., Consp. Fl. Galic. Crit. 3: 26 (1911)

= Alsine zarencznyi var. zarencznyi f. supraglandulosa Zapał., Consp. Fl. Galic. Crit. 3: 26 (1911)

– Alsine gerardii auct. flora carpat., non Willd.

– Arenaria gerardii auct. fl. carpat., non Willd.

– Alsine verna auct. fl. carpat., non (L.) Wahlenb. nec Bartl

– Alsine verna Knapp, Pfl. Galic. u. Bukov.: 331 (1872) [p. p.], non Wahlenb. s. str. *

– Minuartia gerardii auct. fl. carpat., non (Willd.) Hayek *

– Minuartia verna auct. flora carpat., non (L.) Hiern *

– Minuartia verna Kulczyński, Fl. Polska 2: 230 (1921), non (L.) Hiern.

– Minuartia verna [unranked] α caespitosa (Ehrn.) Graebn. in Asch. & Graebn., Syn. Mitteleur. Fl. 5(1): 742 (1918) sensu Tovt [ex herb. UU] *

– Minuartia verna subsp. gerardii (Willd.) Graebn. in Asch. & Graebn., Syn. Mitteleur. Fl. 5(1): 747 (1918) [p. p., tantum quod plantas carpat.], non Sabulina verna subsp. gerardii (Willd.) Dillenb. s. str. *

– Minuartia verna var. gerardi Kulczyński, Fl. Polska 2: 230 (1921), non Schinz. & Keller

– Sabulina gerardii auct. fl. carpat., non (Willd.) Rchb.

– Sabulina verna subsp. gerardii auct. fl. carpat., non (Willd.) Dillenb.

– Tryphane gerardi auct. fl. carpat., non (Willd.) Rchb.

# Silene nutans subsp. dubia (Herbich) Zapał., Bull. Int. Acad. Sci. Cracovie, Cl. Sci. Math., Sér. B, Sci. Nat. 11: 151 (1911)

≡ Silene dubia Herbich, Fl. Bucovina: 388 (1859), non alior

≡ Silene dubia Herbich ex Rohrb., Monogr. Silene: 217 (1869), non alior

≡ Silene nutans var. dubia (Herbich) Zapał., Sprawozd. Kom. Fizjograf. 24: 111 (1889)

= Silene dubia var. glabriuscula (Zapał.) Guşul., Fl. Rep. Pop. Rom. 2: 179 (1953)

= Silene dubia var. hormuzakii Guşul., Fl. Rep. Pop. Rom. 2: 179 (1953)

= Silene dubia var. hormuzakii f. acaulis Guşul., Fl. Rep. Pop. Rom. 2: 179, 665 (1953) [monster forma]

= Silene nutans subsp. dubia var. dubia f. apricorum Zapał., Consp. Fl. Galic. Crit. 3: 195 (1911)

≡ Silene dubia var. hormuzakii f. apricorum (Zapał.) Graebn. in Asch. & Graebn. emend. Guşul., Fl. Rep. Pop. Rom. 2: 179 (1953)

= Silene nutans subsp. dubia [unranked] b herbichii Zapał., Consp. Fl. Galic. Crit. 3: 195 (1911)

≡ Silene dubia var. hormuzakii f. herbichii (Zapał.) Graebn. in Asch. & Graebn. emend. Guşul., Fl. Rep. Pop. Rom. 2: 179 (1953)

= Silene nutans subsp. dubia [unranked] a kelemenensis Zapał., Consp. Fl. Galic. Crit. 3: 195 (1911)

≡ Silene dubia var. hormuzakii f. kelemenensis (Zapał.) Graebn. in Asch. & Graebn. emend. Guşul., Fl. Rep. Pop. Rom. 2: 179 (1953)

= Silene nutans subsp. dubia [unranked] a kelemenensis f. lilacina Zapał., Consp. Fl. Galic. Crit. 3: 195 (1911)

≡ Silene dubia var. hormuzakii f. lilacina (Zapał.) Guşul., Fl. Rep. Pop. Rom. 2: 179 (1953)

= Silene nutans subsp. dubia var. dubia f. luxuriosa Zapał., Consp. Fl. Galic. Crit. 3: 195 (1911)

= Silene nutans subsp. dubia var. dubia f. tenuis Zapał., Consp. Fl. Galic. Crit. 3: 195 (1911)

= Silene nutans [unranked] c. glabriuscula Zapał., Consp. Fl. Galic. Crit. 3: 192 (1911)

= Silene nutans [unanked] β transsilvanica Grec., Consp. Fl. Rom.: 109 (1898)

= Silene transsilvanica Schur, Oesterr. Bot. Z. 8: 22 (1858) [nom. nudum] et Oesterr. Bot. Z. 10: 181 (1860)

= Silene transsilvanica var. angustifolia Hormuz., Oesterr. Bot. Z. 61: 147 (1911)

*= Silene saxatilis* [unranked] a racemosa Schur, Enum. Pl. Transsilv.: 101 (1866)

*= Silene saxatilis* [unranked] a robustior Schur, Enum. Pl. Transsilv.: 101 (1866)

≡ Silene dubia var. hormuzakii f. robustior (Schur) Graebn. in Asch. & Graebn. emend. Guşul., Fl. Rep. Pop. Rom. 2: 179 (1953)

– Silene saxatilis Schur, Enum. Pl. Transsilv.: 101 (1866), non Sims nec M.Bieb.

# Silene zawadzkii Herbich, Enum. Pl. Galic. Bucow.: 191 (1835)

≡ Melandrium zawadskii (Herbich) A.Braun, Flora 26: 387 (1843) [nom. nudum]

≡ Silenanthe zawadzkii (Herbich) Griseb. & Schenk, Arch. Naturgesch. (Berlin) 18(1): 300 (1852)

≡ Elisanthe zawadzkii (Herbich) Fuss, Fl. Transsilv.: 106 (1866)

≡ Elisanthe zawadskii (Herbich) Klokov, Fl. UkrSSR 4: 574 (1952) [nom. illeg.]

# Armeria pocutica Pawł., Fragm. Florist. Geobot. 8: 399 (1962)

– Armeria elongata auct., non (Hoffm.) Koch

– Armeria maritima subsp. elongata auct., non (Hoffm.) Bonnier

– Armeria vulgaris auct., non Willd.

# Scabiosa lucida subsp. barbata Nyár., Enum. Pl. Vasc. Cheia Turzii: 280 (1939)

≡ Scabiosa pseudobanatica subsp. barbata (Nyár.) Chrtek, Preslia 57: 201 (1985)

≡ Scabiosa barbata Nyár. ex Chopyk & Fedoronchuk, Fl. Ukr. Carpath.: 436 (2015) [des. et nom. invalid.] *

= Asterocephalus lucidus [unranked] a. alpicolus Schur, Enum. Pl. Transssilv.: 300 (1866)

≡ Scabiosa lucida subsp. barbata f. alpicola (Schur) Prodan, Fl. Rep. Pop. Rom. 8: 685 (1961)

= Asterocephalus lucidus [unranked] b. subalpinus Schur, Enum. Pl. Transssilv.: 300 (1866)

≡ Scabiosa lucida subsp. barbata f. subalpina (Schur) Prodan, Fl. Rep. Pop. Rom. 8: 685 (1961)

= Scabiosa lucida f. elata Nyár., Enum. Pl. Vasc. Cheia Turzii: 280 (1939)

= Scabiosa lucida f. hirticaulis Nyár., Enum. Pl. Vasc. Cheia Turzii: 280 (1939)

≡ Scabiosa lucida subsp. barbata f. hirticaulis (Nyár.) Prodan, Fl. Rep. Pop. Rom. 8: 685 (1961)

= Scabiosa lucida f. perramosa Nyár., Enum. Pl. Vasc. Cheia Turzii: 280 (1939)

≡ Scabiosa lucida subsp. barbata f. perramosa (Nyár.) Prodan, Fl. Rep. Pop. Rom. 8: 685 (1961)

= Scabiosa lucida f. scaposa Nyár., Enum. Pl. Vasc. Cheia Turzii: 280 (1939)

= Scabiosa opaca Klokov, Novosti Sist. Vyssh. Nizsh. Rast.: 112 (1974) *

= Scabiosa subalpina Brügger, Jahresber. Nat. Gesell. Graubünd. 29: 137 (1886)

≡ Scabiosa columbaria subsp. subalpina Brügger, Flora Curiensis. Naturgeschichtliche Beiträge zur Kenntniss der Umgebungen von Chur: 65 (1874) [nom. nudum]

≡ Scabiosa columbaria subsp. subalpina (Brügger) Killias, Jahresber. Naturf. Ges. Graubündens 31: 82 (1887–1888)

≡ Scabiosa lucida var. subalpina (Brügger) Hayek & Hegi, Ill. Fl. Mitt.-Eur. 6(1): 308 (1908)

≡ Scabiosa columbaria subsp. lucida var. subalpina (Brügger) Braun-Blanq., Jahresber. Naturf. Ges. Graubünd. 58: 94 (1918)

– Scabiosa lucida Vill., Prosp. Hist. Pl. Dauphiné: 18 (1779) [p. p., tantum quod plantas ucrain. carpat.], non W.T.Aiton *

– Scabiosa lucida subsp. lucida Vill., Prosp. Hist. Pl. Dauphiné: 18 (1779) sensu Tasenkevych [non sensu orig.]

# Pyrola carpatica Holub et Křísa, Folia Geobot. Phytotax. 6(1): 82 (1971)

≡ Pyrola rotundifolia subsp. carpatica (Holub & Křísa) Váczy & Beldie, Fl. Rep. Soc. Rom. 13: 46 (1976)

– Pyrola intermedia auct., non Schleich. ex Arcang.

– Pyrola intermedia Schleich. sensu Szafer in Kulczyński & Pawłowski, Rośliny Polskie: 459 (1924) [nom. illeg.], non Schleich. ex Arcang., Comp. Fl. Ital.: 460 (1882)

– Pyrola rotundifolia [unranked] arenaria Scheele sensu Jáv., Magyar Fl.: 797 (1924)

– Pyrola rotundifolia subsp. intermedia (Alef.) Wohlfahrt in W.D.J.Koch, Syn. Deut. Schweiz. Fl., Bd. 2: 1946 (1902) [p. p., tantum quod plantas carpat.]

– Pyrola rotundifolia subsp. intermedia (Schleich.) Dostál, Květena ČSR: 1115 (1949) [p. p., tantum quod plantas carpat., excl. var. arenaria Koch; nom. illeg.]

# Soldanella hungarica Simonk., Enum Fl. Transsilv.: 461 (1886) et Oesterr. Bot. Z. 39: 219 (1889)

**A. Soldanella hungarica Simonk.**, Enum Fl. Transsilv.: 461 (1886) et Oester. Bot. Z. 39: 219 (1889) [s. str.] *

≡ Soldanella montana subsp. hungarica (Simonk.) Lüdi in Hegi, Illustr. Fl. Mittel-Europa 5(3): 1827 (1927)

≡ Soldanella hungarica subsp. hungarica Simonk., Enum Fl. Transsilv.: 461 (1886) et Oesterr. Bot. Z. 39: 219 (1889) [s. str.]

≡ Soldanella montana var. hungarica (Simonk.) Grinţ., Gen.Soldan. Haretii: 10 (1908)

≡ Soldanella alpina var. hungarica (Simonk.) Stojanoff & Stefanoff, Ann. Arch. Min. Agri. Dom. Roy. Bulg. 5: 865 (1925) [p. p.]

= Soldanella major f. parviflora Morariu in Morariu, Nyár. & Guşul., Fl. Rep. Pop. Rom. 7: 642 (1960) [nom. inval.]

= Soldanella major f. purpureifolia R.Rös., Comun. Bot. 7: 58 (1963) [nom. inval.]

= Soldanella pseudomontana F.K.Meyer, Haussknechtia 2: 15 (1985)

– Soldanella alpina [unranked] minor Clus., Rar. Plant. Hist.: 309 (1601) [p.p.]

≡ Soldanella alpina [unranked] β minor (Clus.) Neilr., Nachtraege Fl. Wien: 219 (1851) et Fl. Wien, Bd. 2: 219 (1868) [p. p.], non Seringe *

– Soldanella alpina [unranked] a minor Schur, Enum. Pl. Transsilv.: 556 (1866) [p. p., nom. illeg.], non Seringe

≡ Soldanella montana var. minor (Schur) Borbás, Beih. Bot. Centralb. 10: 282 (1901) [p. p.]

≡ Soldanella hungarica var. minor (Schur) Vierh. in Hannig & Winkler, Pflanzenareale 1(1): Karte 7–8 (1926) [p. p.]

– Soldanella major f. hungarica (Simonk.) Jáv., Fl. Hung.: 811 (1925) [p. p.]

– Soldanella major f. macrocarpa Morariu in Morariu, Nyár. & Guşul., Fl. Rep. Pop. Rom. 7: 642 (1960) [p. p., nom. inval.]

– Soldanella montana subsp. hungarica var. minor (Schur) Vierch. [nom. inval., ex herb. LWS] *

– Soldanella montana var. hungarica f. minor (Schur) G.Kozij [nom. inval., ex herb. LWS] *

**B. Soldanella major (Neilr.) Vierh. in Urban & Graebn.**, Festschr. Asch.: 502 (1904), **emend. Zhang & Kadereit**, Nordic J. Bot. 22(2): 153 (2002) *

≡ Soldanella alpina [unranked] α major Neilr., Nachtraege Fl. Wien: 219 (1851) et Fl. Wien, Bd. 2: 219 (1868) *

≡ Soldanella montana subsp. hungarica var. major (Neilr.) Lüdi in Hegi, Illustr. Fl. Mittel-Europa 5(3): 1827 (1930)

= Soldanella stiriaca F.K.Meyer, Haussknechtia 2: 20 (1985) [nom. inval., superfl.]

– Soldanella hungarica subsp. major (Neilr.) Pawłowska, Fragm. Florist. Geobot. 9: 11 (1963) [p. p.] *

– Soldanella alpina var. vulgaris Seringe, Mus. Helv. Hist. Nat., Ser. Bot. 1: 83 (1823) [p. p., nom. inval.]

– Soldanella major subsp. margittaniana Fodor [nom. nudum, ex herb. UU] *

**C. Soldanella marmarossiensis Klášt.**, Preslia 9: 19 (1930), **emend. Zhang & Kadereit**, Nordic J. Bot. 22(2): 148 (2002) *

≡ Soldanella richteri subsp. marmarossiensis (Klášt.) Niederle, Skalnickáruv rok 75: 27 (2017)

= Soldanella haretii Grinţ., Gen. Soldan. Soldan. Haretii: 7 (1908)

≡ Soldanella major f. haretii (Grinţ.) Guşul. in Morariu, Nyár. & Guşul., Fl. Rep. Pop. Rom. 7: 67 (1960)

= Soldanella montana var. repanda Grinţ., Gen. Soldan. Soldan. Haretii: 12 (1908)

– Soldanella montana subsp. faceta A.Kress, Primulaceen-Studien 11: 22 (1993) [p. p.]

– Soldanella montana subsp. hungarica var. marmarossiensis (Klášt.) Fodor, Fl. Zakarpattia: 57 (1974) [p. p.]

# Genista tinctoria subsp. oligosperma (Andrae) Soó, Feddes Repert. 83(3): 169 (1972)

≡ Genista tinctoria var. oligosperma Andrae, Bot. Zeitung 11: 440 (1853)

≡ Genista oligosperma (Andrae) Simonk., Enum. Fl. Transsilv.: 169 (1886) *

≡ Genista tinctoria subsp. oligosperma (Andrae) Malinovsky, Ukr. Bot. J. 19(3): 75 (1962) [comb. invalid.]

= Genista alpicola Schur, Enum. Pl. Transsilv.: 145 (1866)

≡ Genista oligosperma f. alpicola (Schur) Morariu, Fl. Rep. Pop. Rom. 5: 62 (1957)

= Genista oligosperma f. ghisae Pawł., Bul. Grăd. Bot. Cluj 19: 6 (1939)

= Genista rupestris Schur, Enum. Pl. Transsilv.: 145 (1866) *

= Genista sigeriana Fuss, Fl. Transsilv.: 149 (1866)

= Genista tinctoria var. prostrata auct., non Bab.

– Genista procumbens Baumg. ex Fuss, Fl. Transsilv.: 150 (1866) [nom. inval.], non alior

# Lathyrus transsilvanicus (Spreng.) Rchb.f., Icon. Fl. Germ. Helv. 22: t. 220, fig. 4, nr. 8-12 (1886)

≡ Orobus transsylvanicus Spreng., Syst. Veg., ed. 16, 3: 260 (1826)

≡ Orobus luteus subsp. transsylvanicus (Spreng.) Nyman, Consp. Fl. Eur. 1: 204 (1878) [nom. et. des. inval.]

≡ Lathyrus transsilvanicus (Spreng.) R.M.Fritsch, Sitzungsber. Kaiserl. Akad. Wiss., Math.-Naturwiss. Cl., Abt. 1, 104: 517 (1895) [nom. inval.]

≡ Lathyrus linnaei f. transsilvanicus (Spreng.) Rouy in Rouy & Foucad, Fl. France 5: 269 (1899)

≡ Lathyrus luteus [unranked] c transsylvanicus (Spreng.) Beck in Rchb., Icon. Fl. Germ. Helv. 22: 155 (1903)

≡ Lathyrus luteus [unranked] a transsilvanicus (Spreng.) Ascherson & Graebn., Syn. Mitteleur. Fl. 6(2): 1044 (1906–1910)

≡ Lathyrus laevigatus subsp. transsylvanicus (Spreng.) Breistr., Bull. Soc. Bot. France 87: 53 (1940)

≡ Lathyrus luteus subsp. transsylvanicus (Spreng.) Dostal, Květena ČSR: 821 (1949)

= Lathyrus transsilvanicus f. trichocarpus Borbás in Nyár., Herb. Kv. fl.: 335 (1941-1944)

– Orobus laevigatus Baumg., Enum. stirp. Transsilv. 2: 329 (1816), non Waldst. & Kit.

# Trifolium sarosiense Hazsl., Éjsz. Magyarh. Vir.: 76 (1864) et Hazsl. ex Neilr., Diagn. Gefaesspfl.: 35 (1864)

≡ Trifolium medium subsp. sarosiense (Hazsl.) Simonk., Enum. Fl. Transsilv.: 180 (1887) *

≡ Trifolium flexuosum subsp. sarosiense (Hazsl.) Gibelli & Belli, Mem. Reale Accad. Sci. Torino ser. 2, 39 (1): 333 (1889)

≡ Trifolium medium var. sarosiense (Hazsl.) A.Nyár. in Săvul., Fl. Rep. Pop. Rom. 5: 208 (1952)

= Trifolium medium var. banaticum Heuff., Verh. K.K. Zool.-Bot. Ges. Wien 8(Abh.): 89 (1858)

≡ Trifolium banaticum (Heuff.) Májovský, Acta Fac. Rerum Nat. Univ. Comen., Bot. 35: 6 (1988)

≡ Trifolium medium subsp. banaticum (Heuff.) Hendrych, Preslia 28: 405 (1956)

≡ Trifolium sarosiense subsp. banaticum (Heuff.) Holub, Folia Geobot. Phytotax. 18(2): 205 (1983)

= Trifolium medium var. sarosiense f. bracteolatum A.Nyár. in Săvul., Fl. Rep. Pop. Rom. 5: 208, 540 (1952)

= Trifolium medium var. sarosiense f. eciliatum A.Nyár. in Săvul., Fl. Rep. Pop. Rom. 5: 208, 540 (1952)

= Trifolium medium [unranked] e humile Schur, Enum. Pl. Transsilv.: 155 (1866)

# Gentiana laciniata Kit. ex Kanitz, Verh. Zool.-Bot. Ges. Wien 12: 572 (1862)

≡ Gentiana pyrenaica var. laciniata (Kit. ex Kanitz) Jáv., Shed. Fl. Hung. Exs. 8: Nr 786 (1927) *

≡ Ciminalis dshimilensis subsp. laciniata (Kit. ex Kanitz) Zuev, Turczaninowia 22(3): 147 (2019)

= Gentiana vagneriana Janka, Oesterr. Bot. Z. 35: 109 (1885)

≡ Gentiana wagneri Janka, Oesterr. Bot. Z. 35: 109 (1885) [nom. inval.; ortho. var.]

– Gentiana pyrenaica auct. fl. ucrain. carpat., non L. *

# Swertia punctata Baumg., Enum. Stirp. Transsilv. 1: 190 (1816)

≡ Swertia perennis subsp. punctata (Baumg.) Ciocârlan, Fl. Ilustr. Rom. Vol. 2: 104 (1990), non S. dichotoma var. punctata T.N.Ho & J.X.Yang

= Swertia perennis M.Bieb. ex Boiss., Fl. Orient. [Boissier] 4(1): 78 (1879), non L.

= Swertia stigmantha K.Koch, Linnaea 23: 586 (1850)

– Swertia perennis L., Sp. Pl. 1: 226 (1753) [p. p. minor]

# Galium album subsp. suberectum (Klokov) Michálk., Karpatskaja Fl.: 78 (1988) et Biología, Bot. (Czechoslovakia) 48(1): 48 (1993)

≡ Galium suberectum Klokov, Fl. UkrSSR 10: 463 (1961) *

≡ Galium erectum subsp. suberectum (Klokov) Kobiv et al., Visnyk Lviv Univ., Ser. Biol. 49: 68 (2009) [nom. illeg.]

= Galium mollugo subsp. erectum f. longifolium Kucowa in Pawł., Fl. Polska 11: 311, 324 (1967)

– Galium erectum auct. fl. ucrain. carpat., non Huds.

– Galium mollugo subsp. erectum (Huds.) Syme sensu Kucowa in Pawł., Fl. Polska 11: 311, 324 (1967) [p. p.]

# Galium transcarpaticum Stojko et Tasenk., Ukr. Bot. J. 36(6): 594 (1979)

[no known synonyms]

# Thymus alternans Klokov, Bot. Mater. Gerb. Bot. Inst. Komarova Akad. Nauk SSSR 16: 293 (1954)

– Thymus marschallianus auct., non Willd. *

– Thymus glabrescens auct., non Willd.

– Thymus serpyllum f. margittaianus auct., non Lyka in Jáv.

– Thymus roegneri K.Koch, Linnaea 21(6): 666 (1849) [p. p., tantum quod plantas ucrain. carpat.] *

≡ Thymus serpyllum var. roegneri (K.Koch) Nyman, Consp. Fl. Eur. Suppl. 2: 257 (1890) [p. p., tantum quod plantas ucrain. carpat.]

# Thymus pulcherrimus subsp. pulcherrimus Schur, Verh. Mitth. Siebenbürg. Vereins Naturwiss. Hermannstadt 10: 140 (1859) et Enum. Pl. Transssilv.: 526 (1866)

≡ Thymus pulcherrimus Schur, Verh. Mitth. Siebenbürg. Vereins Naturwiss. Hermannstadt 10: 140 (1859) [s. str.] *

≡ Thymus chamaedrys subsp. pulcherrimus (Schur) Simonk., Enum. Fl. Transsilv.: 442 (1886)

≡ Thymus serpyllum var. pulcherrimus (Schur) Nyman, Consp. Fl. Eur., Suppl. 2: 257 (1890)

≡ Thymus serpyllum subsp. pulcherrimus (Schur) Lyka in Jáv., Magyar Fl.: 902 (1925)

= Thymus rotundifolius Schur, Verh. Mitth. Siebenbürg. Vereins Naturwiss. Hermannstadt 1: 108 (1850), non alior

= Thymus serpyllum f. oreades Lyka ex Jáv., Magyar Fl.: 902 (1925)

≡ Thymus pulcherrimus var. oreades (Lyka) Borza, Consp. Fl. Rom.: 233 (1947)

≡ Thymus pulcherrimus f. oreades (Lyka) Guşul. in Săvul., Fl. Rep. Pop. Rom. 8: 330 (1961)

= Thymus pulcherrimus f. beldiei Guşul. in Săvul., Fl. Rep. Pop. Rom. 8: 689 (1961) [nom. invalid.]

= Thymus circumcinctus Klokov, Bot. Mater. Gerb. Bot. Inst. Komarova Akad. Nauk SSSR 16: 294 (1954) *

– Thymus carpathicus auct fl. ucrain. carpat., non Čelak. *

– Thymus montanus auct., non Waldst. & Kit.

– Thymus sudeticus Opiz ex Rchb., Fl. Germ. Excurs.: 312 (1830–1832) et Opiz ex Borbás, Math. Term. Közlem. 24(2): 103 (1890) [p. p., tantum quod plantas ucrain. carpat.] *

# Syringa josikaea J.Jacq. ex Rchb.f., Iconogr. Bot. Pl. Crit. 8: 32 (1830) et J.Jacq., Flora 14(1): 67, 399 (1831)

= Syringa josikaea [unranked] eximia hort. ex Beissner, Schelle & Zabel, Handb. Laubholzben.: 415 (1903)

≡ Syringa josikaea var. eximia Froebel ex Olbrich, Möller’s Deutsche Gärtn.-Zeitung 16: 561 (1901)

≡ Syringa henryi var. eximia Rehder, Mitt. Deutsch. Dendrol. Ges. 24: 227 (1915)

= Syringa josikaea f. monstrosa Jägger ex Morariu, Fl. Rep. Pop. Rom. 8: 513 (1961)

= Syringa josikaea [unranked] pallida hort. ex Beissner, Schelle & Zabel, Handb. Laubholzben.: 415 (1903)

≡ Syringa josikaea f. pallida Jägger ex Morariu, Fl. Rep. Pop. Rom. 8: 513 (1961)

= Syringa josikaea f. rosea Miemetz ex Morariu, Fl. Rep. Pop. Rom. 8: 513 (1961)

= Syringa josikaea [unranked] rubra hort. ex Beissner, Schelle & Zabel, Handb. Laubholzben.: 415 (1903)

≡ Syringa josikaea f. rubra hort. ex Morariu, Fl. Rep. Pop. Rom. 8: 513 (1961)

= Syringa josikaea f. simia Froebel ex Morariu, Fl. Rep. Pop. Rom. 8: 513 (1961)

= Syringa josikaea [unranked] zabeli hort. ex Beissner, Schelle & Zabel, Handb. Laubholzben.: 415 (1903)

≡ Syringa josikaea f. zabelii Froebel ex Morariu, Fl. Rep. Pop. Rom. 8: 513 (1961)

= Syringa prunifolia Kit. ex Lingelsh., Pflanzenr. [Engler] 72: 78 (1920)

= Syringa vincetoxifolia Baumg. ex Steud., Nomencl. Bot., ed. 2 2: 656 (1841)

# Euphrasia tatrae Wettst., Oesterr. Bot. Z. 44: 248 (1894)

≡ Euphrasia minima subsp. tatrae (Wettst.) Hayek in Hegi, Ill. Fl. Mitteleur. 6(1): 91 (1913) *

≡ Euphrasia minima var. tatrae (Wettst.) Pawł., Fl. Polska 11: 17 (1967)

= Euphrasia minima var. carpathica Freyn in Sagorski & Schneider, Fl. Centralkarpat. 2: 421 (1891) non Euphrasia carpatica Zapał.

= Euphrasia tatrae subsp. glandulifera (Wettst.) Staszk., Fragm. Florist. Geobot. 22(2): 292 (2015)

= Euphrasia tatrae f. glandulifera Wettst., Monogr. Gatt. Euphrasia: 165 (1896)

≡ Euphrasia minima var. tatrae f. glandulifera (Wettst.) Răvăruţ, Fl. Rep. Pop. Rom. 7: 586 (1960)

≡ Euphrasia officinalis [unranked] δ alpestris Freyn, Verh. K.K. Zool.-Bot. Ges. Wien 22: 350 (1872), non Günther, Grab. & Wimm.

# Plantago atrata subsp. carpatica (Pilg.) Soó, Acta Geobot. Hung. 3: 61 (1940)

≡ Plantago montana subsp. atrata var. carpathica Pilg., Repert. Spec. Nov. Regni Veg. 23: 256 (1926–1927)

≡ Plantago atrata subsp. atrata var. carpathica (Pilg.) Pilg., Pflanzenr. (Engler) 102: 296 (1937)

≡ Plantago montana subsp. carpatica (Pilg.) Soó ex Balázs, Acta Geobot. Hung. 2: 40 (1938–1939)

= Plantago lanceolata [unranked] β alpestris Wahlenb., Fl. Carpat. Princip.: 44 (1814)

= Plantago montana [unranked] alpestre Wahlenb., Fl. Carpat. Princip.: 44 (1814)

= Plantago montana subsp. atrata var. carpathica subvar. rigidior Pilg., Repert. Spec. Nov. Regni Veg. 23: 257 (1926–1927)

≡ Plantago atrata subsp. atrata var. carpathica subvar. rigidior (Pilg.) Pilg., Pflanzenr. (Engler): 296 (1937)

≡ Plantago montana subsp. carpatica subvar. rigidior (Pilg.) Balázs, Acta Geobot. Hung. 2: 40 (1938–1939)

= Plantago montana subsp. atrata var. carpathica subvar. vestita Pilg., Repert. Spec. Nov. Regni Veg. 23: 257 (1926–1927)

≡ Plantago atrata subsp. carpathica f. vestita (Pilg.) Soó, Acta Geobot. Hung. 3: 61 (1940)

≡ Plantago atrata subsp. atrata var. carpathica subvar. vestita (Pilg.) Pilg., Pflanzenr. (Engler): 296 (1937)

≡ Plantago montana subsp. carpatica subvar. vestita (Pilg.) Balázs, Acta Geobot. Hung. 2: 40 (1938–1939)

≡ Plantago atrata var. carpathica f. vestita (Pilg.) Borza, Consp. Fl. Rom.: 255 (1949)

– Plantago alpina Vill. sensu Rochel, Pl. Banat. Rar.: 32; Nr. 4, Tab. 1, fig. 4 (1828)

– Plantago alpina Vill. sensu Schur, Enum. Pl. Transsilv.: 564 (1866), non alior

– Plantago atrata Hoppe, Bot. Taschenb. 1799: 85 (1799) [p. p., tantum quod plantas ucrain. carpat.] *

– Plantago montana Lam. sensu Schur, Enum. Pl. Transsilv.: 564 (1866), non alior *

– Plantago saxatilis M.Bieb., Fl. Taur.-Caucasus 1: 109 (1808) [p. p.]

# Melampyrum saxosum Baumg., Enum. Stirp. Transsilv. 2: 199 (1816)

≡ Melampyrum sylvaticum [unranked] M. saxosum (Baumg.) Nyman, Consp. Fl. Eur.: 556 (1881)

≡ Melampyrum sylvaticum subsp. saxosum (Baumg.) G.Beauvis., Bull. Soc. Bot. Geneve 4: 418 (1912) et Mem. Soc. Phys. Hist. Nat. Geneve 38(6): 581 (1916) *

= Melampyrum herbichii Woł., Spraw Kom. Fizyi. Krajow. 21: 133 (1888) *

= Melampyrum sylvaticum subsp. saxosum var. herbichii (Woł.) G.Beauvis., Mem. Soc. Phys. Hist. Nat. Geneve 38: 582 (1916)

= Melampyrum herbichii subsp. woloszczakii Jasiewicz, Fragm. Florist. Geobot. 4: 112 (1958)

= Melampyrum saxosum [unranked] baumgartenii Soó ex Jáv., Magyar Fl.: 1011 (1925)

≡ Melampyrum saxosum subsp. baumgartenii (Soó) Soó, Feddes Repert. 24: 176 (1927)

≡ Melampyrum saxosum var. baumgartenii (Soó) Nyár., Flora Rep. Pop. Rom. 7: 637, 646 (1960)

= Melampyrum saxosum [unranked] javorkae Soó ex Jáv., Magyar Fl.: 1011 (1925)

≡ Melampyrum saxosum subsp. javorkae (Soó) Soó, Feddes Repert. 24: 176 (1927)

≡ Melampyrum saxosum var. javorkae (Soó) Nyár., Flora Rep. Pop. Rom. 7: 637, 646 (1960)

= Melampyrum saxosum var. typicum Nyár., Flora Rep. Pop. Rom. 7: 637, 646 (1960)

= Melampyrum sylvaticum f. csatoi Soó, Feddes Repert. 24: 174 (1927)

≡ Melampyrum herbichii subsp. csatoi (Soó) Soó, Feddes Repert. 83(3): 181 (1972)

= Melampyrum sylvaticum subsp. moeszianum Soó, Feddes Repert. 24: 190 (1927)

= Melampyrum sylvaticum [unranked] α pictum Herbich, Select. Pl. Rar. Galic. Bucov.: Nr 39 (1836) et Fl. Bucov.: 275 (1859)

≡ Melampyrum sylvaticum subsp. saxosum var. β pictum (Herbich) G.Beauvis., Mem. Soc. Phys. Hist. Nat. Geneve 38: 581 (1916)

≡ Melampyrum sylvaticum subsp. saxosum var. pictum subvar. eu-pictum G.Beauvis., Mem. Soc. Phys. Hist. Nat. Geneve 38: 582 (1916)

= Melampyrum sylvaticum var. β saxosum Willkomm, Führer Pfl. Deutsch., Österr. und Schweiz: 535 (1881)

≡ Melampyrum sylvaticum subsp. saxosum var. pictum subvar. eu-saxosum G.Beauvis., Mem. Soc. Phys. Hist. Nat. Geneve 38: 582 (1916)

– Melampyrum pictum Herbich [nom inval., ex herb LWS] *

– Melampyrum sylvaticum Simonk., Enum. Fl. Transsilv.: 429 (1886) [p. p.], non L.

# Linum extraaxillare Kit. ex Rochel, Pl. Banat. Rar.: 26 (1828) [nom. nudum] et Kit., Linnaea 32(4-5): 573 (1864)

≡ Linum perenne subsp. extraaxillare (Kit. ex Rochel) Nyman, Consp. Fl. Eur., Suppl. 2: 71 (1889)

– Linum montanum auct. fl. transsilv., non Schleich.

– Linum alpinum auct. fl. transsilv., non L.

# Salix kitaibeliana Willd., Sp. Pl., ed. 4 [Willdenow] 4(2): 683-684 (1806)

≡ Salix retusa [unranked] γ kitaibeliana (Willd.) Rchb., Reichenbachianae Fl. German.: 15 (1833) et Icon. Fl. Germ. Helv. 11: 16, fig. 1187 (1849)

≡ Salix retusa f. kitaibeliana (Willd.) Rouy, Fl. France [Rouy & Foucaud] 12: 219 (1910) *

≡ Salix retusa subsp. kitaibeliana (Willd.) Jáv., Magyar Fl.: 235 (1924) *

= Salix retusa [unranked] b serrulata Roch., Pl. Banat.: 78, tab. 38, fig. 80 (1828)

≡ Salix retusa var. serrulata Roch. ex Rchb., Fl. Germ. Excurs.: 166 (1831)

= Salix retusa var. major Rchb., Fl. Germ. Excurs.: 166 (1830–1832)

= Salix retusa [unranked] β major W.D.J. Koch, Syn. Fl. Germ. Helv.: 660 (1837) [nom. superfl.]

# Viola declinata Waldst. et Kit., Descr. Icon. Pl. Rar. Hung. 3: 248 (1807)

= Viola declinata var. knechtelii Grec., Consp. Fl. Rom.: 88 (1898)

= Viola declinata [unranked] b montana Schur, Enum. Pl. Transsilv.: 86 (1866)

= Viola gracilis Rchb., Fl. Germ. Excurs. 709 (1832), non alior

= Viola mutabilis [unranked] b intermedia Roch., Enum. Pl. Banat.: 6 (1828) [nom. nudum]

= Viola mutabilis [unranked] e major Roch., Enum. Pl. Banat.: 6 (1828) [nom. nudum]

≡ Viola declinata var. major (Roch.) Grec., Consp. Fl. Rom.: 88 (1898)

# Aconitum bucovinense Zapał., Rozpr. Wydz. Mat.-Przyr. Akad. Umiej., Dział B. Nauki Biol. 48: 8990 (1908)

≡ Aconitum firmum subsp. bucovinense (Zapał.) Aschers. & Graebn., Syn. Mitteleurop. Fl. 5/2: 781 (1929)

≡ Aconitum callibotryon subsp. bucovinense (Zapał.) Grinţ., Fl. Rep. Pop. Rom. 2: 481 (1953)

= Aconitum bucovinense f. orthotricha Gáyer, Magyar Bot. Lap. 8: 168 (1909)

= Aconitum callibotryon subsp. bucovinense f. altum Grinţ., Fl. Rep. Pop. Rom. 2: 482, 685 (1953)

= Aconitum callibotryon subsp. bucovinense f. densum Grinţ., Fl. Rep. Pop. Rom. 2: 482, 685 (1953)

= Aconitum callibotryon subsp. bucovinense f. glaberrimum Grinţ., Fl. Rep. Pop. Rom. 2: 482, 684 (1953)

= Aconitum callibotryon subsp. bucovinense f. laxum Grinţ., Fl. Rep. Pop. Rom. 2: 482, 685 (1953)

= Aconitum callibotryon subsp. bucovinense f. pilosum Grinţ., Fl. Rep. Pop. Rom. 2: 482, 684 (1953)

= Aconitum callibotryon subsp. bucovinense f. pyramidatum Grinţ., Fl. Rep. Pop. Rom. 2: 482, 685 (1953)

= Aconitum callibotryon subsp. rigidum f. glabrum Grinţ., Fl. Rep. Pop. Rom. 2: 482, 683 (1953)

= Aconitum callibotryon subsp. rigidum f. pubescens Grinţ., Fl. Rep. Pop. Rom. 2: 482, 684 (1953)

= Aconitum commutatum Rchb., Uebers. Aconitum: 36 (1819)

= Aconitum laetum [unranked] β rigidum Rchb., Icon. Fl. Germ. Helv. 4: 25, tab. 97, fig. 4708b (1840)

≡ Aconitum firmum f. rigidum (Rchb.) Gáyer, Magyar Bot. Lapok 8: 165 (1909)

≡ Aconitum callibotryon subsp. rigidum (Rchb.) Grinţ., Fl. Rep. Pop. Rom. 2: 482 (1953)

= Aconitum napellus f. commutatum (Rchb.) Gáyer in G. Hegi, Ill. Fl. Mitt.-Eur. 3: 499 (1912)

– Aconitum bernhardianum Rchb., Uebers. Aconitum: 34 (1819) et Illustrat. Spec. Aconitum: tab. 68 (1823–1827), non Wallr.

# Aconitum firmum subsp. firmum Rchb., Uebers. Aconitum: 20 (1819)

≡ Aconitum koelleanum var. firmum (Rchb.) Rchb., Mon. Aconitum: 85, Tab. 14, fig. 1 (1821)

≡ Aconitum napellus subsp. firmum (Rchb.) Gáyer in G.Hegi, Ill. Fl. Mitt.-Eur. 3: 498 (1912)

≡ Aconitum napellus var. firmum (Rchb.) Pawł., FI. Tatr. 1: 274 (1956) *

= Aconitum napellus [unranked] e babiogorense Zapał., Consp. FI. Gal. Crit. 2: 226 (1908)

≡ Aconitum napellus [unranked] e babiogorense f. babiogorense Zapał., Consp. FI. Gal. Crit. 2: 226 (1908)

= Aconitum napellus [unranked] e babiogorense f. subfissum Zapał., Consp. FI. Gal. Crit. 2: 227 (1908)

= Aconitum napellus [unranked] d carpaticum f. carpaticum Zapał., Consp. FI. Gal. Crit. 2: 226 (1908)

= Aconitum napellus [unranked] b subtatrense Zapał., Consp. FI. Gal. Crit. 2: 225 (1908)

≡ Aconitum napellus [unranked] b subtatrense f. subtatrense Zapał., Consp. FI. Gal. Crit. 2: 225 (1908)

= Aconitum napellus [unranked] b subtatrense f. abnorme Zapał., Consp. FI. Gal. Crit. 2: 225 (1908)

= Aconitum napellus [unranked] b subtatrense f. latisectum Zapał., Consp. FI. Gal. Crit. 2: 225 (1908)

= Aconitum napellus [unranked] g tatrense Zapał., Consp. FI. Gal. Crit. 2: 227 (1908)

– Aconitum palmatifidum Rchb., Uebers. Gat. Aconitum: 48 (1819) [p. p.]

– Aconitum skerisorae auct [e.g., Seitz, Soó], non Gáyer *

– Aconitum tatrae Borbás in Pallas, Nagy Lexikona 15: 15 (1897) [p. p.]

– Aconitum tauricum auct. fl. carpat., non Wulfen

# Aconitum firmum subsp. fissurae Nyár., Enum. Pl. Cheia Turzii: 132 (1939)

≡ Aconitum napellus subsp. fissurae (Nyár.) W.Seitz, Feddes Repert. 80: 42 (1969)

= Aconitum flerovii Steinb. in Komarov, Fl. USSR 7: 221, 730 (1937)

= Aconitum hunyadense Degen, Magyar Bot. Lapok 5: 196 (1906)

≡ Aconitum tatrae subsp. hunyadense (Degen) Soó, Feddes Repert. 83: 135 (1972)

= Aconitum romanicum Woł., Fl. Polon. Exsicc. no. 905. *

# Aconitum degenii subsp. degenii Gáyer, Magyar Bot. Lapok 5: 123 (1906)

= Aconitum degenii f. craciunelense Gáyer, Magyar Bot. Lap. 5: 126 (1906)

= Aconitum molle Rchb., Uebers. Gat. Aconitum: 47 (1819)

= Aconitum paniculatum [unranked] b czeremossicum Zapał., Consp. Fl. Gal. Crit. 2: 220 (1908)

= Aconitum paniculatum [unranked] d intermedium Zapał., Consp. Fl. Gal. Crit. 2: 221 (1908)

= Aconitum paniculatum f. latiusculum Zapał., Consp. Fl. Gal. Crit. 2: 220 (1908)

= Aconitum paniculatum [unranked] a percalabense Zapał., Consp. Fl. Gal. Crit. 2: 220 (1908)

= Aconitum paniculatum [unranked] c prutense Zapał., Consp. Fl. Gal. Crit. 2: 221 (1908)

≡ Aconitum prutense (Zapał.) Tzvelev, Bot. Zhurn. (Moscow & Leningrad) 81(12): 115 (1997) *

= Aconitum paniculatum [unranked] c prutense f. lobatum Zapał., Consp. Fl. Gal. Crit. 2: 221 (1908)

= Aconitum paniculatum [unranked] c prutense f. subintermedium Zapał., Consp. Fl. Gal. Crit. 2: 221 (1908)

= Aconitum paniculatum f. tenuifissum Zapał., Consp. Fl. Gal. Crit. 2: 220 (1908)

– Aconitum hebegynum auct. fl. carpat., non DC. [p. p.] *

– Aconitum paniculatum Lam., Fl. Fr. 3: 646 (1778) [p. p., nom. inval.] *

– Cammarum paniculatum (Arcang.) Fourr., Ann. Soc. Linn. Lyon sér. 2 16: 327 (1868) [p. p.]

– Delphinium paniculatum (Arcang.) E.H.L.Krause, Deutschl. Fl. (Sturm), ed. 2. 5: 234 (1901) [p. p.], non Host

# Aconitum lasiocarpum subsp. kotulae (Pawł.) Starm. & Mitka, Acta Soc. Bot. Polon. 69(2): 150 (2000)

≡ Aconitum variegatum subsp. kotulae Pawł., FI. Tatr 1: 275 (1956)

≡ Aconitum variegatum f. kotulae (Pawł.) Skalický, Preslia 54(2): 119 (1982)

= Aconitum cammarum [unranked] a beskidense Zapał., Consp. FI. Gal. Crit. 2: 215 (1908)

≡ Aconitum beskidense (Zapał.) Gáyer, Magyar Bot. Lapok 10: 201 (1911)

≡ Aconitum gracile subsp. grosserratum f. beskidense (Zapał.) Grinț., FI. Rep. Pop. Rom. 2: 485 (1953)

= Aconitum cammarum [unranked] c koscieliskanum Zapał., Consp. Fl. Gallic. Crit., 2: 215 (1908)

= Aconitum paniculatum [unanked] e podolicum Zapał., Consp. FI. Gal. Crit. 2: 221 (1908)

≡ Aconitum podolicum (Zapał.) Voroshylov, Bjul. Glav. Bot. Sada 158: 39 (1990) *

= Aconitum paniculatum [unanked] e podolicum f. latilobum Zapał., Consp. FI. Gal. Crit. 2: 222 (1908)

– Aconitum lasiocarpum Rchb., Uebers. Gat. Aconitum.: 55 (1819) [p. p.]

# Aconitum lasiocarpum subsp. lasiocarpum (Rchb.) Gáyer, Magyar Bot. Lapok 11: 199 (1911)

≡ Aconitum nasutum var. lasiocarpum Rchb., Illustr. Spec. Aconitum: Nr. 47, Tab. 9 (1823–1827)

≡ Aconitum paniculatum subsp. lasiocarpum (Rchb.) Soó, Acta Bot. Hung. 5: 213 (1943)

≡ Aconitum toxicum subsp. lasiocarpum (Rchb.) Grinț., FI. Rep. Pop. Rom. 2: 491 (1953)

= Aconitum toxicum [unranked] a dasycarpum Schur, Enum. Pl. Transsilv.: 33 (1886)

≡ Aconitum dasycarpum (Schur) Schur ex Gáyer, Magyar Bot. Lapok 10: 199 (1911)

= Aconitum vagneri Kern. ex Gáyer, Magyar Bot. Lapok 10: 199 (1911)

– Aconitum lasiocarpum Rchb., Uebers. Gat. Aconitum.: 55 (1819) [p. p., nom. nudum]

# Aconitum moldavicum subsp. hosteanum (Schur) Graebn. et P.Graebn., Syn. Mitteleur. Fl. 5(2): 725 (1929)

≡ Aconitum hosteanum Schur, Verh. Mitth. Siebenbürg. Vereins Naturwiss. Hermannstadt 2: 177 (1851) [nom. nudum] et Verh. Mitth. Siebenbürg. Vereins Naturwiss. Hermannstadt 3: 84 (1852) [nom. nudum] et Verh. Mitth. Siebenbürg. Vereins Naturwiss. Hermannstadt 4: 49 (1853) *

≡ Aconitum moldavicum [unranked] e hosteanum (Schur) Zapał., Consp. Fl. Gal. Crit. 2: 213 (1908)

= Aconitum hosteanum f. borbasii Gáyer, Magyar Bot. Lapok 8: 316 (1909)

= Aconitum hosteanum var. geraniifolium Grinț. in Săvul., Fl. Rep. Pop. Rom. 2: 499, 678 (1953)

= Aconitum moldavicum var. australe f. fragile Grinț. in Săvul., Fl. Rep. Pop. Rom. 2: 496, 676 (1953)

= Aconitum moldavicum var. australe f. obtusidentatum Simonk. ex Gáyer, Magyar Bot. Lap. 8: 315 (1909)

= Aconitum moldavicum [unranked] a dissectifolium Zapał., Consp. Fl. Gal. Crit. 2: 212 (1908)

≡ Aconitum moldavicum var. australe f. dissectifolium (Zapał.) Grinț. in Săvul., Fl. Rep. Pop. Rom. 2: 497 (1953)

= Aconitum moldavicum [unranked] b grandicassum Zapał., Consp. Fl. Gal. Crit. 2: 212 (1908)

= Aconitum moldavicum [unranked] c grandiflorum Schur, Enum. Pl. Transsilv.: 32 (1866)

≡ Aconitum moldavicum var. australe f. grandiflorum (Schur) Grinț. in Săvul., Fl. Rep. Pop. Rom. 2: 497 (1953)

= Aconitum moldavicum [unranked] d leopoliense Zapał., Consp. Fl. Gal. Crit. 2: 213 (1908)

≡ Aconitum moldavicum var. australe f. leopoliensis (Zapał.) Grinț. in Săvul., Fl. Rep. Pop. Rom. 2: 497 (1953)

= Aconitum thyraicum Błocki, Allg. Bot. Z. Syst. 1: 59 (1895) *

≡ Aconitum moldavicum var. australe f. thyraicum (Błocki) Grinț. in Săvul., Fl. Rep. Pop. Rom. 2: 497 (1953)

= Aconitum moldavicum [unranked] e hosteanum f. czywczynense Zapał., Consp. Fl. Gal. Crit. 2: 213 (1908)

= Aconitum moldavicum [unranked] e hosteanum f. rodnense Zapał., Consp. Fl. Gal. Crit. 2: 213 (1908)

– Aconitum moldavicum Hacq., Reis. Dac. Sarm. Karpathen 1: 169 (1790) et Hacq. ex Rchb., In: Übers. Gen. Acon.: 67 (1819) [p. p.]

≡ Delphinium moldavicum (Hacq.) Bránadza, Prodr. Fl. Rom.: 11 (1879) [p. p., nom. inval.]

– Aconitum moldavicum var. australe (Rchb.) Grinț. in Săvul., Fl. Rep. Pop. Rom. 2: 496 (1953) [p. p.]

# Aconitum moldavicum subsp. moldavicum Hacq. ex Rchb., Uebers. Gat. Aconitum: 67 (1819)

≡ Aconitum lycoctonum subsp. moldavicum (Hacq.) Jalas, Ann. Bot. Fenn. 22(3): 219 (1985)

= Aconitum lycoctonum [unranked] β caeruleum Wahlenb., FI. Carp. Princip.: 163 (1814)

= Aconitum moldavicum subsp. hacquetianum Grinț., Cat. Sem. Grăd. Bot. Bucovin.: 6 (1945) [nom. nudum]

≡ Aconitum moldavicum var. hacquetianum Grinț. in Săvul., Fl. Rep. Pop. Rom. 2: 496 (1953)

= Aconitum moldavicum var. hacquetianum f. flexuosum Grinț. in Săvul., Fl. Rep. Pop. Rom. 2: 498, 677 (1953)

= Aconitum moldavicum var. hacquetianum f. macrocassis Grinț. in Săvul., Fl. Rep. Pop. Rom. 2: 498, 677 (1953)

= Aconitum moldavicum var. hacquetianum f. piliferum Grinț. in Săvul., Fl. Rep. Pop. Rom. 2: 498, 677 (1953)

= Aconitum moldavicum var. rubicundum Borbás, Kárp. Egyl. Ėvk. 5: 247 (1886) et Oesterr. Bot. Z. 36: 318 (1886)

= Aconitum moldavicum f. stenanthum Gáyer, Magyar Bot. Lapok 6: 297 (1907)

= Aconitum septentrionale Baumg., Enum. Stirp. Transsilv. 2: 98 (1816), non Koelle *

= Aconitum transilvanicum Lerchenf. ex Schur., Verh. Mitth. Siebenbürg. Vereins Naturwiss. Hermannstadt 10: 165 (1859)

– Aconitum fallacinum Błocki, Allg. Bot. Z. Syst. 1: 117 (1895) [p. p.]

– Aconitum jacquinianum Host, Fl. Austr. 2: 68 (1831) [quoad pl. carpat.]

– Aconitum moldavicum Hacq., Reis. Dac. Sarm. Karpathen 1: 169 (1790) et Hacq. ex Rchb., Übers. Gen. Acon.: 67 (1819) [p. p. major]

≡ Delphinium moldavicum (Hacq.) Bránadza, Prodr. Fl. Rom.: 11 (1879) [p. p. major, nom. inval.]

– Aconitum moldavicum [unranked] c parvicassum Zapał., Consp. Fl. Gal. Crit. 2: 212 (1908) [p. p.]

– Aconitum moldavicum f. puberulum Zapał., Consp. Fl. Gal. Crit. 2: 212 (1908) [p. p.]

– Aconitum septentrionale [unranked] β carpathicum DC., Syst. Nat. 1: 370 (1818) [p. p.]

≡ Aconitum lycoctonum var. carpaticum (DC.) Ser., Mus. helv. d'hist. nat. 1: 136 (1822) [p. p.]

≡ Aconitum carpaticum (DC.) Sagorski & Schneider, Fl. Centralkarpat.: 45 (1891) [p. p.]

≡ Aconitum lycoctonum subsp. carpaticum (DC.) Dostal, Květ. ČSR 2: 150 (1950) [p. p.]

# Ranunculus carpaticus Herbich, Sel. Pl. Rar. Gallic.: 15 (1836), non Wahlenb. ex Nyman

= Ranunculus aduncus Schur, Enum. Pl. Transsilv. 16 (1866), non Gren. & Godr.

= Ranunculus carpaticus f. anomalus A.Nyár., Fl. Rep. Pop. Rom. 2: 620, 687 (1953)

= Ranunculus carpaticus f. flabellatus A.Nyár., Fl. Rep. Pop. Rom. 2: 620, 687 (1953)

= Ranunculus carpaticus f. plenus Zapał., Consp. Fl. Galic. Crit. 2: 274 (1908)

= Ranunculus carpaticus f. pygmaeus Porcius, Phaner. Năsăud: 152 (1881)

= Ranunculus carpaticus var. rupicolus Zapał., Consp. Fl. Galic. Crit. 2: 274 (1908)

= Ranunculus gouani Baumg., Enum. Stirp. Transsilv. 2: 125 (1816), non alior

= Ranunculus lerchenfeldianus Schur, Verh. Mitth. Siebenbürg. Vereins Naturwiss. Hermannstadt 3: 84 (1852) [nom. nudum] et Verh. Mitth. Siebenbürg. Vereins Naturwiss. Hermannstadt 4: 14 (1853)

= Ranunculus montanus Willd. [unranked] α dentatus Baumg., Enum. Stirp. Transsilv. 2: 124 (1816)

≡ Ranunculus dentatus (Baumg.) Freyn in A.Kern., Sched. Fl. Austro-Hung. 5: 47 (1888) *

= Ranunculus pormbachiensis Lerchenf. ex Schur, Enum. Pl. Transsilv.: 16 (1866)

= Ranunculus schurii Fuss ex Schur, Enum. Pl. Transsilv.: 16 (1866)

= Ranunculus tuberosus Schur, Oesterr. Bot. Z. 11: 82 (1861) et Enum. Pl. Transsilv.: 16 (1866), non alior.

– Ranunculus szurulensis Lerchenf. ex Schur, Verh. Mitth. Siebenbürg. Vereins Naturwiss. Hermannstadt 4: 14 (1853) [p. p.]

# Ranunculus malinovskii Elenevsky et Derv.-Sok., Novosti Sist. Vyssh. Rast. 23: 59 (1986)

= Ranunculus kladnii auct. fl. ucrain. carpat., non Schur *

# Sempervivum carpathicum subsp. carpathicum Wettst. ex Prodan, Fl. Rep. Pop. Rom. 1: 530 (1923)

*≡* Sempervivum montanum subsp. carpaticum Wettst. in Sched., Flora Exs. Austro-Hung. (1913) [nom. nudum]

*≡* Sempervivum carpathicum Wettst. in A.Kern., Sched. Fl. Exs. Austro-Hung. 10: 25. 1913, [nom. nudum] et Wettst. ex Prodan, Fl. Rom. 1: 530 (1923)

*≡* Sempervivum montanum subsp. carpaticum Wettst. ex Hayek in Hegi, Ill. Fl. Mitt.-Eur. 4(2): 554 (1923) [nom. nudum]

*≡* Sempervivum montanum subsp. carpathicum (Wettst. ex Prodan) A.Berger in Engler & Prantl, Nat. Pflanzenfam., ed. 2 18a: 422 (1930)

*≡* Sempervivum montanum var. carpathicum (Wettst. ex Prodan) Praeger, An account of the Sempervivum group: 46 (1932) [comb. inval.]

*≡* Sempervivum montanum subsp. eumontanum var. carpathicum (Wettst. ex Prodan) Domin, Rozpr. Ceské Akad. Ved, Tr. 2, Vedy Mat. Prír. 42(29): 28 (1933)

= Sempervivum montanum f. brachypetalum Domin, Rozpr. Ceské Akad. Ved, Tr. 2, Vedy Mat. Prír. 42(29): 28 (1933)

= Sempervivum montanum f. congestum Domin, Rozpr. Ceské Akad. Ved, Tr. 2, Vedy Mat. Prír. 42(29): 28 (1933)

= Sempervivum montanum var. pallidum Wettst. ex Hayek in Hegi, Ill. Fl. Mitt.-Eur. 4(2): 554 (1923) [nom. inval.]

*≡* Sempervivum montanum f. pallidum (Wettst. ex Hayek) Fiori, Nuov. Fl. Italia 1: 716 (1923)

*≡* Sempervivum montanum f. pallidum (Wettst. ex Hayek) Domin, Rozpr. Ceské Akad. Ved, Tr. 2, Vedy Mat. Prír. 42(29): 28 (1933) [comb. illeg.]

*≡* Sempervivum montanum f. pallidum (Wettst. ex Hayek) Hadrava & Miklánek, Kaktusy (Brno) 43 (Special 1): 11 (2007) [nom. illeg.]

= Sempervivum montanum f. neopallidum Hadrava & Miklánek, Kaktusy (Brno) 43 (Special 1): 11 (2007) [nom. illeg.]

= Sempervivum montanum f. speciosum Domin, Rozpr. Ceské Akad. Ved, Tr. 2, Vedy Mat. Prír. 42(29): 28 (1933)

= Sempervivum montanum f. stenophyllum Domin, Rozpr. Ceské Akad. Ved, Tr. 2, Vedy Mat. Prír. 42(29): 28 (1933)

= Sempervivum montanum var. pallidum Wettst. ex Schinz & R. Keller, Fl. Schweiz (Schinz), ed. 2. 2: 96 (1905)

= Sempervivum wettsteinii subsp. wettsteinii Letz, Vybrané Problémy Taxonomickej Diferenciácie rodov Sempervivum a Jovibarba v Európe, Thèse Bratislava: 184 (1998) [nom. invalid.] *

– Sempervivum arachnoideum auct. [e.g., G.Reuss], non L.

– Sempervivum heterophyllum Jáv., Magyar Fl.: 456 (1925), non Haszl.

– Sempervivum montanum L., Sp. Pl. 1: 465 (1753) [p. p., tantum quod plantas ucrain. carpat.], non alior *

– Sempervivum montanum subsp. debile auct., non (Schott.) Dostál

– Sempervivum montanum subsp. heterophyllum auct., non (Haszl.) Jáv. ex Soó

– Sempervivum montanum subsp. montanum auct. [e.g., Pawłowski, Dostál, Lippert], non L.

# Sempervivum globiferum subsp. preissianum (Domin) M.Werner, Avonia 28(4): 191 (2011)

≡ Sempervivum preissianum Domin, Bull. Internat. Acad. Sc., Prague, 33: 126 (1932) *

*≡* Sempervivum hirtum subsp. preissianum (Domin) Dostál, Květena ČSR: 537 (1948)

≡ Sempervivum soboliferum subsp. preissianum (Domin) Pawłowska, Fl. Polska 7: 48, 294 (1955)

*≡* Jovibarba preissiana (Domin) Omelczuk & Chopik, Bot. Zhurn. 60(8): 1184 (1975) *

*≡* Jovibarba hirta subsp. preissiana (Domin) Soó, Acta Bot. Hung. 23: 380 (1977)

*≡* Jovibarba globifera subsp. preissiana (Domin) Holub, Preslia 70(2): 106 (1998)

*≡* Jovibarba globifera var. preissiana (Domin) Hadrava & Miklánek, Kaktusy (Brno) 43 (Special 1): 28 (2007)

= Sempervivum hirtum f. glabrescens Sabr., Oesterr. Bot. Z. 32: 378 (1882)

*≡* Sempervivum hirtum subsp. glabrescens (Sabr.) Jáv., Magyar Fl. 2: 458 (1924)

= Sempervivum soboliferum subsp. preissianum f. minus Domin ex Pawłowska, Fl. Polska 7: 48 (1955)

= Sempervivum tatrense Domin, Rozpr. České Akad. Věd, Tř. 2, Vědy Mat. Přír. 42/29: 20–21 (1933)

*≡* Sempervivum hirtum subsp. tatrense (Domin) Dostál, Květena ČSR: 537 (1948)

*≡* Sempervivum soboliferum subsp. preissianum var. tatrense (Domin) Pawłowska, Fl. Polska 7: 48, 294 (1955)

*≡* Jovibarba hirta subsp. tatrensis (Domin) Á.Löve & D.Löve, Bot. Not. 114: 53 (1961)

*≡* Jovibarba hirta var. tatrense (Domin) Soó, Feddes Repert. 83(3): 174 (1972)

*≡* Jovibarba hirta var. tatrensis (Dom.) Konop & Bendak, Skalnicky 1981(1): 33 (1981) [nom. illeg.]

*≡* *Jovibarba globifera* var. *tatrensis* (Domin) Konop & Bendak, Skalnicky, 1981(1): 33 (1981) [nom. illeg.]

– Jovibarba globifera subsp. hirta (L.) J.Parn., Bot. J. Lin. Soc. 103(3): 219 (1990) [p. p., tantum quod plantas ucrain. carpat.]

– Sempervivum soboliferum Sims, Bot. Mag. 35: t. 1457 (1812) [p. p., tantum quod plantas ucrain. carpat.], non Fleisch. & Lindem. *

*≡* Jovibarba sobolifera (Sims) Opiz, Seznam: 54 (1852) [p. p., tantum quod plantas ucrain. carpat.] *

# Chrysosplenium alpinum Schur, Verh. Mitth. Siebenbürg. Vereins Naturwiss. Hermannstadt 3(6): 86 (1852) et Verh. Mitth. Siebenbürg. Vereins Naturwiss. Hermannstadt 10: 133 (1859)

≡ Chrysosplenium oppositifolium var. alpinum Schur, Verh. Mitth. Siebenbürg. Vereins Naturwiss. Hermannstadt, 4(8): 28 (1853) et Enum. Pl. Transsilv.: 241 (1866) *

= Chrysosplenium glaciale Fuss, Fl. Transs.: 247 (1866) *

= Chrysosplenium rosulare Schott ex Maxim., Gartenflora 6: 115 (1857) [nom. nudum] et Bull. Acad. Imp. Sci. Saint-Pétersbourg 23: 345 (1877)

≡ Chrysosplenium oppositifolium var. rosulare (Schot) Schott ex Engl., Nat. Pflanzenfam. ed. 2, 18a: 165 (1930)

= Chrysosplenium transsilvanicum Schur, Verh. Mitth. Siebenbürg. Vereins Naturwiss. Hermannstadt 4(8): 28 (1853) et Enum. Pl. Transsilv.: 241 (1866)

– Chrysosplenium oppositifolium auct. fl. roman. et ucrain. [e.g., Baumg., Enum. Stirp. Transsilv. 1: 338, Nr. 699 (1816)], non L. *
